# A Decarbonylative Strategy to Enhance Efficiency and Regioselectivity in Photocatalyzed Hydrogen Atom Transfer

**DOI:** 10.1021/jacsau.5c00530

**Published:** 2025-06-16

**Authors:** Elena Cassera, Vittoria Martini, Valerio Morlacci, Serena Abrami, Nicola Della Ca’, Davide Ravelli, Maurizio Fagnoni, Luca Capaldo

**Affiliations:** † PhotoGreen Lab, Department of Chemistry, 19001 University of Pavia, viale Taramelli 12, Pavia 27100, Italy; ‡ SynCat Lab, Department of Chemistry, Life Sciences and Environmental Sustainability, 9370 University of Parma, Parco Area delle Scienze 17/A, Parma 43124, Italy

**Keywords:** photocatalyzed HAT, aldehyde, acyl radical, decarbonylation, radical chemistry

## Abstract

Photocatalyzed hydrogen atom transfer (HAT) is now an established methodology in the synthesis of pharmaceuticals and agrochemicals as well as in the development of late-stage functionalization campaigns. Yet, the realization of the full potential of this manifold is held back by intrinsic challenges that still demand meticulous exploration and resolution, such as the lack of regioselectivity and inefficiency. Herein, we address these limitations by proposing a decarbonylative strategy. The fast direct HAT from aldehydes formyl group and ensuing α-fragmentation of the photogenerated acyl radical (i.e., decarbonylation) is exploited to boost efficiency, redirect regioselectivity, and enable reactivity in methodologies based on HAT. We validated this concept for decarbonylative C–C bond formation. In-depth mechanistic investigation based on laser-flash photolysis and density functional theory highlights the crucial role of kinetic factors in controlling the observed chemistry. Our work demonstrates that the exceptional hydrogen atom-donating ability of aldehydes can be harnessed to redefine paradigms in HAT photocatalysis.

## Graphical abstract

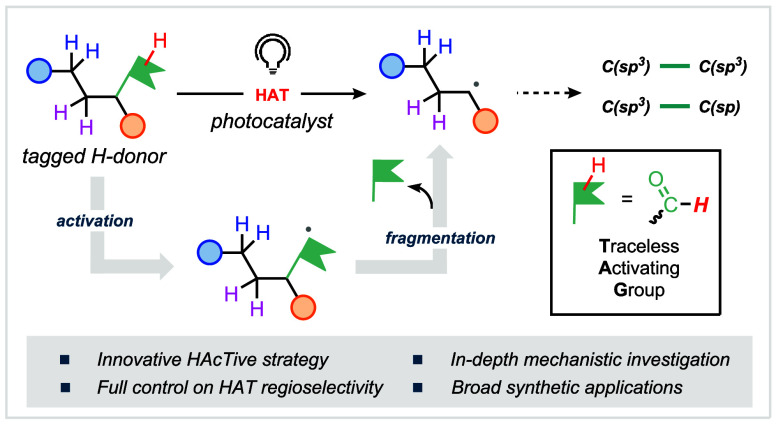

## Introduction

In the new century, there has been a surge in synthetic methods based on the generation of radicals, especially carbon-centered ones. Photocatalyzed hydrogen atom transfer (HAT) has attracted substantial attention from synthesis practitioners to access these intermediates, often being hailed as the holy grail of synthetic methodology (Figure 1A).^1–5^ In fact, a suitable photocatalyst absorbs a photon and cleaves homolytically an aliphatic C–H bond from an H atom donor to produce the desired carbon-centered radicals.

**Figure 1:**
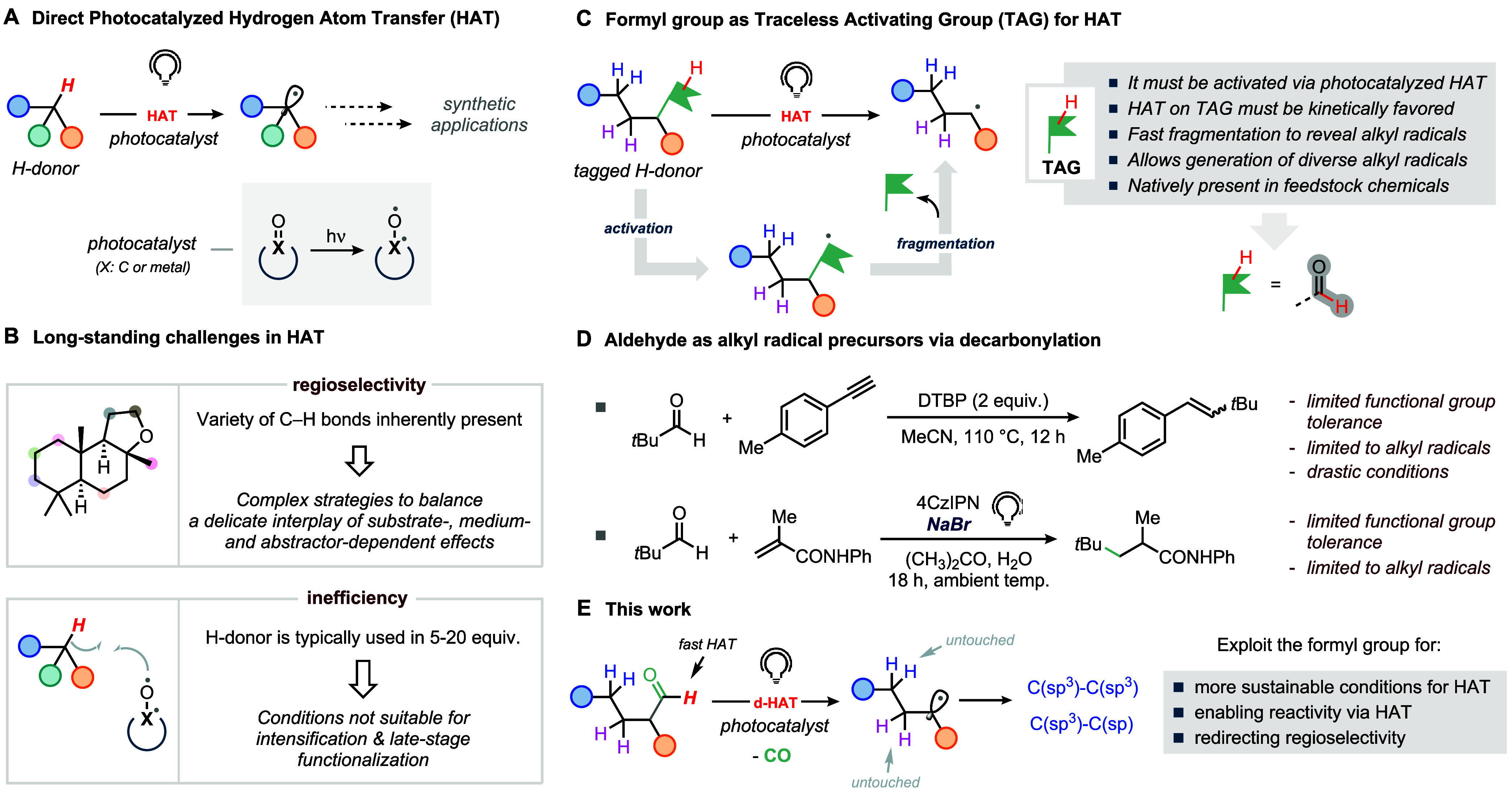
(A) Photocatalyzed hydrogen atom transfer (HAT) for the generation of carbon-centered radicals. (B) Regioselectivity and efficiency are long-standing challenges, still frustrating the wide adoption of HAT for process chemistry and late-stage functionalization campaigns. (C) Formyl group as the traceless activating group (TAG) for photocatalyzed HAT. (D) Aldehydes as alkyl radical precursors. (E) This work.

Although riveting, realization of the full potential of this manifold is accompanied by challenges that still demand meticulous exploration and resolution (Figure 1B). Foremost among these challenges is the issue of regioselectivity. In fact, C–H bonds are omnipresent in organic molecules, which calls for the development of tactics to achieve regioselectivity in the C–H cleavage step. This selectivity can be somewhat adjusted by mastering the complex interplay between substrate, photocatalyst, and medium characteristics,^6–12^ but full control still remains out of reach. Another major limitation for the wide adoption of photocatalyzed HAT is represented by inefficiency. To cope with this, H atom donors are typically used in superstoichiometric amounts (up to 20 equiv.), which is paradoxical given the intrinsic atom-economy of HAT. While this may not be a significant obstacle when working with readily accessible substrates (e.g., tetrahydrofuran or cyclohexane), it is a considerable drawback for late-stage functionalization endeavors.^13^ Indeed, in these circumstances, employing multiple equivalents of the H atom donors may be unfeasible due to restricted availability and substantial cost of the starting materials.^2^

Determined to address the two limitations outlined above, we were inspired by photoredox catalysis,^14–18^ where a redox auxiliary group is often introduced in the substrate to match its redox potential with that of the excited state of a photocatalyst. Upon successful electron transfer, mesolytic cleavage of the auxiliary group yields the desired carbon-centered radical. Overall, the use of redox auxiliary groups ensures site selectivity in the formation of the radical, at the expense of atom and step economy. In limited cases,^19^ the auxiliary group can be traceless and leaves no residues in the reaction mixture. We became intrigued in extrapolating this concept to the field of HAT by taking advantage of a suitable traceless activating group (TAG, Figure 1C). This group should be activated via HAT and react faster than most typical aliphatic C–H bonds found in organic molecules. This ensures that the H-donor can be used in a stoichiometric amount (1 equiv. instead of >5). Moreover, the radical generated via HAT from the TAG should undergo prompt fragmentation to reliably reveal the desired carbon-centered radical with minimum impact on the atom-economy of the process.

Finally, the TAG should be a ubiquitous functional group in organic molecules, granting access to structurally diverse alkyl radicals. In view of the above, the formyl group of aldehydes is the ideal TAG for photocatalyzed HAT (Figure 1C).^20^

In fact, besides C­(sp^3^)–H bonds, photocatalyzed HAT can be exploited for the activation of aldehydic formyl C­(sp^2^)–H bonds to yield acyl radicals.^21–28^ The latter intermediates are directly exploited for the synthesis of unsymmetrical ketones.^22,29–33^ Competitive decarbonylation to unveil alkyl radicals has been in some cases documented,^21,26,28^ but the synthetic potential of this phenomenon was not recognized.

Recently, the use of aldehydes as alkyl radical precursors via decarbonylation has been proposed,^34^ but demands harsh conditions and explosive peroxides, restricting its applicability to fully aliphatic aldehydes with limited functional group tolerance, as exemplified by Li’s work on the decarbonylative hydroalkylation of alkynes (Figure 1D, upper part).^35^ Based on this precedent, Deng proposed aldehydes as a precursor for alkyl radicals via bromine-mediated indirect HAT^5^ for the radical hydroalkylation of acrylamides (Figure 1D, lower part).^36^ Similarly, this protocol was limited by the scarce functional group tolerance and the fact that only alkyl radicals could be generated.

In view of the above, we hypothesized that, besides providing smooth access to alkyl radicals, decarbonylative HAT could serve as a strategy to overcome the outlined limitations of HAT photocatalysis (i.e., regioselectivity and inefficiency).^34^

Herein, we demonstrate the feasibility and implementation of several protocols for C–C bond formation via decarbonylative direct HAT (d-HAT, Figure 1E). We organized our work specifically to prove the beneficial effect of this approach over traditional HAT. First, we demonstrate that it enables more practical conditions for photocatalyzed d-HAT, by significantly lowering the excess of H-donor needed to achieve efficient transformations. Second, we show that the introduction of TAG improves the selectivity of the overall process when the corresponding TAG-free substrate undergoes alternative pathways. Third, we demonstrate that it can be used to redirect the regioselectivity in the hydrogen abstraction step, thus unlocking new opportunities in photocatalyzed synthesis. Remarkably, we exploited this strategy to access electrophilic radicals via HAT, which is a limitation in the state of the art of this methodology.^37,38^ Next, we harnessed our approach to develop a protocol for the formal synthesis of a wide range of amino alcohols by exploiting Garner’s aldehyde as the model substrate. Finally, the working principles of a TAG have been uncovered through a comprehensive mechanistic study including experimental, spectroscopic, and computational studies.

Our work demonstrates that the exceptional hydrogen atom-donating ability of aldehydes can be harnessed to redefine paradigms in HAT photocatalysis.

## Results and Discussion

We started validating our concept by comparing the reactivity of methyl *tert*-butyl ether (**S1**) and alkoxyaldehyde **1a** in a Giese-type radical hydroalkylation reaction. The decatungstate anion (4 mol% as tetrabutylammonium salt, TBADT) was chosen as the photocatalyst to trigger the HAT step.^39–44^ When the reactions were performed in the presence of 2-vinylpyridine (**2a**) as the SOMOphile under irradiation at 390 nm for 16 h, product **3** was detected (Figure 2A, part i). As anticipated, **3** was formed only in trace amounts when 1.2 or 2 equiv. of **S1** were used. Interestingly, even with 5 equiv. of the H-donor, the yield did not exceed 30%, in spite of the total consumption of 2-vinylpyridine.

**Figure 2:**
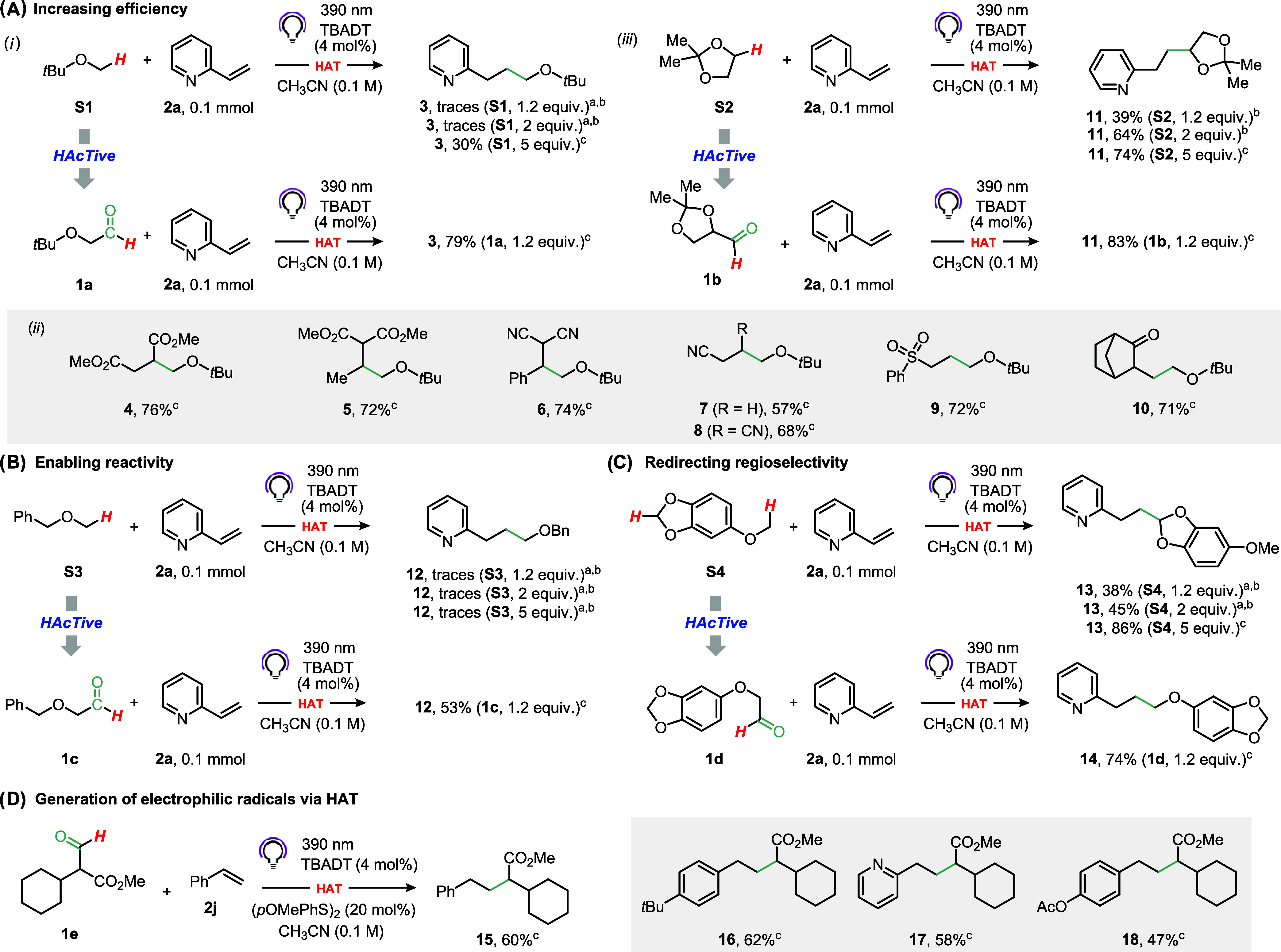
Validation of decarbonylative d-HAT to (A) increase reaction efficiency, (B) enable reactivity for recalcitrant substrates, (C) redirect regioselectivity, and (D) generate electrophilic carbon-centered radicals. See GP1 and GP2 in the Supporting Information for experimental details. ^a^ Incomplete conversion of 2-vinylpyridine was observed. ^b^ GC yields. ^c^ Yields of the isolated products.

When **S1** was replaced with the corresponding tagged H-donor **1a**, the advantage of the decarbonylative strategy was immediately evident. Indeed, compound **3** was formed in good yield (79%) without the need for a large excess of the H-donor (1.2 equiv.). Interestingly, no competitive formation of the corresponding undesired acylated product was detected by GC-MS analysis, and **2a** was completely consumed at the end of the reaction. This approach was verified across different classes of electrophilic olefins, including unsaturated esters (compounds **4** and **5**), nitriles (**6**–**8**), sulfones (**9**), and ketones (**10**), reliably delivering the expected Giese adducts in good yields (Figure 2A, part ii). These results clearly show that our strategy enables more practical conditions for the photocatalyzed HAT. A similar outcome in terms of improved efficiency granted by the decarbonylative strategy was observed in the preparation of **11** when dioxolane **S2** was replaced by aldehyde **1b** (Figure 2A, part iii).

On a different note, benzyl methyl ether (**S3**) is a poor substrate for the Giese reaction, most likely because of the competition between the two α–to–O positions for the HAT step (Figure 2B). In fact, a complex mixture resulted from the irradiation, and the desired product **12** was detected only in traces, regardless of the amount of **S3** used (up to 5 equiv.). The outcome was completely different, however, when **1c** was used in the role of H-donor: compound **12** was isolated in a satisfying yield as the sole product (Figure 2B). Hence, in this case, our strategy successfully revived a reaction that would otherwise be hindered by competitive reaction pathways.

Next, we set out to probe the validity of our approach to steer regioselectivity in photocatalyzed HAT (Figure 2C). Thus, when sesamol methyl ether (**S4**) was used as the H-donor in our radical hydroalkylation conditions, product **13** was obtained in decent yields (38% with 1.2 equiv. of **S4**) with complete selectivity for the functionalization of the acetalic methylene site. The product derived from the hydrogen abstraction on the methyl group was not observed. Higher concentrations of the H-donor improved the yield but left the regioselectivity unaltered. Impressively, when the tagged H-donor **1d** was adopted in only 1.2 equiv., the regioselectivity was completely diverted and compound **14** was formed instead. In this case, product **13** was not observed (Figure 2C).

Finally, we tackled another grand challenge in HAT chemistry: the selective generation of electrophilic radicals.^37^ Given that most H-abstractors are inherently electrophilic, they preferentially target hydridic (electron-rich) C–H bonds, leaving protic (electron-poor) sites largely inaccessible. We surmised we could leverage the TAG to guide the photocatalyst via a polarity-matched HAT. Upon decarbonylation of **1e**, the stabilized electrophilic radical would be unveiled, thus offering a practical solution to this long-standing limitation. Pleasingly, our strategy proved effective, enabling the hydroalkylation of styrenes using a disulfide cocatalyst (Figure 2D, compounds **15–18**).

Motivated by the significant impact of the TAG for the generation of oxyalkyl and electrophilic radicals (Figure 2), we wondered whether the strategy could be exploited for the generation of other α-to-heteroatom radicals, e.g., α-amidoalkyl ones. Results are shown in Figure 3A. Thus, a maximum yield of 54% (after isolation) was obtained for compound **19** when using Boc-protected piperidine **S6** in large excess (5 equiv.), but the use of just 1.2 equiv. of tagged substrate (**1f**) allowed to isolate **19** in 72% yield. Furthermore, when **2a** was reacted with *N*-Boc-(methylamino)­acetaldehyde **1g** as the hydrogen donor, the corresponding *tert*-butyl methyl­(3-(pyridin-2-yl)­propyl)­carbamate **20** was isolated in 82% yield (see Section S7.2, Supporting Information).

**Figure 3:**
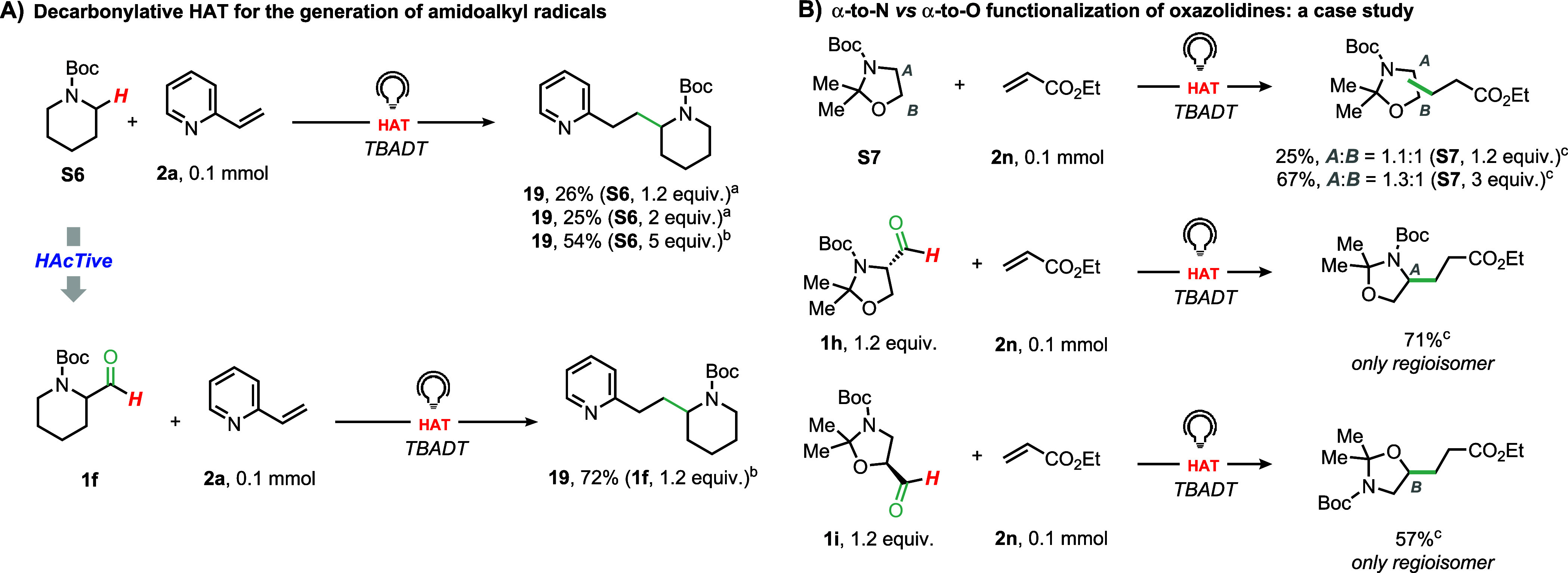
Validation of decarbonylative d-HAT for the generation of amidoalkyl radicals for a Giese-type reaction to (A) increase reaction efficiency and (B) steer regioselectivity. ^a^ GC yields, ^b^ yield after isolation. ^c 1^H NMR yields are shown (internal standard: CH_2_Br_2_).

At this stage, we started investigating whether the observed advantages of decarbonylative HAT could be integrated to design novel synthetic platforms for C–C bond formation. Inspired by recent work from Baran,^45,46^ we opted to apply decarbonylative strategy to achieve the selective functionalization of the 2,2-dimethyl-oxazolidine ring (**S7**, Figure 3B) as a convenient and atom-economical cassette for the synthesis of 1,2-amino alcohols. In fact, the oxazolidine ring contains two methylene groups—the α–to–N and α–to–O positions—that compete for functionalization via photocatalyzed HAT. As the bond dissociation energies of α–to–N and α–to–O C–H bonds are nearly identical (92 ^47^ vs 90 ^48^ kcal·mol^–1^ for tetrahydrofuran and pyrrolidine, respectively), this issue cannot be effectively addressed on thermodynamic grounds. The Garner’s aldehyde (**1h**)^49^ is a tagged surrogate for **S7**, and it is commercially available. It can also be readily prepared on a large scale (see Section S4.2 in the Supporting Information).^50^ We hypothesized that the formyl group could promote a fast HAT step, delivering an acyl radical. Ensuing decarbonylation would yield a stabilized α-amidoalkyl radical. Overall, the formyl group would serve as a TAG to outcompete H-abstraction from the α-to-O site.

These hypotheses were fully confirmed by our experimental work (Figure 3B). In fact, under the reaction conditions outlined in Figure 2, the adoption of oxazolidine **S7** (1.2 equiv.) as the hydrogen donor and ethyl acrylate (**2n**) as the radical trap led to functionalization at both the α–to–N and α–to–O positions in a 1.1:1 ratio and with a modest overall yield of 25%. If **S7** was used in excess (3 equiv.), the product was obtained in 67% yield with unaltered selectivity. In sharp contrast, using **1h** (1.2 equiv.) as the hydrogen donor significantly improved the reaction outcome, resulting in selective α–to–N functionalization in 71% yield (Figure 3B). In complete accordance with the founding principles of our approach, the use of regioisomeric aldehyde **1i** redirected the selectivity toward the α–to–O position (57%, Figure 3B, see also Section S9 in the Supporting Information). Taken together, these findings suggest that decarbonylative d-HAT represents a streamlined approach for the versatile synthesis of amino alcohols.

After a quick round of optimization of reaction conditions (Section S5.1 in Supporting Information), we reacted **1h** with a wide array of electron-poor olefins to forge C­(sp^3^)–C­(sp^3^) bonds (Figure 4). Starting from α,β-unsaturated ketones, the reaction occurred smoothly with methyl vinyl ketone and methylene norbornanone, allowing us to obtain the expected adducts **21** and **22** in 66% and 76% yield after isolation, respectively. Next, we found that acrylates were elective compounds in the role of SOMOphiles (**23**–**30**), reliably producing the corresponding hydroalkylation products in high yields. Intriguingly, complex acrylates derived from biologically relevant alcohols, such as prolinol, galactose, cholesterol, and menthol, were also successfully employed (**27**–**30**, 50–83%), underscoring the robustness and applicability of this protocol. Olefins bearing other electron-withdrawing functional groups were also well tolerated (**31**–**34**, 40–79%). Radical addition onto 2-benzylidenemalononitrile proceeded well and resulted in the formation of adduct **32** in 79% yield (dr 1.2:1). Similarly, (vinylsulfonyl)­benzene (**2g**) and **2a** allowed us to prepare the corresponding Giese adducts **33** and **34** in good yields (65% and 75%, respectively). Motivated by these findings, we subsequently developed a protocol for the SOMOphilic alkynylation of the Garner’s aldehyde (see Section S5.3 in the Supporting Information).^51,52^

**Figure 4:**
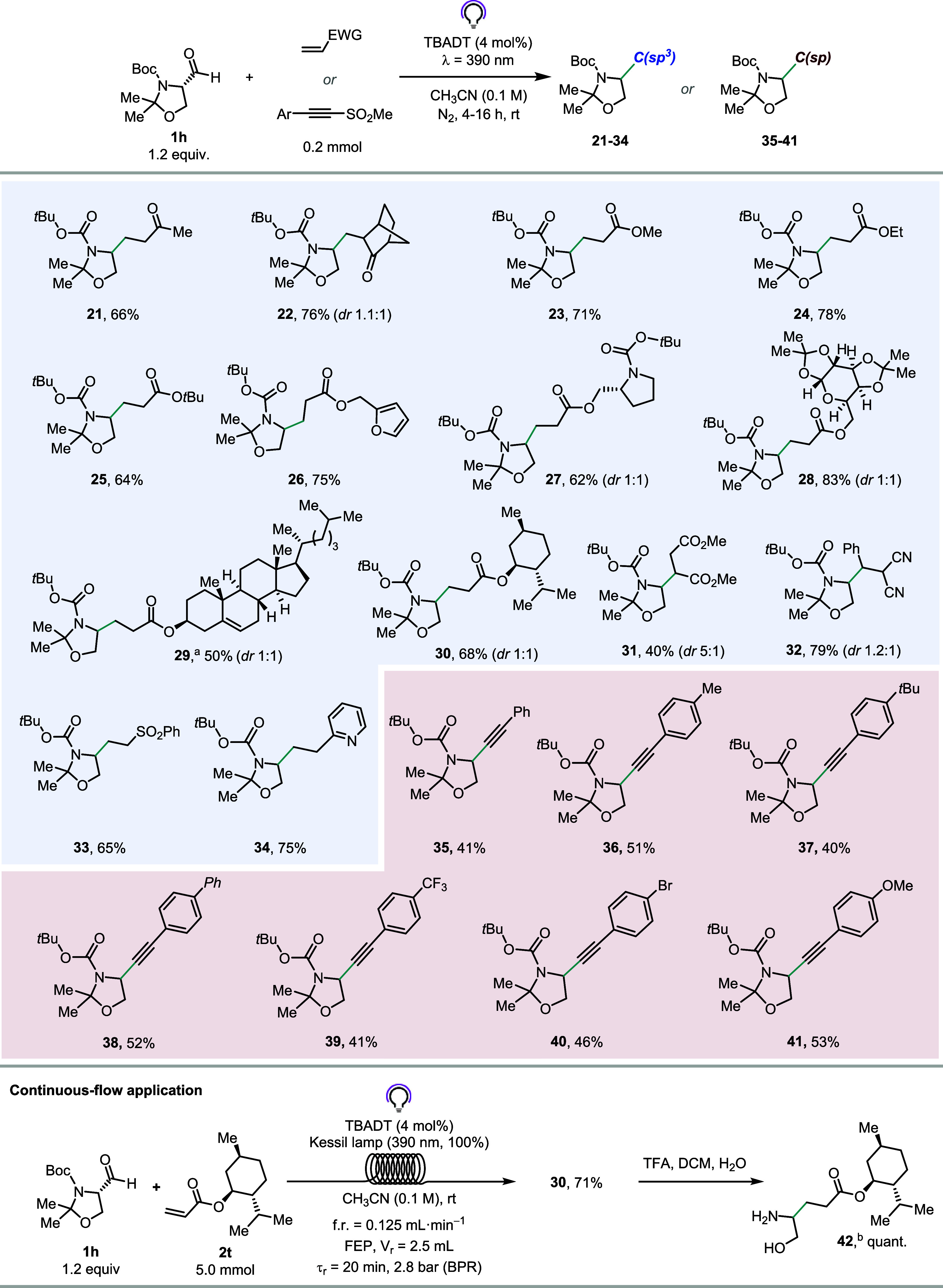
Scope of the decarbonylative alkylation (blue background) and SOMOphilic alkynylation (red background) of Garner’s aldehyde (**1h**) for the formal synthesis of amino alcohols. All yields are meant after isolation. Reaction conditions for C­(sp^3^)–C­(sp^3^) bond formation: **1h** (1.2 equiv.), olefin (0.2 mmol), and TBADT (4 mol %) in CH_3_CN (0.1 M). The reaction mixture was bubbled with N_2_ (2 min) and irradiated at 390 nm for 16 h. Reaction conditions for C­(sp^3^)–C­(sp) bond formation: **1h** (1.2 equiv.), alkynyl sulfone (0.2 mmol), and TBADT (4 mol %) in CH_3_CN (0.1 M). The reaction mixture was bubbled with N_2_ (2 min) and irradiated at 390 nm for 4 h. ^a^ Reaction was performed in CH_3_CN/CH_2_Cl_2_ (0.05 M). ^b^ Reaction was performed on 0.2 mmol of **30**.

Although the expected products were generally not obtained in outstanding yields (Figure 4), it is important to stress that our approach offers a new metal-free retrosynthetic perspective for the synthesis of alkynyl amino alcohols (and amino acids), which are more traditionally obtained via a Sonogashira-type cross-coupling.^53^ The α–to–O functionalization was never observed. Thus, several *para*-substituted ((methylsulfonyl)­ethynyl)­benzenes allowed the expected products **35**–**41** to be obtained in moderate yields after isolation (40–53%). In an attempt to reduce the reaction time and enable scalability of the protocol, we managed to translate the transformation into continuous flow for product **30** (Figure 4, lower part; see Section S6.3 in the Supporting Information). Thus, a CH_3_CN solution of **1h**, menthyl acrylate (**2t**), and TBADT was pushed by means of a syringe pump into a photochemical reactor (FEP, *V*_R_ = 2.5 mL, ID = 0.8 mm, see Figure S2).

As expected, when the reaction was performed in the absence of a back-pressure regulator (BPR), the flow regime was irregular because of CO gas evolution, and the residence time was not reproducible. The installation of a BPR (2.8 bar) at the outlet of the photochemical reactor solved this issue and allowed to obtain **30** in 71% yield on a 5 mmol scale. As expected, the oxazolidine ring could be opened under acidic conditions to unveil the corresponding α–to–N-functionalized amino alcohol **42** in quantitative yield.

Next, we shifted our focus to understanding the origin of the selectivity of the HAT step by adopting a combined experimental and computational approach. On the one hand, we used laser-flash photolysis (LFP) to appreciate the kinetic advantage offered by our strategy. In more detail, we opted to measure the quenching rates of the excited state of TBADT (see Section S9.2 in the Supporting Information) by a set of tagged H-donors used in this work and compared them with those obtained by using the corresponding untagged scaffolds.

In practice, we constructed relevant Stern–Volmer (SV) plots and calculated the corresponding absolute kinetic constants for the HAT step. The kinetic data for substrates **S1** vs **1a** and **S7** vs **1h** are presented in Figure 5A (left), while additional substrate comparisons are shown in Figure 5A (right). In all cases, SV analysis consistently showed the kinetic advantage of using tagged substrates and, in some cases, quenching rates proved to be 5–10 times higher than those of untagged ones. Of note, the reported kinetic constants have not been corrected for the number of hydrogen atoms. These experiments strongly suggest that kinetics plays a fundamental role in the reported strategy.

In parallel, we conducted DFT calculations at the ωB97xD/def2TZVP level of theory (implicit CH_3_CN solvent) to have insights into the reasons for the observed selectivity (see Section S9.3 in the Supporting Information for further details). In particular, we modeled H-abstraction from all the available positions in model compound **1a** (**H**_**a**–**c**_) and untagged derivative **S1** (**H**_**a,b**_; see Figure 5B). As the hydrogen abstractor, we adopted the *tert*-butoxyl radical (*t*BuO^•^) based on the assumption that most photocatalysts operating via direct HAT (when in the excited state) show a behavior analogous to this alkoxyl radical.^54^ Furthermore, *t*BuO^•^ is a convenient choice due to the limited computational cost and the symmetric substitution pattern of the *tert*-butyl group, which limits the number of conformations to be screened. Figure 5B (left) describes the thermodynamic (Δ*G*) and kinetic (Δ*G* ^‡^) parameters associated with the hydrogen abstraction processes by the *tert*-butoxyl radical from the different positions of **1a** and **S1**. Importantly, Δ*G* values offer indications of both the relative strength of the cleaved C–H bonds and the relative stability of the generated C-centered radicals. On the one hand, the introduction of the TAG has a negligible impact on both thermodynamic and kinetic parameters of **H**_**a**_ abstraction. On the other hand, it has a tremendous impact on the lability of C–**H**_**b**_ bond (ΔΔ*G* ≃ 18 kcal mol^–1^), since a highly stabilized captodative radical is formed upon cleavage of named bond in **1a**.

**1a** features an additional (quite) labile position, namely, formyl C–**H**_**c**_, with the overall bond energy order: C–**H**_**a**_ ≫ C–**H**_**c**_ > C–**H**_**b**_, somehow contradicting the experimental selectivity toward the C–**H**_**c**_ position. Actually, in the context of radical chemistry, it is well established that selectivity profiles often emerge from kinetic rather than thermodynamic factors.^41,55^ The activation energy for C–H cleavage (Δ*G* ^‡^) follows the order: C–**H**_**a**_ ≫ C–**H**_**b**_ > C–**H**_**c**_. This behavior can be rationalized based on polar effects governing the transition state for the C–H cleavage: it is no surprise that the electrophilic *tert*-butoxyl radical targets the hydridic formyl C–H site (C–**H**_**c**_) with exquisite selectivity. Collectively, these results point toward kinetic selectivity too: although weaker bonds are present in tagged substrates, the TAG is able to guide HAT.

Another key aspect for the success of our strategy is the decarbonylation step, which must be fast, avoiding any competitive reactivity of the initially formed acyl radical (Figure 5B, right). Thus, decarbonylation of the acyl radicals deriving from substrates **1a**, **1e**, and **1h** has to confront modest barriers (Δ*G* ^‡^; +9.44, +9.26, and +6.86 kcal·mol^–1^, respectively), especially in the latter case. At the same time, all the described decarbonylation steps are exergonic processes, characterized by a negative energy change; the most negative value is observed for the generation of the carboxymethyl-substituted radical that can benefit from direct conjugation of the radical site with the π system of the C=O moiety. For the sake of comparison, the same process has been modeled for the acyl radicals arising from propanaldehyde and isobutyraldehyde, taken as reference for primary and secondary acyl radicals, respectively. Thus, decarbonylation of the former is slightly endergonic (Δ*G* = +3.75 kcal·mol^–1^) and kinetically challenging (Δ*G* ^‡^ ≃ +16 kcal·mol^–1^). In contrast, the decarbonylation of the secondary acyl radical is almost thermoneutral and occurs with a smaller activation energy (Δ*G* ^‡^ ≃ +13 kcal·mol^–1^) than the primary one. To promote decarbonylation in these borderline cases, temperature was proved to be an effective tool.^28^ On the one hand, this analysis highlights an important aspect of the decarbonylative strategy: its efficiency is closely linked to the decarbonylation step, making it particularly well suited for generating stabilized carbon-centered radicals. On the other hand, it suggests the formyl group as an excellent hydrogen atom donor, capable of guiding selectivity despite the presence of weaker C–H bonds within the substrates and boosting efficiency in the d-HAT step thanks to kinetic factors. Importantly, an additional advantage of the proposed approach is the traceless nature of the CO group, which does not leave residues to be removed at the end of the process.

**Figure 5:**
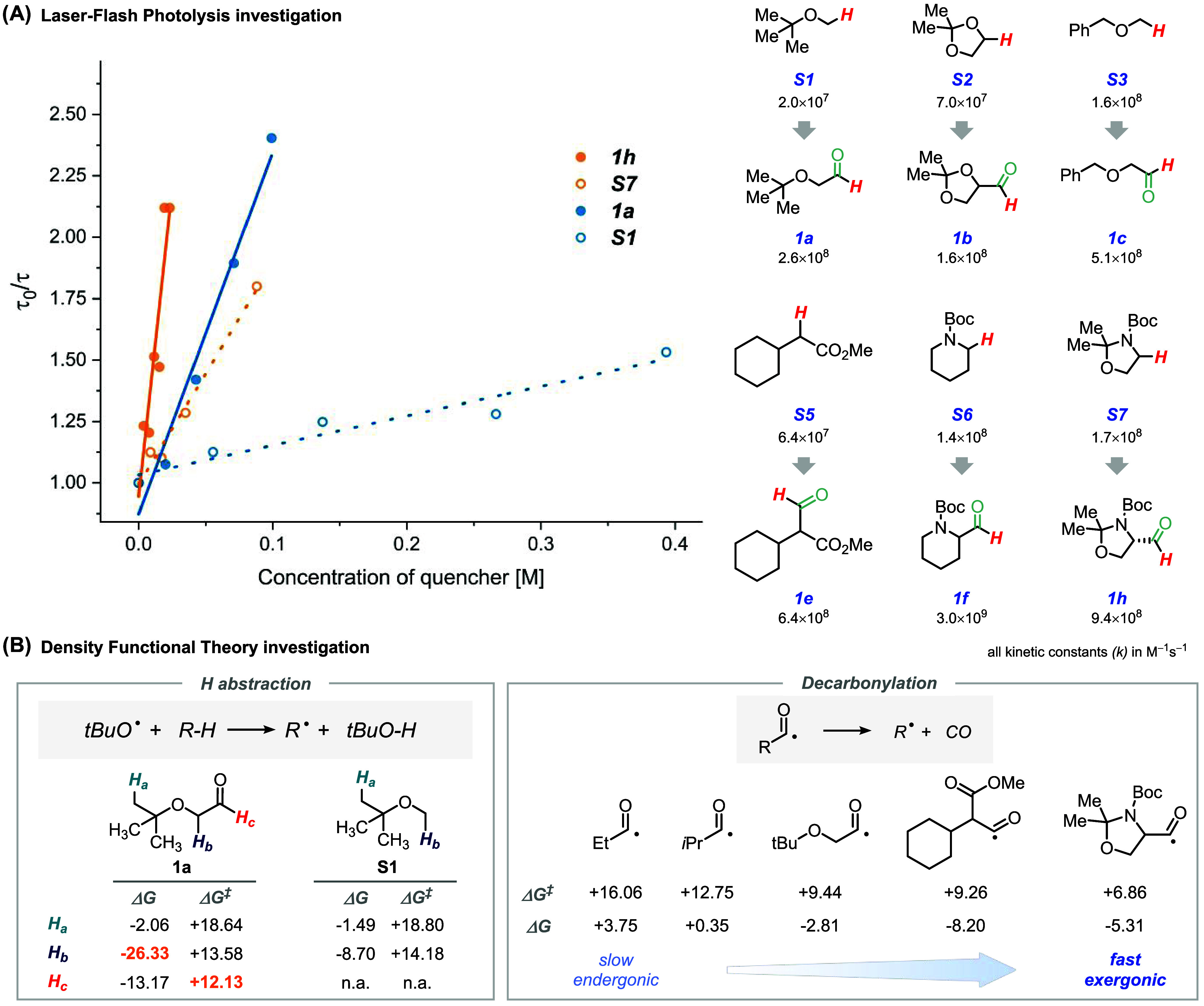
Mechanistic studies: (A) laser-flash photolysis experiments and (B) density functional theory analysis.

## Conclusions

We reported a decarbonylative strategy for photocatalyzed direct hydrogen atom transfer, which harnesses kinetic factors inherent to the HAT step to address the issues of regioselectivity and inefficiency. We advance the name HAcTive for this approach, which involves exploiting the formyl group as a traceless activating group (TAG) at the targeted position on the substrate. The rapid HAT from the highly hydridic formyl C­(sp^2^)–H bond of aldehydes is followed by the α-fragmentation of the resulting acyl radical (i.e., decarbonylation).

Given the widespread availability of aldehydes and their straightforward synthesis from other functional groups (e.g., esters and alcohols), we are confident that the findings presented in this work will enhance the application of photocatalyzed HAT in the synthesis of pharmaceuticals and agrochemicals, as well as in the advancement of late-stage functionalization strategies. In some cases, a degree of prefunctionalization is required, seemingly at odds with the atom-economy traditionally associated with HAT. However, this is amply justified by the key challenges our approach overcomes: biased regioselectivity and lower reaction efficiency. Our work demonstrates that aldehydes are exceptional hydrogen donors, and their reactivity can be harnessed to redefine paradigms in HAT photocatalysis.

Additional studies are underway to further expand the strategy.

## Supplementary Material



## Notes

Additional experimental procedures, characterization data for synthesized compounds, NMR spectra, laser-flash analysis data, DFT analysis, and photographs of the experimental setup (PDF)
