# Supplementary material for: A Decarbonylative Strategy to Enhance Efficiency and Regioselectivity in Photocatalyzed Hydrogen Atom Transfer
Source: JACS Au. 2025 Jun 16;5(7):3491–9. doi: 10.1021/jacsau.5c00530 (PMC12308375; doi:10.1021/jacsau.5c00530)
Supplement: Supplementary file 1 [file au5c00530_si_001.pdf]

Supporting Information

A Decarbonylative Strategy to Enhance Efficiency and Regioselectivity in Photocatalyzed Hydrogen-Atom Transfer

Elena Cassera,[a,‡] Vittoria Martini,[b,‡] Valerio Morlacci,[b] Serena Abrami,[b] Nicola Della Ca',[b] Davide Ravelli,[a] Maurizio Fagnoni,[a,*] Luca Capaldo[b,*]

[a] PhotoGreen Lab, Department of Chemistry, University of Pavia, viale Taramelli 12, 27100 Pavia, Italy.
[b] SynCat Lab, Department of Chemistry, Life Sciences and Environmental Sustainability, University of Parma, Parco Area delle Scienze 17/A, 43124 Parma, Italy.

E-mail: luca.capaldo@unipr.it; maurizio.fagnoni@unipv.it

‡ These authors contributed equally to this work

Contents

1. General information S3
2. Reactor design S5
3. Chart of starting materials S6
4. Synthesis of starting materials S7
   4.1. Synthesis of untagged H-donors S7
   4.2. Synthesis of tagged H-donors S8
   4.3. Synthesis of radical traps S11
5. Optimization of reaction conditions S15
   5.1. Hydroalkylation of activated olefins S15
   5.2. Hydroalkylation of unactivated olefins S16
   5.3. SOMOphilic alkynylation S17
   5.4. Adjustment of reaction conditions for continuous-flow S18
6. General procedures for preparative experiments S19
   6.1. Hydroalkylation of activated and unactivated olefins S19
   6.2. SOMOphilic alkynylation scope S19
   6.3. Scale-up for the synthesis of compound **30** in continuous-flow S20
   6.4. Opening of the oxazolidine ring S20
7. Additional control experiments S21
   7.1. Steering regioselectivity S21
   7.2. Further example S21
8. Characterization data for products **3-43** S22
9. Mechanistic investigations S32
   9.1. Control experiments S32
   9.2. Laser-Flash Photolysis S34
   9.3. DFT analysis S39
10. References S71
11. Copy of NMR spectra S73

1. General information

Reagents and consumables. All reagents and solvents were bought from Sigma Aldrich, TCI, Fluorochem, and BLDPharm and used as received unless otherwise specified. The solvents employed in this work were purchased from Carlo Erba or Sigma Aldrich and used as received. Disposable syringes were purchased from B. Braun. TLC analysis was performed using Silica on aluminum foils TLC plates (F254, Merck) with visualization under ultraviolet light (254 nm and 365 nm) or appropriate TLC staining (potassium permanganate). Flash column chromatography was performed on silica gel 60 (70–230 mesh) manually, or automatically by a CombiFlash Nextgen 100 from Teledyne or on an Isolera Spektra One purchased from Biotage, Sweden, using Sepachrom PUREZZA open-load flash cartridges purchased from Sepachrom Srl, Italy. Syringe pumps were purchased from Chemix Inc. (model Fusion 200 Touch). All capillary tubing (FEP, inner diameter 0.8 mm), microfluidic fittings and Back Pressure Regulator (BPR) were purchased from IDEX Health & Science. The photocatalyst was synthesized according to a published procedure.[1]

NMR spectroscopy. ^{1}H and ^{13}C NMR spectra were recorded at 300 K on a Bruker AVANCE 400 Hz, JEOL 600 MHz ECZ600R or on a 300 MHz Bruker spectrometer, in $CDCl_3$ or CD_3OD-d_4, using the solvent residual signals as internal reference (7.26 and 77.0 ppm for $CDCl_3$, 3.31 and 49.0 ppm for CD_3OD-d_4, respectively for ^{1}H and ^{13}C). High temperature NMR spectra were acquired in DMSO-d_6 or toluene-d_8 at 70 °C (2.50 and 39.5 ppm for DMSO-d_6, 7.00 and 128.3 ppm for toluene-d_8, respectively for ^{1}H and ^{13}C). ^{19}F spectra were registered on JEOL 600 MHz ECZ600R at 565 Hz. The terms *m*, *s*, *d*, *t*, and *q* refer to multiplet, singlet, doublet, triplet and quadruplet, respectively, while *bs* refers to a broad signal. Chemical shifts (δ) and coupling constants (*J*) are given in ppm and in Hz, respectively. NMR data were processed using the MestReNova 14 software package. Known products were characterized by comparing to the corresponding ^{1}H NMR, ^{13}C NMR with those available in the literature.

Melting point. Melting points were measured with an Electrothermal apparatus and are uncorrected.

Mass spectrometry. Mass spectrometry analyses were performed using an Agilent GCMS 5977B instrument equipped with a quadrupole analyzer and EI source, coupled with a gas chromatographic system equipped with a capillary column. HRMS data were acquired using a X500B QTOF System (SCIEX, Framingham, MA 01701 USA), equipped with the Twin Sprayer ESI probe and coupled to an ExionLC™ system (SCIEX). The SCIEX OS software 2.1.6 was used as operating platform.

For MS detection the following parameters were applied: Curtain gas 30 psi, Ion source gas 1 45 psi, Ion source gas 2 55 psi, Temperature 450 °C, Polarity positive, Ion spray voltage -4500 V, TOF mass range 50-1600 Da, declustering potential -60 V and collision energy -10 V.

Laser Flash Photolysis experiments. Nanosecond Laser Flash Photolysis profiles were measured by means of a photolysis apparatus. The minimum response time of the detection system was about 10 ns. The laser beam (a Nd/YAG operated at λ = 355 nm) was focused on a 3 mm wide circular area of the cell and the first 5 mm in depth were analyzed at a right angle geometry. The incident

pulse energies used were 5-7 mJ per pulse. The sample absorbance at 355 nm was typically 0.6-0.8 over 1 cm. The temperature was kept constant at 295±2 K. Acquisition and processing of the absorption signals were performed by LP900 7.0.2 (Build 0) Software. Non-linear fitting procedures by the least square method were used to judge the goodness of the fit. Stock solutions of quenchers were prepared so that it was only necessary to add microliter volumes to the sample cell to obtain appropriate concentrations of the quencher.

2. Reactor design

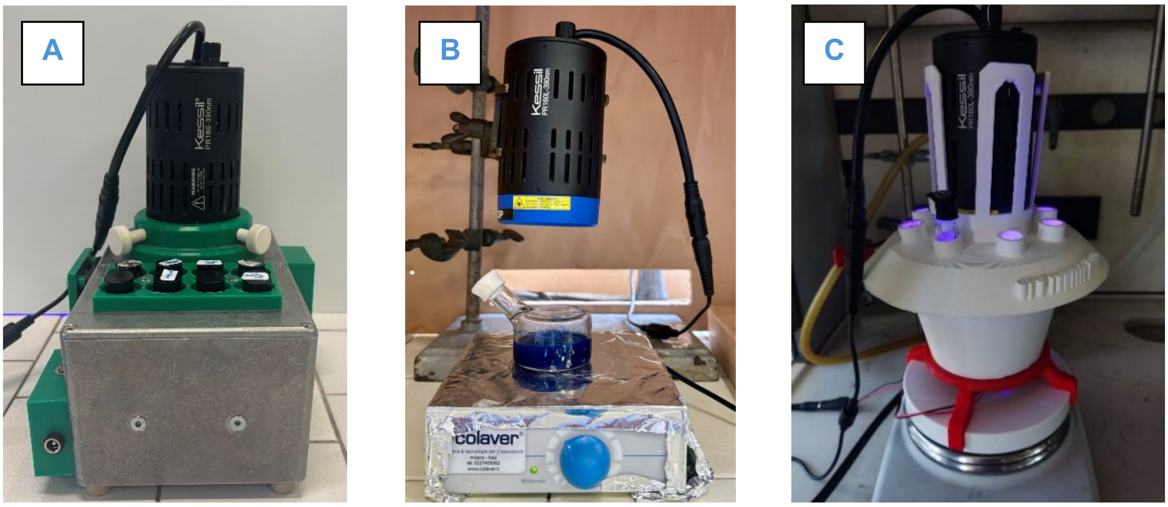

Figure S1. Photoreactors used in this work for batch irradiations.

Reactor A: Homemade photoreactor equipped with internal mirrors, a fan cooling system and a commercially available Kessil LED PR160L 390 nm.

Reactor B: Setup employed for 5 mL scale reactions, consisting in a custom-made pyrex vessel, (equipped with a stirring bar), a commercially available Kessil LED PR160L 390 nm and a fan cooling system. The Kessil LED was positioned 5 cm above the reactor.[2]

Reactor C (UFO reactor): 3D-printed reactor (PLA) equipped with a commercially available Kessil LED PR160L 390 nm (or 370 nm 2nd generation). The full characterization of this reactor is available in the literature.[3]

Reactor D (Uflow reactor): 3D-printed reactor (PLA) equipped with a commercially available Kessil LED PR160L 390 nm, tubular microreactor (V = 2.5 mL, ID: 0.8 mm, FEP), and a fixed back-pressure regulator (BPR, 40 psi) installed at the exit of the photoreactor to ensure reproducible flow rates. The full characterization of this reactor is available in the literature.[3]

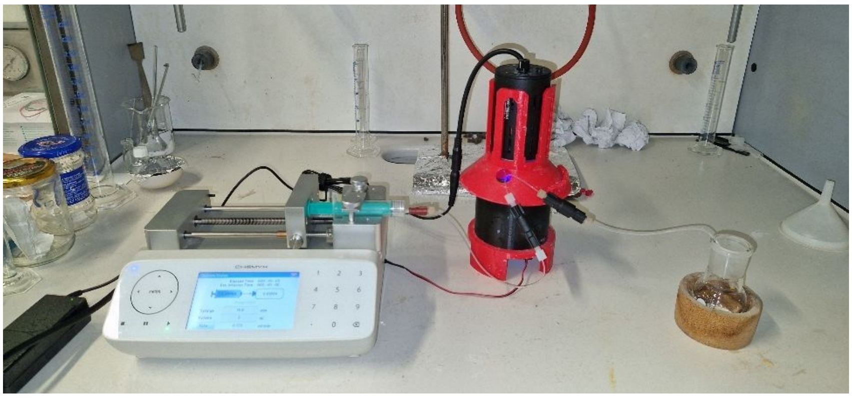

Figure S2: Photoreactor used in this work for flow irradiations

3. Chart of starting materials

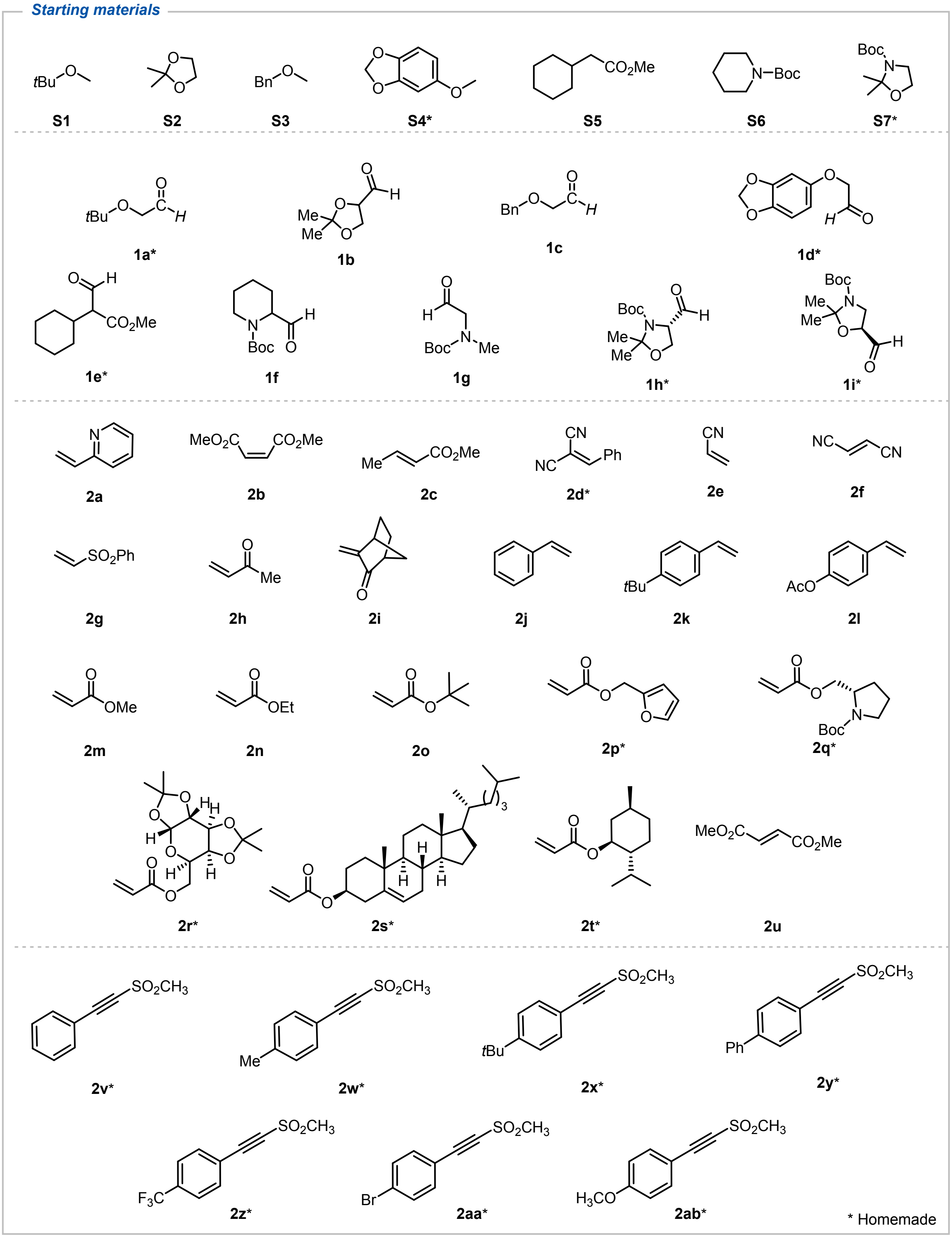

4. Synthesis of starting materials

4.1. Synthesis of untagged H-donors

*Synthesis of **S4***

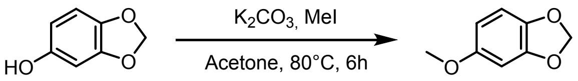

S4

Compound **S4** was synthesized following a literature procedure.[4] In particular, to a stirred solution of sesamol (4 mmol, 552 mg) in acetone (0.5 M, 20 ml) was added K_2CO_3 (5.0 equiv., 2.76 g); the mixture was stirred at 80°C for 1h, before a dropwise addition of methyl iodide (2.0 equiv., 500 μL). Upon completion (6h, monitored by TLC), the resulting solution was cooled to room temperature and filtered through celite to remove K_2CO_3. Purification on silica gel column chromatography (PET/EtOAc 85:15) allowed to quantitatively obtain (602mg) pure **S4** as a colourless oil. Spectroscopic data are in accordance with the literature.[4]

*Synthesis of **S7***

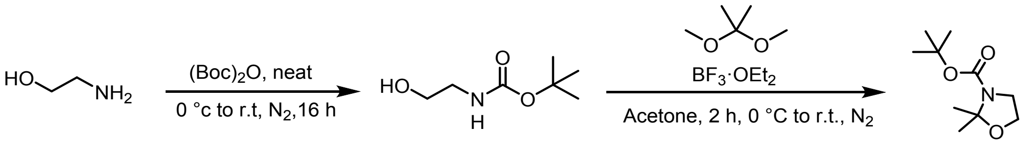

S7

In a single necked 50 mL round-bottomed flask, ethanolamine (1.2 g, 10 mmol, 1.0 equiv.) and di-*tert*-butyl dicarbonate (2.18 g, 10 mmol, 1.0 equiv.) were mixed at 0 °C. Evolution of effervescence was observed. The reaction mixture was stirred at room temperature overnight. After reaction completion, the reaction crude was dissolved in chloroform and the organic phases were extracted with water (3x50 mL), dried over anhydrous Na_2SO_4 and the solvent was removed under reduced pressure to give 0.85 g of *N*-Boc-ethanolamine as a colorless oil. Spectroscopic data are in accordance with the literature.[5] The product was used without further purification for the next step.

Compound **S7** was synthesized adapting a procedure reported in the literature.[6] In particular, to a solution of *N*-Boc-ethanolamine (846 mg, 5 mmol, 1 equiv.) in acetone (20 mL) was added 2,2-dimethoxypropane (6 mL, 44 mmol, 8.8 equiv.) and boron trifluoride etherate ($BF_3 \cdot OEt_2$, 100 μL, 0.8 mmol, 0.16 equiv.). The resulting orange solution was stirred at room temperature for 2.5 h until completion (monitored by TLC analysis). The reaction mixture was treated with 0.9 mL of triethylamine and the solvent was removed under reduced pressure. The residual brown mixture was partitioned between diethyl ether (50 mL) and a saturated aqueous sodium bicarbonate solution (50 mL). The aqueous layer was extracted with diethyl ether (2x50 mL), the combined organic phases were dried over anhydrous Na_2SO_4, and concentrated under reduced pressure to give a pale yellow oil. The latter was purified via flash chromatography on silica (Hexane/Ethyl acetate 9:1) to afford

the product **S7** as a colorless liquid (640 mg, 64% after isolation). Characterization data are in accordance with the literature.[7]

4.2. Synthesis of tagged H-donors

*Synthesis of **1a***

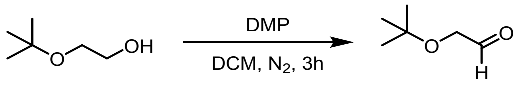

1a

Compound **1a** was prepared adapting a procedure from the literature:[8] in a flame-dried 250 mL round-bottomed flask 2-*tert*-butoxyethanol (17 mmol, 2 mL) was dissolved in 80 mL of dry CH_2Cl_2. Dess-Martin Periodinane (8 g, 1.11 equiv.) was added, and the mixture was stirred at rt for 3 h under N_2 atmosphere. The reaction was quenched by 1:1 dilution with Et_2O, followed by addition of a saturated solution of $NaHCO_3$ and $Na_2S_2O_3$ 1:7. The layers were separated, and the aqueous one was extracted three times with 50 mL of Et_2O. The combined organic layers were washed with brine and dried over Na_2SO_4 before concentration under reduced pressure. The crude product was purified by silica gel column chromatography (Pentane/ Et_2O 4:1), to give 885 mg of aldehyde **1a** (45% yield) as a colourless oil. Characterization data are in accordance with literature.[8]

^{1}H NMR (300 MHz, CDCl3) δ 9.67 (s, 1H), 3.95 (s, 2H), 1.20 (s, 9H). **^{13}C NMR (75 MHz, $CDCl_3$)** δ 202.2, 74.5, 68.4, 27.3.

*Synthesis of **1d***

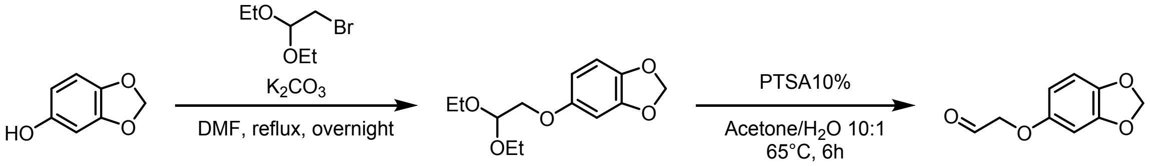

1d

Compound **1d** was prepared following a procedure from the literature.[9] In particular, to a solution of sesamol (14.5 mmol) in dry DMF (15 mL), K_2CO_3 (2.2 g, 1.1 equiv.) and bromoacetaldehyde diethyl acetal (2.4 mL, 1.1 equiv.) were added under nitrogen. The reaction mixture was heated to reflux and stirred overnight, before being diluted with 40 mL 1:1 EtOAc:H_2O. After phase separation, the aqueous layer was extracted with EtOAc and the combined organic layers were washed with H_2O and brine, dried with Na_2SO_4, and concentrated under reduced pressure to give sesamoxy acetaldehyde diethyl acetal (3.7 g, quantitative conversion) which was used for the next step without purification.

To a solution of the crude sesamoxy acetaldehyde diethyl acetal (3.7 g, 14.5 mmol), in acetone/H_2O (10:1, 15 mL), p-toluenesulfonic acid (241 mg, 10 mol-%) was added. The reaction mixture was heated to 65°C for 6h; after that, the mixture was cooled to room temperature, diluted with 40 mL 1:1 Et_2O and H_2O. After phase separation, the aqueous layer was extracted with Et_2O and the combined organic layers were washed brine and dried with Na_2SO_4. The filtrate was concentrated

under reduced pressure to give the crude aldehyde **1d** (2.4 g, 93%) as a pale orange oil, which was used directly without purification. Characterization data are in accordance with the literature.[9]

*Synthesis of aldehyde **1e***

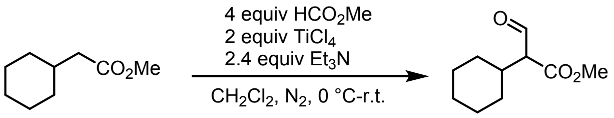

1e

Compound **1e** was synthesized adapting a procedure reported in the literature.[10] Under an argon atmosphere, methyl 2-cyclohexylacetate (0.94 g, 6.0 mmol), methyl formate (1.44 g, 24.0 mmol) and dry CH_2Cl_2 (12 mL) were introduced into a Schlenk flask. The solution was cooled to 0 °C and $TiCl_4$ (2.27 g, 12.0 mmol) followed by triethylamine (1.45 g, 14.4 mmol) were added dropwise through the syringe. The reaction mixture was stirred at 0 °C for 0.5 h and at room temperature for an extra hour. The reaction was quenched with cold water and the aqueous layer was then extracted with CH_2Cl_2 (3×50 mL). The combined organic layer was washed with water, brine and dried over Na_2SO_4. The mixture was subsequently purified with flash chromatography (Hexane:AcOEt 95:5) to obtain the desired formyl ester **1e** as a colorless oil (1.05 g, 95%). Spectroscopic data are in accordance with the literature.

^{1}H NMR (400 MHz, CDCl$_3$) δ 11.61 (d, *J* = 12.4 Hz, 1H, *enol*), 9.71 (d, *J* = 4.1 Hz, 1H, *aldehyde*), 7.03 (dd, *J* = 12.4, 0.9 Hz, 1H, *enol*), 3.80 (s, 3H, *aldehyde*), 3.77 (s, 3H, *enol*), 3.05 (m, 1H, *aldehyde*), 2.30 – 2.08 (m, 2H, *enol* + *aldehyde*), 1.84 – 1.64 (m, 10H, *enol* + *aldehyde*), 1.38 – 1.07 (m, 10H, *enol* + *aldehyde*).

^{13}C NMR (151 MHz, CDCl$_3$) δ (*enol* + *aldehyde)* 198.4, 173.1, 169.7, 160.3, 110.4, 64.7, 52.2, 51.5, 37.9, 36.3, 33.2, 30.7, 30.7, 27.0, 26.3, 26.1, 26.0, 26.0.

*Synthesis of aldehyde **1h***

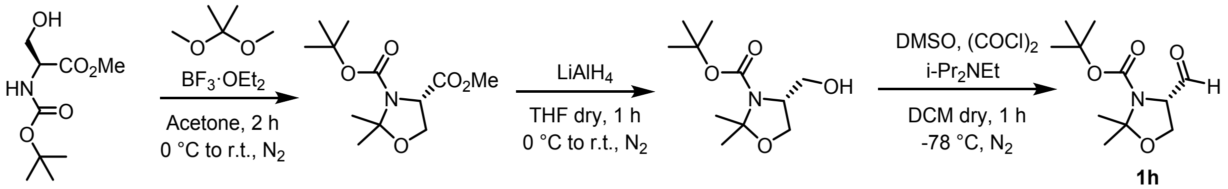

1h

Compound **1h** was prepared adapting a procedure from the literature (10 mmol scale).[6] In particular, to a solution of *N*-Boc-L-serine methyl ester (2.2 g, 10 mmol, 1 equiv.) in acetone (37 mL) was added 2,2- dimethoxypropane (11 mL, 88 mmol, 8.8 equiv.) and boron trifluoride etherate ($BF_3{\cdot}OEt_2$, 200 µL, 1.6 mmol, 0.16 equiv.). The resulting orange solution was stirred at room temperature for 2.5 hours until reaction completion (as judged via TLC). The reaction mixture was treated with 1.8 mL of triethylamine and the solvent removed under reduced pressure. The residual brown syrup was partitioned between Et_2O (100 mL) and saturated aqueous $NaHCO_3$ solution (100 mL). The aqueous layer is extracted with Et_2O (2x100 mL) and the combined organic phases are dried with anhydrous

Na_2SO_4 and concentrated under reduced pressure to give 2.8 g of oxazolidine methyl ester as a pale yellow oil. The product was used for the next step without further purification.

Thus, a 100-mL, two-necked, round-bottomed flask was equipped with a magnetic stirring bar and it was charged with $LiAlH_4$ (679.3 mg, 17.9 mmol, 1.79 equiv.). Dry THF (25 mL) was added and a suspension was obtained. To this stirring suspension, a solution of the oxazolidine ester (2.8 g, 10 mmol) in dry THF (15 mL) was added dropwise over 20 min. The dropping funnel was rinsed with 2x3 mL of dry THF, and the suspension was kept under stirring for an additional 20 minutes, until the TLC analysis showed the complete formation of the alcohol. The reaction mixture was cooled with an ice-water bath at 0°C and the reaction was quenched with 5 mL of a 10% aqueous KOH solution (NB: use caution, the reaction is exothermic!). The mixture was stirred for 1 hour at room temperature, then the white precipitate was removed by filtration through a celite pad, which was rinsed with 3x15 mL of Et_2O. The combined organic filtrates were washed with 50 mL of saturated aqueous sodium bicarbonate solution and the aqueous layer was back-extracted with Et_2O (additional 30 mL). The combined organic phases were dried with anhydrous Na_2SO_4 and concentrated under reduced pressure to give around 2 g of a pale yellow oil. The crude was then purified via flash chromatography on silica (Hexane/Ethyl acetate 7:3) to give 1.97 g of the alcohol (85% after isolation) as a yellow oil. Characterization data are in accordance with the literature.[6]

The so-obtained alcohol was oxidized via Swern oxidation. Under inert atmosphere, a dry 100 mL, two-necked, round-bottomed flask was equipped with a magnetic stirring bar and a dropping funnel. Oxalyl chloride (1.05 mL, 12.4 mmol, 1.5 equiv.) was inserted in the flask and dissolved in 20 mL of dry CH_2Cl_2, then cooled to −78 °C. While stirring, dry DMSO (1.75 mL, 24.6 mmol, 3 equiv.) was added dropwise over 20 minutes. The solution was stirred for another 20 minutes at −60 °C (NB: temperature control is crucial!). Subsequently, the dropping funnel was charged with a solution of the alcohol (1.97 g, 8.5 mmol, 1 equiv.) in 15 mL of dry CH_2Cl_2, which was added dropwise over 40 minutes maintaining the temperature at -60 °C. The funnel was rinsed with two 3-mL portions of CH_2Cl_2. At −45 °C, the dropping funnel was charged with a solution of DIPEA (8.3 mL, 47.7 mmol, 5.8 equiv.) which was added dropwise over 5 minutes. Afterwards, the solution was warmed to 0 °C within 10 minutes. The reaction solution was transferred to a 250-mL separatory funnel charged with 40 mL of an ice-cold 1 M hydrochloric acid solution. The two phases were separated, the aqueous phase was extracted with CH_2Cl_2 (3x30 mL), and the combined organic phases were washed with a saturated aqueous $NaHCO_3$ solution (3x50 mL), dried over anhydrous Na_2SO_4 and concentrated under reduced pressure to give around 1.7 g of crude. The crude is purified via flash chromatography on silica (Hexane/Ethyl acetate 8:3) to give 1.52 g of Garner's aldehyde **1h** (78% after isolation, 66% after isolation over three steps) as a yellow oil. Characterization data are in accordance with the literature.[6]

*Synthesis of aldehyde **1i***

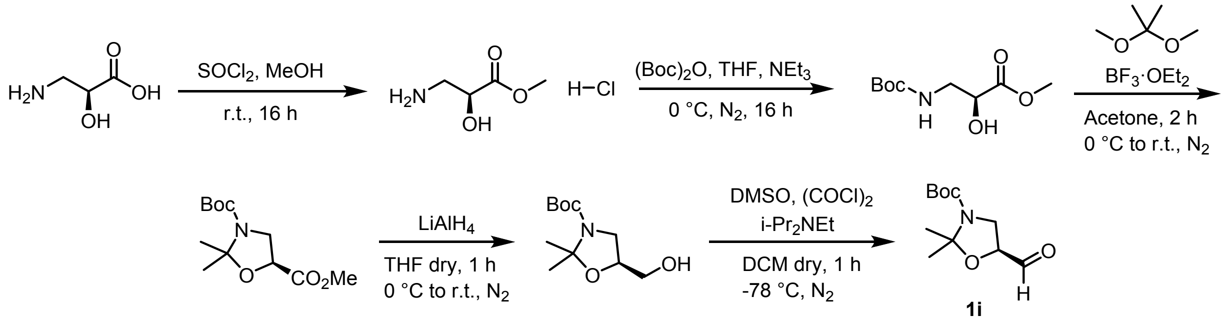

To a solution of *L*-isoserine (1 g, 9.5 mmol, 1.0 equiv.) in methanol (15 mL) at 0 ºC was added thionyl chloride (2.08 mL, 28.5 mmol, 3.0 equiv.). The reaction was maintained at 0 °C until the solid was dissolved, then stirred at room temperature overnight. Then, solvent was removed to obtain methyl (2S)-isoserinate hydrochloride as a yellow solid (□1.4 g). The product was used without further purification.

For the following steps leading to compound **1i**, the same steps described for the synthesis of **1h** were followed (9.5 mmol scale).

Crude **1i** was purified via flash chromatography on silica (Hexane/Ethyl acetate 6:4) to give 631 mg of a yellow oil (35% after isolation over all steps). Characterization data are in accordance with the literature.[11]

4.3. Synthesis of radical traps

*Synthesis of **2d***

Compound **2d** was synthesized according to a procedure reported in the literature on a 10 mmol scale.[2] The crude is purified via flash chromatography on silica (Hexane/Ethyl acetate 8:2) to afford the product as a white solid (1.1 g, 71% after isolation). Characterization data are in accordance with the literature.[2]

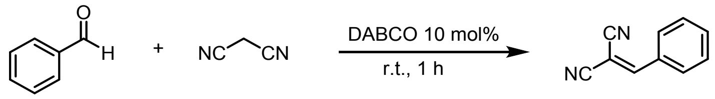

2d
71%

^{1}H NMR (400 MHz, $CDCl_3$) δ 7.91 (d, 2H), 7.78 (s, 1H), 7.64 (t, 1H), 7.55 (t, 2H). **^{13}C NMR** (101 MHz, $CDCl_3$) δ 160.1, 134.8, 131.1, 130.9, 129.8, 113.8, 112.7, 83.1.

*Synthesis of acrylates **2p-2t***

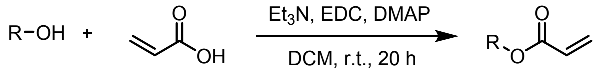

Compounds **2p-2s** were prepared adapting conditions for the Steglich esterification (3 mmol scale).[12] To a solution of the chosen alcohol (3 mmol, 1 equiv.) in CH_2Cl_2 (30 mL) at 0 °C was added triethylamine (24 mmol, 8 equiv.) followed by acrylic acid (24 mmol, 8 equiv.), 1-(3-dimethylamimopropyl)-3-ethylcarbodiimide hydrochloride (EDC, 24 mmol, 8 equiv.) and *N*,*N*-dimethylaminopyridine (0.15 mmol, 0.05 equiv.). The reaction was allowed to warm up to room

temperature over 20 hours. Upon reaction completion (as judged by TLC), the reaction was diluted with EtOAc, the organic phases were washed with water then with a solution of HCl 1 N, brine, and dried over anhydrous Na_2SO_4. Concentration in vacuo and flash chromatography (Hexane/Ethyl acetate 98:2) afforded the desired products.

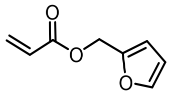

Furan-2-ylmethyl acrylate (2p).
The crude was purified via flash chromatography on silica (Hexane/MTBE 9:1) to afford the product as colourless oil (311 mg, 67% after isolation).
^{1}H NMR (400 MHz, $CDCl_3$) δ 7.47 – 7.39 (m, 1H), 6.49 – 6.40 (m, 2H), 6.37 (dd, *J* = 3, 2 Hz, 1H), 6.14 (dd, *J* = 17, 10 Hz, 1H), 5.84 (dd, *J* = 10, 1 Hz, 1H), 5.15 (s, 2H).**^{13}C NMR** (101 MHz, $CDCl_3$) δ 165.9, 149.5, 143.5, 131.5, 128.2, 110.9, 110.7, 58.2.
Spectroscopic data are in accordance with the literature.[13]

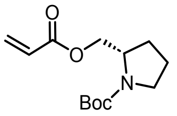

(S)-tert-butyl 2-((acryloyloxy)methyl)pyrrolidine-1-carboxylate (2q).
The alcohol was previously protected following a procedure from the literature.[14] Prepared starting from *N*-Boc-L-prolinol (603.81 mg, 3 mmol, 1 equiv.). The crude was purified via flash chromatography on silica (Hexane/Ethyl Acetate 9:1) to afford the product as a colorless liquid (421 mg, 55% after isolation).
Mixtures of rotamers**: ^{1}H NMR** (400 MHz, $CDCl_3$) δ 6.38 – 6.26 (m, 1H), 6.09 – 5.97 (m, 1H), 5.81 – 5.69 (m, 1H), 4.23 – 4.12 (m, 1H), 4.12-3.86 (m, 2H), 3.39 – 3.18 (m, 2H), 1.99 – 1.66 (m, 4H), 1.39 (s, 9H). **^{13}C NMR** (101 MHz, $CDCl_3$) δ 165.9, 154.4, 130.9, 130.7, 128.3, 79.6, 79.3, 64.8, 64.6, 55.5, 46.6, 46.4, 28.8, 28.4, 27.9, 23.7, 23.0. **HRMS** (ESI) m/z calcd for $C_{13}H_{21}NO_4Na^+$: $[M+Na]^+$ 278.1363; found: 238.1361.

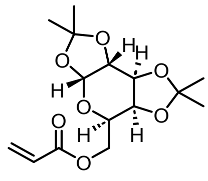

((3aR,5R,5aS,8aS,8bR)-2,2,7,7-tetramethyltetrahydro-3aH-bis([1,3]dioxolo)[4,5-b:4',5'-d]pyran-5-yl)methyl acrylate (2r).
The secondary hydroxyl groups were protected via acetals adapting a procedure from literature on 10 mmol.[15] Prepared using diacetone-D-galactose (1 g, 3 mmol, 1 equiv.). The crude was purified via flash chromatography on silica (Hexane/Ethyl Acetate 8:2) to afford the product as colourless oil (427mg, 45% after isolation).
^{1}H NMR (400 MHz, $CDCl_3$) δ 6.43 (dd, *J* = 17, 1 Hz, 1H), 6.16 (dd, *J* = 17, 10 Hz, 1H), 5.83 (dd, *J* = 10, 2 Hz, 1H), 5.54 (d, *J* = 5 Hz, 1H), 4.63 (dd, *J* = 8, 3 Hz, 1H), 4.38 (dd, *J* = 12, 5 Hz, 1H), 4.33 (dd, *J* = 5, 3 Hz, 1H), 4.30 – 4.24 (m, 2H), 4.07 (ddd, *J* = 7, 5, 2 Hz, 1H), 1.51 (s, 3H), 1.46 (s, 3H), 1.34 (s, 3H), 1.33 (s, 3H). **^{13}C NMR** (101 MHz, $CDCl_3$) δ 166.2, 131.2, 128.3, 109.8, 108.9, 96.4, 71.2, 70.8, 70.6, 66.2, 63.7, 26.1, 26.1, 25.1, 24.6. **HRMS** (ESI) m/z calcd for $C_{15}H_{22}O_7Na^+$: $[M+Na]^+$ 337.1258; found: 337.1254.

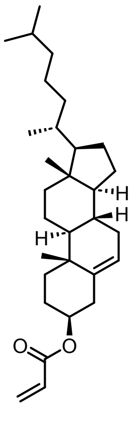

(3S,8S,9S,10R,13R,14S,17R)-10,13-dimethyl-17-((R)-6-methylheptan-2-yl)-2,3,4,7,8,9,10,11,12,13,14,15,16,17-tetradecahydro-1H-cyclopenta[a]phenanthren-3-yl acrylate (2s).
The crude was purified via flash chromatography on silica (Hexane/Ethyl Acetate 95:5) to afford the product as white solid (1.03 g, 74% after isolation).
^{1}H NMR (400 MHz, $CDCl_3$) δ 6.38 (dd, *J* = 17, 2 Hz, 1H), 6.10 (dd, *J* = 17, 10 Hz, 1H), 5.79 (dd, *J* = 10, 2 Hz, 1H), 5.39 (d, *J* = 4 Hz, 1H), 4.74 – 4.63 (m, 1H), 2.36 (d, *J* = 8 Hz, 2H), 2.05 – 1.93 (m, 2H), 1.93 – 1.77 (m, 3H), 1.70 – 1.53 (m, 4H), 1.52 – 1.42 (m, 3H), 1.39 – 1.29 (m, 3H), 1.25 (s, 3H), 1.22 – 1.06 (m, 6H), 1.03 (s, 3H), 1.01 – 0.94 (m, 2H), 0.91 (d, *J* = 7 Hz, 3H), 0.86 (dd, *J* = 7, 2 Hz, 6H), 0.68 (s, 3H). **^{13}C NMR** (101 MHz, $CDCl_3$) δ 165.8, 139.8, 130.4, 129.2, 122.9, 74.3, 56.8, 56.3, 50.2, 42.5, 39.9, 39.7,

38.3, 37.1, 36.8, 36.3, 36.0, 32.1, 32.0, 28.4, 28.2, 27.9, 24.4, 24.0, 23.0, 22.7, 21.2, 19.5, 18.9, 12.0.
m.p.: 123-125 °C (lit.[16] 122.5-124.5)
Spectroscopic data are in accordance with the literature.[17]

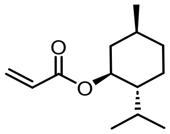

(1S,2R,5S)-2-isopropyl-5-methylcyclohexyl acrylate (2t).
The crude was purified via flash chromatography on silica (Hexane/Ethyl Acetate 92:8) to afford the product as colourless liquid (2.7 g, quantitative yield).
^{1}H NMR (400 MHz,$CDCl_3$**)** δ 6.37 (dd, *J* = 17, 2 Hz, 1H), 6.09 (dd, *J* = 17, 10 Hz, 1H), 5.78 (dd, *J* = 10, 2 Hz, 1H), 4.75 (td, *J* = 11, 4 Hz, 1H), 2.05 – 1.98 (m, 1H), 1.91 – 1.82 (m, 1H), 1.72 – 1.64 (m, 2H), 1.57 – 1.36 (m, 2H), 1.13 – 0.94 (m, 2H), 0.92 – 0.86 (m, 7H), 0.76 (d, *J* = 7 Hz, 3H). **^{13}C NMR** (101 MHz, $CDCl_3$) δ 166.0, 130.3, 129.2, 74.4, 47.2, 41.0, 34.4, 31.5, 26.5, 23.7, 22.2, 20.8, 16.6.
Spectroscopic data are in accordance with the literature.[18]

*Synthesis of methanesulfonylalkynes **2v-2ab***

Compounds **2v-2ab** were prepared by following a procedure reported in the literature.[19]

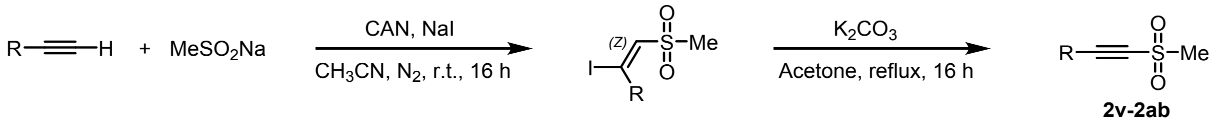

2v-2ab

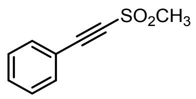

((Methylsulfonyl)ethynyl)benzene (2v).
The crude was purified via flash chromatography on silica (Hexane/Ethyl Acetate 8:2) to afford the product as a pale yellow solid (220 mg, 49% after isolation).
^{1}H NMR (400 MHz, $CDCl_3$) δ 7.62 – 7.56 (m, 2H), 7.56 – 7.48 (m, 1H), 7.45 – 7.38 (m, 2H), 3.30 (s, 3H). **^{13}C NMR** (101 MHz, $CDCl_3$) δ 133.0, 131.9, 128.9, 117.6, 91.7, 84.5, 46.9.
m.p.: 63–65 °C, (lit.[19] 65–66 °C)
Spectroscopic data are in accordance with the literature.[19]

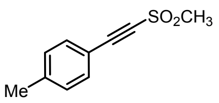

1-Methyl-4-((methylsulfonyl)ethynyl)benzene (2w).
The crude was purified via flash chromatography on silica (PET/Ethyl Acetate 7:3) to afford the product as a pale brown solid (231 mg, 47% after isolation).
^{1}H NMR (400 MHz, $CDCl_3$) δ 7.48 (d, *J* = 8 Hz, 2H), 7.22 (d, *J* = 8 Hz, 2H), 3.29 (s, 3H), 2.40 (s, 3H).**^{13}C NMR (**101 MHz, $CDCl_3$) δ 142.8, 133.0, 129.7, 114.5, 92.4, 84.2, 47.0, 21.9.
m.p.: 66–68 °C (lit.[19] 65-67 °C)
Spectroscopic data are in accordance with the literature.[19]

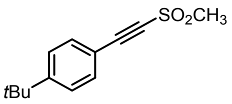

1-(Tert-butyl)-4-((methylsulfonyl)ethynyl)benzene (2x).
The crude was purified via flash chromatography on silica (Cyclohexane/Ethyl Acetate 9:1) to afford the product as an orange-brown solid (102 mg, 17% after isolation).
^{1}H NMR (400 MHz, $CDCl_3$) δ 7.53 (d, *J* = 9 Hz, 2H), 7.43 (d, *J* = 9 Hz, 2H), 3.30 (s, 3H), 1.32 (s, 9H). **^{13}C NMR** (101 MHz, $CDCl_3$) δ 155.8, 132.9, 126.1, 114.5, 92.4, 84.2, 47.0, 35.4, 31.1.
m.p.: 70-72 °C.
Spectroscopic data are in accordance with the literature.[20]

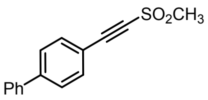

4-((Methylsulfonyl)ethynyl)-1,1'-biphenyl (2y).
The crude was purified via flash chromatography on silica (Cyclohexane/Ethyl Acetate 7:3) to afford the product as a pale yellow solid (222 mg, 34% after isolation).

^{1}H NMR (400 MHz, $CDCl_3$) δ 7.69 – 7.63 (m, 4H), 7.62-7.57 (m, 2H), 7.48 (t, J = 8 Hz, 2H), 7.44 – 7.38 (m, 1H), 3.32 (s, 3H).
^{13}C NMR (101 MHz, $CDCl_3$) δ 144.7, 139.6, 133.5, 129.2, 128.6, 127.6, 127.3, 116.2, 91.9, 85.1, 47.0.
m.p.: 141-143 °C (lit.[19] 143-145 °C)
Spectroscopic data are in accordance with the literature.[19]

1-((Methylsulfonyl)ethynyl)-4-(trifluoromethyl)benzene (2z).

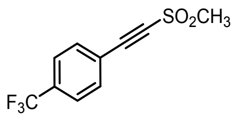

The crude was purified via flash chromatography on silica (Pentane/Ethyl Acetate 7:3) to afford the product as a yellow solid (121 mg, 20% after isolation).
^{1}H NMR (400 MHz, $CDCl_3$) δ 7.76 – 7.66 (m, 4H), 3.32 (s, 3H). **^{13}C NMR** (101 MHz, $CDCl_3$) δ 133.7 – 132.9 (m), 133.3, 129.7 – 124.6 (m) 126.0 (d, *J* = 3.9 Hz)121.5 (t, *J* = 58 Hz), 89.2, 86.2, 46.9. **^{19}F NMR** (565 MHz, $CDCl_3$) δ -62.8.
m.p.: 89-91 °C (lit.[19] 91-93 °C)
Spectroscopic data are in accordance with the literature.[19]

1-Bromo-4-((methylsulfonyl)ethynyl)benzene (2aa).

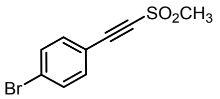

The crude was purified via flash chromatography on silica (PET/Ethyl Acetate 7:3) to afford the product as a pale orange solid (206 mg, 32% after isolation).
^{1}H NMR (400 MHz, $CDCl_3$) δ 7.57 (d, *J* = 9 Hz, 2H), 7.45 (d, *J* = 9 Hz, 2H), 3.30 (s, 3H). **^{13}C NMR** (101 MHz, $CDCl_3$) δ 134.2, 132.4, 126.9, 116.5, 90.3, 85.5, 46.9.
m.p.: 113–115 °C (lit.[19] 116-118 °C)
Spectroscopic data are in accordance with the literature.[19]

1-Methoxy-4-((methylsulfonyl)ethynyl)benzene (2ab).

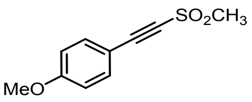

The crude was purified via flash chromatography on silica (Pentane/Ethyl Acetate 7:3) to afford the product as a yellow oil (127 mg, 50% after isolation).
^{1}H NMR (400 MHz, $CDCl_3$) δ 7.53 (d, *J* = 9 Hz, 2H), 6.91 (d, *J* = 9 Hz, 2H), 3.85 (s, 3H), 3.28 (s, 3H). **^{13}C NMR** (101 MHz, $CDCl_3$) δ 162.5, 135.0, 114.7, 109.2, 92.8, 83.9, 55.6, 47.0.
Spectroscopic data are in accordance with the literature.[19]

5. Optimization of reaction conditions

5.1. Hydroalkylation of activated olefins

For aldehydes **1a-1d, 1f, 1g**: The optimization of the reaction conditions was carried out by studying the radical addition of *tert*-butoxy acetaldehyde (**1a**) onto dimethyl maleate (**2b**) to give dimethyl-2-(tert-butoxymethyl)succinate (**4**) on a 0.1 mmol scale (see Table S1). In a 4 mL vial equipped with a screw cap, **1a** (1.2 equiv.), **2b** (0.1 mmol) and TBADT (n mol%) were dissolved in CH_3CN (0.1 M). The mixture was irradiated and for the indicated time with a 40 W Kessil lamp (λ = 390 or 370 nm, full intensity) in Reactor A (see Figure S1A). After irradiation, the vial was opened to air and the solvent was removed under reduced pressure. Next, the crude was suspended in 750 μL of EtOAc, bicyclohexyl (0.1 mmol, 19.2 μL) was added and the mixture was filtered through a short cotton plug in a Pasteur pipette. The filtrate was analyzed via GC-FID. GC-Yields were determined through calibration curves obtained with authentic samples of the relevant compounds. GC-FID analyses were performed on an Agilent 7820A instrument. The injection was performed at 250 °C in split mode. The initial oven temperature of 80 °C was maintained for 2 min, increased by 10 °C/min to 250 °C and held for 5 min. An Agilent HP5 30 m × 0.32 mm × 0.25 μm film thickness capillary column was used with nitrogen as the carrier gas at a constant flow rate of 6.0 mL·min^{-1}.

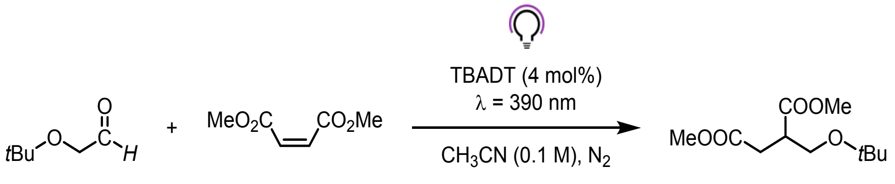

Table S1. Optimization of the Giese Reaction for aldehyde 1a.

Entry	TBADT (mol%)	λ (nm)	time (h)	Atmosphere	Yield [a]
1	none	390	16	N_2	nd
2	2	390	16	N_2	56%
3	2	390	24	N_2	64%
4	2	390	40	N_2	82%
**5**	**4**	**390**	**16**	**N_2**	**85% (76%)[b]**
6	4	370	16	N_2	76%
7	4	427	16	N_2	traces
8	4	390	16	Ar	88%
9	4	390	16	air	traces
10	4	390	16	O_2	nd

[a] Yield evaluated via GC-FID using bicyclohexyl as internal standard; [b] Yield after isolation. nd: not detected.

For aldehyde **1h**. The optimization of the reaction conditions was carried out by studying the radical addition of Garner's aldehyde (**1h**) onto freshly distilled methyl vinyl ketone (**2h**) to give *tert*-butyl 2,2-dimethyl-4-(3-oxobutyl)oxazolidine-3-carboxylate (**21**) on a 0.1 mmol scale (see Table S2). In a 7 mL vial equipped with a screw cap and a stirring bar, **1h** (n equiv.) and TBADT (n mol%) were dissolved in CH_3CN (0.1 M). The mixture was N_2-bubbled (1 min) and the vial was sealed. **2h** (0.1

mmol) was added through the septum via syringe. The mixture was sonicated (1 min), then it was irradiated and stirred for the indicated time with a 40 W Kessil lamp (λ = 390 or 370 nm, full intensity) in the UFO reactor (Figure S1C). After irradiation, the vial was opened to air (NB: CO is toxic, carry out this operation under a fumehood) and the solvent was removed under reduced pressure. Next, the crude was suspended in 600 μL of $CDCl_3$, CH_2Br_2 (0.1 mmol, 7 μL) was added and the mixture was sonicated and filtered through a short cotton plug in a Pasteur pipette. The filtrate was analyzed via ^{1}H-NMR.

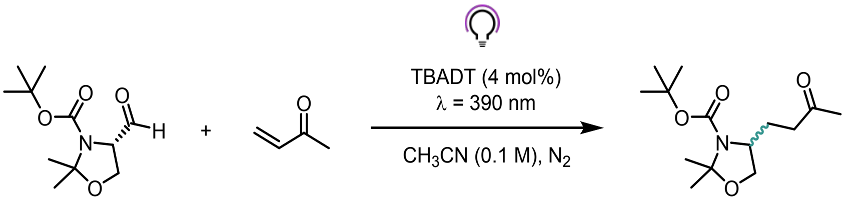

1h **2h** **21**

Table S2. Optimization of the Giese Reaction for aldehyde 1h.

Entry	1h (equiv.)	2h (equiv.)	TBADT (mol%)	λ (nm)	time (h)	Yield [a]
1	1	1	4	390	3	50%
2	1	1	4	390	6	60%
3	1	1	4	390	16	68%
**4**	**1.2**	**1**	**4**	**390**	**16**	**73% (66%)[b,c]**
5	1.2	1	2	390	16	64%
6	1	1.5	4	390	16	48%
7	1.2	1	4	390	3	65%
8	1.2	1	4	390	6	67%
9	1.2	1	4	370	3	70%
10	1.2	1	2	370	3	65%

[a] Yield evaluated via ^{1}H NMR using CH_2Br_2 as internal standard; [b] Yield after isolation. [c] When fluorenone or benzophenone (4 mol%) were used as photocatalyst in place of TBADT, product **21** was observed only in traces.

5.2. Hydroalkylation of unactivated olefins

The optimization of the reaction conditions was carried out by studying the reaction of **1e** with **2j** to give methyl 2-cyclohexyl-4-phenylbutanoate (**15**) on a 0.2 mmol scale (see Table S). In a 4 mL vial equipped with a screw cap, formyl ester **1e**, styrene, additive and TBADT (n mol%) were dissolved in CH_3CN. The mixture was N_2-bubbled (1 min) and the vial was sealed. Next, the mixture was irradiated and stirred for the indicated time with a 40 W Kessil lamp (λ = 390 nm, full intensity) in the UFO reactor (Figure S1. Photoreactors used in this work for batch irradiations. Figure S1C). After irradiation, the vial was opened to air (NB: CO is toxic, carry out this operation under a fumehood) and the solvent was removed under reduced pressure. Next, the crude was suspended in 600 μL of $CDCl_3$, CH_2Br_2 (0.1 mmol, 7 μL) was added and the mixture was sonicated and filtered through a short cotton plug in a Pasteur pipette. The filtrate was analyzed via ^{1}H-NMR.

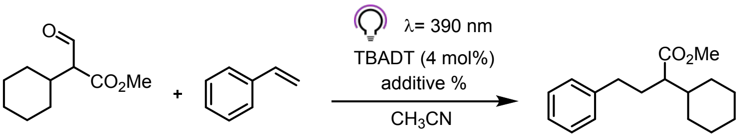

1e **2j** **15**

Table S3. Optimization of the hydroalkylation for aldehyde 1e.

Entry	1e (equiv)	2j (equiv)	Conc.	Additive	Additive %	Yield [a]
1	3	1	CH_3CN 0.2M	$(PhS)_2$	20%	41%
2	3	1	CH_3CN 0.2M	PhSH	20%	38%
3	1.2	1	CH_3CN 0.2M	$(PhS)_2$	20%	21%
4	1	1.2	CH_3CN 0.2M	$(PhS)_2$	20%	24%
5	1	3	CH_3CN 0.2M	$(PhS)_2$	20%	30%
6	2	1	CH_3CN 0.2M	$(pOMePhS)_2$	20%	42%
**7**	**3**	**1**	**CH_3CN 0.2M**	**$(pOMePhS)_2$**	**20%**	**65% (60%)**
8	3	1	CH_3CN 0.4M	$(pOMePhS)_2$	20%	48%
9	2	1	CH_3CN 0.2M	$(pOMePhS)_2$	20%	42%
10	3	1	CH_3CN 0.2M	$(pOMePhS)_2$	40%	31%

[a] Yield evaluated via ^{1}H-NMR using CH_2Br_2 as internal standard; [b] Yield after isolation.

5.3. SOMOphilic alkynylation

The optimization of the reaction conditions was carried out by studying the radical addition of Garner's aldehyde (**1h**) onto 4-((methylsulfonyl)ethynyl)-1,1'-biphenyl (**2y**) to give tert-butyl 4-([1,1'-biphenyl]-4-ylethynyl)-2,2-dimethyloxazolidine-3-carboxylate (**38**) on a 0.1 mmol scale (see Table S4). In a 7 mL vial equipped with a screw cap and a stirring bar, **1h** (n equiv.), TBADT (n mol%), the base (n equiv.) when used, and **2y** (0.1 mmol) were dissolved in CH_3CN (0.1 M). The mixture was N_2-bubbled (1 min) and the vial was sealed. The mixture was sonicated (1 min), then it was irradiated and stirred for the indicated time with a 40 W Kessil lamp (λ = 390, full intensity) in the UFO reactor (Figure S1C). After irradiation, the vial was opened to air (NB: CO is toxic, carry out this operation under a fumehood) and the solvent was removed under reduced pressure. Next, the crude was suspended in 600 μL of $CDCl_3$, CH_2Br_2 (0.1 mmol, 7 μL) was added and the mixture was sonicated and filtered through a short cotton plug in a Pasteur pipette. The filtrate was analyzed via ^{1}H-NMR.

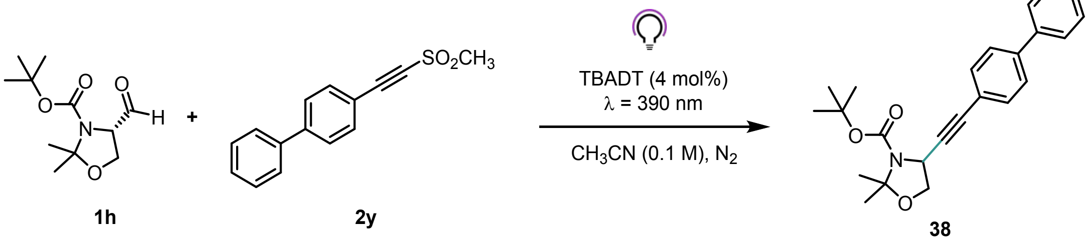

Table S4. Optimization of the SOMOphilic Alkynylation.

Entry	1h (equiv.)	2y (equiv.)	TBADT (mol%)	time (h)	Yield[a]
1	1.2	1	4	16	59%
2	1	1	4	16	43%
3	1	1	2	16	22%
4	1	1	4	4	58%
**5**	**1.2**	**1**	**4**	**4**	**60%, (52%)[b]**
6	1.2	1	4	2	58%
7	2	1	4	2	65%

8	1	1	4	6	48%
9	1.2	1	8	4	55%
10[c]	1.2	1	4	4	45%

[a] Yield evaluated via ^1H NMR using CH_2Br_2 as internal standard; [b] Yield after isolation. [c] 2 equiv. of $NaHCO_3$ were used to quench developing CH_3SO_2H.

5.4. Adjustment of reaction conditions for continuous-flow

The optimization of the reaction conditions was carried out by studying the radical addition of Garner's aldehyde (**1h**) onto freshly distilled ethyl acrylate (**2n**) to give *tert*-butyl 4-(3-ethoxy-3-oxopropyl)-2,2-dimethyloxazolidine-3-carboxylate (**24**) on a 0.1 mmol scale (see Table S5). Thus, 10 mL of a CH_3CN stock solution of **1h** (0.12 M) and TBADT ($4{\cdot}10^{-3}$ M) were prepared in a volumetric flask and N_2-bubbled for 5 minutes. The flask was sealed with a septum and ethyl acrylate (**2n**, 0.1 M) was added through a needle. Volume was taken up to the mark with degassed CH_3CN. The solution was thoroughly shaken and, under positive pressure of N_2 (balloon), 1 mL was withdrawn with a disposable syringe and mounted on a syringe pump. The solution was pushed into a 2.5 mL FEP photoreactor at different flow rates (mL·min^{-1}) and irradiated with a Kessil lamp at 390 nm (100%) by utilizing the Uflow reactor (Figure S2). The crude was collected at the end of the photoreactor in a round-bottomed flask under air, the solvent was removed under reduced pressure and the crude was analyzed via ^{1}H-NMR as described for batch experiments.

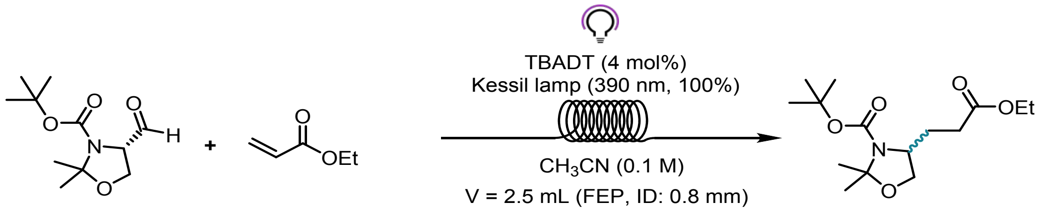

1h 1.5 eq. **2n** 0.1 mmol **24**

Table S5. Optimization of the Giese reaction in continuous-flow.

Entry	f.r. (mL/min)	BPR (psi)	t_R (min)	Yield[a]
1	0.5	-	5	50%[b]
2	0.25	-	10	63%[b]
3	0.125	-	20	72%[b]
4	0.083	-	30	78%[b]
**5**	**0.125**	**40**	**20**	**80%**

[a] Yield evaluated via ^1H NMR using CH_2Br_2 as internal standard; [b] irregular flow was observed, resulting in unpredictable residence time.

Conditions shown in Entry 5 were adopted for the scale-up for compound **30** (see Section 6.4)

6. General procedures for preparative experiments

6.1. Hydroalkylation of activated and unactivated olefins

GP1 (compounds **3-14, 19, 20**): the desired aldehyde (1.2 equiv.), the electron poor olefin (0.5 mmol) and TBADT (4 mol%, 0.02 mmol, 66.6 mg) were dissolved in CH_3CN (0.1 M). The mixture was N_2-bubbled (2 min) and the vessel was sealed. Then it was irradiated using Reactor B shown in Figure S1B with a 40 W Kessil lamp (λ = 390 nm, full intensity). After irradiation, the vessel was opened to air and the reaction mixture was directly adsorbed on silica to be purified by flash chromatography to afford the products **3-14, 19, 20**.

GP2 (compounds **15-18**): **1e** (110.5 mg, 3 equiv.), the olefin (0.2 mmol) and TBADT (4 mol%, 0.008 mmol, 26.6 mg) were dissolved in CH_3CN (0.2 M). The mixture was N_2-bubbled (1 min) and the vessel was sealed. The mixture was irradiated using the reactor shown in Figure S1C with a 40 W Kessil lamp (λ = 390 nm, full intensity). After irradiation, the vessel was opened to air and the reaction mixture was directly adsorbed on silica to be purified by flash chromatography to afford the products **15-18**.

GP3 (compounds **21-34**): In a 7 mL vial equipped with a screw cap and a stirring bar, **1h** (1.2 equiv., 55.0 mg) and TBADT (4 mol%, 0.008 mmol, 26.6 mg) were dissolved in CH_3CN (0.1 M). The mixture was N_2-bubbled (2 min) and the vial was sealed. The electron poor olefin (0.2 mmol) was added through the septum via syringe (if solid, it was inserted before with other reagents). The mixture was sonicated (2 min), then it was irradiated and stirred for 16 hours with a 40 W Kessil lamp (λ = 390 nm, full intensity) in the UFO reactor (Figure S1C). After irradiation, the vial was opened to air (NB: CO is toxic, carry out this operation under a fumehood) and the solvent was removed under reduced pressure. Next, the crude was purified by flash chromatography on silica to afford the products (**21-34**). Diastereomeric ratios were determined via GC-MS or NMR analysis as indicated.

6.2. SOMOphilic alkynylation scope

GP4: In a 7 mL vial equipped with a screw cap and a stirring bar, **1h** (1.2 equiv., 55.0 mg), TBADT (4 mol%, 0.008 mmol, 26.6 mg) and the substituted methanesulfonylalkyne (**2v-2ab**, 0.2 mmol, 1 equiv., if liquid it was added through the septum of the sealed vial via syringe) were dissolved in CH_3CN (0.1 M). The mixture was N_2-bubbled (2 min) and the vial was sealed. The mixture was sonicated (2 min), then it was irradiated and stirred for 4 hours with a 40 W Kessil lamp (λ = 390, full intensity) in the UFO reactor (Figure S1C). After irradiation, the vial was opened to air (NB: CO is toxic, carry out this operation under a fumehood) and the solvent was removed under reduced

pressure. Next, the crude was purified by flash chromatography on silica to afford the products (**35-41**). They were then characterized by ^{1}H and ^{13}C NMR techniques.

*6.3. Scale-up for the synthesis of compound **30** in continuous-flow*

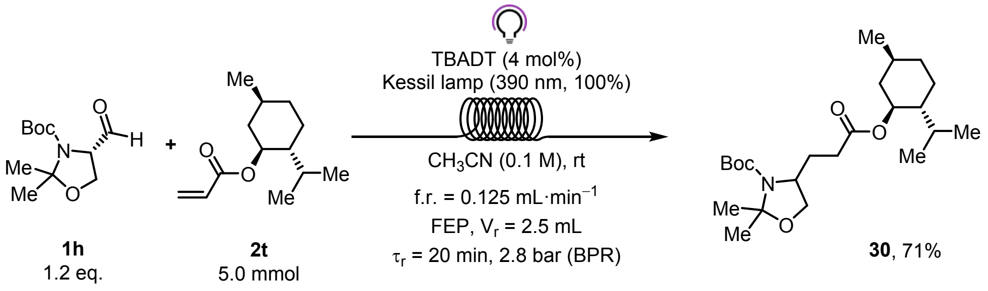

In an oven-dried 100 mL Schlenk-type round-bottom flask, a CH_3CN solution of the Garner's aldehyde (**1h**, 0.1 M, 6.0 mmol, 1.37 g, 1.2 equiv.), menthyl acrylate (**2t**, 1.05 g, 5.0 mmol, 1.0 equiv.) and TBADT (4 mol%, 0.2 mmol, 664 mg) was prepared. The solution was freeze-pump-thawed (3 cycles) to remove air. The mixture was swirled until homogenous, taken up with a 25 mL disposable syringe and mounted on a syringe pump. The flow rate was set at 0.125 mL·min^{-1}, corresponding to a residence time of 20 min (V = 2.5 mL), and the pump was started. When empty, the syringe was replaced with one containing fresh solution. When all the starting solution was injected into the photoreactor, again pure acetonitrile was loaded into a syringe and injected to collect all product at the end of the reactor in a flask. The solvent was removed under reduced pressure and purified via column chromatography on silica gel.

NB: a BPR (40 psi) was mounted at the end of the tubular reactor to maintain a stable continuous flow.

6.4. Opening of the oxazolidine ring

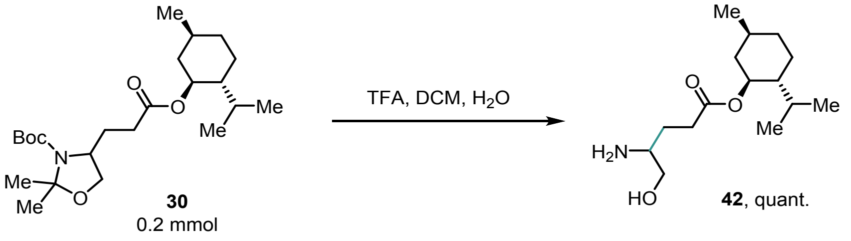

Procedure to open the oxazolidine ring and obtain the deprotected 1,2-aminoalchol. A procedure from literature[21] was followed and adapted on 0.1 mmol scale. To a solution of **30** (82 mg, 0.2 mmol, 1 equiv.) in CH_2Cl_2 (2 mL) was added H_2O (0.2 mL) and trifluoroacetic acid (TFA) (0.5 mL). The reaction was stirred vigorously for 24 h at room temperature. The solvent was removed under reduced pressure to give a light red oil. The crude was purified by column chromatography on silica gel ($CHCl_3$/MeOH 9:1) to afford the corresponding 1,2 aminoalcohol **42** as a pale yellow-foam (54 mg, quantitative yield, dr 1:1).

7. Additional control experiments

This section gathers additional experiments that we conducted to prove the generality of the decarbonylative strategy for direct HAT.

7.1. Steering regioselectivity

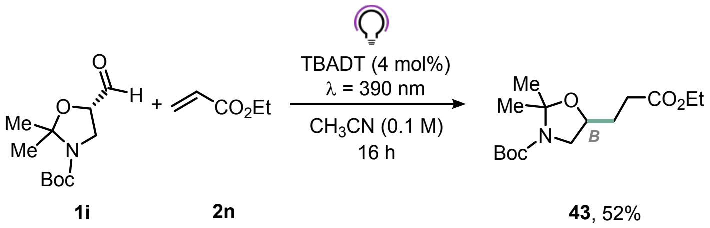

Scheme S1: Use of aldehyde 1i as H-donor for the decarbonylative Giese-type reaction.

To further prove that the decarbonylative strategy proposed in this work can be used to steer regioselectivity in photocatalyzed HAT, we synthesized aldehyde **1i** (*tert*-butyl (S)-5-formyl-2,2-dimethyloxazolidine-3-carboxylate). Thus, **1i** (55.0 mg, 0.24 mmol, 1.2 equiv.) as H-donor was reacted with freshly distilled ethyl acrylate (**2n**, 1 equiv., 0.2 mmol, 22 µL) as radical trap (Scheme S1) according to general procedure **GP3**. The crude was then purified by flash chromatography on silica (Hexane/Ethyl Acetate 8:2) to afford product **43** as a pale yellow oil (31.4 mg, 52% after isolation).

This experiment provides additional evidence that the proposed strategy can be utilized to control regioselectivity. Moreover, it shows that, in addition to 2-substituted amino alcohols (as illustrated in Figure 3 of the main text), the approach can also be applied to obtain 1-substituted amino alcohols.

7.2. Further example

A further example of Giese reaction is reported (Scheme S2): this experiment was conducted with *N*-Boc-(methylamino)acetaldehyde **1g** as hydrogen donor and allowed to obtain product **20** in a good yield.

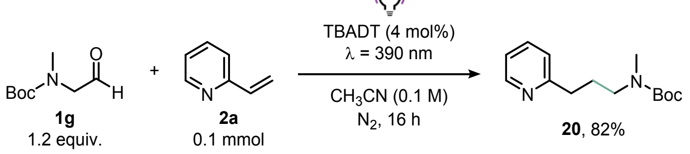

Scheme S2: Further examples of Giese reactions, with aldehyde 1g as H donor.

8. Characterization data for products **3-43**

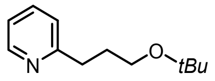

2-(3-(tert-Butoxy)propyl)pyridine (3)

The crude was purified by flash chromatography on silica (Cy/EtOAc 9:1) to afford the product as a pale yellow oil (76.5 mg, 79% after isolation). **^{1}H NMR (300 MHz, $CDCl_3$)** δ 8.49 (d, *J* = 5.9 Hz, 1H), 7.54 (td, *J* = 7.7, 1.9 Hz, 1H), 7.14 (d, *J* = 7.8 Hz, 1H), 7.09-7.02 (m, 1H), 3.36 (t, *J* = 6.4 Hz, 2H), 2.91 – 2.75 (m, 2H), 2.07 – 1.80 (m, 2H), 1.15 (s, 9H). **^{13}C NMR (75 MHz, $CDCl_3$)** δ 162.2, 149.3, 136.3, 122.9, 121.0, 72.6, 60.9, 35.1, 30.6, 27.7.
HRMS (ESI) m/z calcd for $C_{12}H_{20}NO^+$: $[M+H]^+$ 194.1539; found: 194.1538.

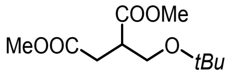

Dimethyl-2-(tert-butoxymethyl)succinate (4)

The crude was purified by flash chromatography on silica (Cy/EtOAc 9:1) to afford the product as a pale yellow oil (88.1 mg, 76% % after isolation). **^{1}H NMR (300 MHz, $CDCl_3$)** δ 3.68 (s, 3H), 3.66 (s, 3H), 3.57 – 3.39 (m, 2H), 3.12 – 2.93 (m, 1H), 2.76-2.58 (m, 2H), 1.12 (s, 9H). **^{13}C NMR (75 MHz, $CDCl_3$)** δ 173.6, 172. 8, 73.2, 61.8, 52.0, 51.8, 42.6, 33.1, 27.4.
Spectroscopic data are in accordance with the literature.[22]

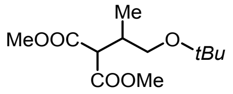

dimethyl 2-(1-(tert-butoxy)propan-2-yl)malonate (5)

The crude was purified by flash chromatography on silica (Cy/EtOAc 9:1) to afford the product as a pale yellow oil (88.2 mg, 72% after isolation). **^{1}H ^{1}H NMR (300 MHz, $CDCl_3$)** δ 3.69 (s, 6H), 3.51 (d, *J* = 7.4 Hz, 1H), 3.30-3.20 (m, 2H), 2.55-2.37 (m,1H), 1.11 (s, 9H), 0.97 (d, *J* = 6.9 Hz, 3H).
^{13}C NMR (75 MHz, $CDCl_3$) δ 169.6, 169.4, 72.7, 64.2, 54.0, 52.2, 52.2, 34.5, 27.4, 14.7.
HRMS (ESI) m/z calcd for $C_{12}H_{22}O_5Na^+$: $[M+Na]^+$ 269.1359; found: 269.1354.

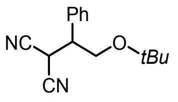

2-(2-(tert-Butoxy)-1-phenylethyl)malononitrile (6)

The crude was purified by flash chromatography on silica (Cy/EtOAc 9:1) to afford the product as a pale yellow oil (90.0 mg, 74% % after isolation). **^{1}H NMR (300 MHz, $CDCl_3$)** δ 7.41 (s, 5H), 4.47 (d, *J* = 5.7 Hz, 1H), 3.86 – 3.68 (m, 2H), 3.42-3.35 (m, 1H), 1.25 (s, 9H). **^{13}C NMR (75 MHz, $CDCl_3$)** δ 135.1, 129.3, 129.3, 128.4, 112.5, 111.9, 74.3, 61.3, 47.0, 27.5, 26.4.
HRMS (ESI) m/z calcd for $C_{15}H_{19}N_2O^+$: $[M+H]^+$ 243.1492; found: 243.1493.

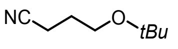

4-(tert-Butoxy)butanenitrile (7)

The crude was purified by flash chromatography on silica (Cy/EtOAc 9:1) to afford the product as a pale yellow oil (40.2 mg, 57% after isolation). **^{1}H-NMR (300 MHz, $CDCl_3$)** δ 3.45 (t, *J* = 5.7 Hz, 2H), 2.45 (t, *J* = 7.0 Hz, 2H), 1.92 – 1.78 (m, 2H), 1.19 (s, 9H). **^{13}C NMR (75 MHz, $CDCl_3$)** δ 119.5, 72.71, 58.6, 27.2, 26.1, 13.8.
HRMS (ESI) m/z calcd for $C_8H_{16}NO^+$: $[M+H]^+$ 142.1226; found: 142.1227.

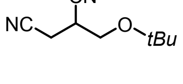

2-(tert-Butoxymethyl)succinonitrile (8)

The crude was purified by flash chromatography on silica (Cy/EtOAc 9:1) to afford the product as a pale yellow oil (56.3 mg, 68% after isolation). **^{1}H NMR (300 MHz, $CDCl_3$)** δ 3.87 – 3.50 (m, 2H), 3.12-2.97

(m,1H), 2.97 – 2.63 (m, 2H), 1.22 (s, 9H) **^{13}C NMR (75 MHz, CDCl$_3$)** δ 117.7, 115.8, 74.7, 59.8, 29.7, 27.4, 18.0.
HRMS (ESI) m/z calcd for $C_9H_{14}N_2ONa^+$: $[M+Na]^+$ 189.0998; found: 189.0996.

((3-(tert-Butoxy)propyl)sulfonyl)benzene (9)

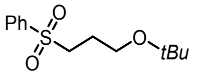

The crude was purified by flash chromatography on silica (Cy/EtOAc 9:1) to afford the product as a pale yellow oil (92.4 mg, 72% after isolation). **^{1}H NMR (300 MHz, CDCl$_3$)** δ 7.90 (d, *J* = 8.2 Hz, 2H), 7.77 – 7.49 (m, 3H), 3.37 (t, *J* = 5.9 Hz, 2H), 3.25 – 3.13 (m, 2H), 2.06 – 1.82 (m, 2H), 1.11 (s, 9H).**^{13}C NMR (75 MHz, CDCl$_3$)** δ 139.3, 133.7, 129.5, 128.2, 73.1, 59.4, 53.8, 27.5, 24.2**.**
Spectroscopic data are in accordance with the literature.[23]

3-(2-(tert-Butoxy)ethyl)bicyclo[2.2.1]heptan-2-one (10)

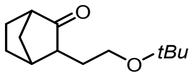

The crude was purified by flash chromatography on silica (Cy/EtOAc 9:1) to afford the product as a pale yellow oil (74.5 mg, 71% after isolation). **^{1}H NMR (300 MHz, CDCl$_3$** δ 3.43 (t, J = 6.6 Hz, 2H), 2.65 – 2.57 (m, 2H), 2.14 (quin, J = 4.6 Hz, 1H), 1.93 – 1.73 (m, 2H), 1.72 – 1.33 (m, 6H), 1.18 (s, 9H). **^{13}C NMR (75 MHz, CDCl$_3$)** δ 220.29, 72.77, 60.16, 50.97, 50.70, 39.13, 37.30, 27.90, 27.73, 25.52, 21.58.
HRMS (ESI) m/z calcd for $C_{13}H_{22}O_2Na^+$: $[M+Na]^+$ 233.1512; found: 233.1511.

2-(2-(2,2-Dimethyl-1,3-dioxolan-4-yl)ethyl)pyridine (11)

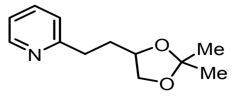

The crude was purified by flash chromatography on silica (Cy/EtOAc 9:1) to afford the product as a pale yellow oil (86 mg, 83% after isolation). **^{1}H NMR (300 MHz, CDCl$_3$)** δ 8.56 – 8.47 (m, 1H), 7.58 (td, *J* = 7.6, 1.9 Hz, 1H), 7.25 – 7.05 (m, 2H), 4.12 (p, *J* = 6.4 Hz, 1H), 4.07-3.96 (m, 1H), 3.55 (t, *J* = 7.5 Hz, 1H), 3.02 – 2.74 (m, 2H), 2.08-1.94 (m, 2H), 1.41 (s, 3H), 1.35 (s, 3H). **^{13}C NMR (75 MHz, CDCl$_3$)** δ 161.8, 149.9, 136.9, 123.4, 121.7, 109.4, 76.1, 69.9, 35.0, 34.0, 27.5, 26.3.
HRMS (ESI) m/z calcd for $C_{12}H_{18}NO_2^+$: $[M+H]^+$ 208.1332; found: 208.1330.

2-(3-(Benzyloxy)propyl)pyridine (12)

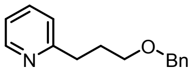

Benzyloxy acetaldehyde was purified by column chromatography prior to use. The crude was purified by flash chromatography on silica (Cy/EtOAc 9:1) to afford the product as a pale yellow oil (60.1 mg, 53% after isolation).
^{1}H NMR (400 MHz, CDCl$_3$) δ δ 8.53 (d, *J* = 4.7 Hz, 1H), 7.57 (td, *J* = 7.6, 1.9 Hz, 1H), 7.38 – 7.25 (m, 5H), 7.19 – 7.05 (m, 2H), 4.51 (s, 2H), 3.53 (t, *J* = 6.4 Hz, 2H), 2.90 (t, *J* = 7.4 Hz,2 H), 2.14 – 2.01 (m, 2H). **^{13}C NMR (101 MHz, CDCl$_3$)** δ 161.86, 149.39, 138.74, 136.43, 128.49, 127.79, 127.65, 123.03, 121.15, 73.01, 69.76, 35.04, 29.84.
Spectroscopic data are in accordance with the literature.[24]

2-(2-(5-Methoxybenzo[1,3]dioxol-2-yl)ethyl)pyridine (13)

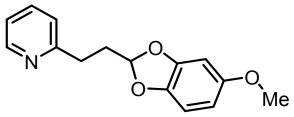

5 equiv. of hydrogen donor instead **S3** were used. The crude was purified by flash chromatography on silica (Cy/EtOAc 9:1) to afford the product as a pale yellow oil (99.1 mg, 86% after isolation). **^{1}H NMR (300 MHz, CDCl$_3$)** δ 8.57 – 8.49 (m, 1H), 7.58 (td, *J* = 7.7, 1.9 Hz, 1H), 7.20 – 7.06 (m, 2H), 6.64 (d, *J* = 8.5 Hz, 1H), 6.43 (d, *J* = 2.5 Hz, 1H), 6.28 (dd, *J* = 8.4, 2.5 Hz, 1H), 6.16 (t, *J* = 4.7 Hz, 1H), 3.72 (s, 3H), 3.07 – 2.95 (m, 2H), 2.46 – 2.34 (m, 2H). **^{13}C NMR (75 MHz, CDCl$_3$)** δ 160.5, 155.1, 149.5, 148.6, 142.1, 136.6, 123.0, 121.4, 111.6, 107.7, 104.4, 97.4, 56.1, 34.2, 31.6.

HRMS (ESI) m/z calcd for $C_{15}H_{16}NO_3^+$: $[M+H]^+$ 258.1125; found: 258.1125.

2-(3-(Benzo[d][1,3]dioxol-5-yloxy)propyl)pyridine (14)

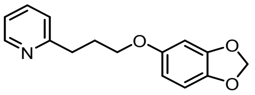

The crude was purified by flash chromatography on silica (Cy/EtOAc 9:1) to afford the product as a pale yellow oil (95.4 mg, 74% after isolation). **^{1}H NMR (300 MHz, $CDCl_3$)** δ 8.54 (d, J = 4.6 Hz, 1H), 7.59 (td, J = 7.7, 1.9 Hz, 1H), 7.17 (d, J = 7.8 Hz, 1H), 7.16 – 7.06 (m, 1H), 6.69 (d, J = 8.5 Hz, 1H), 6.48 (d, J = 2.5 Hz, 1H), 6.31 (dd, J = 8.5, 2.5 Hz, 1H), 5.90 (s, 2H), 3.93 (t, J = 6.3 Hz, 2H), 2.97 (t, J = 6.7 Hz, 2H), 2.37 – 1.96 (m, 2H). **^{13}C NMR (75 MHz, $CDCl_3$)** δ 161.46, 154.70, 149.52, 148.36, 141.68, 136.49, 123.13, 121.28, 108.07, 105.86, 101.22, 98.25, 68.20, 34.82, 29.33.
HRMS (ESI) m/z calcd for $C_{15}H_{16}NO_3^+$: $[M+H]^+$ 258.1125; found: 258.1124.

Methyl 2-cyclohexyl-4-phenylbutanoate (15)

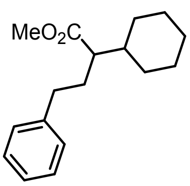

The crude was purified twice by flash chromatography on silica (Hexane/EtOAc 99:1) to afford the product as a colorless oil (31.2 mg, 60% after isolation). **^{1}H NMR** (400 MHz, $CDCl_3$) δ 7.33 – 7.27 (m, 2H), 7.24 – 7.16 (m, 3H), 3.71 (s, 2H), 2.62 (m, 1H), 2.50 (m, 1H), 2.24 (m, 1H), 2.03 – 1.88 (m, 1H), 1.90 – 1.49 (m, 7H), 1.33 – 0.85 (m, 6H). **^{13}C NMR** (101 MHz, $CDCl_3$) δ 176.3, 142.0, 128.5, 128.5, 126.0, 51.7, 51.3, 40.5, 34.3, 31.4, 31.1, 30.7, 26.5, 26.5, 26.4. **HRMS** (ESI) m/z calcd for $C_{17}H_{24}O_2Na^+$: $[M+Na]^+$ 283.1674; found: 283.1672.

Methyl 4-(4-(tert-butyl)phenyl)-2-cyclohexylbutanoate (16).

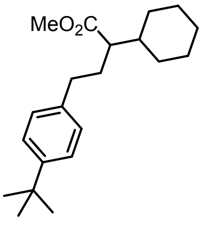

The crude was purified by twice flash chromatography on silica (Hexane/EtOAc 99:1) to afford the product as a colorless oil (39.2 mg, 62% after isolation). **^{1}H NMR** (400 MHz, $CDCl_3$) δ 7.29 (d, *J* = 8.4 Hz, 2H), 7.10 (d, *J* = 8.4 Hz, 2H), 3.68 (s, 3H), 2.56 (m, 1H), 2.45 (m, 1H), 2.23 (m, 1H), 1.92 (m, 1H), 1.86 – 1.58 (m, 7H), 1.30 (s, 9H), 1.26 – 0.84 (m, 5H). **^{13}C NMR** (101 MHz, $CDCl_3$) δ 176.3, 148.8, 138.9, 128.2, 125.3, 51.9, 51.3, 40.5, 34.5, 33.7, 31.5, 31.4, 31.1, 30.8, 26.5, 26.5, 26.4. **HRMS** (ESI) m/z calcd for $C_{21}H_{32}O_2Na^+$: $[M+Na]^+$ 339.2300; found: 339.2304.

Methyl 2-cyclohexyl-4-(pyridin-2-yl)butanoate (17).

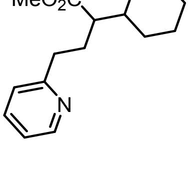

The crude was purified twice by flash chromatography on silica (Hexane/EtOAc 99:1) to afford the product as a colorless oil (30.3 mg, 58% after isolation). **^{1}H NMR** (400 MHz, $CDCl_3$) δ 8.54 (q, *J* = 1.7 Hz, 1H), 7.60 (td, *J* = 7.6, 1.9 Hz, 1H), 7.18 – 7.08 (m, 2H), 3.71 (s, 3H), 2.86 – 2.64 (m, 2H), 2.26 (m, 1H), 2.11 – 1.93 (m, 2H), 1.86 – 1.51 (m, 6H), 1.25 – 0.88 (m, 5H). ^{13}C NMR (101 MHz, $CDCl_3$) δ 176.1, 161.5, 149.3, 136.3, 122.9, 121.1, 51.7, 51.3, 40.3, 36.5, 31.0, 30.6, 29.4, 26.3, 26.3, 26.3. **HRMS** (ESI) m/z calcd for $C_{16}H_{24}NO_2^+$: $[M+H]^+$ 262.1802; found: 262.1809.

Methyl 4-(4-acetoxyphenyl)-2-cyclohexylbutanoate (18).

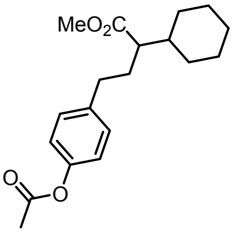

The crude was purified by flash chromatography on silica (Hexane/EtOAc 99:1) to afford the product as a colorless oil (29.9 mg, 47% after isolation). **^{1}H NMR** (400 MHz, $CDCl_3$) δ 7.18 (d, *J* = 8.6 Hz, 2H), 7.00 (d, *J* = 8.6 Hz, 2H), 3.70 (s, 3H), 2.60 (m, 1H), 2.49 (m, 1H), 2.30 (s, 3H), 2.24 (m, 1H), 2.01 – 1.87 (m, 1H), 1.88 – 1.48 (m, 7H), 1.34 – 0.87 (m, 5H). **^{13}C NMR** (101 MHz, $CDCl_3$) δ 176.2, 169.8, 149.0,

139.5, 129.4, 121.5, 51.7, 51.3, 40.4, 33.6, 31.3, 31.1, 30.7, 26.5, 26.4, 26.4, 21.3. **HRMS** (ESI) m/z calcd for $C_{19}H_{26}O_4Na^+$: $[M+Na]^+$ 341.1729; found: 341.1723.

***tert*-Butyl-2-(2-(pyridin-2-yl)ethyl)piperidine-1-carboxylate (19)**

1-N-Boc-2-Piperidinecarbaldehyde was purified by column chromatography prior to use.

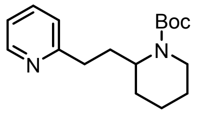

The crude was purified by flash chromatography on silica (Cy/EtOAc 9:1) to afford the product as a pale yellow oil (103.9 mg, 72% after isolation). **^{1}H NMR (400 MHz, $CDCl_3$)** δ 8.50 (dd, *J* = 5.2, 1.9 Hz, 1H), 7.56 (td, *J* = 7.6, 1.9 Hz, 1H), 7.13 (d, *J* = 7.8 Hz, 1H), 7.08 (ddd, *J* = 7.6, 4.8, 1.2 lHz, 1H), 4.31 (bs, 1H), 3.98 (bs, 1H), 2.87 - 2.65 (m, 3H), 2.18 - 1.78 (m, 3H), 1.65 - 1.50 (m, 5H), 1.42 (s, 9H). **^{13}C NMR (101 MHz, $CDCl_3$)** δ 162.1, 155.4, 149.4, 136.6, 123.2, 121.3, 79.4, 50.7, 39.0, 35.5, 30.2, 28.7, 28.6, 25.9, 19.3.

HRMS (ESI) m/z calcd for $C_{17}H_{27}N_2O_2^+$: $[M+H]^+$ 291.2067; found: 291.2065.

***tert*-butyl methyl(3-(pyridin-2-yl)propyl)carbamate (20).**

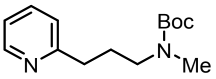

The crude was purified by flash chromatography on silica (Cy/EtOAc 9:1) to afford the product as a pale yellow oil (102.6 mg, 82% % after isolation). **^{1}H NMR (300 MHz, $CDCl_3$)** δ 8.56 – 8.47 (m, 1H), 7.58 (td, *J* = 7.7, 1.9 Hz, 1H), 7.19 – 7.05 (m, 2H), 3.28 (t, *J* = 7.3 Hz, 2H), 2.88 – 2.71 (m, 5H), 1.95 (quint, *J* = 7.6 Hz, 2H), 1.43 (s, 9H). **^{13}C NMR (75 MHz, $CDCl_3$)** δ 161.6, 155.9, 149.4, 136.5, 122.9, 121.2, 79.3, 48.6, 35.6, 34.2, 28.6, 28.0.

HRMS (ESI) m/z calcd for $C_{14}H_{23}N_2O_2^+$: $[M+H]^+$ 251.1754; found: 251.1751.

***tert*-Butyl 2,2-dimethyl-4-(3-oxobutyl)oxazolidine-3-carboxylate (21).**

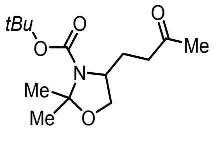

Freshly distilled **2h** was used. The crude was purified by flash chromatography on silica (Hexane/Ethyl Acetate 8:2) to afford the product as a colourless oil (35.8 mg, 66% after isolation).

Mixture of rotamers: **^{1}H NMR** (400 MHz, $CDCl_3$) δ 3.98 – 3.75 (m, 2H), 3.68 (d, *J* = 8 Hz, 1H), 2.45 (t, *J* = 8 Hz, 2H), 2.15 (s, 3H), 2.00 – 1.76 (m, 2H), 1.63-1.51 (m, 3H),1.46 (s, 12H). **^{13}C NMR** (101 MHz, $CDCl_3$) δ 208.2, 207.9, 152.7, 152.1, 94.1, 93.6, 80.2, 79.9, 67.3, 56.8, 56.6, 40.4, 40.2, 30.0, 28.6, 27.7, 26.9, 24.6, 23.2.

HRMS (ESI) m/z calcd for $C_{14}H_{25}NO_4Na^+$: $[M+Na]^+$ 294.1676; found: 294.1675.

***tert*-Butyl 2,2-dimethyl-4-(((1S,2S,4R)-3-oxobicyclo[2.2.1]heptan-2-yl)methyl)oxazolidine-3-carboxylate (22).**

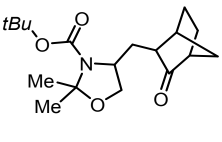

The crude was purified by flash chromatography on silica (Hexane/Ethyl Acetate 8:2) to afford the product as a colourless oil (51 mg, 76% after isolation) as a mixture of diastereomers (dr 1.1:1 via GC-MS analysis).

Mixture of diastereomers and rotamers: **^{1}H NMR** (400 MHz, $CDCl_3$) δ 4.22 – 3.69 (m, 3H), 2.73 – 2.49 (m, 2H), 2.12 – 1.33 (m, 24H). **^{13}C NMR** (151 MHz, $CDCl_3$) δ 219.3, 218.8, 152.6, 152.3, 152.0, 151.8, 94.1, 93.9, 93.6, 93.5, 80.1, 79.9, 79.8, 67.6, 67.2, 66.6, 66.3, 56.6, 56.4, 56.1, 51.4, 51.2, 50.9, 50.3, 39.9, 39.5, 38.4, 37.4, 37.3, 30.6, 30.0, 28.5, 27.7, 27.0, 26.9, 25.4, 25.4, 24.7, 24.6, 23.4, 23.2, 21.8, 21.5, 21.4.

***tert*-Butyl 4-(3-methoxy-3-oxopropyl)-2,2-dimethyloxazolidine-3-carboxylate (23).**

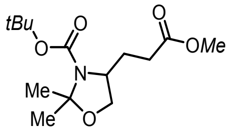

Freshly distilled **2j** was used. The crude was purified by flash chromatography on silica (Hexane/Ethyl Acetate 8:2) to afford the product as a colourless oil (41 mg, 71% after isolation).

Mixture of rotamers: **^{1}H NMR** (400 MHz, $CDCl_3$) δ 3.98-3.77 (m, 2H), 3.69 (d, *J* = 8 Hz, 1H), 3.65 (s, 3H), 2.36-2.26 (m, 2H), 2.08 – 1.81 (m, 2H), 1.59 – 1.50 (m, 3H), 1.45 (s, 12H). **^{13}C NMR** (101 MHz, $CDCl_3$) δ 173.6, 152.5, 152.0, 94.0, 93.5, 80.2, 79.9, 67.0, 66.9, 56.8, 56.5, 51.9, 51.7, 30.9, 30.8, 29.0, 28.5, 27.6, 26.8, 24.5, 23.2.

HRMS (ESI) m/z calcd for $C_{14}H_{25}NO_5Na^+$: $[M+Na]^+$ 310.1625; found: 310.1622.

***tert*-Butyl 4-(3-ethoxy-3-oxopropyl)-2,2-dimethyloxazolidine-3-carboxylate (24).**

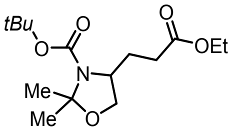

Freshly distilled **2k** was used. The crude was purified by flash chromatography on silica (Hexane/Ethyl Acetate 8:2) to afford the product as a pale yellow oil (47 mg, 78% after isolation).

Mixture of rotamers: **^{1}H NMR** (400 MHz, $CDCl_3$) δ 4.11 (q, *J* = 7 Hz, 2H), 3.98 – 3.79 (m, 2H), 3.71 (d, *J* = 8 Hz, 1H), 2.48 – 2.22 (m, 2H), 2.10 – 1.84 (m, 2H), 1.70 – 1.51 (m, 4H), 1.46 (s, 11H), 1.24 (t, *J* = 7 Hz, 3H). **^{13}C NMR** (101 MHz, $CDCl_3$) δ 173.2, 152.5, 152.0, 94.1, 93.5, 80.3, 80.0, 78.4, 67.1, 67.0, 60.6, 56.9, 56.6, 31.2, 31.1, 29.1, 28.6, 27.7, 26.9, 24.6, 23.3, 14.3.

HRMS (ESI) m/z calcd for $C_{15}H_{27}NO_5Na^+$: $[M+Na]^+$ 324.1781; found: 324.1781.

***tert*-Butyl 4-(3-(*tert*-butoxy)-3-oxopropyl)-2,2-dimethyloxazolidine-3-carboxylate (25).**

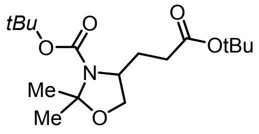

The crude was purified by flash chromatography on silica (Hexane/Ethyl Acetate 8:2) to afford the product as a yellow oil (44.2 mg, 64% after isolation).

Mixture of rotamers: **^{1}H NMR** (400 MHz, $CDCl_3$) δ 3.95 – 3.75 (m, 2H), 3.70 (d, *J* = 7 Hz, 1H), 2.20 (t, *J* = 8, 7 Hz, 2H), 2.04 – 1.74 (m, 2H), 1.59 – 1.49 (m, 3H), 1.45 (s, 12H), 1.41 (s, 9H). **^{13}C NMR** (101 MHz, $CDCl_3$) δ 172.5, 152.4, 152.0, 94.0, 93.4, 80.4, 80.3, 80.1, 79.8, 67.0, 66.9, 57.0, 56.7, 32.4, 29.1, 28.5, 28.2, 27.7, 26.8, 24.5, 23.2.

HRMS (ESI) m/z calcd for $C_{17}H_{31}NO_5Na^+$: $[M+Na]^+$ 352.2094; found: 352.2090.

***tert*-Butyl 4-(3-(furan-2-ylmethoxy)-3-oxopropyl)-2,2-dimethyloxazolidine-3-carboxylate (26).**

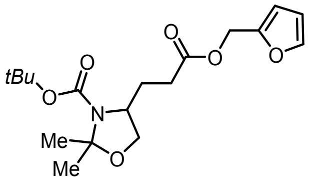

The crude was purified by flash chromatography on silica (Hexane/Ethyl Acetate 85:15) to afford the product as a colourless oil (53.0 mg, 75% after isolation).

Mixture of rotamers: **^{1}H NMR** (400 MHz, $CDCl_3$) δ 7.40 (s, 1H), 6.38 (d, *J* = 3 Hz, 1H), 6.37 – 6.32 (m, 1H), 5.05 (s, 2H), 4.02 – 3.77 (m, 2H), 3.68 (d, *J* = 8 Hz, 1H), 2.48 – 2.25 (m, 2H), 2.09 – 1.82 (m, 2H), 1.61 – 1.49 (m, 3H), 1.45 (s, 12H). **^{13}C NMR** (101 MHz, $CDCl_3$) δ 172.8, 152.5, 152.0, 149.6, 149.5, 143.3, 110.7, 110.7, 94.1, 93.5, 80.3, 79.9, 67.0, 67.0, 58.2, 56.8, 56.5, 30.9, 28.9, 28.5, 27.7, 26.8, 24.5, 23.2.

HRMS (ESI) m/z calcd for $C_{18}H_{27}NO_6Na^+$: $[M+Na]^+$ 376.1731; found: 376.1728.

tert-Butyl 4-(3-(((R)-1-(tert-butoxycarbonyl)pyrrolidin-2-yl)methoxy)-3-oxopropyl)-2,2-dimethyloxazolidine-3-carboxylate (27).

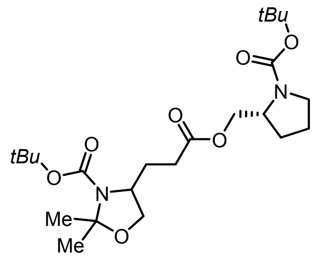

The crude was purified by flash chromatography on silica (Hexane/Ethyl Acetate 8:2) to afford the product as a colourless oil (57.1 mg, 62% after isolation) as a mixture of diastereomers (dr 1:1 via NMR analysis).

Mixture of diastereomers and rotamers: **^{1}H NMR** (400 MHz, $CDCl_3$) δ 4.20 – 3.79 (m, 5H), 3.71 (d, J = 9 Hz, 1H), 3.45 – 3.23 (m, 2H), 2.53 – 2.23 (m, 2H), 2.09 – 1.70 (m, 6H), 1.64 – 1.51 (m, 3H), 1.50 – 1.38 (m, 21H). **^{13}C NMR** (101 MHz, $CDCl_3$) δ 173.0, 154.6, 152.5, 152.0, 94.08, 93.5, 80.3, 79.9, 79.5, 67.0, 64.9, 56.9, 56.6, 55.6, 46.6, 31.1, 31.0, 29.0, 28.9, 28.6, 28.5, 28.0, 27.7, 26.9, 24.5, 23.9, 23.2, 23.1.

tert-Butyl 2,2-dimethyl-4-(3-oxo-3-(((3aR,5R,5aS,8aS)-2,2,7,7-tetramethyltetrahydro-5H-bis([1,3]dioxolo)[4,5-b:4',5'-d]pyran-5-yl)methoxy)propyl)oxazolidine-3-carboxylate (28).

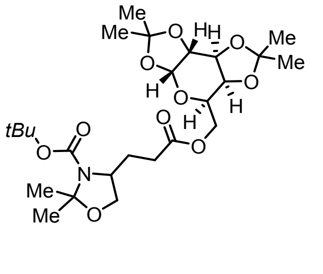

The crude was purified by flash chromatography on silica (Hexane/Ethyl Acetate 8:2) to afford the product as a yellow oil (85.2 mg, 83% after isolation) as a mixture of diastereomers (dr 1:1 via NMR analysis).

Mixture of diastereomers and rotamers: **^{1}H NMR** (400 MHz, $CDCl_3$) δ 5.53 (d, J = 5 Hz, 1H), 4.61 (dd, J = 8, 3 Hz, 1H), 4.37 – 4.14 (m, 4H), 4.04 – 3.98 (m, 1H), 3.97 – 3.80 (m, 2H), 3.72 (d, J = 8 Hz, 1H), 2.43 – 2.31 (m, 2H), 2.11 – 1.83 (m, 2H), 1.62 – 1.53 (m, 4H), 1.50 (s, 4H), 1.47 (s, 10H), 1.45 (s, 3H), 1.33 (d, J = 5 Hz, 6H). **^{13}C NMR** (101 MHz, $CDCl_3$) δ 173.1, 152.5, 109.8, 108.9, 96.4, 94.1, 93.5, 80.3, 79.9, 71.2, 70.8, 70.6, 67.0, 66.1, 63.6, 63.5, 63.4, 56.9, 56.9, 56.6, 31.1, 31.0, 29.8, 29.0, 28.6, 27.7, 26.9, 26.2, 26.1, 25.1, 24.6, 23.3.

tert-Butyl 4-(3-(((3S,8S,9S,10R,13R,14S,17R)-10,13-dimethyl-17-((R)-6-methylheptan-2-yl)-2,3,4,7,8,9,10,11,12,13,14,15,16,17-tetradecahydro-1H-cyclopenta[a]phenanthren-3-yl)oxy)-3-oxopropyl)-2,2-dimethyloxazolidine-3-carboxylate (29).

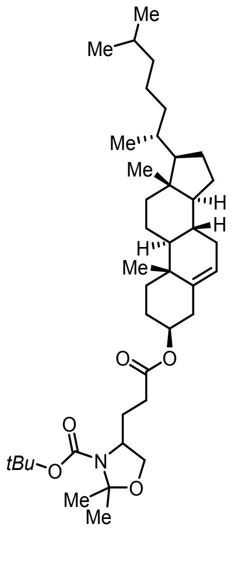

Reaction performed in CH_3CN/CH_2Cl_2 1:1. The crude was purified by flash chromatography on silica (Hexane/Ethyl Acetate 9:1) to afford the product as a pale yellow oil (94.7 mg, 50% after isolation) as a mixture of diastereomers (dr 1:1 via NMR analysis).

Mixture of diastereomers and rotamers: **^{1}H NMR** (400 MHz, $CDCl_3$) δ 5.39 – 5.31 (m, 1H), 4.65 – 4.53 (m, 1H), 4.00 – 3.79 (m, 2H), 3.72 (d, J = 8 Hz, 1H), 2.35 – 2.22 (m, 4H), 2.07 – 1.91 (m, 3H), 1.90 – 1.73 (m, 4H), 1.64 – 1.49 (m, 8H), 1.46 (s, 13H), 1.43 – 1.22 (m, 6H), 1.20 – 1.04 (m, 7H), 1.03 – 0.94 (m, 5H), 0.90 (d, J = 7 Hz, 3H), 0.85 (dd, J = 7, 2 Hz, 6H), 0.66 (s, 3H). **^{13}C NMR** (101 MHz, $CDCl_3$) δ 172.6, 152.5, 152.0, 139.7, 122.8, 94.0, 93.5, 80.2, 79.9, 74.2, 67.1, 67.0, 57.0, 56.8, 56.7, 56.2, 50.1, 42.4, 39.8, 39.6, 38.2, 37.1, 36.7, 36.3, 35.9, 32.0, 32.0, 31.5, 31.4, 29.1, 28.6, 28.4, 28.1, 27.9, 27.7, 26.8, 24.5, 24.4, 23.9, 23.2, 22.9, 22.7, 21.1, 19.4, 18.8, 12.0.

tert-Butyl 4-(3-(((1S,2R,5S)-2-isopropyl-5-methylcyclohexyl)oxy)-3-oxopropyl)-2,2-dimethyloxazolidine-3-carboxylate (30).

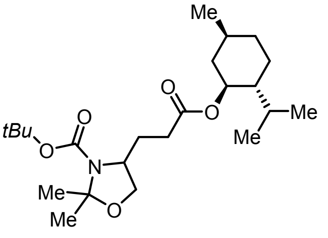

The crude was purified by flash chromatography on silica (Hexane/Ethyl Acetate 8:2) to afford the product as a colourless oil (55.7 mg, 68% after isolation) as a mixture of diastereomers (dr 1:1 via NMR analysis).

Mixture of diastereomers and rotamers: **^{1}H NMR** (400 MHz, $CDCl_3$) δ 4.66 (td, J = 11, 4 Hz, 1H), 4.00 – 3.77 (m, 2H), 3.72 (d, J = 8 Hz, 1H),

2.47 – 2.19 (m, 2H), 2.07 – 1.91 (m, 2H), 1.91 – 1.72 (m, 2H), 1.71 – 1.61 (m, 2H), 1.61-1.51 (m, 3H), 1.46 (s, 12H), 1.40 – 1.22 (m, 2H), 1.09 – 0.91 (m, 2H), 0.90 – 0.80 (m, 7H), 0.73 (d, *J* = 7 Hz, 3H). **^{13}C NMR** (101 MHz, $CDCl_3$) δ 172.7, 152.5, 152.0, 94.1, 93.5, 80.2, 79.9, 74.4, 74.3, 67.0, 57.0, 56.8, 47.1, 41.0, 34.4, 31.5, 29.1, 28.6, 27.7, 26.8, 26.4, 24.6, 23.6, 23.3, 22.1, 20.9, 16.4.

Dimethyl 2-(3-(tert-butoxycarbonyl)-2,2-dimethyloxazolidin-4-yl)succinate (31).

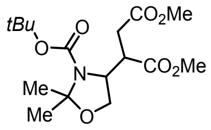

The crude was purified by flash chromatography on silica (Hexane/Ethyl Acetate 8:2) to afford the product as a pale yellow oil (27.4 mg, 40% after isolation) as a mixture of diastereomers (dr 5:1 via GC-MS analysis).

Mixture of diastereomers and rotamers: **^{1}H NMR** (400 MHz, $CDCl_3$) δ 4.34 – 4.18 (m, 1H), 3.99 – 3.80 (m, 2H), 3.76 – 3.63 (m, 6H), 3.60 – 3.46 (m, 1H), 2.86 – 2.71 (m, 1H), 2.51 (dd, *J* = 17, 4 Hz, 1H), 1.69 – 1.55 (m, 3H), 1.52 – 1.46 (m, 9H), 1.44 (s, 3H). **^{13}C NMR** (101 MHz, $CDCl_3$) δ 173.3, 172.9, 172.6, 153.0,152.7, 152.2, 152.1, 94.7, 94.1, 80.9, 80.8, 80.7, 65.5, 64.7, 58.4, 57.8, 57.1, 52.3, 52.2, 52.0, 44.2, 43.5, 43.0, 31.5, 30.5, 28.5, 28.4, 26.9, 26.0, 23.9, 22.5.

tert-Butyl 4-(2,2-dicyano-1-phenylethyl)-2,2-dimethyloxazolidine-3-carboxylate (32).

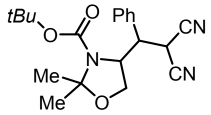

The crude was purified by flash chromatography on silica (Hexane/Ethyl Acetate 8:2) to afford the product as a colourless oil (56 mg, 79% after isolation) as a mixture of diastereomers (dr 1.2:1 via NMR analysis).

Mixture of diastereomers and rotamers: **^{1}H NMR** (400 MHz, $CDCl_3$) δ 7.45 – 7.35 (m, 3H), 7.34-7.28 (m, 1H), 7.20 (bs, 1H), 5.34 – 5.25 (m, 0.5H), 4.64 – 4.41 (m, 1.5H), 4.23 – 4.13 (m, 0.5H minor), 4.06 – 3.97 (m, 0.5H minor), 3.79 (dd, *J* = 10, 6 Hz, 0.5H major), 3.58 – 3.45 (m, 1.5H), 3.37 (dd, *J* = 11, 7 Hz, 0.5H major), 1.65 (s, 2H), 1.56 (s, 5H), 1.53 – 1.47 (m, 7H), 1.35 (s, 1H). **^{13}C NMR** (101 MHz, $CDCl_3$) δ 154.4, 154.1, 135.7, 133.9, 129.7, 129.5, 129.4, 129.1, 129.0, 128.0, 128.6, 113.7, 113.3, 112.6, 112.1, 95.4, 95.2, 82.2, 81.6, 68.0, 65.9, 60.1, 58.5, 52.1, 51.4, 28.5, 28.4, 27.6, 27.3, 25.1, 24.2.

tert-Butyl 2,2-dimethyl-4-(2-(phenylsulfonyl)ethyl)oxazolidine-3-carboxylate (33).

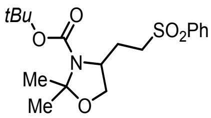

The crude was purified by flash chromatography on silica (Hexane/Ethyl Acetate 7:3) to afford the product as a pale yellow solid (47.8 mg, 65% after isolation).

Mixture of rotamers: **^{1}H NMR** (400 MHz, $CDCl_3$) δ 7.90 (d, *J* = 8 Hz, 2H), 7.71-7.60 (m, 1H), 7.60-7.50 (m, 2H), 4.06 – 3.86 (m, 2H), 3.64 (d, *J* = 9 Hz, 1H), 3.21 – 3.02 (m, 2H), 2.10 – 1.89 (m, 2H), 1.54 – 1.45 (m, 3H), 1.42 (s, 9H), 1.35 (s, 3H). **^{13}C NMR** (101 MHz, $CDCl_3$) δ 152.6, 151.7, 139.0, 133.9, 129.5, 128.2, 94.4, 93.9, 80.7, 80.4, 67.1, 55.9, 55.6, 53.4, 53.2, 28.4, 27.7, 27.2, 27.1, 26.7, 24.3, 22.9.

m.p.: 56-58 °C.

HRMS (ESI) m/z calcd for $C_{18}H_{27}NO_5SNa^+$: $[M+Na]^+$ 392.1502; found: 392.1499.

***tert*-Butyl 2,2-dimethyl-4-(2-(pyridin-2-yl)ethyl)oxazolidine-3-carboxylate (34).**

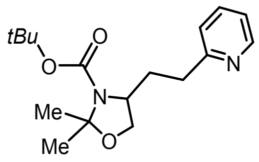

Freshly distilled **2a** was used. The crude was purified by flash chromatography on silica (CH_2Cl_2/MeOH 98:2) to afford the product as a colourless oil (45.9 mg, 75% after isolation).

Mixture of rotamers: **^{1}H NMR** (400 MHz, $CDCl_3$) δ 8.48 (d, *J* = 5 Hz, 1H), 7.56 (t, *J* = 8 Hz, 1H), 7.20 – 7.01 (m, 2H), 4.00 – 3.73 (m, 3H), 2.84 – 2.66 (m, 2H), 2.46 – 1.86 (m, 2H), 1.60 – 1.50 (m, 3H), 1.49 – 1.41 (m, 9H), 1.38 (s, 3H). **^{13}C NMR** (101 MHz, $CDCl_3$) δ 161.4, 152.4, 151.9, 149.4, 149.3, 136.5, 122.8, 121.2, 93.8, 93.4, 80.1, 79.6, 66.9, 66.8, 57.5, 56.9, 35.2, 33.7, 32.9, 28.6, 27.6, 26.9, 24.7, 23.3.

HRMS (ESI) m/z calcd for $C_{17}H_{27}N_2O_3^+$: $[M+H]^+$ 307.2016; found: 307.2011.

***tert*-Butyl 2,2-dimethyl-4-(phenylethynyl)oxazolidine-3-carboxylate (35).**

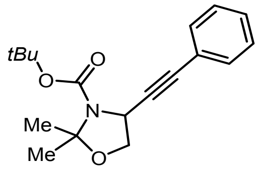

The crude was purified by flash chromatography on silica (Hexane/Ethyl Acetate 9:1) to afford the product as a white solid (24.7 mg, 41% after isolation). Spectroscopic data are in accordance with the literature.[25]

^{1}H NMR (400 MHz, $CDCl_3$) δ 7.44 – 7.37 (m, 2H), 7.34 – 7.27 (m, 3H), 4.89 – 4.68 (m, 1H), 4.16 – 4.06 (m, 2H), 1.67 (s, 3H), 1.54 (s, 3H), 1.51 (s, 9H). **^{13}C NMR** (101 MHz, $CDCl_3$) δ 151.8, 131.9, 131.8, 128.4, 123.0, 94.6, 88.3, 82.2, 80.4, 69.0, 49.3, 28.6, 26.1, 24.7.

m.p.: 102-104 °C

HRMS (ESI) m/z calcd for $C_{18}H_{23}NO_3Na^+$: $[M+Na]^+$ 324.1570; found: 324.1569.

***tert*-Butyl 2,2-dimethyl-4-(*p*-tolylethynyl)oxazolidine-3-carboxylate (36).**

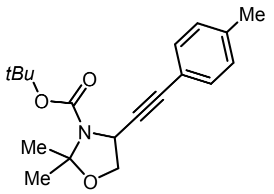

The crude was purified by flash chromatography on silica (Hexane/MTBE 9:1) to afford the product as a pale yellow oil (31.8 mg, 51% after isolation).

^{1}H NMR (400 MHz, $CDCl_3$) δ 7.30 (d, *J* = 8 Hz, 2H), 7.10 (d, *J* = 8 Hz, 2H), 4.90 – 4.67 (m, 1H), 4.14 – 4.05 (m, 2H), 2.34 (s, 3H), 1.67 (s, 3H), 1.54 (s, 3H), 1.50 (s, 9H). **^{13}C NMR** (101 MHz, $CDCl_3$) δ 151.8, 138.6, 138.5, 131.9, 131.7, 129.1, 120.0, 94.6, 94.5, 87.5, 86.7, 82.4, 80.3, 69.1, 49.3, 28.6, 28.4, 26.0, 24.7, 21.6.

HRMS (ESI) m/z calcd for $C_{19}H_{25}NO_3Na^+$: $[M+Na]^+$ 338.1727; found: 338.1728.

***tert*-Butyl 4-((4-(*tert*-butyl)phenyl)ethynyl)-2,2-dimethyloxazolidine-3-carboxylate (37).**

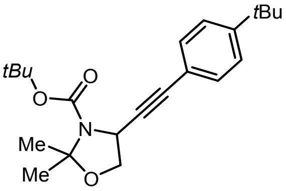

The crude was purified by flash chromatography on silica (PET/Ethyl Acetate 9:1) to afford the product as a white solid (28.4 mg, 40% after isolation).

Mixture of rotamers: **^{1}H NMR (**400 MHz, $CDCl_3$) δ 7.33 (q, *J* = 9 Hz, 4H), 4.90 – 4.65 (m, 1H), 4.17 – 4.02 (m, 2H), 1.67 (s, 3H), 1.54 (s, 3H), 1.51 (s, 9H), 1.30 (s, 9H). **^{13}C NMR** (101 MHz, $CDCl_3$) δ 151.8, 151.6, 131.5, 125.4, 120.0, 94.5, 87.6, 82.3, 80.3, 69.1, 49.3, 34.9, 31.3, 31.1, 28.6, 28.4, 26.0, 24.7.

m.p.: 94-96 °C.

HRMS (ESI) m/z calcd for $C_{22}H_{31}NO_3Na^+$: $[M+Na]^+$ 380.2196; found: 380.2195.

tert-Butyl 4-((1,1'-biphenyl)-4-ylethynyl)-2,2-dimethyloxazolidine-3-carboxylate (38).

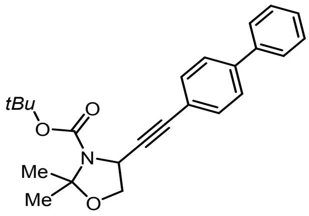

The crude was purified by flash chromatography on silica (Hexane/Ethyl Acetate 9:1) to afford the product as a white solid (39.3 mg, 52% after isolation).

^{1}H NMR (400 MHz, $CDCl_3$) δ 7.62 – 7.52 (m, 4H), 7.51 – 7.40 (m, 4H), 7.35 (t, *J* = 7 Hz, 1H), 4.95 – 4.71 (m, 1H), 4.12 (d, *J* = 3 Hz, 2H), 1.69 (s, 3H), 1.63 – 1.54 (m, 3H), 1.52 (s, 9H). **^{13}C NMR** (101 MHz, $CDCl_3$) δ 151.8, 141.2, 140.5, 132.2, 129.0, 127.8, 127.2, 121.9, 94.6, 88.9, 82.1, 80.4, 69.1, 49.3, 29.8, 28.6, 26.1, 24.7.

m.p.: 102-104 °C.

HRMS (ESI) m/z calcd for $C_{24}H_{27}NO_3Na^+$: $[M+Na]^+$ 400.1883; found: 400.1883.

tert-Butyl 2,2-dimethyl-4-((4-(trifluoromethyl)phenyl)ethynyl)oxazolidine-3-carboxylate (39).

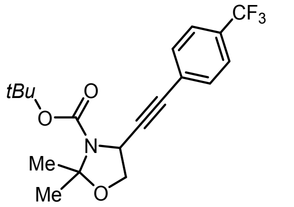

The crude was purified by flash chromatography on silica (Hexane/MTBE 9:1) to afford the product as a pale yellow oil (30 mg, 41% after isolation).

^{1}H NMR (400 MHz, $CDCl_3$) δ 7.60 – 7.47 (m, 4H), 4.93 – 4.66 (m, 1H), 4.18 – 4.05 (m, 2H), 1.67 (s, 3H), 1.50 (s, 9H), 1.25 (s, 3H).

^{13}C NMR (101 MHz, $CDCl_3$) δ 151.6, 132.0 (m), 130.1 (q, *J* = 36 Hz), 128.4, 125.7, 125.4 (bs), 124.9, 124.0 (q, *J* = 270 Hz), 94.7, 94.2, 90.9, 90.8, 81.0, 80.5, 68.8, 49.2, 32.1, 29.8, 29.5, 28.6, 27.2, 26.1, 25.3, 24.6, 22.8. **^{19}F NMR** (565 MHz, $CDCl_3$) δ -62.76.

HRMS (ESI) m/z calcd for $C_{19}H_{22}F_3NO_3Na^+$: $[M+Na]^+$ 392.1444; found: 392.1444.

tert-Butyl 4-((4-bromophenyl)ethynyl)-2,2-dimethyloxazolidine-3-carboxylate (40).

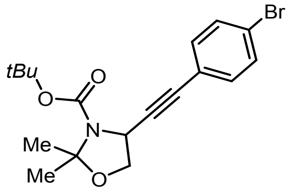

The crude was purified by flash chromatography on silica (Hexane/MTBE 9:1) to afford the product as a pale yellow oil (35 mg, 46% after isolation).

^{1}H NMR (400 MHz, $CDCl_3$) δ 7.43 (d, *J* = 8 Hz, 2H), 7.26 (d, *J* = 8 Hz, 2H), 4.88 – 4.66 (m, 1H), 4.14 – 4.06 (m, 2H), 1.65 (s, 3H), 1.53 (s, 3H), 1.50 (s, 9H). **^{13}C NMR** (151 MHz, $CDCl_3$) δ 151.6, 133.4, 133.2, 131.7, 122.6, 122.0, 94.6, 89.5, 81.0, 80.4, 68.9, 49.2, 28.6, 27.3, 26.1, 25.3, 24.6. **HRMS** (ESI) m/z calcd for $C_{18}H_{22}BrNO_3Na^+$: $[M+Na]^+$ 402.0675; found: 402.0673.

tert-Butyl 4-((4-methoxyphenyl)ethynyl)-2,2-dimethyloxazolidine-3-carboxylate (41).

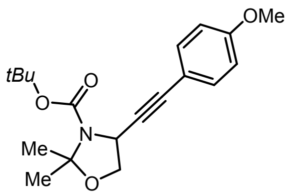

The crude was purified by flash chromatography on silica (Hexane/MTBE 9:1) to afford the product as a white solid (66.3 mg, 53% after isolation).

^{1}H NMR (400 MHz, $CDCl_3$) δ 7.34 (d, *J* = 8 Hz, 2H), 6.82 (d, *J* = 8 Hz, 2H), 4.89 – 4.65 (m, 1H), 4.16-4.02 (m, 2H), 3.79 (s, 3H), 1.66 (s, 3H), 1.53 (s, 3H), 1.50 (s, 9H). **^{13}C NMR** (101 MHz, $CDCl_3$) δ 159.7, 151.8, 133.2, 115.1, 114.0, 94.5, 86.8, 82.1, 80.3, 69.1, 55.4, 49.3, 28.6, 26.0, 24.7.

m.p.: 72-74 °C.

HRMS (ESI) m/z calcd for $C_{19}H_{25}NO_4Na^+$: $[M+Na]^+$ 354.1676; found: 354.1673.

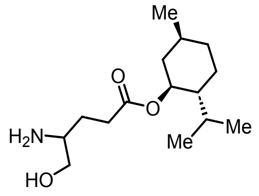

(1R,2S,5S)-5-Isopropyl-2-methylcyclohexyl 4-amino-5-hydroxypentanoate (42).

Mixture of diastereomers (dr 1:1 via NMR): **^{1}H NMR** (400 MHz, CD_3OD) δ 4.70 (td, *J* = 11, 4 Hz, 1H), 3.76 (dd, *J* = 12, 4 Hz, 1H), 3.58 (dd, *J* = 12, 6 Hz, 1H), 3.29 – 3.23 (m, 1H), 2.49 (t, *J* = 8 Hz, 2H), 1.98 – 1.82 (m, 4H), 1.75-1.64 (m, 2H), 1.54 – 1.34 (m, 2H), 1.16 – 0.96 (m, 2H), 0.91 (t, *J* = 7 Hz, 7H), 0.76 (d, *J* = 7 Hz, 3H). **^{13}C NMR** (101 MHz, CD_3OD) δ 173.7, 173.7, 75.8, 61.7, 53.8, 53.8, 48.3, 41.9, 35.3, 32.6, 31.1, 31.1, 27.4, 27.4, 25.6, 24.4, 24.4, 22.4, 21.1, 21.0, 16.6, 16.6.

HRMS (ESI) m/z calcd for $C_{15}H_{30}NO_3^+$: $[M+H]^+$ 272.2220; found: 272.2217.

tert-Butyl 5-(3-ethoxy-3-oxopropyl)-2,2-dimethyloxazolidine-3-carboxylate (43).

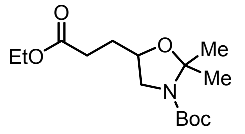

The crude was purified by flash chromatography on silica (Hexane/EtOAc 9:1) to afford the product as a pale yellow oil (34.4 mg, 52% after isolation). **^{1}H NMR (400 MHz, $CDCl_3$)** δ 4.13 (q, *J* = 7.1 Hz, 2H), 4.11 – 4.01 (m, 1H), 3.77 – 3.54 (m, 1H), 3.15 – 2.98 (m, 1H), 2.51 – 2.30 (m, 2H), 2.01 – 1.82 (m, 2H), 1.58 – 1.49 (m, 3H), 1.47 (s, 12H), 1.25 (t, *J* = 7.1 Hz, 3H). **^{13}C NMR (101 MHz, $CDCl_3$)** δ 173.2, 152.0, 93.7, 93.2, 80.2, 79.6, 72.9, 72.7, 60.6, 50.7, 30.4, 29.8, 28.4, 27.4, 26.4, 25.4, 24.5, 14.4.

HRMS (ESI) m/z calcd for $C_{15}H_{27}NO_5Na^+$: $[M+Na]^+$ 324.1781; found: 324.1781.

9. Mechanistic investigations

9.1. Control experiments

*Experiments C1: Giese reaction with **S7** as H-donor*

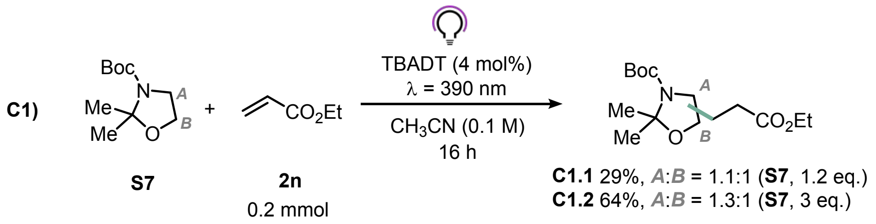

Two experiments were conducted: C1.1 and C1.2.

In C1.1, **S7** (1.2 equiv., 0.24 mmol, 48.3 mg) as H-donor was reacted with freshly distilled ethyl acrylate (**2n**, 1 equiv., 0.2 mmol, 22 µL) as radical trap according to **GP3**. Purification by flash chromatography on silica (Hexane/Ethyl Acetate 8:2) afforded the Giese adduct as a mixture of regioisomers (**24**+**43**, 29% combined yield, rr 1.1:1 via GCMS, see Figure S3) as a colourless oil. Regioisomer A (**24**), Calculated Mass = 301.2; Experimental mass = 301.1; Elution time = 10.4; regioisomer B (**43**), Calculated mass = 301.2; Experimental mass = 301.1; Elution time = 10.7).

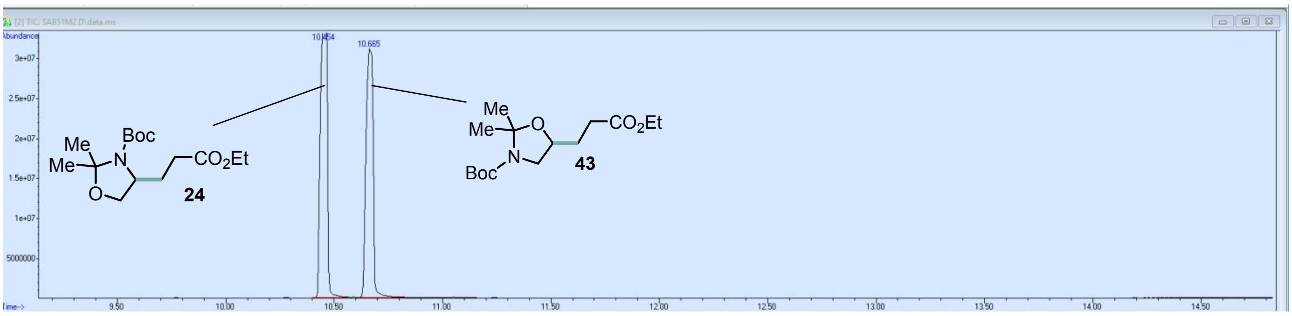

Figure S3: GC-MS of the two regioisomers obtained in control experiment C1.1. GC-MS analyses were performed on an Agilent GCMS 5977B instrument. The injection was performed at 280 °C in split mode (20:1). The initial oven temperature of 60 °C was maintained for 3 min, increased by 20 °C/min to 280 °C and held for 18 min. An Agilent HP5 30 m × 0.25 mm × 0.25 µm film thickness capillary column was used with helium as the carrier gas at a constant flow rate of 1.0 mL·min^{-1}

In C1.2, the same procedure of experiment **C1.1** was followed, but using 3 equiv. of **S7** (0.6 mmol, 120.7 mg). In this case, the two regioisomers (compounds **24** and **43**) were obtained in a 1.3:1 rr as a colourless oil (40 mg, 64% yield combined).

Overall, these experiments showed low regioselectivity (if none) in the Giese reaction in absence of the TAG on the H-donor.

*Experiment C2: SOMOphilic with **S7** as H-donor*

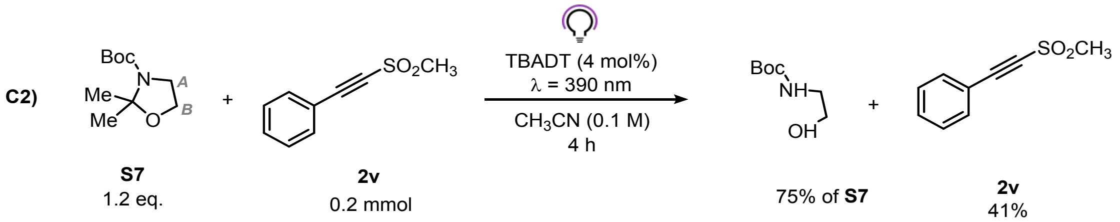

In this experiment, **S7** (1.2 equiv., 0.24 mmol, 48.3 mg) as H-donor was reacted with ((methylsulfonyl)ethynyl)benzene (**2v**, 1 equiv., 0.2 mmol, 36 mg) as radical trap according to general procedure **GP4**. The reaction crude was suspended in 600 μL of $CDCl_3$, CH_2Br_2 (0.2 mmol, 14 μL) was added and the mixture was sonicated and filtered through a short cotton plug in a Pasteur pipette. The filtrate was analyzed via ^{1}H-NMR. We found unconverted **2v** (41%) and significant amounts of *N*-Boc-ethanolamine (75% respect to initial **S7**), deriving from the opening of oxazolidine **S7**.

Experiment C2 shows that the HAcTive strategy has the potential to enable transformations that would otherwise lead to major decomposition of the starting materials.

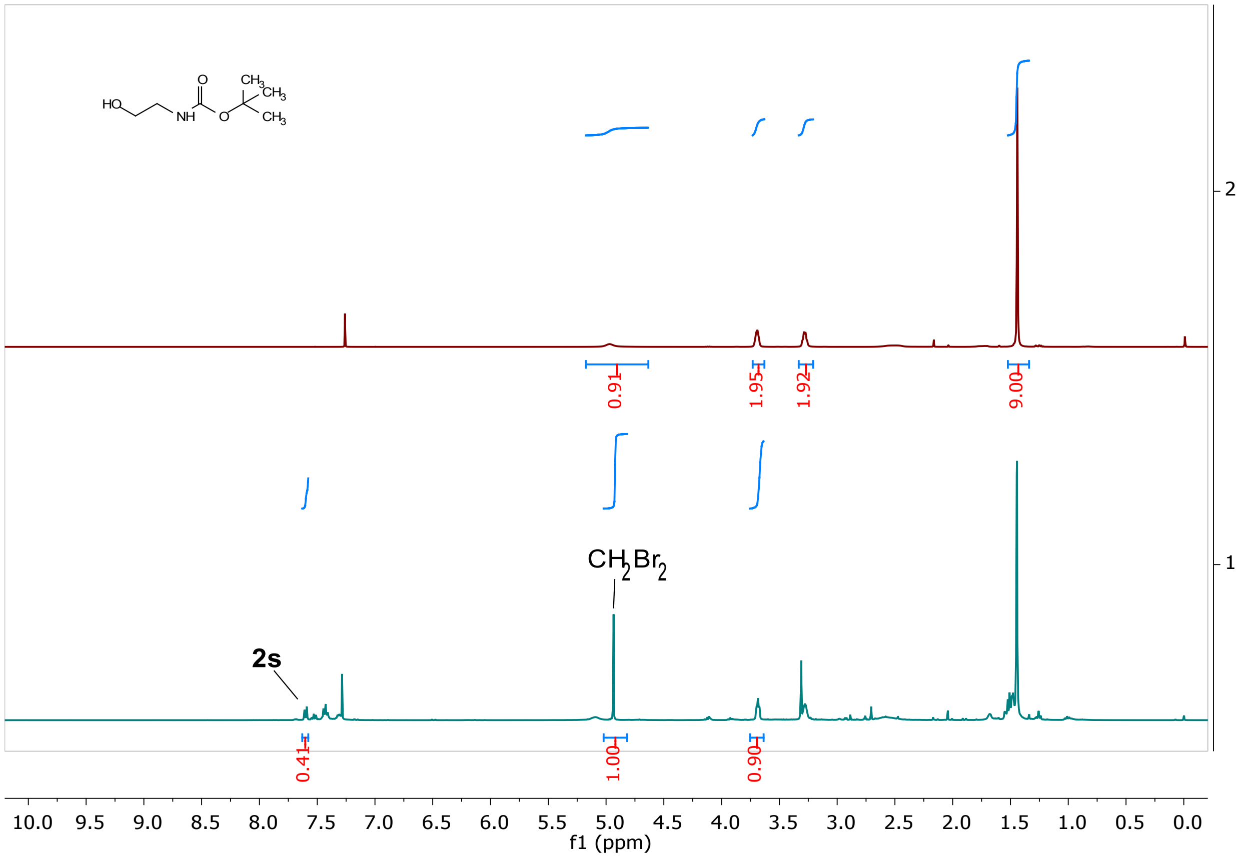

Figure S4: ^{1}H-NMR of the crude of experiment C2. ^{1}H-NMR of pure N-Boc-ethanolamine is shown for a comparison. Both spectra were taken in $CDCl_3$.

9.2. Laser-Flash Photolysis

Instrumentation. Laser flash photolysis studies were performed by adopting the third harmonic of a Q-switched Nd:YAG laser (λ_{EXC} = 355 nm). The sample solution was placed in a 1x1 cm quartz cell and excited with single pulses (5-7 mJ) delivered from the laser and analyzed with a pulsed Xe arc lamp. Lifetimes of the reactive transient ***wO*** were obtained by fitting the first order decay profiles recorded at 780 nm by using the following equation:

$$y = y_0 + A \cdot e^{-\frac{x}{\tau}}$$

Thus, 3 mL of an air-equilibrated $2.0 \cdot 10^{-4}$ M CH_3CN solution of TBADT $(n\mathrm{Bu}_4)_4[W_{10}O_{32}]$ were placed in a cuvette. This solution had an absorbance value of ~0.6-0.8 at 355 nm. The lifetime of TBADT was monitored in the presence of different concentrations of the quencher and Stern-Volmer plots were obtained from the following equation:

$$\frac{\tau_0}{\tau} = 1 + k_Q \tau_0 [Q]$$

Summary

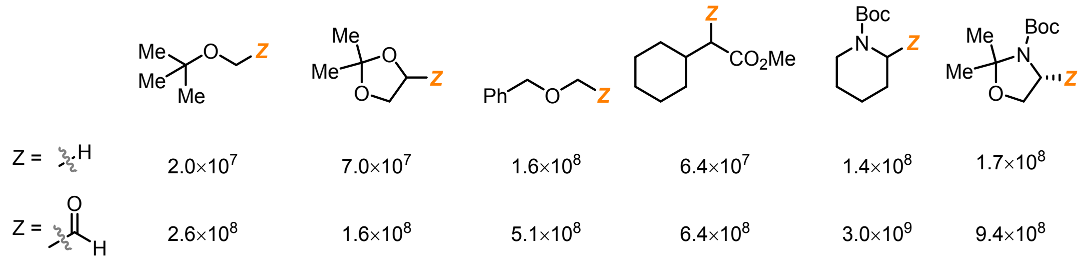

*Methyl tert-butyl ether (**S1**)*

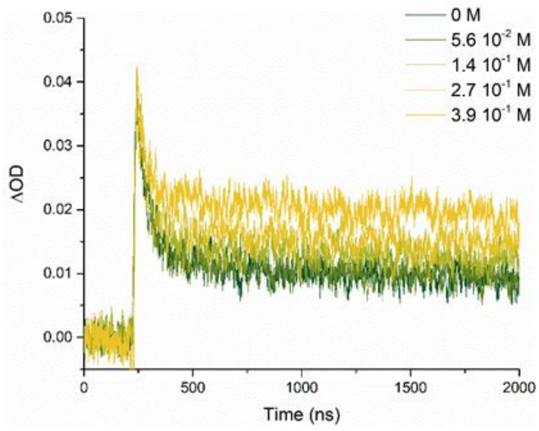

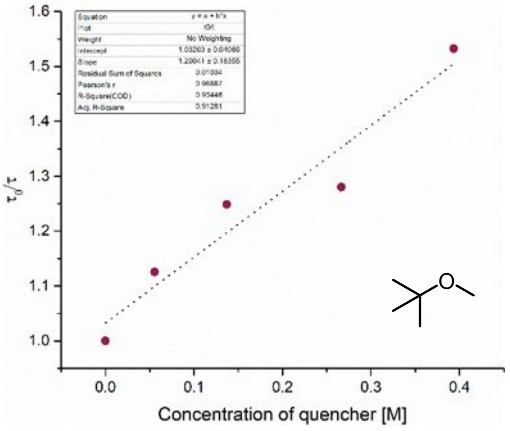

$\tau_0 = 59 \times 10^{-9}$ s $\quad$ k_Q(**S1**) = $2.0 \times 10^7\ M^{-1} \cdot s^{-1}$

*tert-Butoxy acetaldehyde (**1a**)*

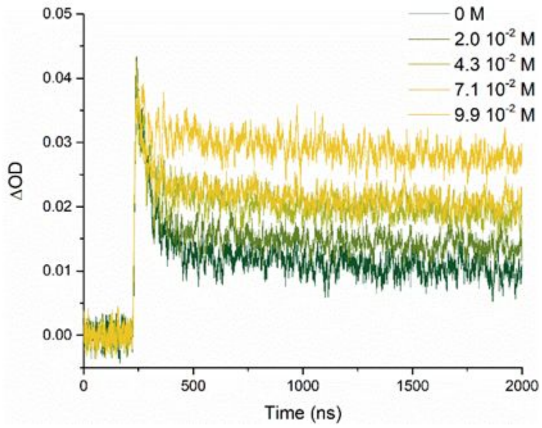

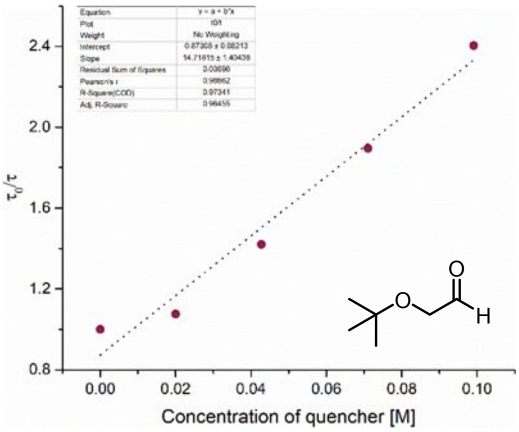

τ_0 = 56×10^{-9} s k_Q(**1a**) = 2.6×10^8 M^{-1}·s^{-1}

*2,2-Dimethyl-1,3-dioxolane (**S2**)*

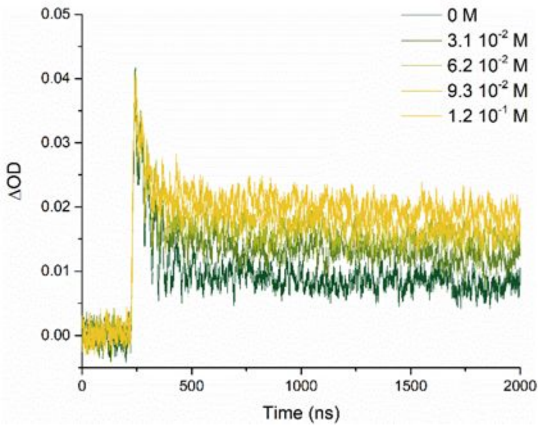

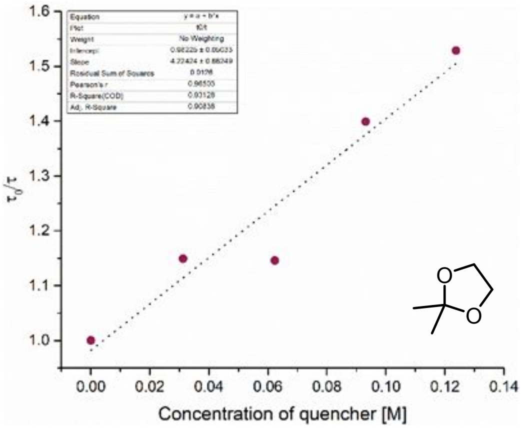

τ_0 =60×10^{-9} s k_Q(**S2**) =7.0×10^7 M^{-1}·s^{-1}

*2,2-Dimethyl-1,3-dioxolane-4-carbaldehyde (**1b**)*

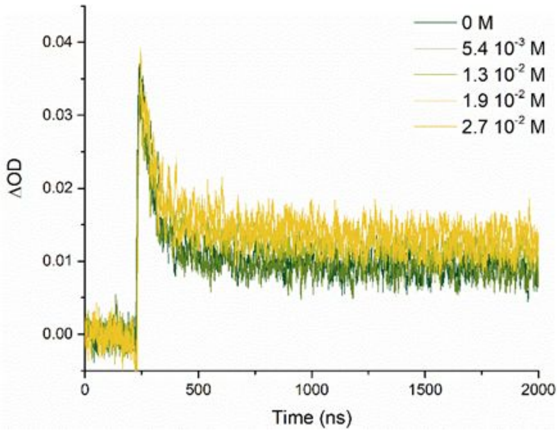

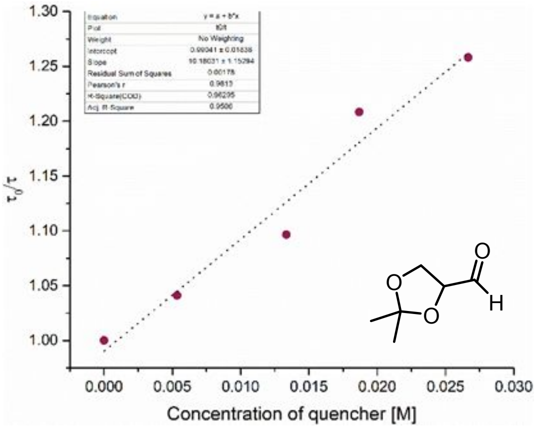

τ_0 = 62×10^{-9} s k_Q(**1b**) = 1.6×10^8 M^{-1}·s^{-1}

*Benzyl methyl ether (**S3**)*

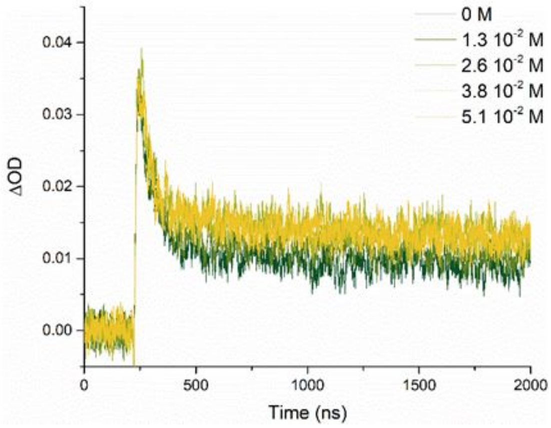

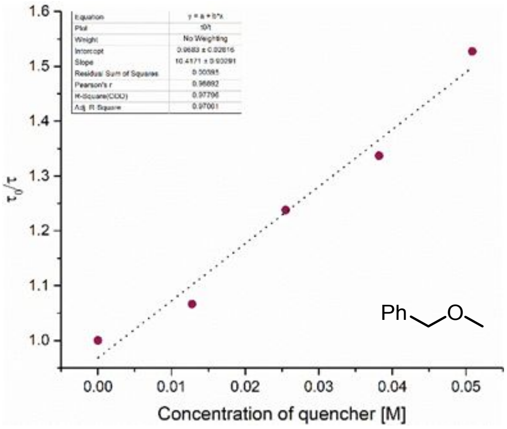

τ_0 =63×10^{-9} s k_Q(**S3**) = 1.6×10^8 M^{-1}·s^{-1}

*Benzyloxy acetaldehyde (**1c**)*

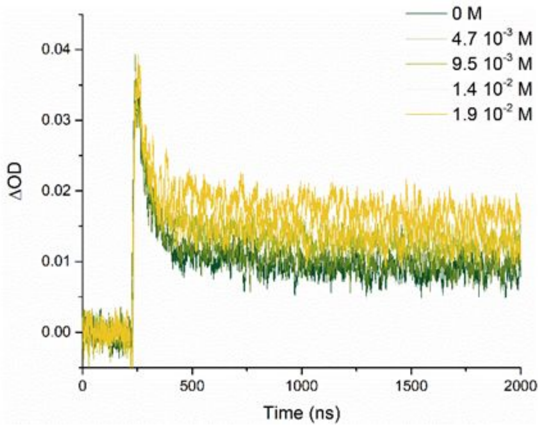

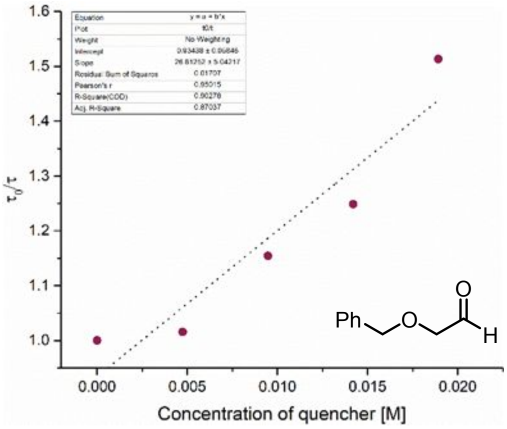

τ_0 = 52×10^{-9} s k_Q(**1c**) = 5.1×10^8 M^{-1}·s^{-1}

*methyl 2-cyclohexylacetate (**S5**)*

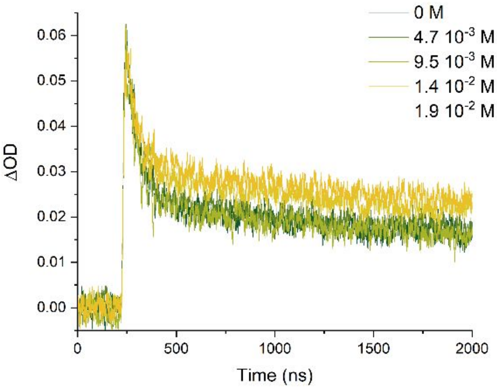

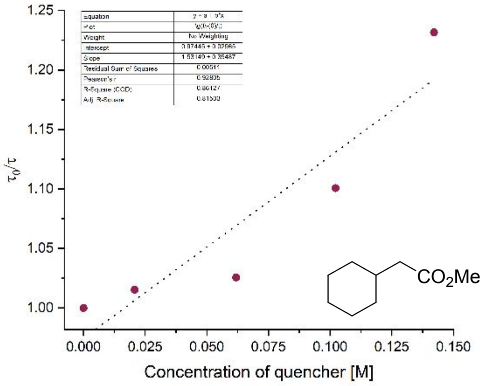

τ_0 =60×10^{-9} s k_Q(**S5**) = 2.6×10^7 M^{-1}·s^{-1}

*Methyl 2-cyclohexyl-3-oxopropanoate (**1e**)*

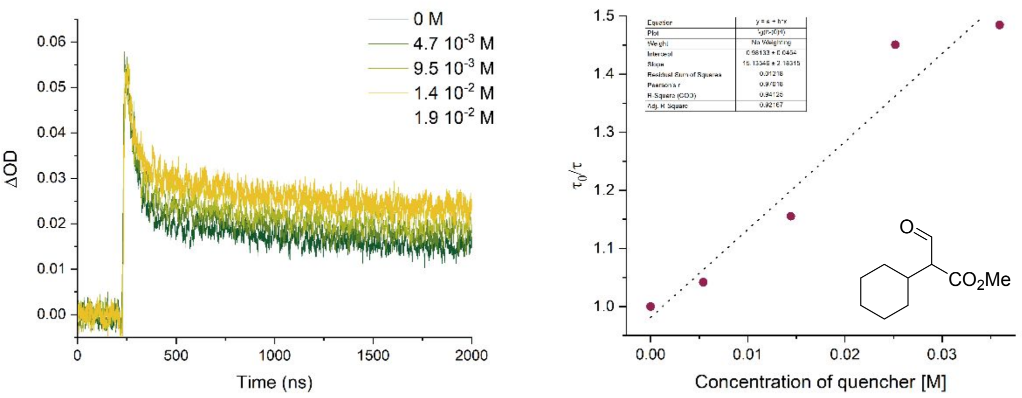

τ_0 =57×10^{-9} s k_Q(**1e**) = 2.6×10^8 M^{-1}·s^{-1}

*N-Boc-2-Piperidine (**S6**)*

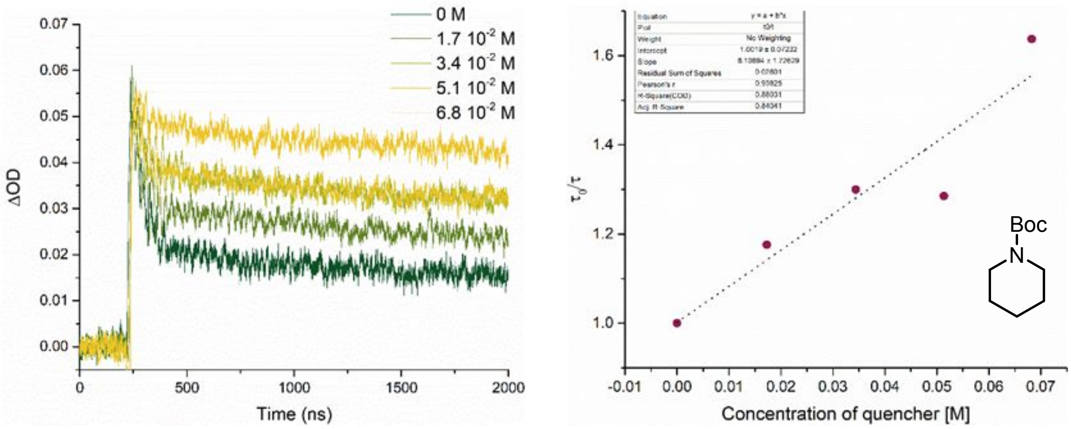

τ_0 =57×10^{-9} s k_Q(**S6**) = 1.4×10^8 M^{-1}·s^{-1}

*N-Boc-2-Piperidinecarbaldehyde (**1f**)*

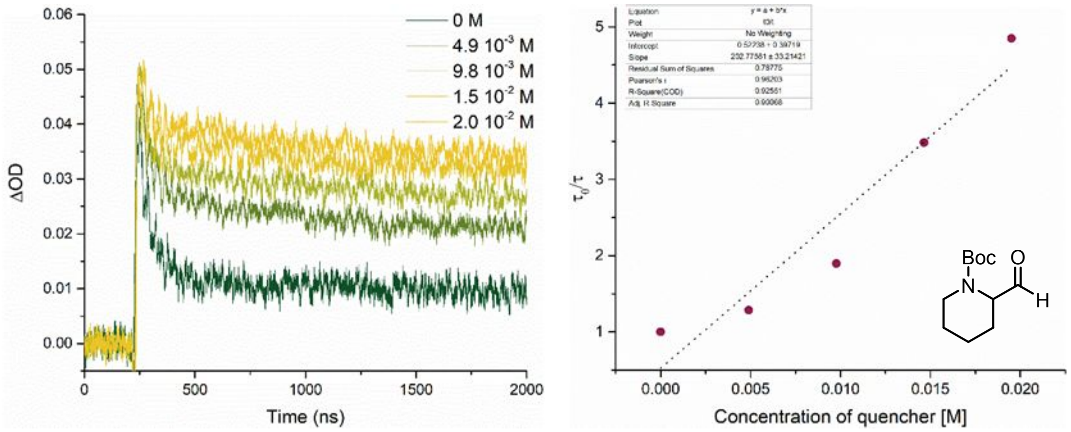

τ_0 =68×10^{-9} s k_Q(**1f**) = 3.0×10^9 M^{-1}·s^{-1}

*tert-Butyl 5-formyl-2,2-dimethyloxazolidine-3-carboxylate (**S7**)*

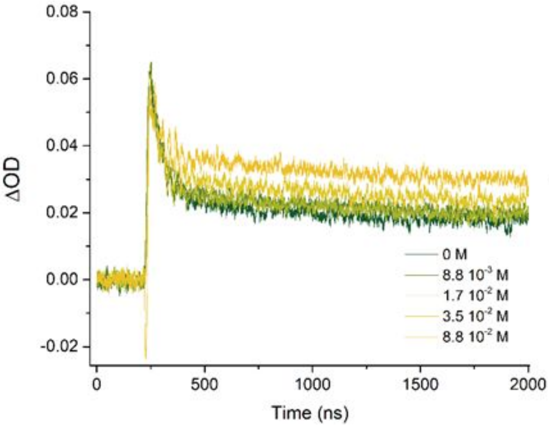

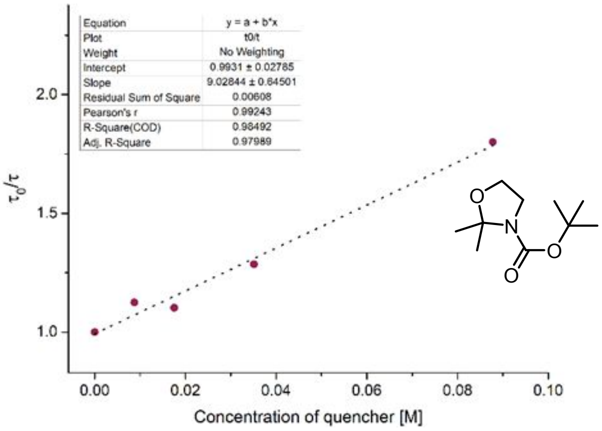

τ_0 = 54×10^{-9} s k_Q(**S7**) = 1.7×10^8 M^{-1}·s^{-1}

*Garner's aldehyde (**1h**)*

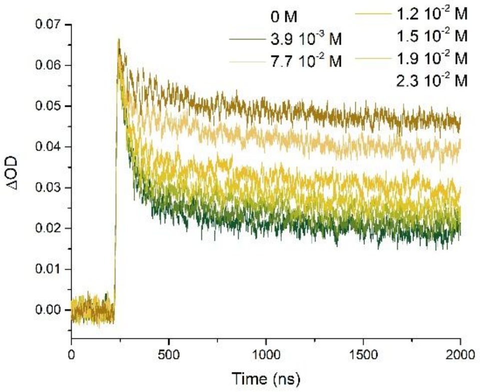

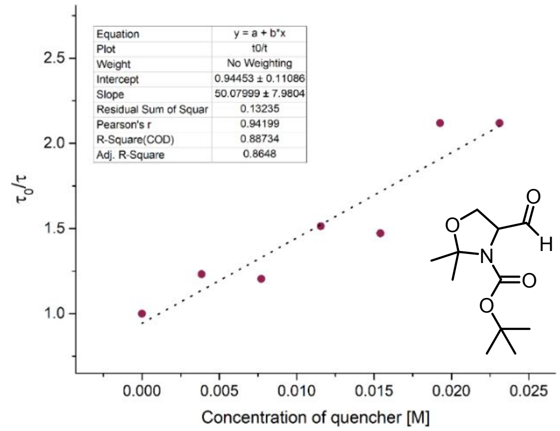

τ_0 = 53×10^{-9} s k_Q(**1h**) = 9.4×10^8 M^{-1}·s^{-1}

9.3. DFT analysis

All the calculations were carried out using the Gaussian 16 program package, revision C.01,[26] installed on Galileo100 at CINECA facility (Italy). The level of theory chosen for the optimization of the reported stationary points was DFT (Density Functional Theory) by using the ωB97xD functional and the def2TZVP basis set in the gas phase. When appropriate, an unrestricted formalism (U prefix) has been adopted via the UωB97xD/def2TZVP keyword. No symmetry constraint was applied to the structures investigated and a thorough conformers search has been performed to locate the absolute minimum for each species. The structures and data used for this work correspond to those of the located absolute minimum. Frequency calculations were performed in the gas phase to check that minima and transition states (TS) had 0 or 1 imaginary frequencies, respectively.

Solvent effect was included by single-point calculations at the same level of theory (ωB97xD/def2TZVP) adopting the standard implicit solvent model implemented in Gaussian 16 via the keyword SCRF=(SOLVENT=ACETONITRILE) on the optimized geometries obtained in vacuo. The DFT Gibbs free energies reported in the main text have been calculated by means of Eq. S1 reported below:

$$G_{DFT} = E_{0(DFT,MeCN)} + \Delta G_{CORR(vacuo)} \qquad (S1)$$

Where:

- $E_{0(DFT,MeCN)}$ is the total electronic energy calculated at the SCRF-ωB97xD/def2TZVP level (acetonitrile bulk);
- $\Delta G_{CORR(vacuo)}$ is the unscaled thermal correction to Gibbs Free Energy as from the output of the frequency calculation in vacuo, also including the zero-point vibrational energy (ZPVE).

The two terms from Eq. S1 have been reported in blue color for all the stationary points reported below.

As for TSs, Intrinsic Reaction Coordinate (IRC) calculations were performed in both directions (40 steps each) at the same level of theory adopted for optimizations (ωB97xD/def2TZVP in the gas phase) in order to investigate the process in detail and to confirm the nature of the TS itself. The "LQA" option for the IRC keyword has been consistently specified, in order to adopt the local quadratic approximation for the predictor step.

The modeling of the decarbonylation processes has considered the separated species, namely the alkyl radical and carbon monoxide (CO), as separated products. Accordingly, the sum of the corresponding Gibbs free energies (G_{DFT}, as defined in equation S1) has been considered to calculate the overall ΔG of the process. On the other hand, the IRC profiles observed in Figure S6 (see below) describe the stepwise removal of CO from the first-formed acyl radical in the gas phase, showing a different energetic profile.

Optimized geometry listed in cartesian format (coordinates are given in Å), minimum energies and thermochemical data (in Hartree; the default options were adopted in the latter case, *viz.* temperature: 298.150 K and pressure: 1.00000 atm) are reported below.

The conversion factor adopted between Hartree and kcal mol^{-1} is: 1 Hartree = 627.509 kcal·mol^{-1}.

Table S6. Modeling of the H abstraction processes

SPECIES	G_{DFT} [Hartree]	SPECIES	G_{DFT} [Hartree]
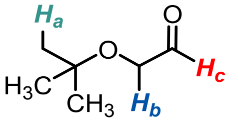 **1a**	-386.192805	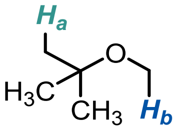 **S1**	-272.868069[a]
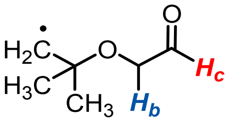 **1a• (*Ha*)**	-385.532544	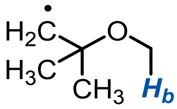 **S1• (*Ha*)**	-272.206907
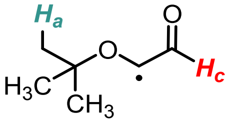 **1a• (*Hb*)**	-385.571231	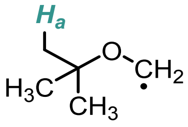 **S1• (*Hb*)**	-272.218400
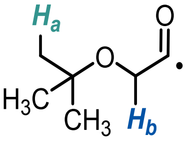 **1a• (*Hc*)**	-385.550248	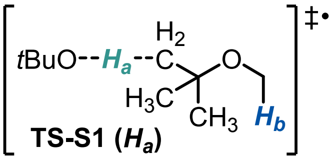 **TS-S1 (*Ha*)**	-505.770332
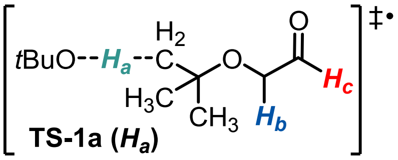 **TS-1a (*Ha*)**	-619.095330	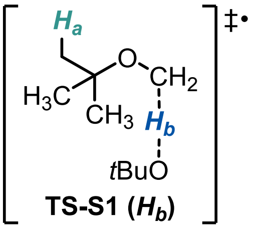 **TS-S1 (*Hb*)**	-505.777690
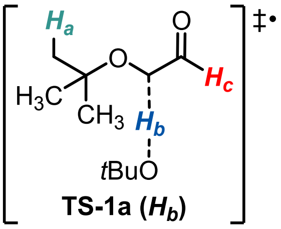 **TS-1a (*Hb*)**	-619.103379	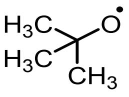 *t*BuO•	-232.932223
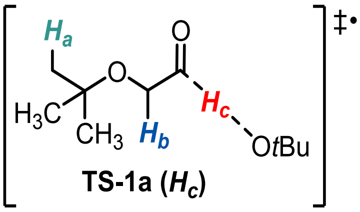 **TS-1a (*Hc*)**	-619.105691	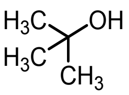 *t*BuOH	-233.595761

[a] The “FREQ=NUMER” option has been adopted for this structure.

Gibbs free energies (shown in Hartree) of the species optimized to model the H abstraction processes adopted in our computational study (see Figure 5B, left panel), as determined from ωB97xD/def2TZVP calculations in bulk acetonitrile.

Table S7. Modeling of the decarbonylation processes.

SPECIES	G_{DFT} [Hartree]	SPECIES	G_{DFT} [Hartree]
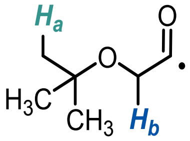<br>**1a• (H_c)**	-385.550248<br>(see Table above)	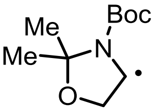<br>**S7•**	-672.131742
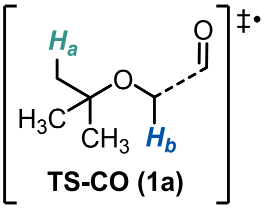<br>**TS-CO (1a)**	-385.535197	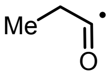<br>**Propanoyl**	-192.466663
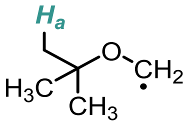<br>**S1• (H_b)**	-272.218400<br>(see Table above)	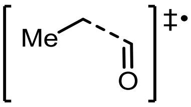<br>**TS-CO (propanoyl)**	-192.441073
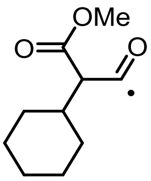<br>**1e•**	-615.587484	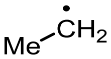<br>**Ethyl**	-79.124357
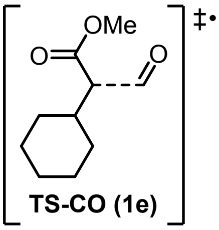<br>**TS-CO (1e)**	-615.572723	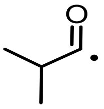<br>**Isobutyryl**	-231.757245
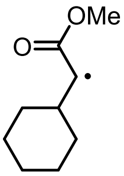<br>**S5•**	-502.264225	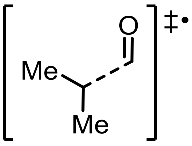<br>**TS-CO (isobutyryl)**	-231.736920
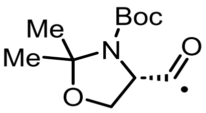<br>**1h•**	-785.459613	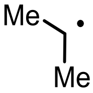<br>**Isopropyl**	-118.420358
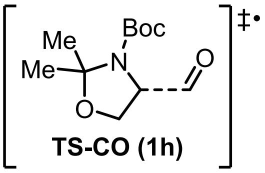<br>**TS-CO (1h)**	-785.448675	CO	-113.336329

Gibbs free energies (shown in Hartree) of the species optimized to model the decarbonylation processes in our computational study, as determined from ωB97xD/def2TZVP calculations in bulk acetonitrile.

9.3.1 IRC Plots

TS-1a˙ (*H*$_a$)

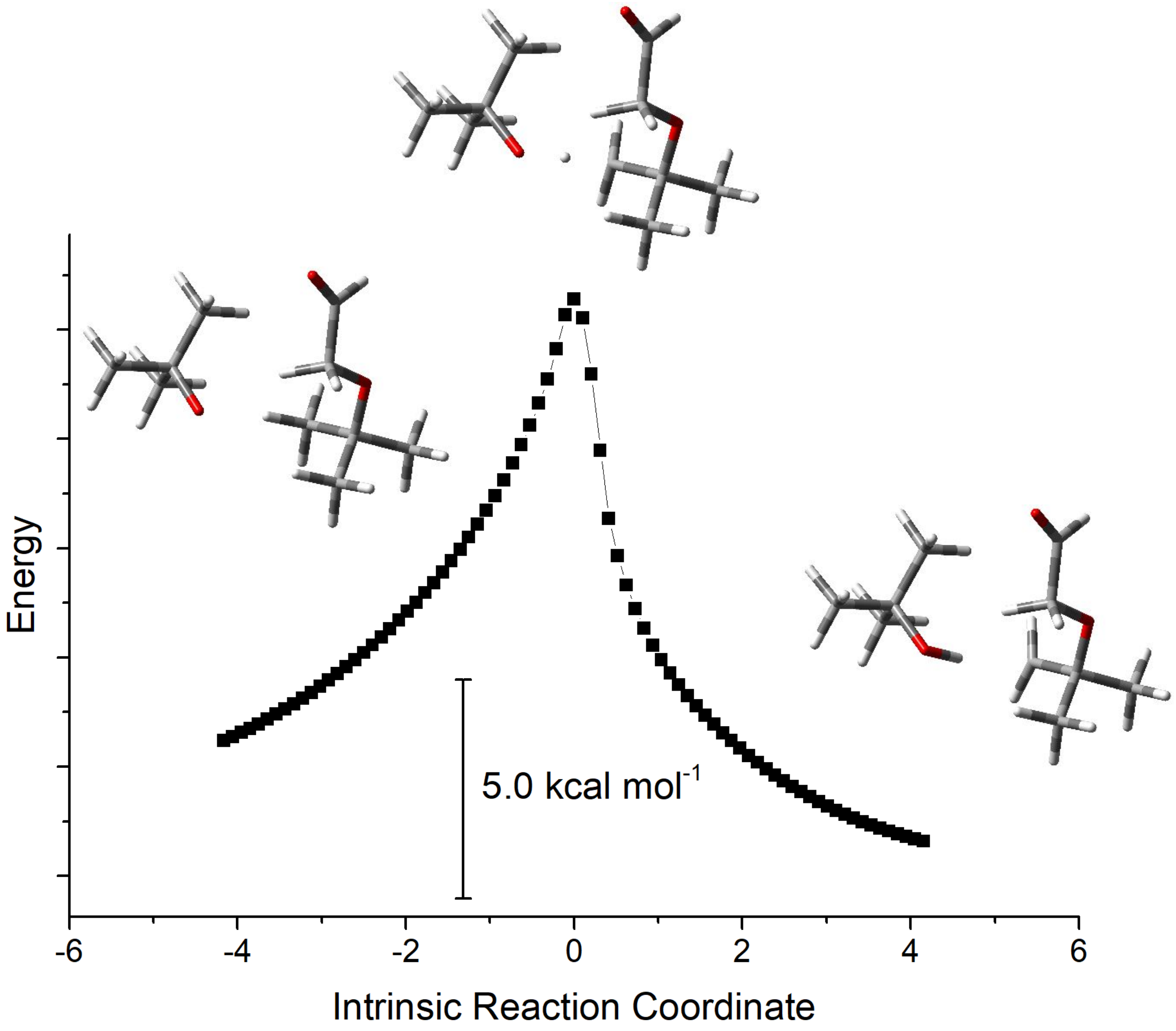

TS-1a˙ (*H*$_b$)

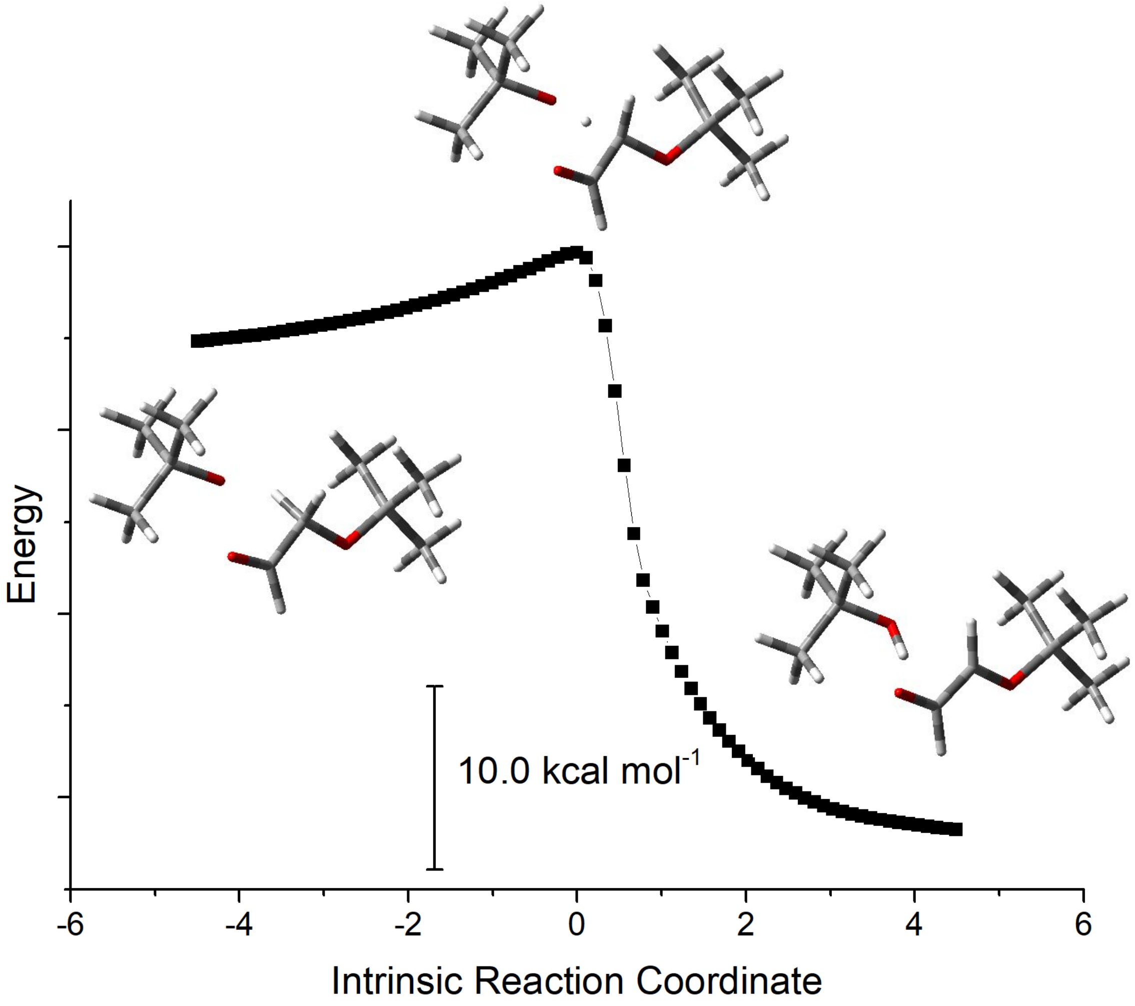
Energy
10.0 kcal mol-1
-6
-4
-2
0
2
4
6
Intrinsic Reaction Coordinate

TS-1a• (H_c)

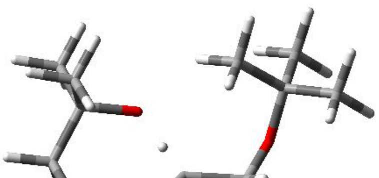

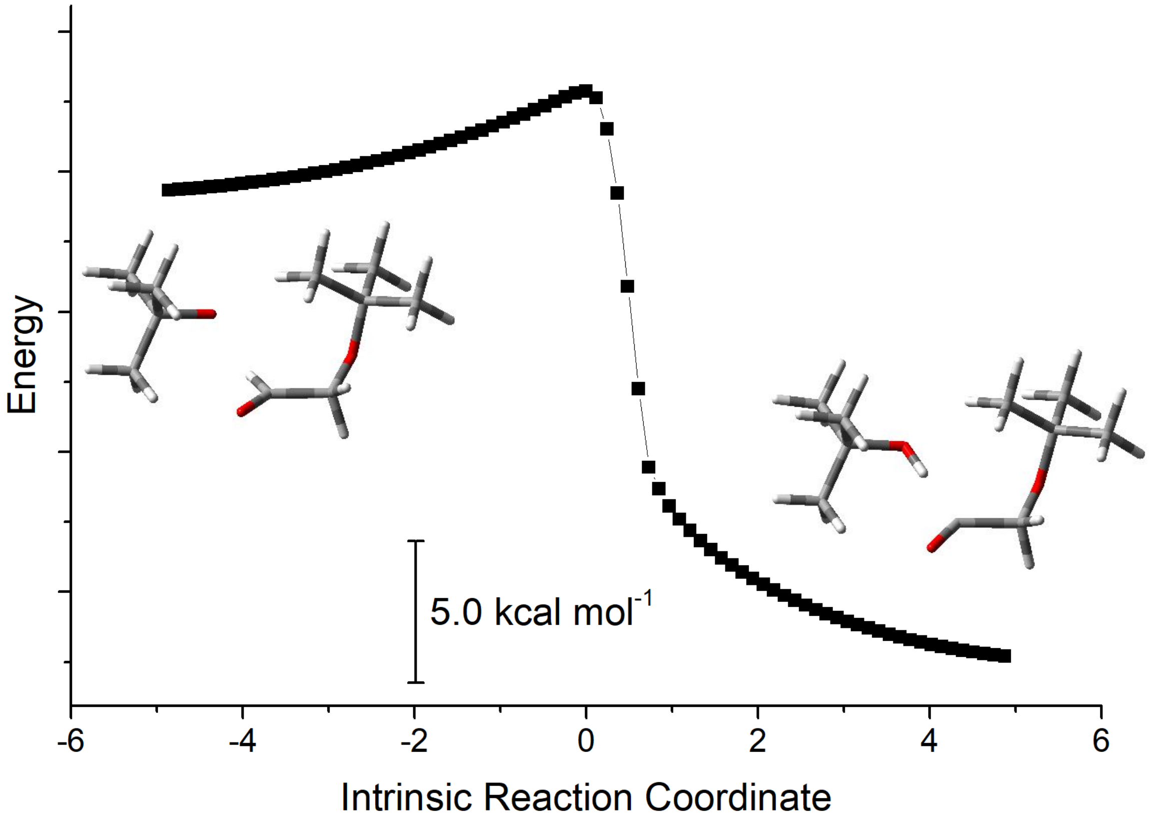

Figure S5: IRC plots of the transition states TS-1a describing the reaction profile described in Figure 5B (left panel), as from calculations at the ωB97xD/def2TZVP level of theory in the gas phase (total electronic energy values have been reported). The three structures reported in each graph refer, respectively, to those of the first point (left), the transition state (center) and the last point (right) along the reaction coordinate.

TS-S1$^{\bullet}$ (H_a)

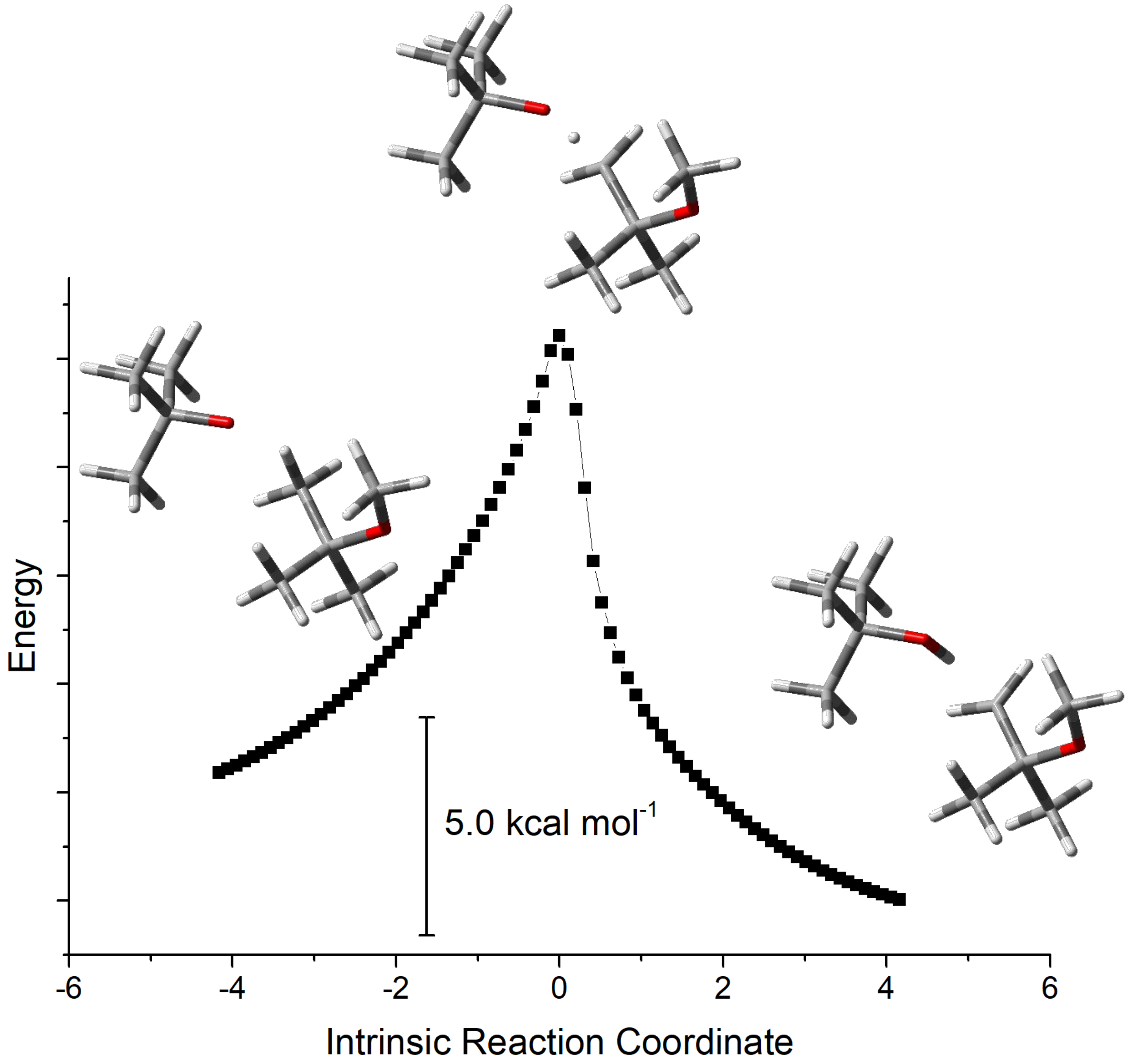

TS-S1$^{\bullet}$ (H_b)

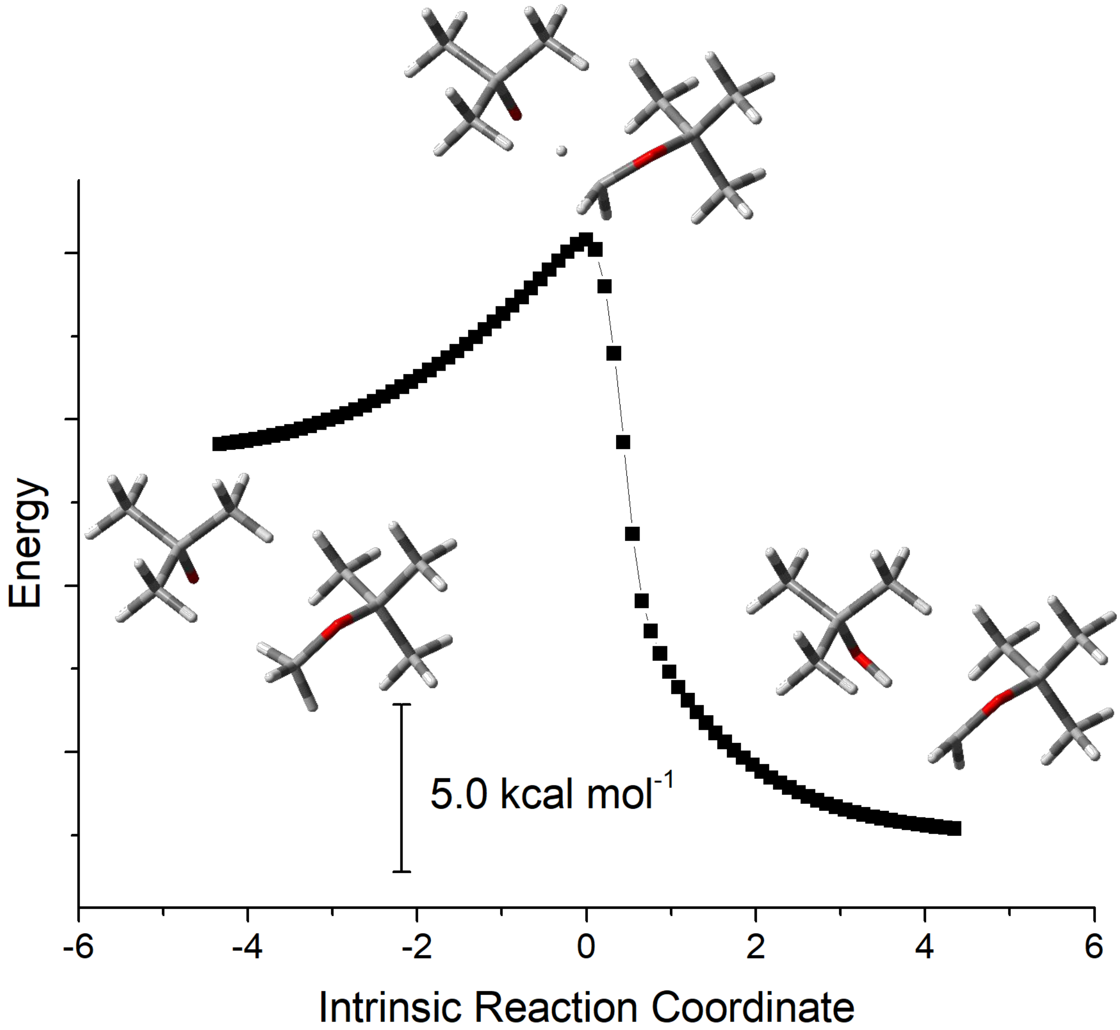

Figure S6: IRC plots of the transition states TS-S1 describing the reaction profile reported in Figure 5B (left panel), as from calculations at the ωB97xD/def2TZVP level of theory in the gas phase (total electronic energy values have been reported). The three structures reported in each graph refer, respectively, to those of the first point (left), the transition state (center) and the last point (right) along the reaction coordinate.

TS-CO (1a)

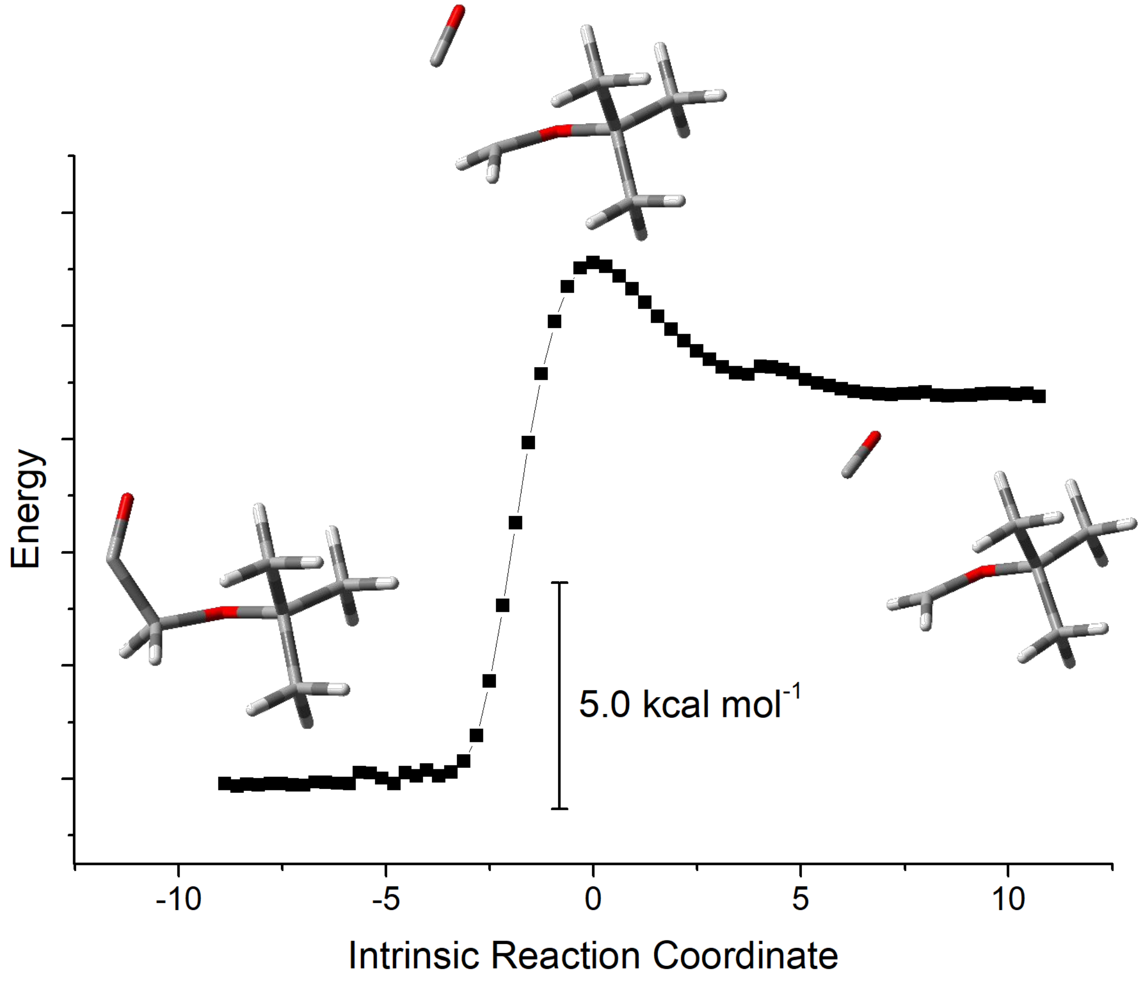

TS-CO (1e)

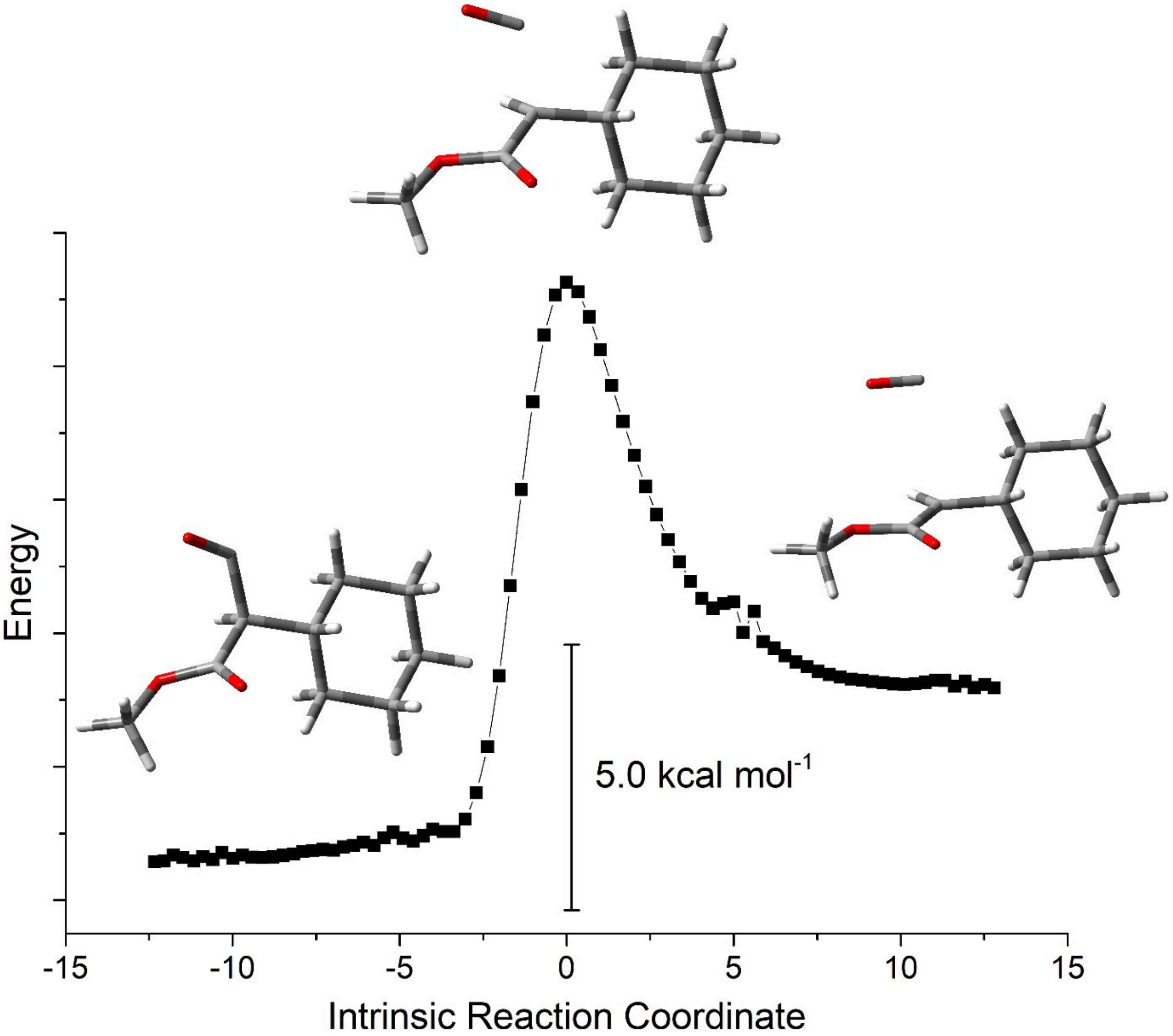

Energy
5.0 kcal mol-1
-15
-10
-5
0
5
10
15
Intrinsic Reaction Coordinate

TS-CO (1h)

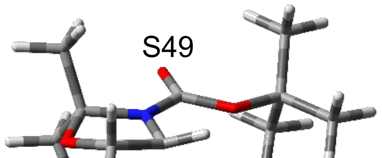

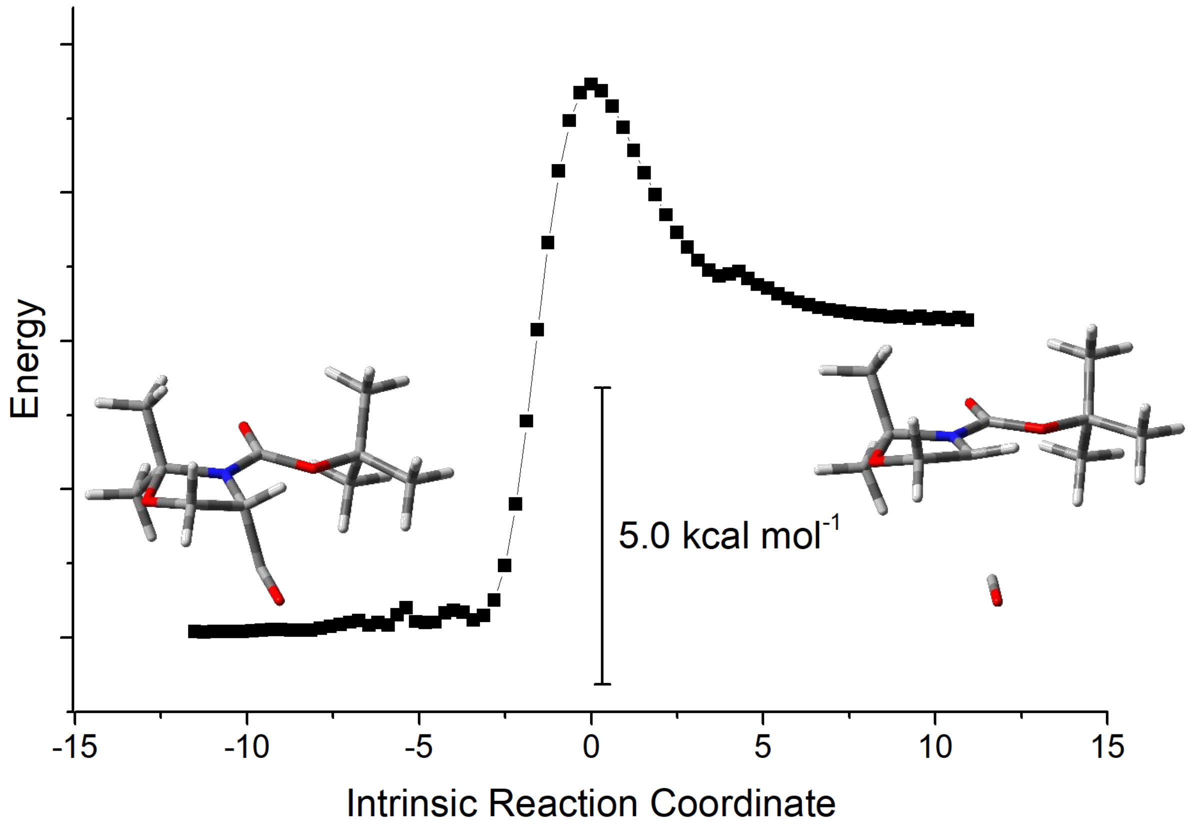

TS-CO (propanoyl)

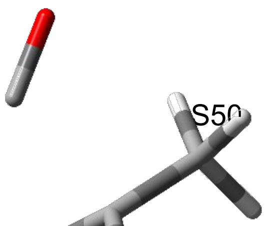

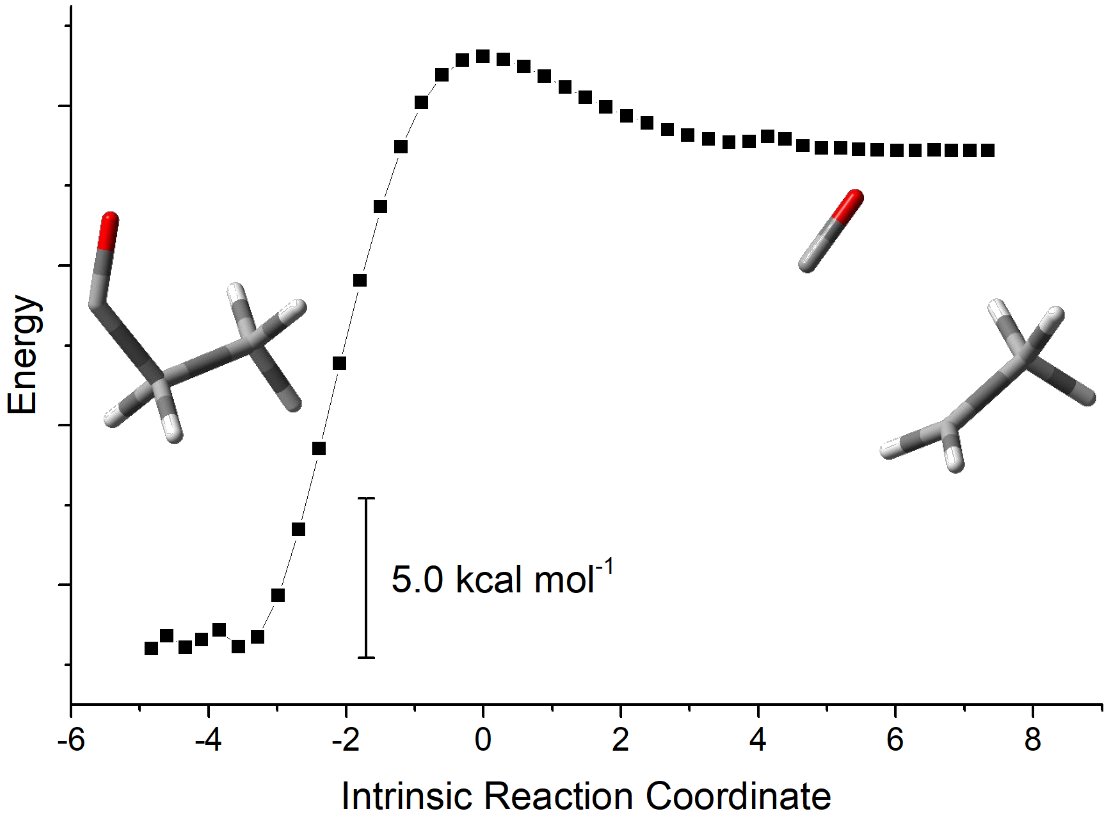

TS-CO (isobutyryl)

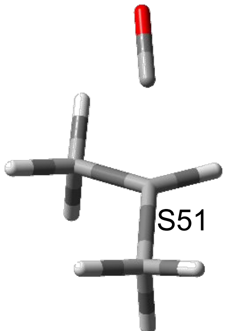

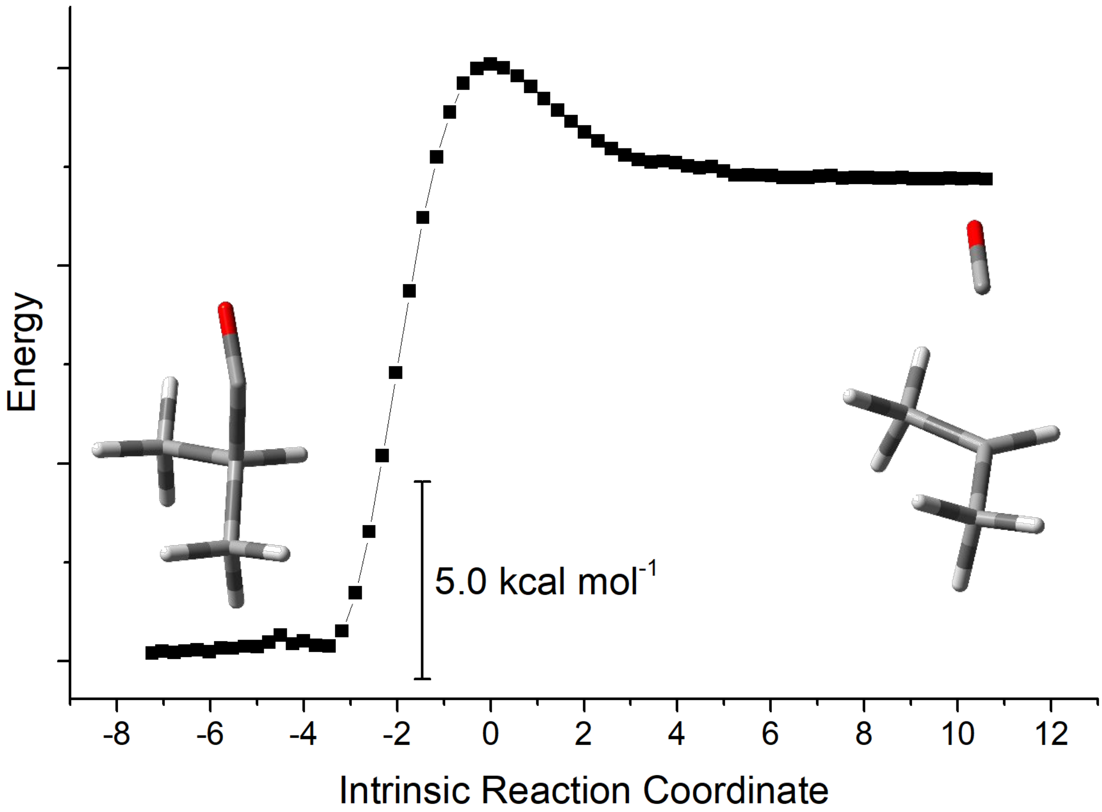

Figure S7: IRC plots of the transition states TS-CO describing the reaction profile reported in Figure 5B (right panel), as from calculations at the ωB97xD/def2TZVP level of theory in the gas phase (total electronic energy values have been reported). The three structures reported in each graph refer, respectively, to those of the first point (left), the transition state (center) and the last point (right) along the reaction coordinate.

9.3.2. Optimized Structures

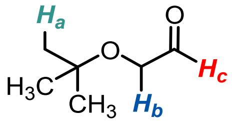

1a

C	-1.14519800	0.37022200	-0.00004300
O	-0.08474600	-0.53701300	0.00050000
C	1.25069200	-0.01844600	0.00000700
C	1.51039000	0.80739300	-1.25857000
C	1.51040300	0.80995500	1.25689100
C	2.12260400	-1.26608500	0.00127000
H	1.24773000	0.23360000	-2.14869400
H	0.94127000	1.73842000	-1.26168300
H	2.56715700	1.07123400	-1.31844300
H	1.24768700	0.23801800	2.14819500
H	2.56719000	1.07383400	1.31625100
H	0.94135800	1.74103200	1.25808400
H	3.17930900	-0.99582700	0.00094300
H	1.91603100	-1.86889200	0.88633500
H	1.91595700	-1.87073400	-0.88251900
H	-1.15345400	1.01995600	0.88463200
H	-1.15319500	1.01921700	-0.88527200
C	-2.43321300	-0.42014200	0.00006100
O	-3.51688300	0.08992000	0.00004200
H	-2.29808100	-1.52049300	0.00013500

Zero-point correction=	0.173754
Thermal correction to Energy=	0.183798
Thermal correction to Enthalpy=	0.184742
Thermal correction to Gibbs Free Energy=	0.137736
E(wB97XD, MeCN)	-386.330541

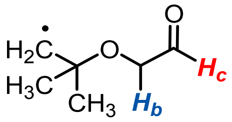

1a• (*Ha*)

C	-1.11095300	0.38601200	0.02601700
O	-0.04863000	-0.44192200	-0.34953600
C	1.26247500	-0.01666900	0.04495100
C	1.60892500	1.33480000	-0.57978600
C	1.36268500	0.03001600	1.53383500
C	2.18295900	-1.10664500	-0.50860300
H	1.46522600	1.29459900	-1.66031500
H	0.99224600	2.13727400	-0.17270900
H	2.65191400	1.58224000	-0.37774300
H	0.93195200	-0.77367600	2.11758800
H	1.98664900	0.76018300	2.03089500
H	1.91034300	-2.07542500	-0.09010900
H	2.08374200	-1.15535200	-1.59458400
H	-1.02097200	0.71977300	1.07030800
H	-1.19288500	1.28242100	-0.60059500
C	-2.39527200	-0.39863000	-0.09491300

O	-3.48121700	0.09588100	0.01019500
H	-2.25654800	-1.48311600	-0.27995300
H	3.22219600	-0.89389400	-0.25706800

Zero-point correction= 0.158803
Thermal correction to Energy= 0.169066
Thermal correction to Enthalpy= 0.170010
Thermal correction to Gibbs Free Energy= **0.122697**

E(UwB97XD, MeCN) **-385.655241**

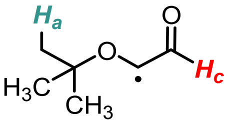

1a• (Hb)

C	-1.15983000	-0.16927000	0.00018700
O	-0.08202300	0.59958600	0.00066200
C	1.23891500	-0.00891600	-0.00003700
C	1.41726400	-0.84439700	1.26152800
C	1.41706600	-0.84122100	-1.26371600
C	2.18208500	1.18270600	0.00145200
H	1.22283400	-0.24023000	2.14825900
H	0.75129900	-1.70774700	1.27607900
H	2.44205300	-1.21395400	1.31238500
H	1.22323300	-0.23464300	-2.14892600
H	2.44159400	-1.21139800	-1.31530300
H	0.75040100	-1.70399500	-1.28057700
H	3.21742100	0.84061800	0.00071600
H	2.01809200	1.79921500	-0.88265900
H	2.01852100	1.79668100	0.88740700
H	-1.06572100	-1.24914700	-0.00020300
C	-2.43982500	0.43443500	0.00019700
O	-3.48129100	-0.21648100	-0.00007300
H	-2.44726900	1.53973700	0.00045600

Zero-point correction= 0.161611
Thermal correction to Energy= 0.171268
Thermal correction to Enthalpy= 0.172212
Thermal correction to Gibbs Free Energy= **0.126723**

E(UwB97XD, MeCN) **-385.697954**

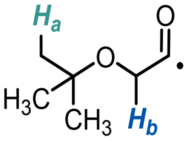

1a• (Hc)

C	-0.46432400	-0.96847600	1.14253200
C	-0.99803800	-0.09048900	0.01482700
H	0.15015900	-1.77529300	0.74462200
H	0.13418700	-0.39769200	1.85599400
H	-1.29925200	-1.40315700	1.69387900

C	-1.83132200	1.06418400	0.56768500
C	-1.82370300	-0.92149600	-0.95582700
H	-2.17325500	1.70527500	-0.24563800
H	-2.70449100	0.67688400	1.09471400
H	-1.26842100	1.67335000	1.27748300
H	-2.68594100	-1.35602100	-0.44814600
H	-2.17920800	-0.29962200	-1.77811500
H	-1.21687700	-1.72714200	-1.36968300
C	1.10666500	1.11137000	-0.19196300
H	1.46830300	1.87838300	-0.88290300
H	0.82095100	1.62845300	0.72972600
C	2.34581000	0.27793200	0.14179800
O	2.55066100	-0.85697000	-0.06853400
O	0.06725300	0.42677400	-0.80474700

Zero-point correction= 0.162252
Thermal correction to Energy= 0.171747
Thermal correction to Enthalpy= 0.172692
Thermal correction to Gibbs Free Energy= 0.127777

E(UwB97XD, MeCN) -385.678025

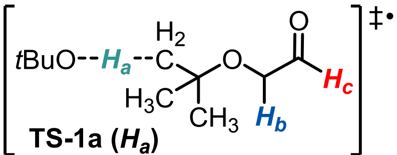

TS-1a (*H*$_a$)

C	1.32636100	1.30047400	0.44839500
O	2.06447300	0.36967100	-0.29896100
C	1.89550300	-1.01465900	-0.00078900
C	2.00440700	-1.29157600	1.49685400
C	0.57973200	-1.52009500	-0.56296100
C	3.03794100	-1.69822500	-0.75211500
H	2.94326200	-0.89288300	1.88422000
H	1.17483700	-0.85051300	2.04963400
H	1.98725300	-2.36715400	1.67710700
H	0.39431200	-1.18391700	-1.58185200
H	0.44163300	-2.59494600	-0.45415500
H	2.98139500	-1.46818400	-1.81644900
H	3.99323700	-1.33492700	-0.37092900
H	0.32546300	0.93909500	0.70982100
H	1.83303800	1.57107100	1.38276900
C	1.15380300	2.54887900	-0.38002400
O	0.64632000	3.55184000	0.03616000
H	1.52268300	2.46641000	-1.42305500
H	2.99483300	-2.77984200	-0.62157700
C	-2.41303900	-0.30973700	-0.00927800
C	-3.51749100	0.08567400	0.97645800
H	-3.76944900	-0.75553800	1.62236600
H	-3.19052800	0.91936500	1.59812000
H	-4.41029000	0.38838500	0.42642000
C	-2.05906400	0.87749000	-0.90430100
H	-1.77605700	1.74192700	-0.30202900
H	-1.22410100	0.62748700	-1.56278300
H	-2.90754600	1.15895600	-1.53045800
C	-2.85598700	-1.51251700	-0.84101900

H	-2.07714500	-1.79793600	-1.55100500
H	-3.05883400	-2.36413200	-0.19054700
H	-3.75976500	-1.28116400	-1.40752700
O	-1.32873400	-0.65531200	0.82542800
H	-0.38770300	-1.06540000	0.13357500

Zero-point correction= 0.293387
Thermal correction to Energy= 0.310396
Thermal correction to Enthalpy= 0.311340
Thermal correction to Gibbs Free Energy= **0.248039**

E(UwB97XD, MeCN) **-619.343369**

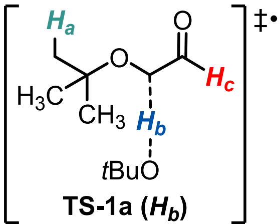

TS-1a (H_b)

C	-0.57363600	0.89472000	0.09890500
O	-1.72115000	0.54353900	-0.56187600
C	-2.56008500	-0.48882800	-0.00311600
C	-1.75660600	-1.75938900	0.24919500
C	-3.21268900	0.01905400	1.28009100
C	-3.61030000	-0.72023700	-1.07853400
H	-1.24435800	-2.07617800	-0.65875200
H	-1.00187000	-1.61454400	1.02268900
H	-2.42809700	-2.55544900	0.57430300
H	-3.73939900	0.95581700	1.09347900
H	-3.93203500	-0.71579200	1.64377100
H	-2.48249800	0.18072500	2.07444700
H	-4.32297700	-1.47934000	-0.75407600
H	-4.15379600	0.20270000	-1.28431000
H	-3.13713200	-1.05682800	-2.00128100
H	-0.61947000	0.83638000	1.18995300
C	-0.10178600	2.23814500	-0.35815900
O	0.70996500	2.89145600	0.23923800
H	-0.54356300	2.58114400	-1.31382500
C	3.19688000	0.49526300	-0.88415800
C	2.51595600	-0.56508300	-0.01974700
H	3.20703400	0.17681500	-1.92742700
H	2.66663300	1.44537100	-0.81275100
H	4.22615100	0.66145300	-0.55963400
C	2.46048600	-0.12689500	1.44526400
C	3.25980600	-1.90646100	-0.13761500
H	1.91402600	-0.86243900	2.03796900
H	3.46757600	-0.02748800	1.85398100
H	1.96582300	0.84242000	1.53335800
H	4.29222900	-1.77641700	0.19170100
H	2.78094300	-2.66340600	0.48350000
H	3.26256500	-2.24993500	-1.17224000
O	1.23551500	-0.86300300	-0.49864900
H	0.31941000	0.13731600	-0.19732000

Zero-point correction= 0.294231

Thermal correction to Energy=	0.311297
Thermal correction to Enthalpy=	0.312241
Thermal correction to Gibbs Free Energy=	**0.248493**

E(UwB97XD, MeCN)	**-619.351872**

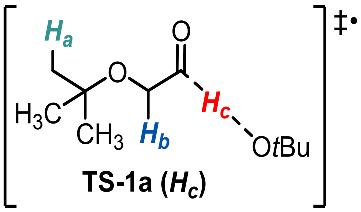

TS-1a (*Hc*)

C	-1.47934400	1.56618200	-0.58317400
O	-1.89852700	0.30479500	-0.96229200
C	-2.43896600	-0.56962800	0.04789000
C	-1.53387500	-0.64837200	1.27478100
C	-3.83935700	-0.09661500	0.42945600
C	-2.49673000	-1.92180600	-0.64579000
H	-0.52864100	-0.95801900	0.98756800
H	-1.47695000	0.30427300	1.80709000
H	-1.93617300	-1.38147900	1.97527200
H	-4.46813800	-0.02619300	-0.45884400
H	-4.29799900	-0.80069500	1.12545300
H	-3.81910300	0.87919100	0.91906900
H	-2.92876300	-2.67280200	0.01723600
H	-3.10851200	-1.85755700	-1.54635100
H	-1.49193800	-2.23527400	-0.92997700
H	-1.53681500	2.21482300	-1.46351300
H	-2.08704700	2.02917200	0.20271500
C	-0.01980800	1.62969300	-0.13341300
O	0.48262500	2.56853100	0.39148900
H	0.60063900	0.62892800	-0.39282600
C	2.59125600	-0.43379600	-0.02018400
C	3.38423300	0.72864200	-0.62022300
H	3.44725900	0.61717200	-1.70342000
H	2.89794300	1.67877000	-0.39395100
H	4.39617400	0.76092400	-0.21152600
C	2.45895600	-0.28464400	1.49705000
H	1.95278800	0.64967900	1.74566300
H	1.88280100	-1.11495800	1.90838800
H	3.44082400	-0.27259900	1.97417400
C	3.27236500	-1.76929800	-0.36002800
H	2.70118300	-2.60323800	0.04846700
H	3.35010200	-1.89183100	-1.44027300
H	4.27527700	-1.78347400	0.07089600
O	1.33374100	-0.54419700	-0.62413400

Zero-point correction=	0.294683
Thermal correction to Energy=	0.311594
Thermal correction to Enthalpy=	0.312539
Thermal correction to Gibbs Free Energy=	**0.249110**

E(UwB97XD, MeCN)	**-619.354801**

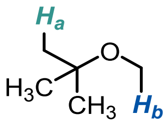

S1

C	-2.02255900	0.00003000	-0.14610000
O	-0.78348100	0.00009900	-0.80038800
C	0.39453800	-0.00000200	0.00711800
C	0.46658000	1.25749200	0.87368500
C	0.46580300	-1.25683800	0.87470200
C	1.53009300	-0.00076300	-1.00775100
H	0.33926600	2.14821100	0.25625000
H	-0.29782500	1.26093600	1.65224700
H	1.43757800	1.31581600	1.36801600
H	0.33785700	-2.14799100	0.25802000
H	1.43680400	-1.31538600	1.36899600
H	-0.29854500	-1.25912600	1.65332400
H	2.49822300	-0.00081300	-0.50467800
H	1.46504400	-0.88511400	-1.64281500
H	1.46554100	0.88307500	-1.64357800
H	-2.16980700	-0.89032000	0.47585500
H	-2.16931200	0.88962700	0.47705300
H	-2.78370700	0.00077600	-0.92551100

Zero-point correction=	0.164620
Thermal correction to Energy=	0.172751
Thermal correction to Enthalpy=	0.173695
Thermal correction to Gibbs Free Energy=	**0.132130**
E(wB97XD, MeCN)	**-273.000199**

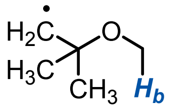

S1• (*H_a*)

C	-2.00213300	-0.06942500	-0.10355900
O	-0.76903400	-0.51945000	-0.60119000
C	0.39586200	-0.00864800	0.04822700
C	0.46946900	1.51650800	-0.05513800
C	0.43829400	-0.45543900	1.47326300
C	1.55571400	-0.64022600	-0.72786800
H	0.36756600	1.82423900	-1.09688300
H	-0.31225700	1.99996200	0.53189500
H	1.43185700	1.86949300	0.31838600
H	0.13089600	-1.46391700	1.71903600
H	0.91887800	0.14468700	2.23429500
H	1.48253500	-1.72700400	-0.68786000
H	1.51168600	-0.32684800	-1.77278000
H	-2.20099000	0.97434700	-0.37266600
H	-2.77146600	-0.69481100	-0.55493000
H	-2.06329900	-0.16641400	0.98720300
H	2.51362700	-0.33475800	-0.30572100

Zero-point correction=	0.149837
Thermal correction to Energy=	0.158122

```
Thermal correction to Enthalpy=                    0.159066
Thermal correction to Gibbs Free Energy=           0.117920

E(wB97XD, MeCN)                                 -272.324827
```

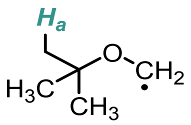

S1• (*H_b*)

C	2.03743300	-0.14772000	0.06480800
O	0.84562600	-0.59641700	0.50020300
C	-0.35753600	0.00967200	-0.00979800
C	-0.43969500	1.46946300	0.42926900
C	-0.40422700	-0.11788800	-1.52912000
C	-1.47552000	-0.79917700	0.63108400
H	-0.33088000	1.54445000	1.51198900
H	0.33096800	2.08151700	-0.04024400
H	-1.40859200	1.88486100	0.14845400
H	-0.30713500	-1.16366700	-1.82311400
H	-1.35499500	0.26325800	-1.90505000
H	0.40029200	0.44701200	-2.00079800
H	-2.44811500	-0.42566500	0.30801900
H	-1.39239400	-1.84878900	0.34749700
H	-1.41856100	-0.73016400	1.71801500
H	2.12371800	0.85733400	-0.33017300
H	2.87795100	-0.62490900	0.54632300

```
Zero-point correction=                             0.150717
Thermal correction to Energy=                      0.158761
Thermal correction to Enthalpy=                    0.159705
Thermal correction to Gibbs Free Energy=           0.119195

E(UwB97XD, MeCN)                                -272.337595
```

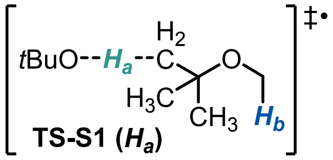

C	1.84891200	2.06196300	-0.07424700
O	2.61005100	0.89677600	-0.28099500
C	1.98892700	-0.34790700	0.00212300
C	1.58162900	-0.45749400	1.47196100
C	0.80022500	-0.58912600	-0.91228100
C	3.06881300	-1.38155400	-0.32203000
H	2.44100000	-0.24920700	2.11090800
H	0.77588100	0.23405800	1.71611700
H	1.22555100	-1.46653600	1.68702600
H	0.96265300	-0.25640900	-1.93681400
H	0.43154300	-1.61300900	-0.87224000
H	3.36478800	-1.30332500	-1.36861700
H	3.94617700	-1.19423000	0.29853700
H	1.57693000	2.19892400	0.97728100
H	2.47886100	2.89575200	-0.38115700

H	0.93099800	2.07283200	-0.66968400
H	2.71016400	-2.39319600	-0.12900300
C	-2.29702100	-0.03284300	-0.00688800
C	-2.66191300	-0.65019100	-1.35654400
H	-2.77706500	0.13234900	-2.10743400
H	-1.87922000	-1.33386500	-1.69081500
H	-3.59564600	-1.21193500	-1.28954000
C	-2.09133000	-1.11387400	1.05412400
H	-1.29648800	-1.79999900	0.75606400
H	-1.81031000	-0.65916000	2.00487400
H	-3.00325700	-1.69600900	1.20043200
C	-3.39467000	0.93885000	0.44083800
H	-3.12731100	1.40118700	1.39133200
H	-3.53194400	1.72413600	-0.30272800
H	-4.33682500	0.40158000	0.56395100
O	-1.15547000	0.78313200	-0.12169700
H	-0.18455700	0.10986300	-0.51928500

Zero-point correction= 0.284225
Thermal correction to Energy= 0.299341
Thermal correction to Enthalpy= 0.300285
Thermal correction to Gibbs Free Energy= **0.242157**

E(UwB97XD, MeCN) **-506.012489**

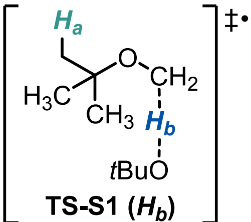

TS-S1 (H_b)

C	-0.54904900	-1.50774900	-0.01187800
O	-1.35071200	-0.64831800	-0.71461900
C	-2.28277300	0.17527700	0.01402700
C	-3.43519600	-0.68964000	0.51848100
C	-1.59466700	0.90140900	1.16505800
C	-2.77748100	1.17010900	-1.02505000
H	-3.89620500	-1.22714800	-0.31113900
H	-3.10080000	-1.41553000	1.26117200
H	-4.19321300	-0.06261400	0.99016100
H	-0.74191900	1.47497800	0.80408600
H	-2.30291300	1.58382300	1.63729500
H	-1.23234900	0.20998100	1.92599600
H	-3.52611200	1.83388000	-0.59058400
H	-1.94730300	1.77411000	-1.39327800
H	-3.22471300	0.64519400	-1.87003300
H	-1.01849700	-1.97404000	0.85982500
H	-0.14422300	-2.25331600	-0.69429200
C	2.83860700	-1.05728400	-0.88945900
C	2.36067000	0.09286600	-0.00082700
H	3.26325600	-1.85251700	-0.27539900
H	2.00706700	-1.46893800	-1.46415800
H	3.59771800	-0.71579400	-1.59605600
C	1.67369300	1.17341900	-0.83836700
C	3.55096700	0.69201900	0.76047500

H	1.37795500	2.01006900	-0.20293800
H	2.34286400	1.54972500	-1.61502700
H	0.77967200	0.77196200	-1.31895400
H	4.26993100	1.10771900	0.05166300
H	3.21292400	1.48723400	1.42534100
H	4.04629700	-0.07483100	1.35651900
O	1.51354700	-0.37233000	1.01487300
H	0.41924700	-0.94132200	0.46300600

Zero-point correction= 0.285216
Thermal correction to Energy= 0.300108
Thermal correction to Enthalpy= 0.301052
Thermal correction to Gibbs Free Energy= **0.243105**

E(UwB97XD, MeCN) **-506.020795**

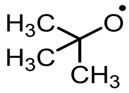

*t*BuO•

C	0.00013500	-0.02519500	0.08411900
C	1.27145700	-0.78386400	-0.30948200
H	2.15536800	-0.21230500	-0.02597500
H	1.30487000	-1.74745100	0.20109300
H	1.29646800	-0.96591600	-1.38552300
C	-1.26422300	-0.79531600	-0.30954000
H	-1.28888900	-1.75943600	0.20054100
H	-2.15326100	-0.23195000	-0.02556300
H	-1.28781700	-0.97705000	-1.38565000
C	-0.00625800	1.37544600	-0.58197100
H	-0.89737900	1.93163900	-0.29437800
H	0.87943100	1.94001100	-0.29389800
H	-0.00524500	1.23357100	-1.66361800
O	-0.00127600	0.27030700	1.42177600

Zero-point correction= 0.122952
Thermal correction to Energy= 0.129414
Thermal correction to Enthalpy= 0.130359
Thermal correction to Gibbs Free Energy= **0.093462**

E(UwB97XD, MeCN) **-233.025685**

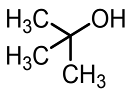

*t*BuOH

C	-0.00618500	-0.00007600	0.01827100
C	0.67893600	-1.25505300	-0.51783700
H	1.73644300	-1.26609500	-0.23962600
H	0.20607300	-2.14723800	-0.10530300
H	0.62024300	-1.30070200	-1.60703600
C	-1.48573100	-0.00169200	-0.33477000
H	-1.97081500	-0.88652600	0.07924100
H	-1.97264300	0.88203000	0.07941000
H	-1.62318600	-0.00182200	-1.41716500

C	0.67601900	1.25655900	-0.51801300
H	0.20094300	2.14758100	-0.10557500
H	1.73348600	1.27006100	-0.23976400
H	0.61727400	1.30181100	-1.60722400
O	0.03939100	0.00011100	1.44483700
H	0.95882300	0.00158500	1.71843600

Zero-point correction= 0.136405
Thermal correction to Energy= 0.143061
Thermal correction to Enthalpy= 0.144006
Thermal correction to Gibbs Free Energy= **0.107490**

E(wB97XD, MeCN) **-233.703251**

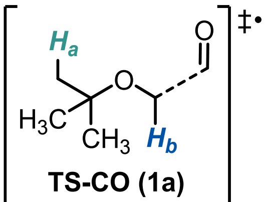

TS-CO (1a)

C	-0.41623400	-0.88216700	1.18395800
C	-1.04823400	-0.11967800	0.02585600
H	0.27140200	-1.64186000	0.81363300
H	0.13004600	-0.21938100	1.85686500
H	-1.19837200	-1.37365400	1.76410200
C	-1.99505300	0.96727300	0.52470200
C	-1.77190300	-1.07294600	-0.91186600
H	-2.39581800	1.53514500	-0.31578000
H	-2.82711900	0.51319000	1.06437600
H	-1.49736600	1.65848800	1.20624700
H	-2.57874400	-1.58261000	-0.38385600
H	-2.19677800	-0.52724900	-1.75499300
H	-1.07861200	-1.82068600	-1.29755500
C	0.88180300	1.29198400	-0.26475100
H	1.37788600	1.91061800	-1.00332400
H	0.65698900	1.75908000	0.68933500
C	2.66067000	0.23009600	0.29819200
O	2.71416700	-0.81645700	-0.15916600
O	-0.03039300	0.47915000	-0.81278500

Zero-point correction= 0.158752
Thermal correction to Energy= 0.168828
Thermal correction to Enthalpy= 0.169773
Thermal correction to Gibbs Free Energy= **0.123029**

E(UwB97XD, MeCN) **-385.658226**

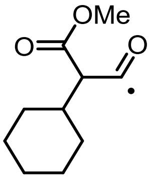

1e•

C	-3.62017600	-0.59191700	0.22708400
C	-3.24183900	0.83352100	-0.16221400
C	-1.84706100	1.20034600	0.33641000
C	-0.79342700	0.20952300	-0.16135900
C	-1.17705400	-1.21763000	0.24068500
C	-2.57102300	-1.58699300	-0.25769100
H	-1.58921200	2.21378300	0.02329900
H	-3.26777300	0.92840100	-1.25318200
H	-3.97299900	1.54290800	0.23100100
H	-3.70213500	-0.65741300	1.31787500
H	-4.60164900	-0.84781500	-0.17784000
H	-0.75550900	0.25446800	-1.25484000
H	-0.44550800	-1.92801900	-0.14846900
H	-1.15162200	-1.29322000	1.33510100
H	-2.56502000	-1.60251000	-1.35277700
H	-2.82444100	-2.59797600	0.06822700
H	-1.84310900	1.20129500	1.43357600
C	0.60458200	0.55519600	0.36280400
H	0.60731700	0.51368500	1.45674200
C	1.69050700	-0.35907300	-0.17385300
C	3.76546900	-1.32680500	0.30265400
H	4.43114100	-1.38661300	1.15902700
H	3.45565700	-2.32322200	-0.01144900
H	4.25785200	-0.82616100	-0.53039900
O	2.63874400	-0.56784300	0.74032700
O	1.71821900	-0.79982400	-1.28927800
C	1.00009500	2.00334300	0.04801400
O	2.03135900	2.42908500	-0.30993700

Zero-point correction= 0.240241
Thermal correction to Energy= 0.253637
Thermal correction to Enthalpy= 0.254581
Thermal correction to Gibbs Free Energy= 0.198570

E(UwB97XD, MeCN) -615.786054

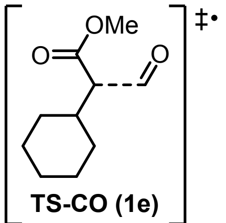

TS-CO (1e)

C	3.71911900	-0.39205400	-0.22987800
C	3.19570900	0.97588900	0.19686900
C	1.77747300	1.21598300	-0.31072100
C	0.81950100	0.11244900	0.14017500
C	1.35165100	-1.26456300	-0.30235000
C	2.76988700	-1.50225900	0.20872500
H	1.41230900	2.18498900	0.03498900
H	3.19914400	1.03822200	1.29049200
H	3.85766300	1.76615800	-0.16376000
H	3.82012000	-0.41455200	-1.32085300
H	4.71648700	-0.56157800	0.18167600
H	0.76694700	0.10722100	1.23377000
H	0.68469600	-2.04836600	0.05966300

H	1.34696000	-1.31154000	-1.39781200
H	2.75007400	-1.55111400	1.30262900
H	3.12950900	-2.47197400	-0.14236100
H	1.78664600	1.25103600	-1.40717100
C	-0.56371500	0.31800800	-0.38648000
H	-0.66306700	0.60426700	-1.42980600
C	-1.65170100	-0.52730300	0.12985900
C	-3.84247100	-1.26328900	-0.24635200
H	-4.59483700	-1.15063200	-1.02223600
H	-3.59354600	-2.31538400	-0.10676600
H	-4.20630600	-0.85811800	0.69803700
O	-2.70656900	-0.53408200	-0.69892900
O	-1.62604100	-1.11726000	1.18278000
C	-1.20617900	2.13449900	0.37585500
O	-2.24594700	2.51224300	0.10806100

Zero-point correction= 0.237066
Thermal correction to Energy= 0.250814
Thermal correction to Enthalpy= 0.251758
Thermal correction to Gibbs Free Energy= **0.194638**

E(UwB97XD, MeCN) **-615.767361**

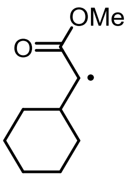

S5•

C	3.48035800	0.24421000	0.17138300
C	2.94099400	-0.99764300	-0.53222000
C	1.56243300	-1.38467200	-0.00546500
C	0.56751500	-0.22472600	-0.11934800
C	1.11975900	1.02672400	0.59980100
C	2.49797800	1.40685400	0.06696400
H	1.18058700	-2.25364700	-0.54630800
H	2.87113100	-0.80067600	-1.60752500
H	3.63310300	-1.83387200	-0.41123600
H	3.65279400	0.01241400	1.22847700
H	4.44743600	0.52727800	-0.24991400
H	0.44589900	0.04482000	-1.17392300
H	0.41842400	1.85300200	0.47192500
H	1.18548700	0.81849700	1.67366500
H	2.40399400	1.70856900	-0.98161800
H	2.87660700	2.27491200	0.61122600
H	1.64545600	-1.67841200	1.04776400
C	-0.76446000	-0.58212500	0.41673400
H	-0.85157200	-1.23341500	1.27840200
C	-1.97454800	-0.01123800	-0.12821300
C	-4.30927100	0.08056600	0.05038500
H	-5.07870300	-0.35925200	0.67985100
H	-4.33005100	1.16805600	0.13004200
H	-4.46836900	-0.19636000	-0.99241100
O	-3.07439700	-0.43658000	0.52687200
O	-2.02019900	0.76162900	-1.06168800

Zero-point correction=	0.229928
Thermal correction to Energy=	0.241313
Thermal correction to Enthalpy=	0.242257
Thermal correction to Gibbs Free Energy=	**0.191058**

E(UwB97XD, MeCN) **-502.455283**

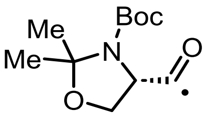

1h•

C	1.06817400	1.20472300	0.50995300
C	2.56898500	1.18188000	0.81082400
C	2.11090300	-0.88164700	-0.10402800
H	0.47178700	1.51552200	1.36942800
H	3.07712000	2.11236700	0.56381000
H	2.72291700	0.96103900	1.87364200
O	3.06881400	0.16253800	-0.01287100
C	0.75006600	2.20446400	-0.62505900
O	0.60992200	3.36098500	-0.49231300
N	0.84422400	-0.16586500	0.15847800
C	-0.38717800	-0.72402600	0.00506800
O	-0.57845400	-1.88389400	-0.27903700
O	-1.33055200	0.20197700	0.22496600
C	-2.74960600	-0.09559100	0.07025000
C	-3.17810600	-1.15856300	1.07312900
C	-3.04352000	-0.50570100	-1.36665000
C	-3.40579000	1.23977900	0.38953200
H	-2.90085700	-0.85768800	2.08491700
H	-2.72015700	-2.11918400	0.84988400
H	-4.26318200	-1.26856100	1.03671800
H	-2.66670800	0.24921300	-2.05865800
H	-4.12322400	-0.58494600	-1.50300600
H	-2.59001300	-1.46485300	-1.60512900
H	-4.48901800	1.15087600	0.30066500
H	-3.06174400	2.01075200	-0.30079400
H	-3.16622600	1.55205100	1.40679700
C	2.36502600	-1.94238600	0.95776200
H	1.57999100	-2.69657000	0.92333400
H	2.37788900	-1.49391400	1.95208600
H	3.32815900	-2.41838100	0.77415600
C	2.18715400	-1.44002500	-1.51164000
H	1.47409100	-2.25207400	-1.63543400
H	3.19568300	-1.81289500	-1.69046000
H	1.96942400	-0.65198700	-2.23211100

Zero-point correction=	0.286318
Thermal correction to Energy=	0.303803
Thermal correction to Enthalpy=	0.304748
Thermal correction to Gibbs Free Energy=	**0.240258**

E(UwB97XD, MeCN) **-785.699871**

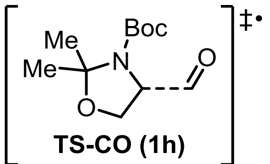

TS-CO (1h)

C	1.11120400	1.01648800	0.71701400
C	2.57229400	0.94510400	1.03044500
C	2.03217600	-0.99282400	-0.11469100
H	0.40521700	1.48075800	1.39136200
H	3.10749800	1.87608100	0.84250600
H	2.73418100	0.65772700	2.08051300
O	3.05195700	-0.04186900	0.14577500
C	0.93228900	2.42250200	-0.82058300
O	1.24128100	3.47871500	-0.50373100
N	0.80015400	-0.23655300	0.22265100
C	-0.46435000	-0.70421900	-0.03017100
O	-0.69283800	-1.80199800	-0.48127700
O	-1.36637700	0.21701100	0.31048600
C	-2.79273700	0.01005700	0.08249800
C	-3.29390800	-1.16329300	0.91382100
C	-3.06103000	-0.17820900	-1.40467300
C	-3.40032500	1.31549800	0.57431200
H	-3.03372800	-1.02152300	1.96416600
H	-2.87066500	-2.10268000	0.56622500
H	-4.38106600	-1.21778400	0.83726900
H	-2.62740800	0.64612800	-1.97303400
H	-4.13858800	-0.18255900	-1.57608700
H	-2.64586100	-1.11609100	-1.76608100
H	-4.48367700	1.28856300	0.45293600
H	-3.00724700	2.15884600	0.00552300
H	-3.17404600	1.47090500	1.62997000
C	2.17961700	-2.20055300	0.79912400
H	1.35727400	-2.89621300	0.63862700
H	2.18193800	-1.88766400	1.84419900
H	3.12231800	-2.70240300	0.58168200
C	2.11127400	-1.35355200	-1.58496300
H	1.36966300	-2.10889200	-1.83431700
H	3.10825200	-1.74046400	-1.79561700
H	1.94364800	-0.46372400	-2.19122900

Zero-point correction=	0.282880
Thermal correction to Energy=	0.300890
Thermal correction to Enthalpy=	0.301834
Thermal correction to Gibbs Free Energy=	**0.235337**
E(UwB97XD, MeCN)	**-785.684012**

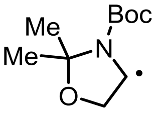

S7•

C	-1.13043700	1.64932200	-0.13090400
C	-2.59110300	1.85779600	0.03902100

C	-2.21943000	-0.42869900	-0.02406200
H	-0.33864100	2.33580600	0.11110200
H	-3.01003500	2.60978900	-0.63280200
H	-2.86033300	2.14117500	1.07066100
O	-3.14926200	0.60244100	-0.29803600
N	-0.91898400	0.28537000	-0.08943600
C	0.28659600	-0.35489100	-0.00743300
O	0.40449600	-1.55665200	0.09754000
O	1.28440600	0.53381400	-0.05614500
C	2.67806300	0.11384400	-0.00368800
C	2.97380700	-0.57571500	1.32218300
C	3.00752100	-0.77162100	-1.19871400
C	3.42857500	1.43479400	-0.09623100
H	2.67086300	0.06143700	2.15487500
H	2.45460400	-1.52812800	1.39648500
H	4.04757800	-0.75309400	1.40269000
H	2.73077400	-0.26995100	-2.12745600
H	4.08216100	-0.96022400	-1.22020500
H	2.48574900	-1.72385900	-1.14010400
H	4.50362600	1.25339100	-0.07073500
H	3.18453300	1.94951300	-1.02618400
H	3.16653100	2.08398800	0.74019400
C	-2.43849400	-0.99275800	1.37404000
H	-1.68795600	-1.74965800	1.59732000
H	-2.37398400	-0.19721800	2.11788600
H	-3.42990400	-1.44233900	1.42807200
C	-2.37078400	-1.47661100	-1.10831600
H	-1.69861600	-2.31257400	-0.93151500
H	-3.40160600	-1.83109300	-1.10599500
H	-2.15545900	-1.03414900	-2.08047900

Zero-point correction=	0.275395
Thermal correction to Energy=	0.291063
Thermal correction to Enthalpy=	0.292008
Thermal correction to Gibbs Free Energy=	**0.232826**
E(UwB97XD, MeCN)	**-672.364568**

CO

C	0.00000000	0.00000000	-0.64167900
O	0.00000000	0.00000000	0.48125900

Zero-point correction=	0.005119
Thermal correction to Energy=	0.007480
Thermal correction to Enthalpy=	0.008424
Thermal correction to Gibbs Free Energy=	**-0.013994**
E(UwB97XD, MeCN)	**-113.322335**

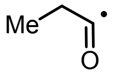

Propanoyl

C	0.94390200	0.50024700	-0.00012900
O	1.52724000	-0.52003500	0.00004200

C	-0.55172800	0.72885500	0.00002800
H	-0.76352800	1.35557600	-0.86992900
H	-0.76307400	1.35523300	0.87040800
C	-1.37945900	-0.55079900	-0.00001200
H	-1.16203200	-1.15478400	0.88069900
H	-2.44330500	-0.31105400	0.00015500
H	-1.16227600	-1.15450900	-0.88099500

Zero-point correction=	0.072651
Thermal correction to Energy=	0.077641
Thermal correction to Enthalpy=	0.078585
Thermal correction to Gibbs Free Energy=	**0.044687**

E(UwB97XD, MeCN) **-192.511350**

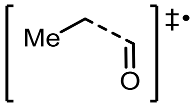

TS-CO (propanoyl)

C	-1.29435300	0.55952000	-0.00022400
O	-1.63926400	-0.52468800	0.00011500
C	0.90624700	0.77928000	0.00011200
H	0.96958800	1.36080100	0.91204900
H	0.96989200	1.36116500	-0.91156900
C	1.44847000	-0.60678900	-0.00006600
H	1.13382000	-1.16310600	-0.88420500
H	2.54521400	-0.58992100	0.00019000
H	1.13341400	-1.16349000	0.88368800

Zero-point correction=	0.067914
Thermal correction to Energy=	0.073659
Thermal correction to Enthalpy=	0.074603
Thermal correction to Gibbs Free Energy=	**0.038203**

E(UwB97XD, MeCN) **-192.479276**

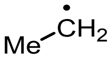

Ethyl

C	-0.79068800	0.00000000	-0.01817300
H	-1.34642700	-0.92590900	0.03970400
H	-1.34643100	0.92590700	0.03970500
C	0.69129900	0.00000100	-0.00185800
H	1.10176900	0.88533400	-0.49197000
H	1.08565200	-0.00005000	1.02480200
H	1.10177000	-0.88528600	-0.49205300

Zero-point correction=	0.059515
Thermal correction to Energy=	0.063487
Thermal correction to Enthalpy=	0.064431
Thermal correction to Gibbs Free Energy=	**0.035317**

E(UwB97XD, MeCN) **-79.159674**

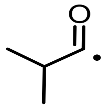

Isobutyryl

C	-0.94196100	-0.67508800	0.20382800
O	-1.93761200	-0.20828300	-0.20900000
C	0.40952100	-0.00036200	0.41466400
H	0.54113200	-0.01708800	1.50201000
C	1.51008700	-0.85446200	-0.21164900
H	1.40108800	-0.89058400	-1.29730800
H	2.48694100	-0.42542200	0.01436600
H	1.48321900	-1.87568100	0.16825200
C	0.41197000	1.44083600	-0.08551300
H	-0.38052700	2.02422200	0.38231700
H	1.37014900	1.91207000	0.14010900
H	0.26119300	1.47320000	-1.16572300

Zero-point correction=	0.101033
Thermal correction to Energy=	0.107318
Thermal correction to Enthalpy=	0.108262
Thermal correction to Gibbs Free Energy=	**0.070773**
E(UwB97XD, MeCN)	**-231.828018**

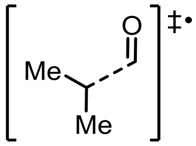

TS-CO (isobutyryl)

C	-1.30589700	-0.74180200	-0.18521900
O	-2.17251400	-0.03193700	0.02464700
C	0.63838600	0.03839900	0.42644100
H	0.42059700	-0.04226600	1.48799000
C	1.60551800	-0.95645200	-0.11780400
H	1.57330300	-0.97949200	-1.20930600
H	2.63108100	-0.69782900	0.17349100
H	1.39811000	-1.96082100	0.25162300
C	0.63258100	1.42150100	-0.13363400
H	-0.22062200	2.00014200	0.22196700
H	1.54211700	1.95837100	0.16441100
H	0.61199800	1.40752200	-1.22606100

Zero-point correction=	0.096564
Thermal correction to Energy=	0.103621
Thermal correction to Enthalpy=	0.104565
Thermal correction to Gibbs Free Energy=	**0.064882**
E(UwB97XD, MeCN)	**-231.801802**

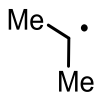

Isopropyl

C	0.00000000	0.53589700	-0.04185300
H	-0.00003900	1.60996300	0.09404200
C	-1.28932000	-0.19813600	0.00259300
H	-1.30319600	-1.02348600	-0.71775800
H	-1.46470600	-0.65252700	0.98940000
H	-2.13873700	0.45212300	-0.20867600
C	1.28933300	-0.19811300	0.00261100
H	2.13895000	0.45265200	-0.20621400
H	1.46347700	-0.65469600	0.98862100
H	1.30418100	-1.02191300	-0.71952000

Zero-point correction= 0.088279
Thermal correction to Energy= 0.093500
Thermal correction to Enthalpy= 0.094444
Thermal correction to Gibbs Free Energy= **0.060943**

E(UwB97XD, MeCN) **-118.481301**

10. References

(1) Protti, S.; Ravelli, D.; Fagnoni, M.; Albini, A. Solar light-driven photocatalyzed alkylations. Chemistry on the window ledge. *Chem. Commun.* **2009**, 10.1039/b917732a, 7351-7353. DOI: 10.1039/b917732a.
(2) Capaldo, L.; Merli, D.; Fagnoni, M.; Ravelli, D. Visible Light Uranyl Photocatalysis: Direct C–H to C–C Bond Conversion. *ACS Catal.* **2019**, *9*, 3054-3058. DOI: 10.1021/acscatal.9b00287.
(3) Masson, T. M.; Zondag, S. D. A.; Schuurmans, J. H. A.; Noël, T. Open-source 3D printed reactors for reproducible batch and continuous-flow photon-induced chemistry: design and characterization. *React. Chem. Eng.* **2024**, *9*, 2218-2225. DOI: 10.1039/d4re00081a.
(4) Luo, T.; Li, Z.; Deng, X. M.; Jiang, K.; Liu, D.; Zhang, H. H.; Shi, T.; Liu, L. Y.; Wen, H. X.; Li, Q. E.; et al. Isolation, synthesis and bioactivity evaluation of isoquinoline alkaloids from Corydalis hendersonii Hemsl. against gastric cancer in vitro and in vivo. *Biorg. Med. Chem.* **2022**, *60*, 116705. DOI: 10.1016/j.bmc.2022.116705.
(5) Kulkarni, C.; Bejagam, K. K.; Senanayak, S. P.; Narayan, K. S.; Balasubramanian, S.; George, S. J. Dipole-moment-driven cooperative supramolecular polymerization. *J. Am. Chem. Soc.* **2015**, *137*, 3924-3932. DOI: 10.1021/jacs.5b00504.
(6) Alessandro Dondoni; Perrone, D. *Synthesis of 1,1-Dimethylethyl (S)-4-Formyl-2,2-Dimethyl-3-Oxazolidinecarboxylate by Oxidation of the Alcohol*; 2000. DOI: 10.15227/orgsyn.077.0064.
(7) Li, B.; Liu, S.; Fan, W.; Shen, X.; Xu, J.; Li, S. Ligand enabled none-oxidative decarbonylation of aliphatic aldehydes. *Chin. Chem. Lett.* **2023**, *34*. DOI: 10.1016/j.cclet.2022.108027.
(8) Scharf, M. J.; List, B. A Catalytic Asymmetric Pictet-Spengler Platform as a Biomimetic Diversification Strategy toward Naturally Occurring Alkaloids. *J. Am. Chem. Soc.* **2022**, *144*, 15451-15456. DOI: 10.1021/jacs.2c06664.
(9) Szcześniak, P.; Buda, S.; Lefevre, L.; Staszewska-Krajewska, O.; Mlynarski, J. Total Asymmetric Synthesis of (+)-Paroxetine and (+)-Femoxetine. *Eur. J. Org. Chem.* **2019**, *2019*, 6973-6982. DOI: 10.1002/ejoc.201901389.
(10) Yang, J.-M.; Guo, F.-K.; Zhao, Y.-T.; Zhang, Q.; Huang, M.-Y.; Li, M.-L.; Zhu, S.-F.; Zhou, Q.-L. Insertion of Alkylidene Carbenes into B–H Bonds. *J. Am. Chem. Soc.* **2020**, *142*, 20924-20929. DOI: 10.1021/jacs.0c09596.
(11) Ung, A. T.; Pyne, S. G. Synthesis of fluorescent and biotinylated analogues of (1R, 2S, 3R)-2-acetyl-4(5)-(1,2,3,4-Tetrahydroxybutyl)imidazole. *Tetrahedron Lett.* **1996**, *37*, 6209-6212. DOI: 10.1016/0040-4039(96)01325-1.
(12) Goess, B. C.; Hannoush, R. N.; Chan, L. K.; Kirchhausen, T.; Shair, M. D. Synthesis of a 10,000-membered library of molecules resembling carpanone and discovery of vesicular traffic inhibitors. *J. Am. Chem. Soc.* **2006**, *128*, 5391-5403. DOI: 10.1021/ja056338g.
(13) Long, F.; Jiang, K.; Song, W.; Luo, W.; Yin, B. Photoinduced Pd-Catalyzed Dearomative 2,5-Difunctionalizition of Furans via Cascade C-C/C-O Bond Formation. *Org. Lett.* **2024**, *26*, 1083-1087. DOI: 10.1021/acs.orglett.3c04345.
(14) Kong, J.; Lacroix, C.; Bournaud, C.; Yamashita, Y.; Kobayashi, S.; Vo-Thanh, G. Enantioselective Acyl-Transfer/Protonation Reactions with Designed Chiral Thiourea-Iminophosphorane Catalysts. *Adv. Synth. Catal.* **2024**, *366*, 1101-1106. DOI: 10.1002/adsc.202301394.
(15) Ji, P.; Zhang, Y.; Wei, Y.; Huang, H.; Hu, W.; Mariano, P. A.; Wang, W. Visible-Light-Mediated, Chemo- and Stereoselective Radical Process for the Synthesis of C-Glycoamino Acids. *Org. Lett.* **2019**, *21*, 3086-3092. DOI: 10.1021/acs.orglett.9b00724.
(16) Meth-Cohn, O.; Moore, C.; Taljaard, H. C. A stereocontrolled approach to electrophilic epoxides. *J. Chem. Soc., Perkin Trans. 1* **1988**, 2663, 10.1039/p19880002663. DOI: 10.1039/p19880002663.
(17) Zhang, Y.; Chen, S. S.; Li, K. D.; Huang, H. M. Cyclic Amine Synthesis via Catalytic Radical-Polar Crossover Cycloadditions. *Angew. Chem. Int. Ed.* **2024**, *63*, e202401671. DOI: 10.1002/anie.202401671.
(18) Rai, P.; Maji, K.; Maji, B. Photoredox/Cobalt Dual Catalysis for Visible-Light-Mediated Alkene-Alkyne Coupling. *Org. Lett.* **2019**, *21*, 3755-3759. DOI: 10.1021/acs.orglett.9b01201.
(19) Capaldo, L.; Ravelli, D. Decatungstate as Direct Hydrogen Atom Transfer Photocatalyst for SOMOphilic Alkynylation. *Org. Lett.* **2021**, *23*, 2243-2247. DOI: 10.1021/acs.orglett.1c00381.

(20) Zhu, Y.; Gao, H.; Tu, J.-L.; Yang, C.; Guo, L.; Zhao, Y.; Xia, W. Iron-catalyzed fragmentation-alkynylation, -alkenylation and -alkylation cascade enabled by photoinduced ligand-to-metal charge transfer. *Org. Chem. Front.* **2024**, *11*, 1729-1735. DOI: 10.1039/d3qo01822a.
(21) Sun, J.; Endo, H.; Emmanuel, M. A.; Oderinde, M. S.; Kawamata, Y.; Baran, P. S. Simplified Modular Access to Enantiopure 1,2-Aminoalcohols via Ni-Electrocatalytic Decarboxylative Arylation. *J. Am. Chem. Soc.* **2024**, *146*, 6209-6216. DOI: 10.1021/jacs.3c14119.
(22) Rohe, S.; Morris, A. O.; McCallum, T.; Barriault, L. Hydrogen Atom Transfer Reactions via Photoredox Catalyzed Chlorine Atom Generation. *Angew. Chem. Int. Ed.* **2018**, *57*, 15664-15669. DOI: 10.1002/anie.201810187.
(23) Zhou, W.; Wu, S.; Melchiorre, P. Tetrachlorophthalimides as Organocatalytic Acceptors for Electron Donor–Acceptor Complex Photoactivation. *J. Am. Chem. Soc.* **2022**, *144*, 8914-8919. DOI: 10.1021/jacs.2c03546.
(24) Gao, Y.; Hu, Y.; Ye, J.; Ma, Z.; Feng, J.; Liu, X.; Lei, P.; Szostak, M. Pd-NHC (NHC = N-Heterocyclic Carbene)-Catalyzed B-Alkyl Suzuki Cross-Coupling of 2-Pyridyl Ammonium Salts by N-C Activation: Application to the Discovery of Agrochemical Molecular Hybrids. *Org. Lett.* **2024**, *26*, 2309-2314. DOI: 10.1021/acs.orglett.4c00549.
(25) Crisp, G. T.; Yu-Lin, J.; Pullman, P. J.; De Savi, C. Elaboration of the side-chain of amino acid derivatives by palladium catalysed couplings. *Tetrahedron* **1997**, *53*, 17489-17500. DOI: 10.1016/s0040-4020(97)10197-1.
(26) *Gaussian 16 Rev. C.01*; Wallingford, CT, 2016.

11. Copy of NMR spectra

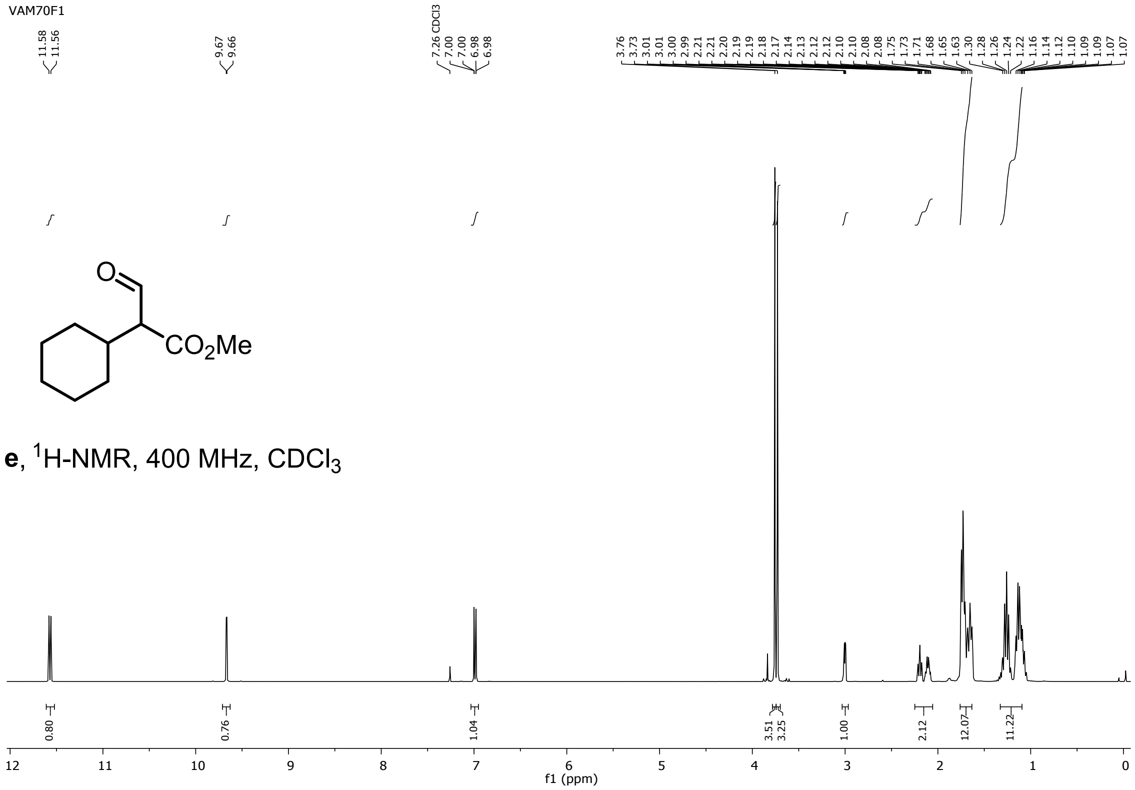

^{1}H NMR (400 MHz, $CDCl_3$) of ***methyl 2-cyclohexyl-3-oxopropanoate (1e).***

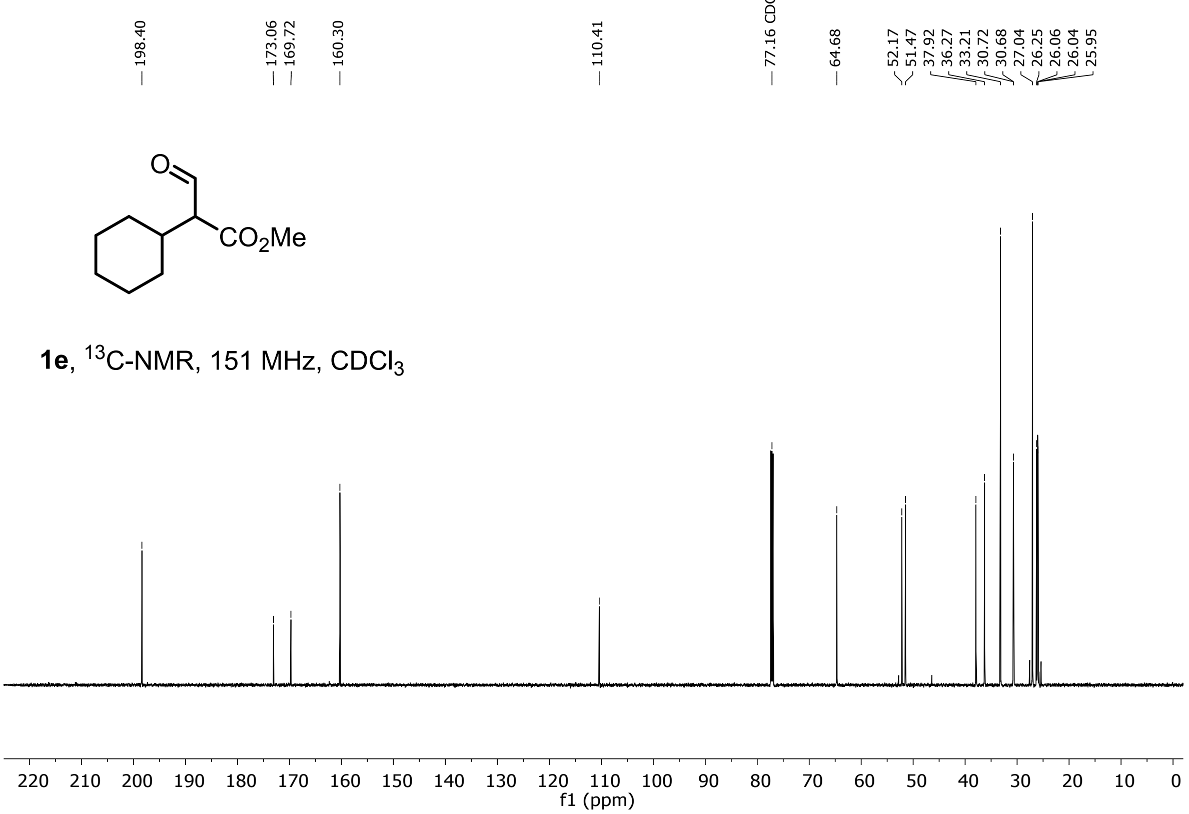

^{13}C NMR (151 MHz, $CDCl_3$) of ***methyl 2-cyclohexyl-3-oxopropanoate (1e).***

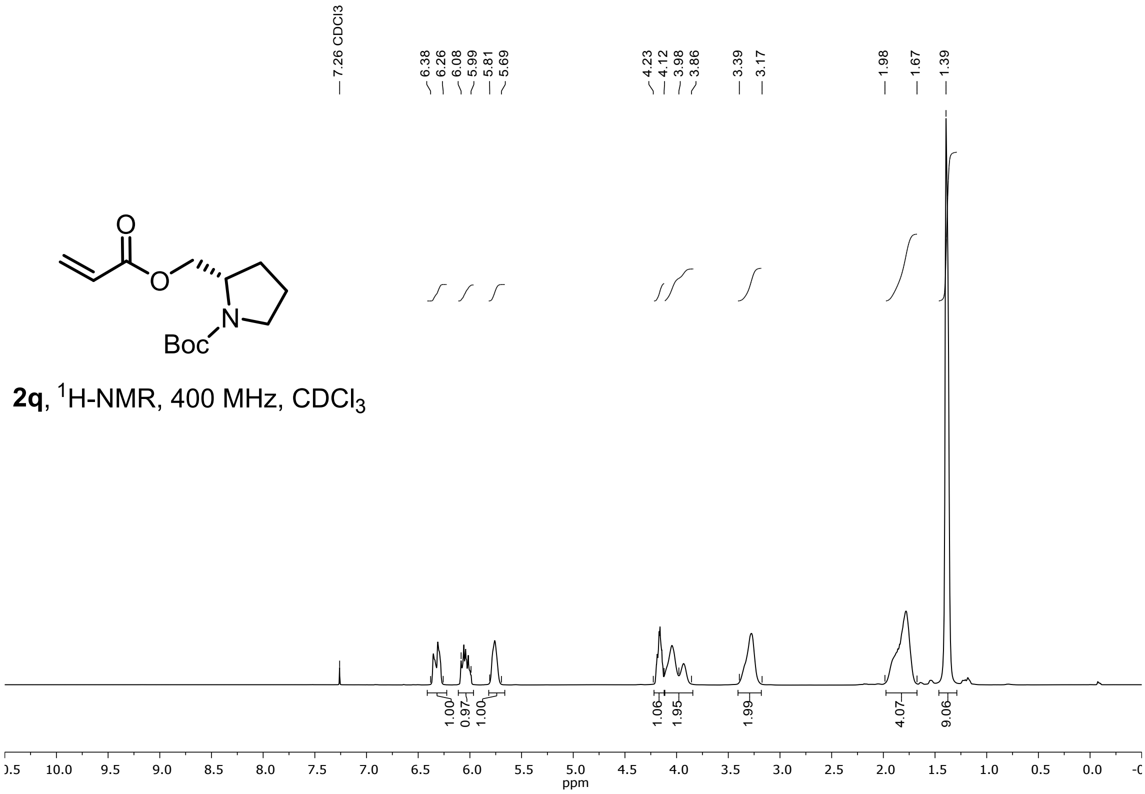

^{1}H NMR (400 MHz, $CDCl_3$) of ***(S)-tert-butyl 2-((acryloyloxy)methyl)pyrrolidine-1-carboxylate (2q).***

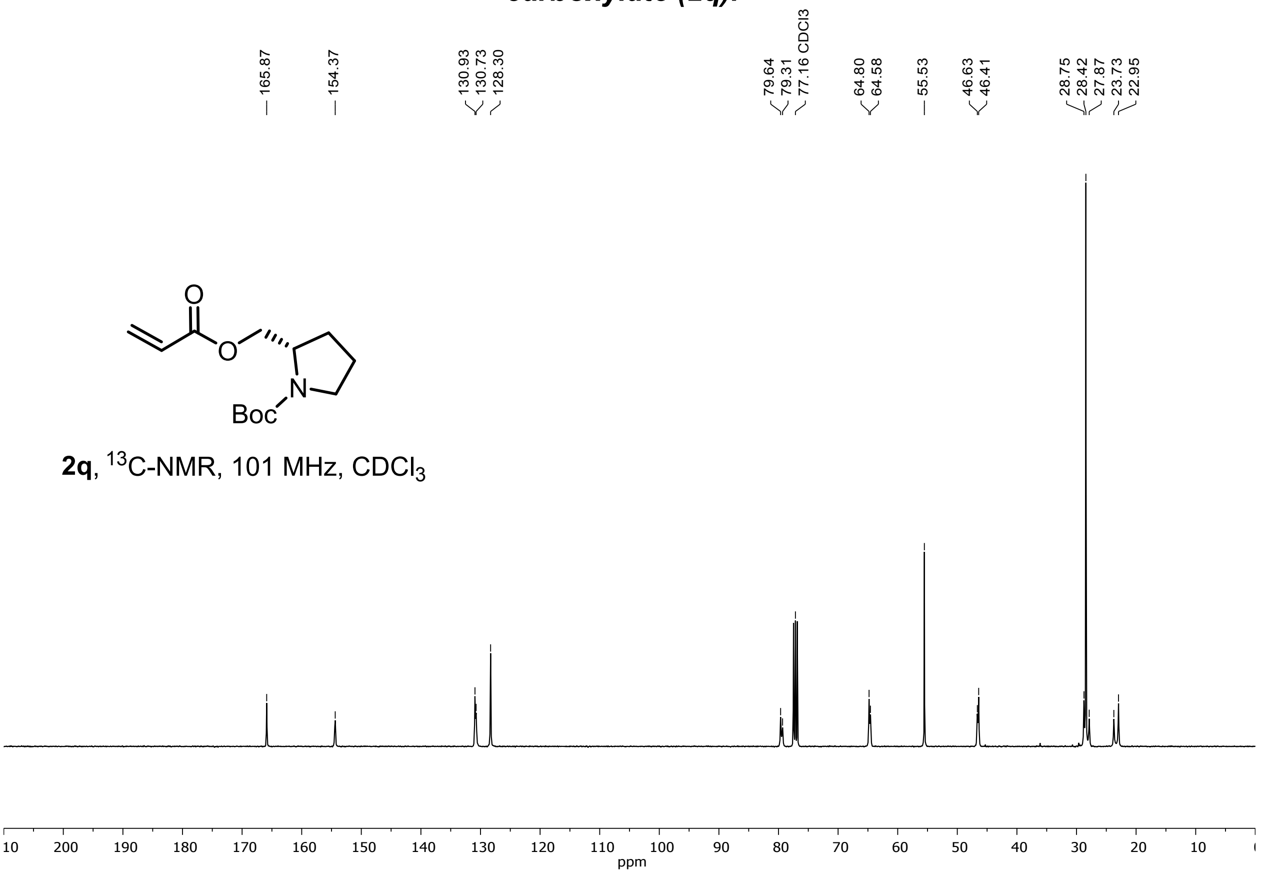

^{13}C NMR (101 MHz, $CDCl_3$) of ***(S)-tert-butyl 2-((acryloyloxy)methyl)pyrrolidine-1-carboxylate (2q).***

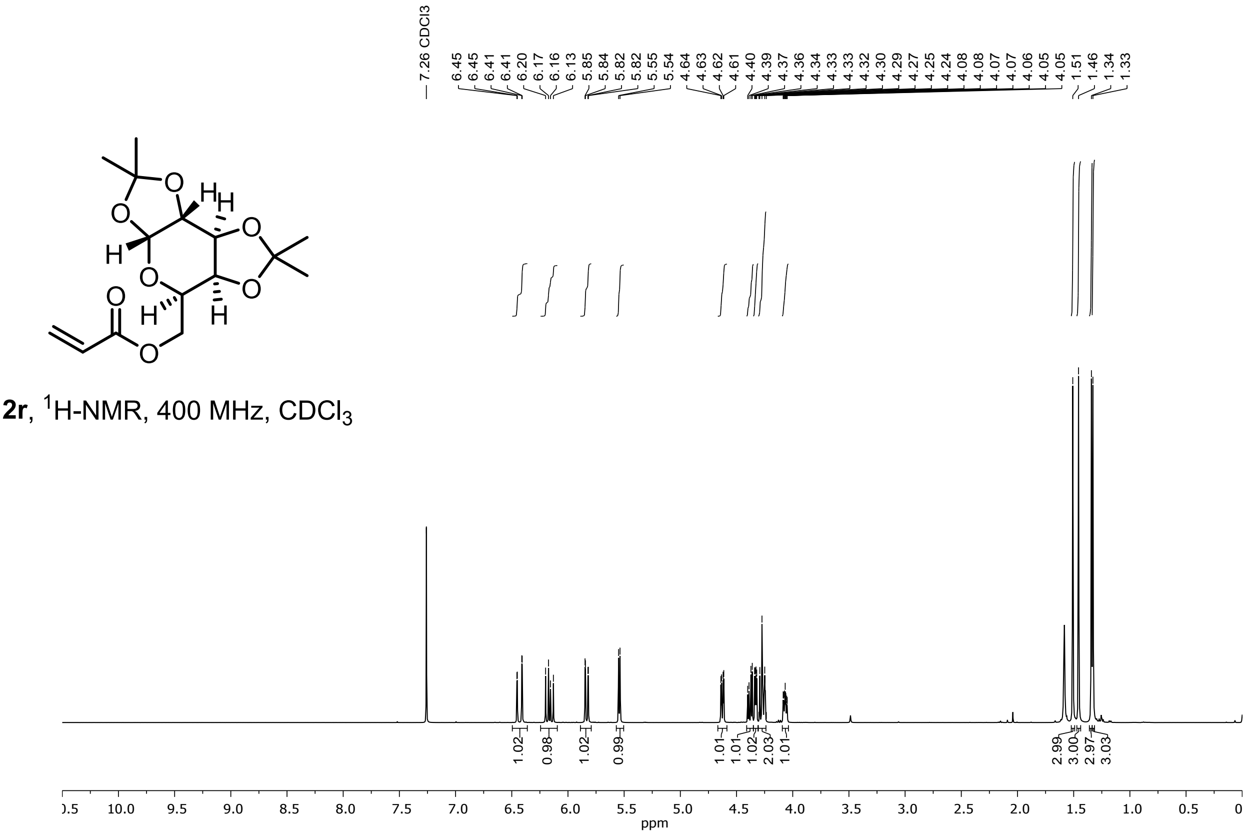

¹H NMR (400 MHz, CDCl$_3$) of ***((3aR,5R,5aS,8aS,8bR)-2,2,7,7-Tetramethyltetrahydro-3aH-bis([1,3]dioxolo)[4,5-b:4',5'-d]pyran-5-yl)methyl acrylate (2r).***

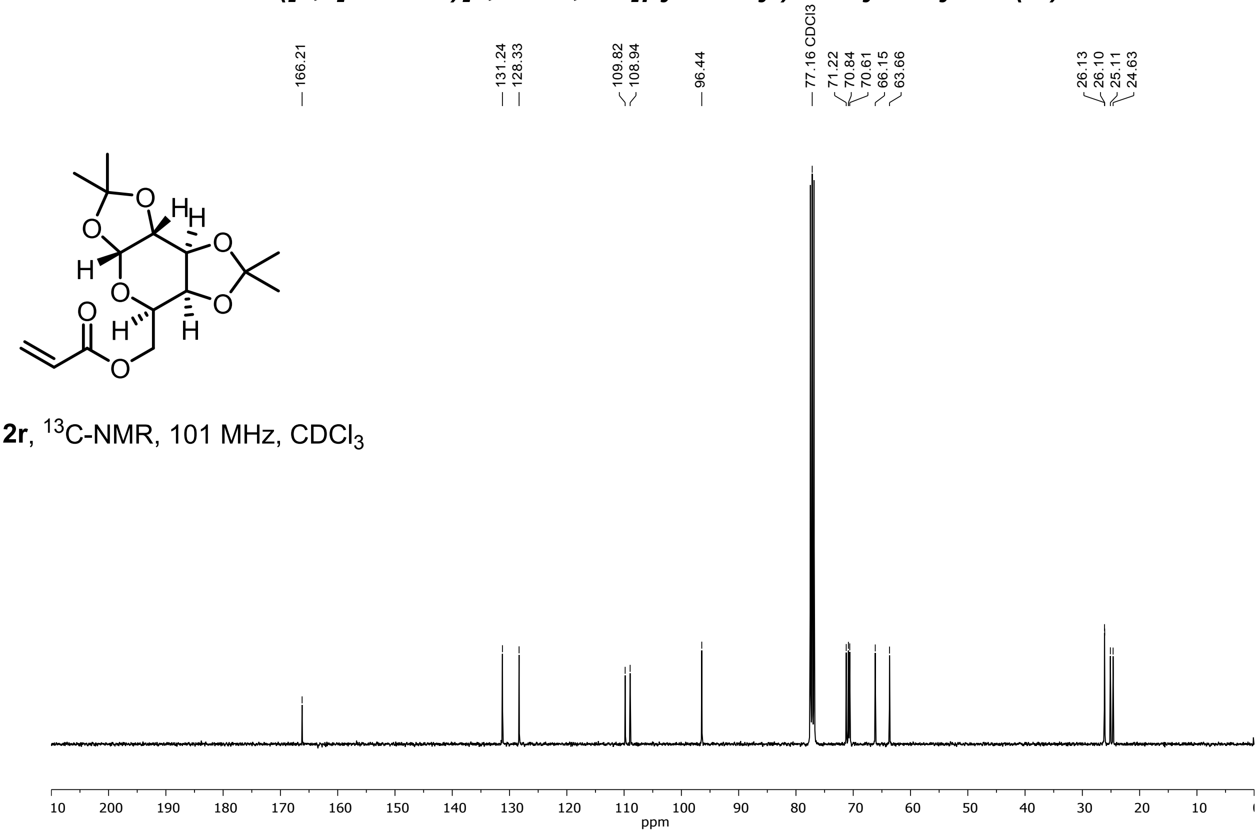

¹³C NMR (101 MHz, CDCl$_3$) of ***((3aR,5R,5aS,8aS,8bR)-2,2,7,7-Tetramethyltetrahydro-3aH-bis([1,3]dioxolo)[4,5-b:4',5'-d]pyran-5-yl)methyl acrylate (2r).***

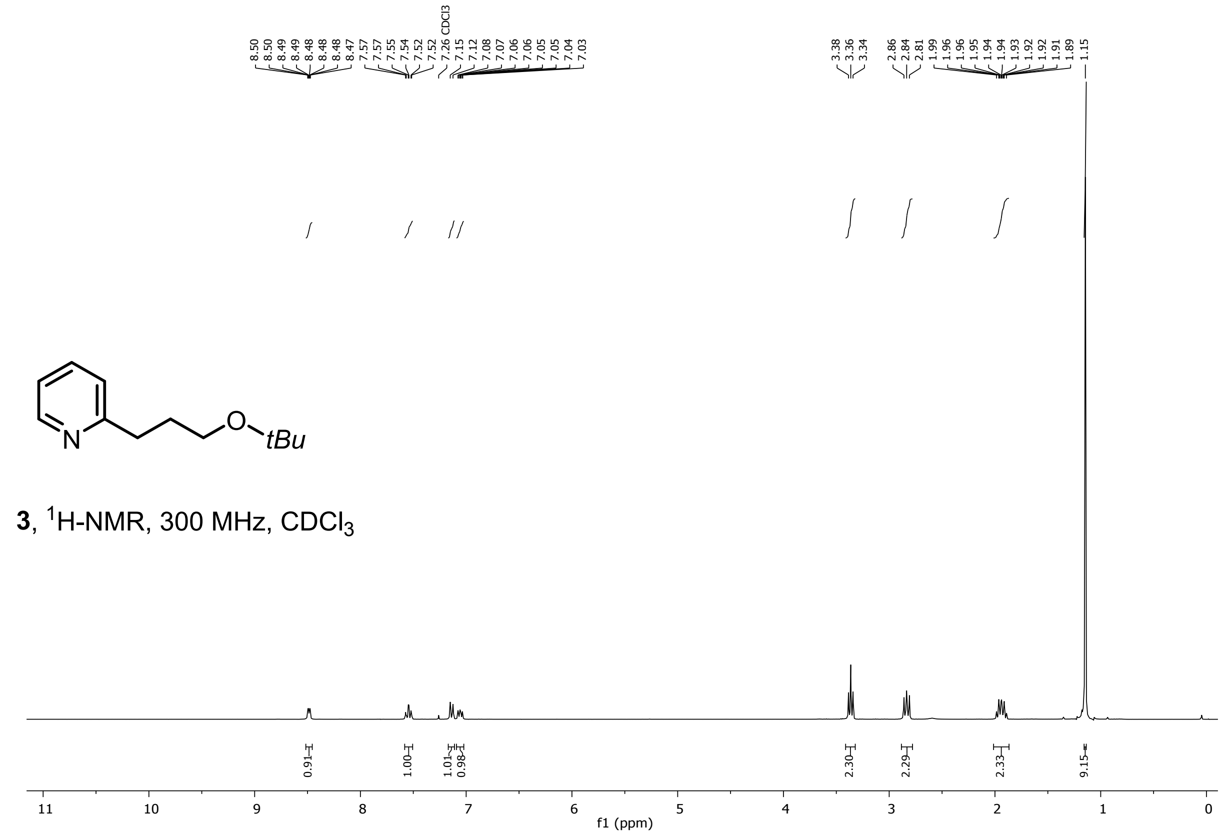

¹H NMR (300 MHz, CDCl₃) of **2-(3-(tert-Butoxy)propyl)pyridine *(3)*.**

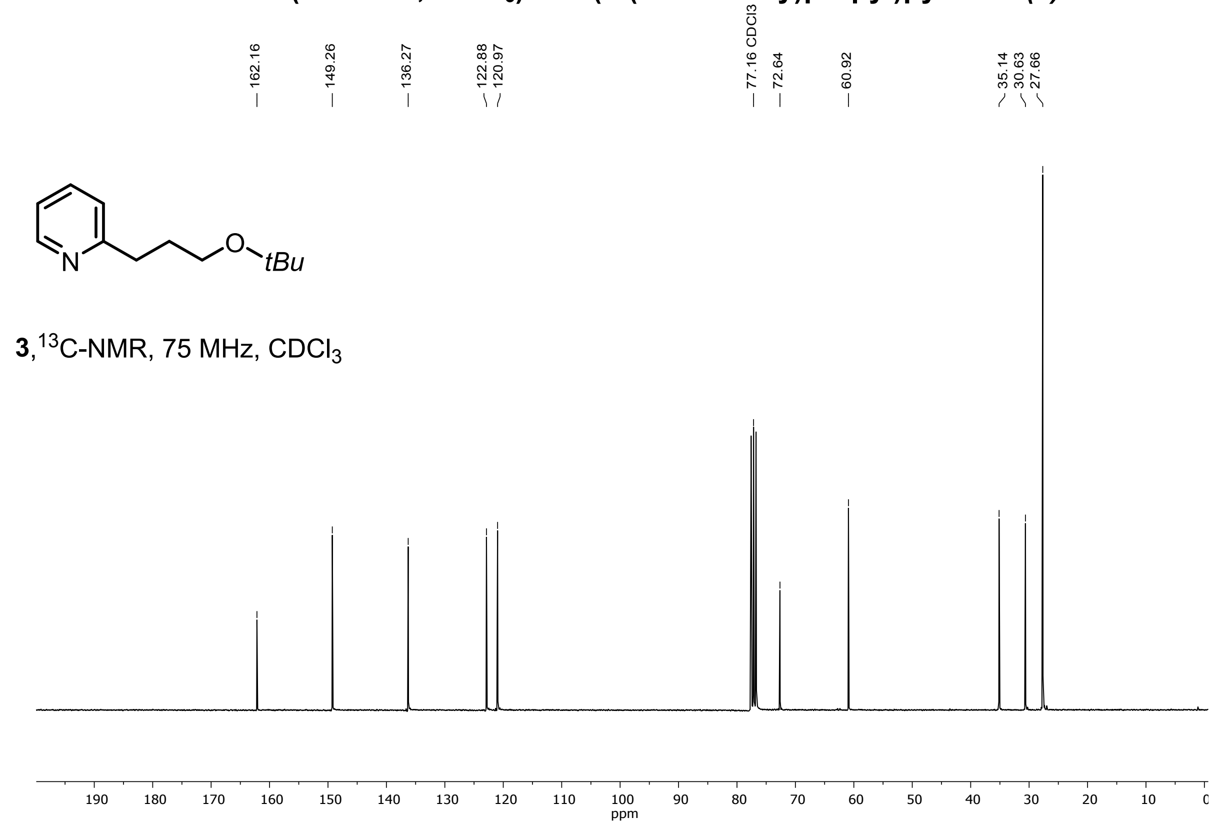

¹³C NMR (75 MHz, CDCl₃) of **2-(3-(tert-Butoxy)propyl)pyridine *(3)***

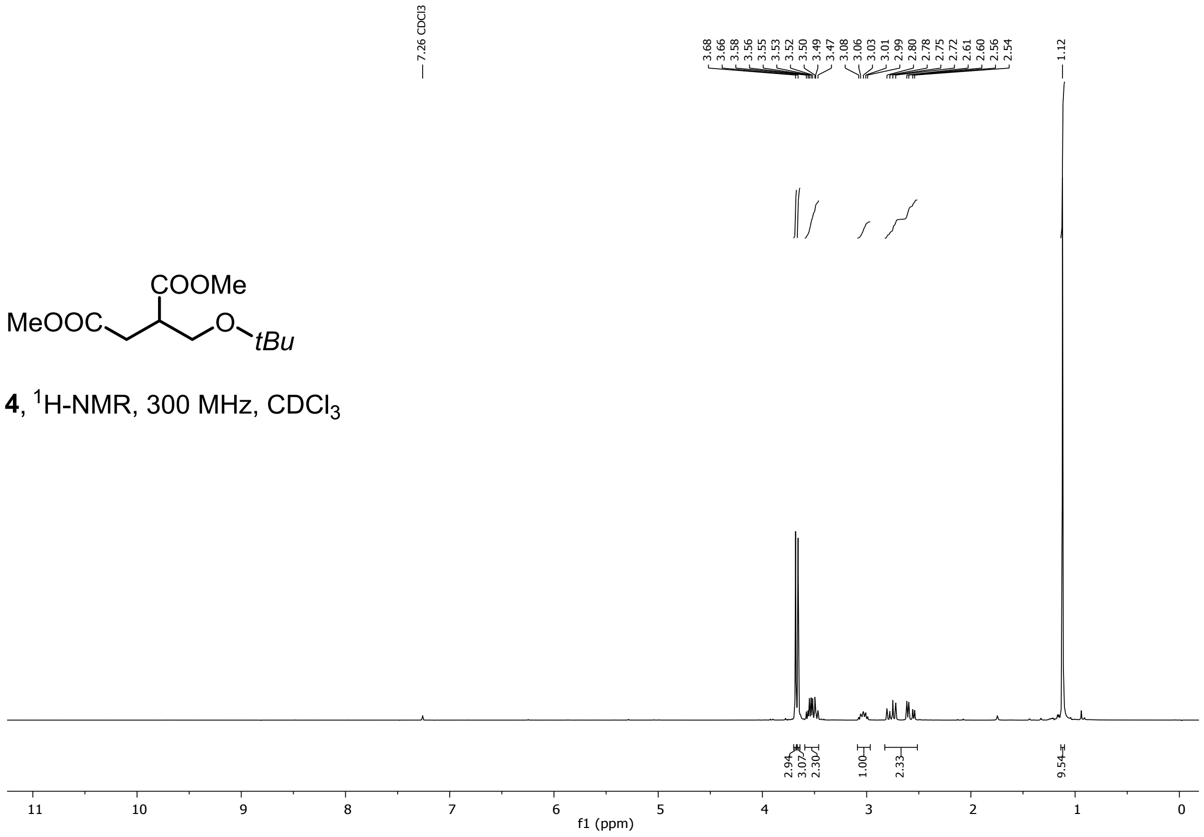

^{1}H NMR (300 MHz, $CDCl_3$) of **Dimethyl-2-(tert-butoxymethyl)succinate *(4)***

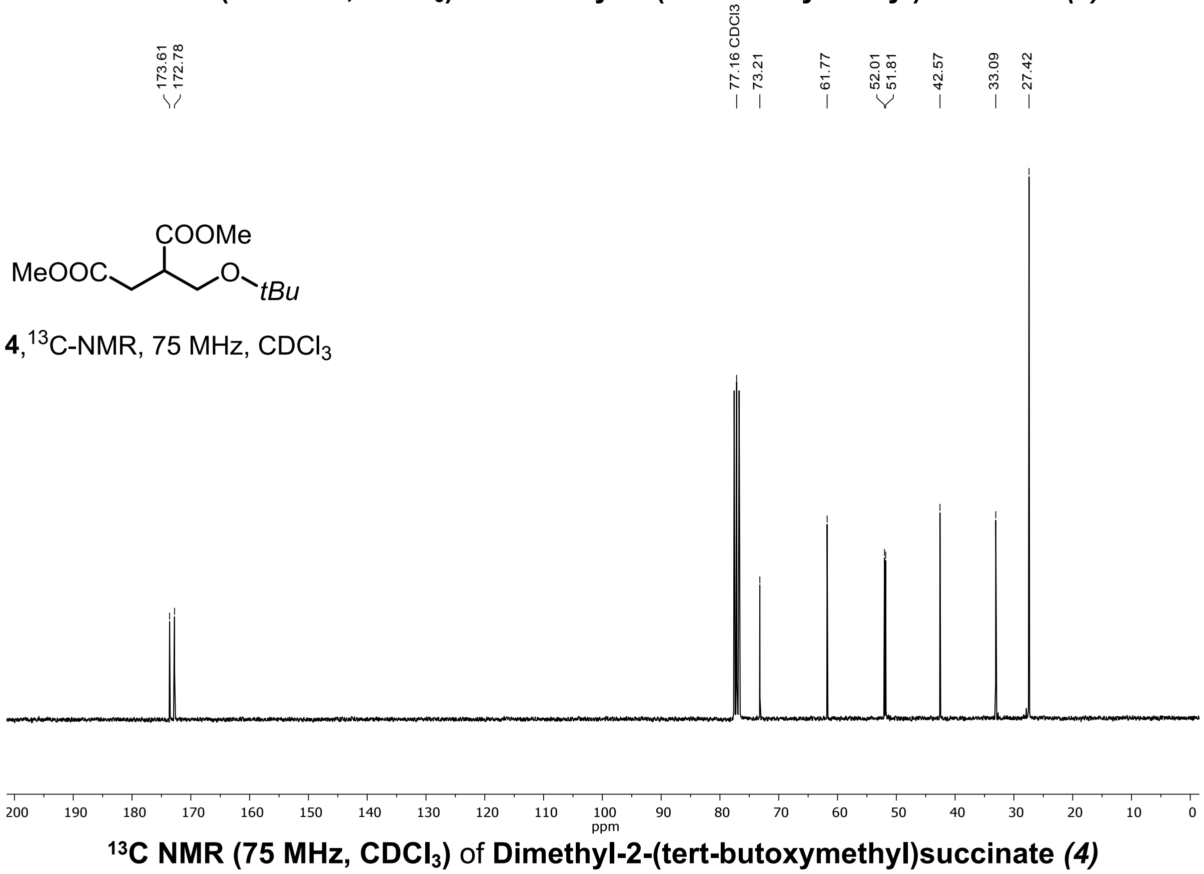

^{13}C NMR (75 MHz, $CDCl_3$) of **Dimethyl-2-(tert-butoxymethyl)succinate *(4)***

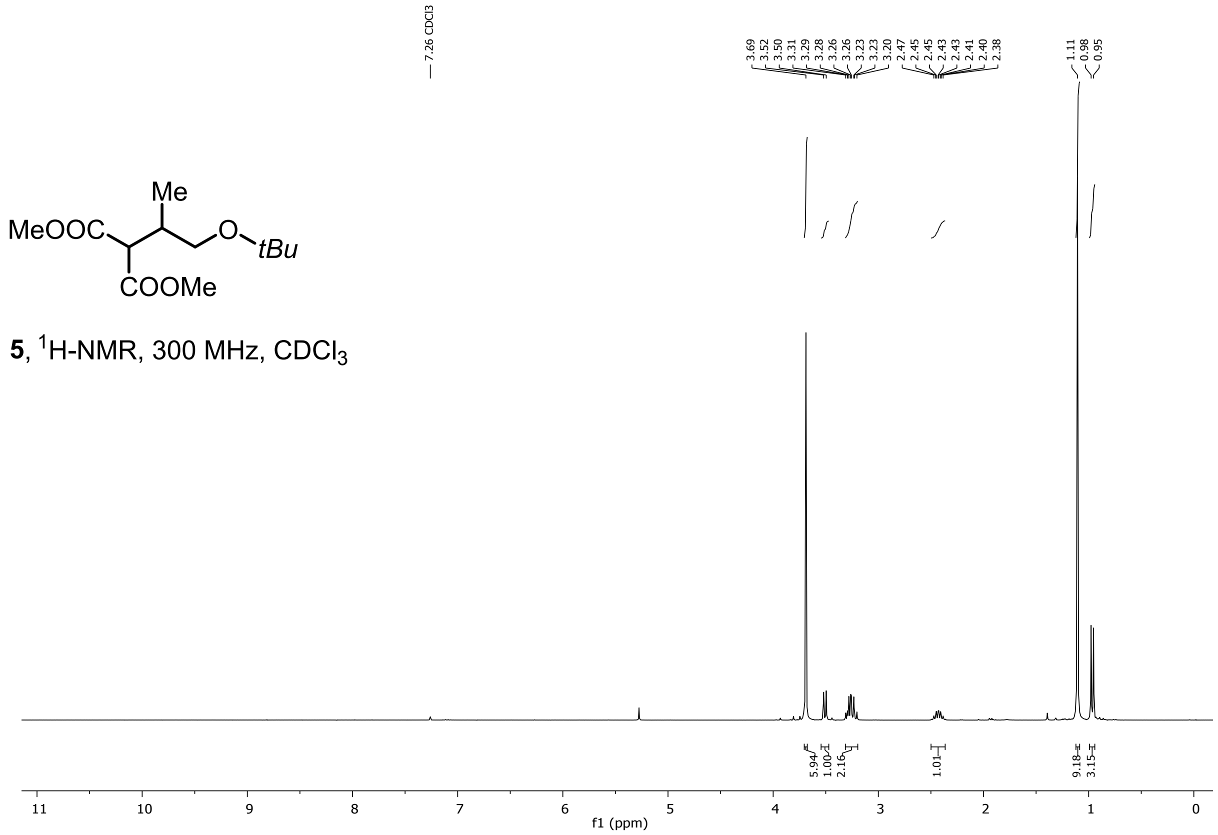

^{1}H NMR (300 MHz, $CDCl_3$) of **dimethyl 2-(1-(tert-butoxy)propan-2-yl)malonate *(5)***

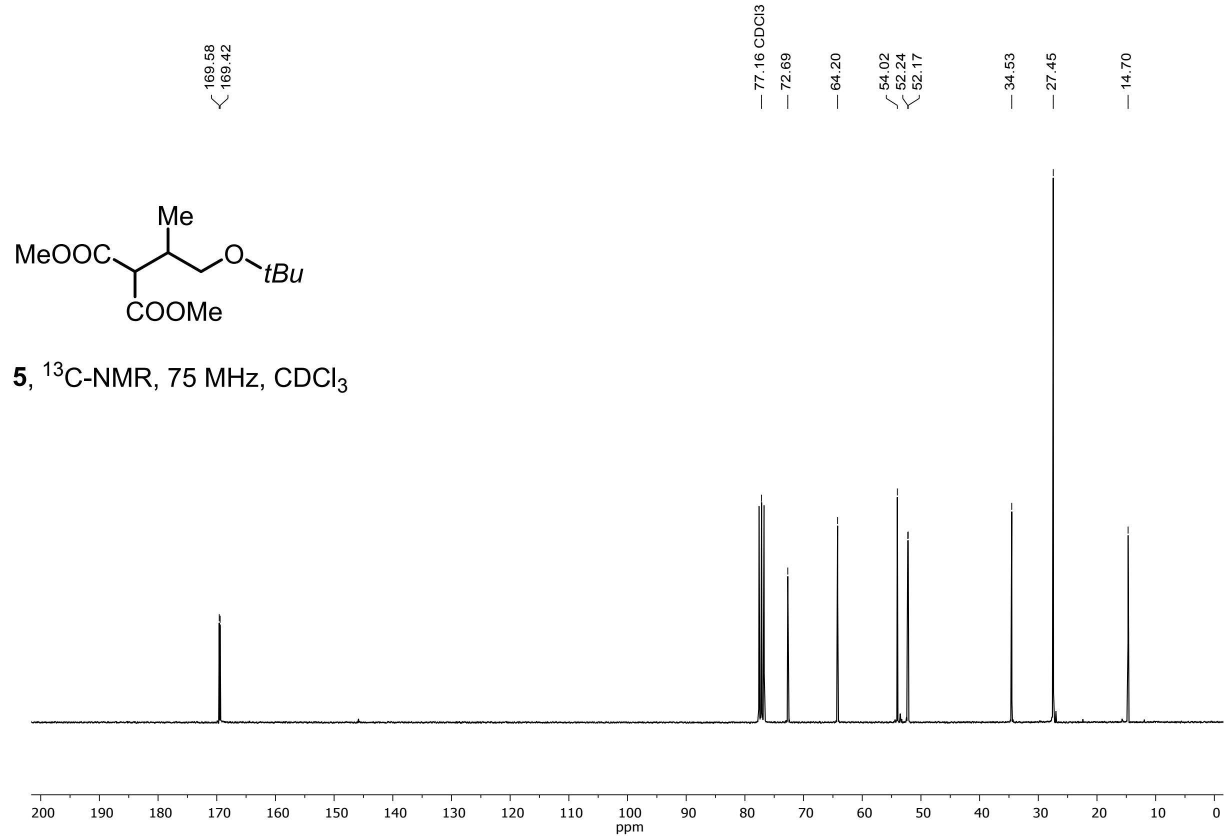

^{13}C NMR (75 MHz, $CDCl_3$) of **dimethyl 2-(1-(tert-butoxy)propan-2-yl)malonate *(5)***

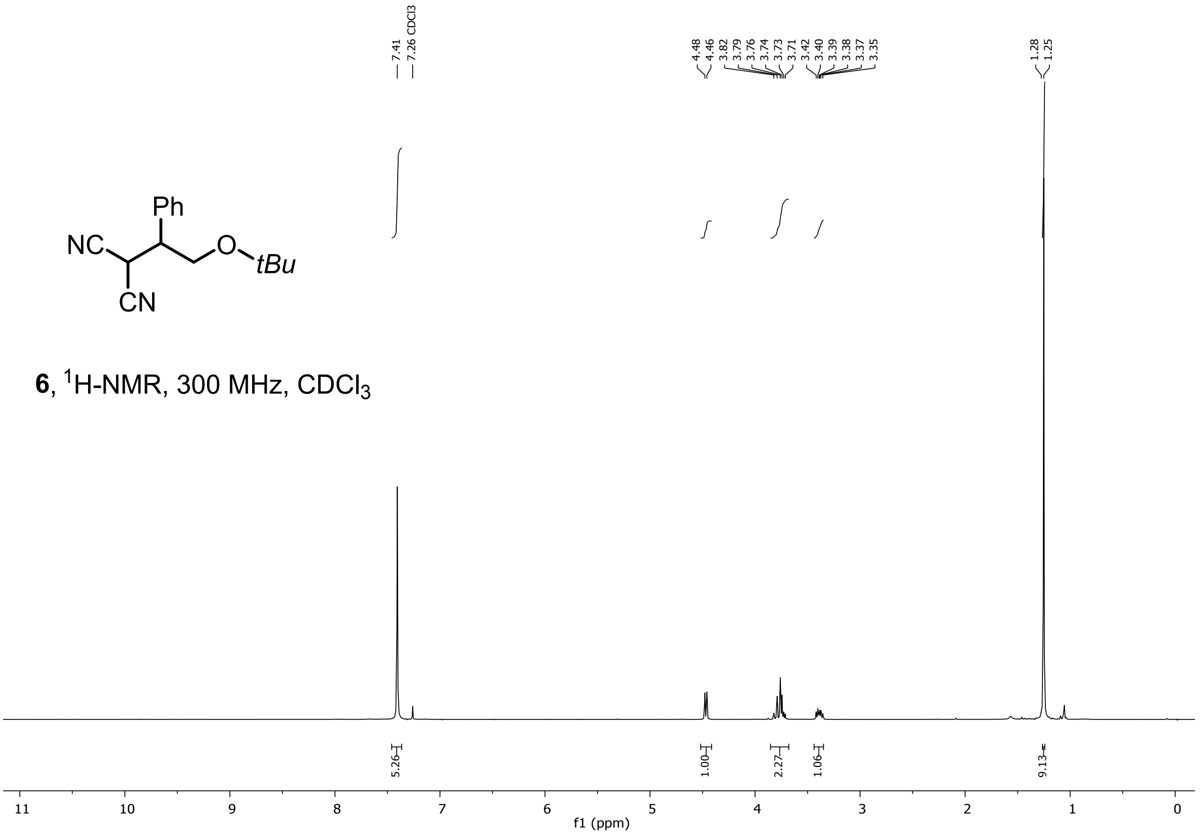

^{1}H NMR (300 MHz, CDCl$_3$) of **2-(2-(tert-Butoxy)-1-phenylethyl)malononitrile *(6)***

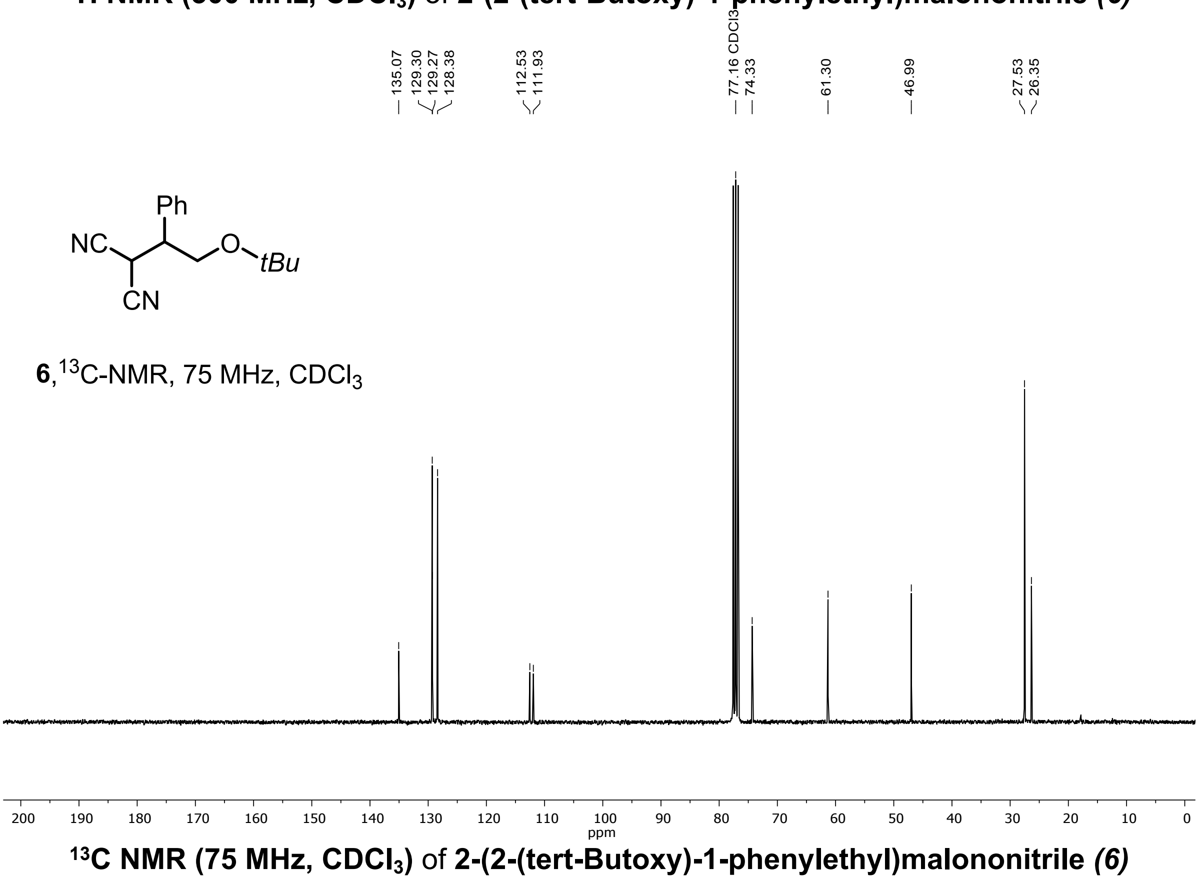

^{13}C NMR (75 MHz, CDCl$_3$) of **2-(2-(tert-Butoxy)-1-phenylethyl)malononitrile *(6)***

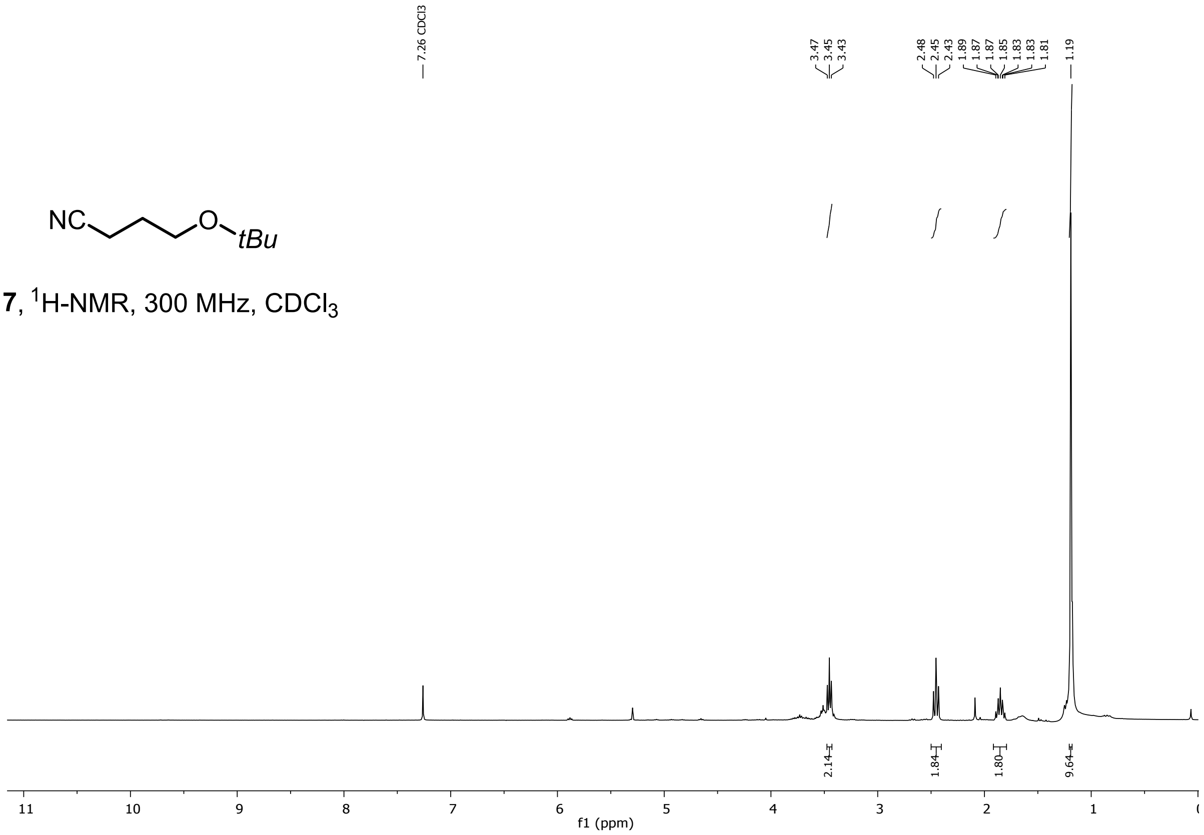

¹H NMR (300 MHz, CDCl₃) of **4-(tert-Butoxy)butanenitrile *(7)***

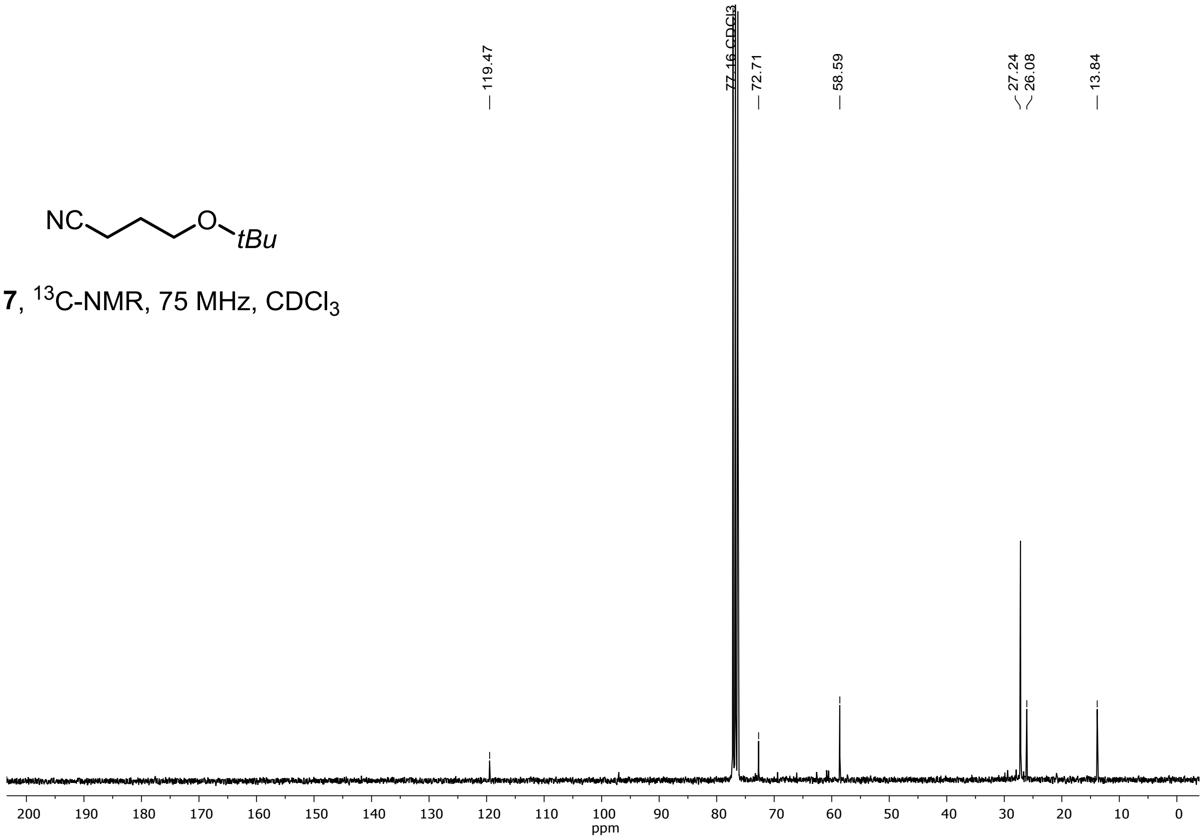

¹³C NMR (75 MHz, CDCl₃) of **4-(tert-Butoxy)butanenitrile *(7)***

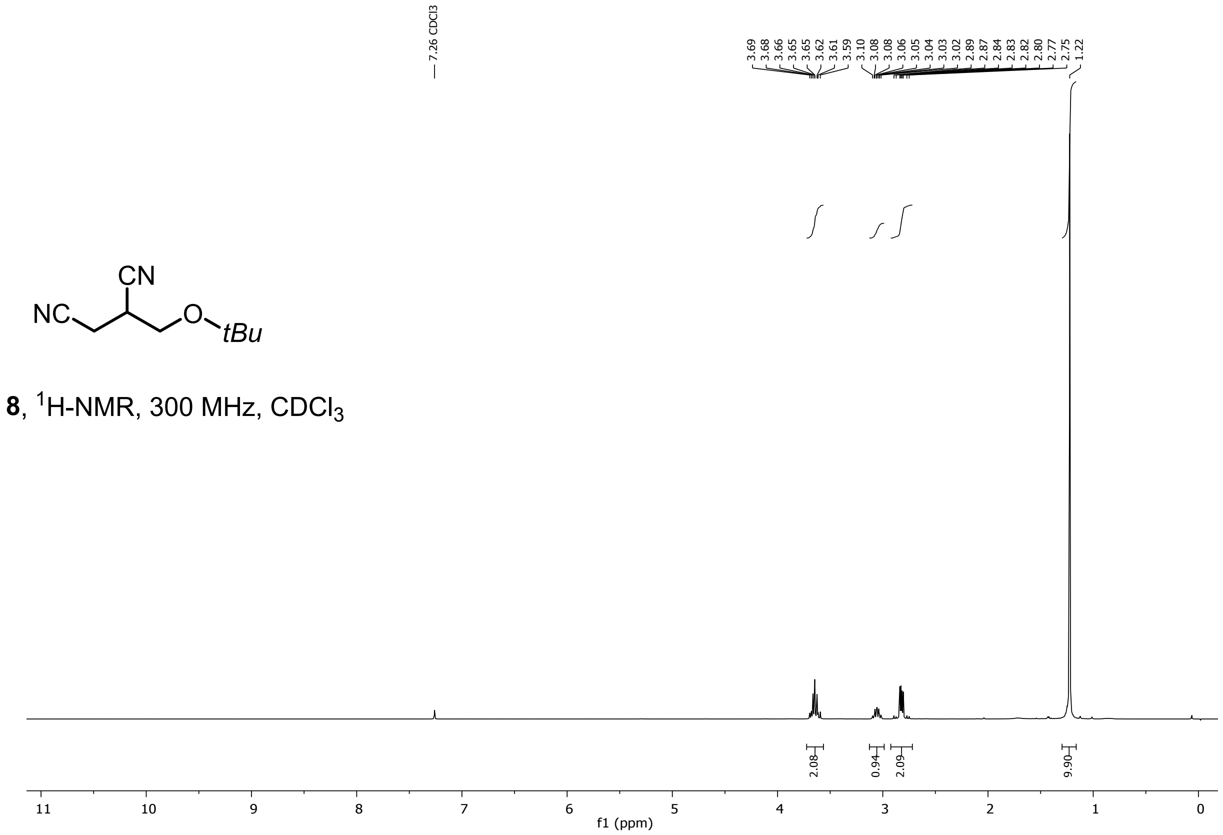

^{1}H NMR (300 MHz, $CDCl_3$) of **2-(tert-Butoxymethyl)succinonitrile *(8)***

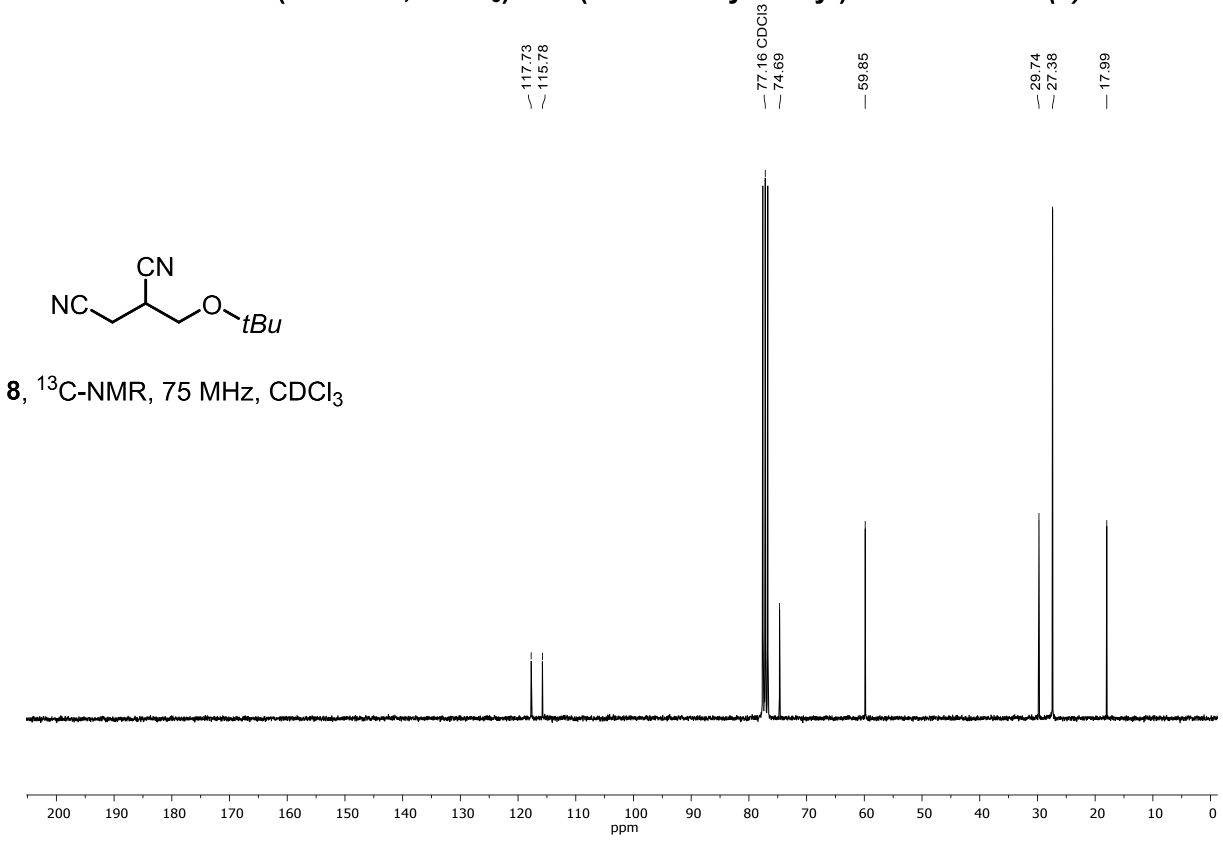

^{13}C NMR (75 MHz, $CDCl_3$) of **2-(tert-Butoxymethyl)succinonitrile *(8)***

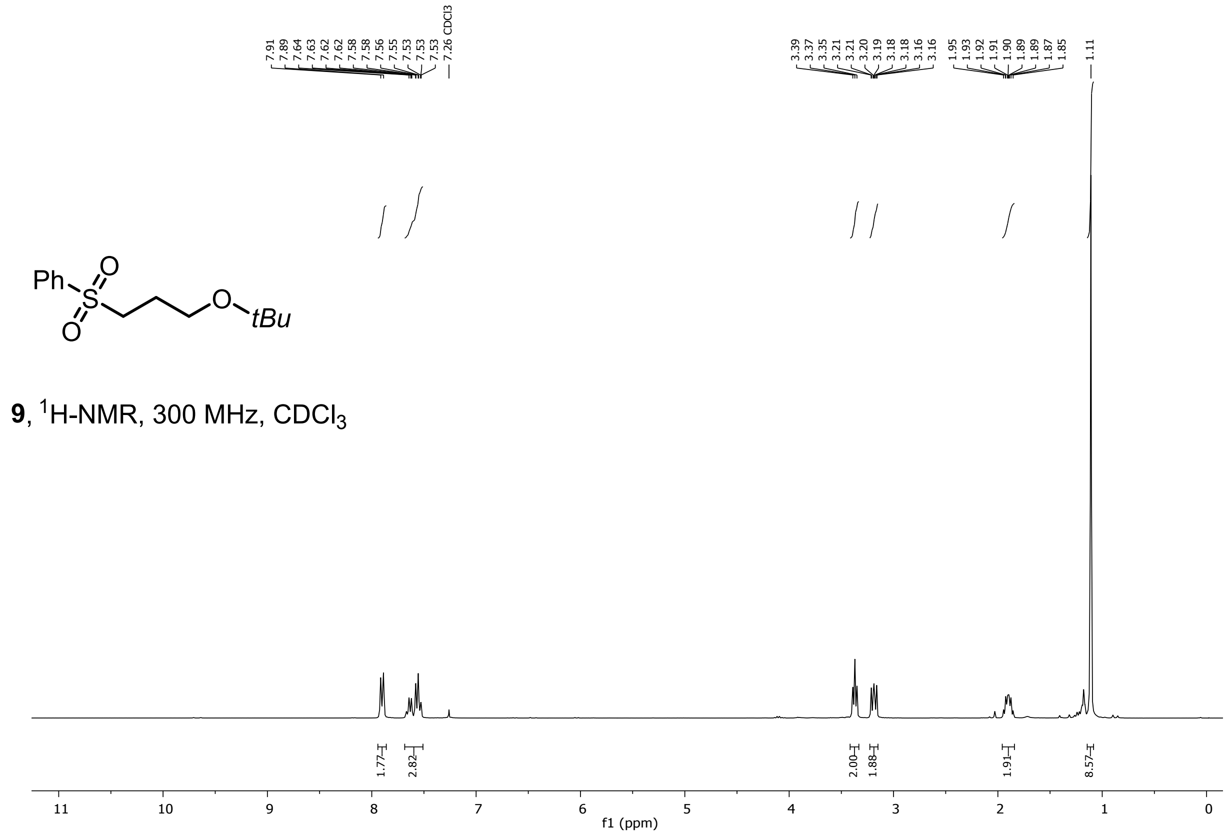

^{1}H NMR (300 MHz, $CDCl_3$) of **((3-(tert-Butoxy)propyl)sulfonyl)benzene** ***(9)***

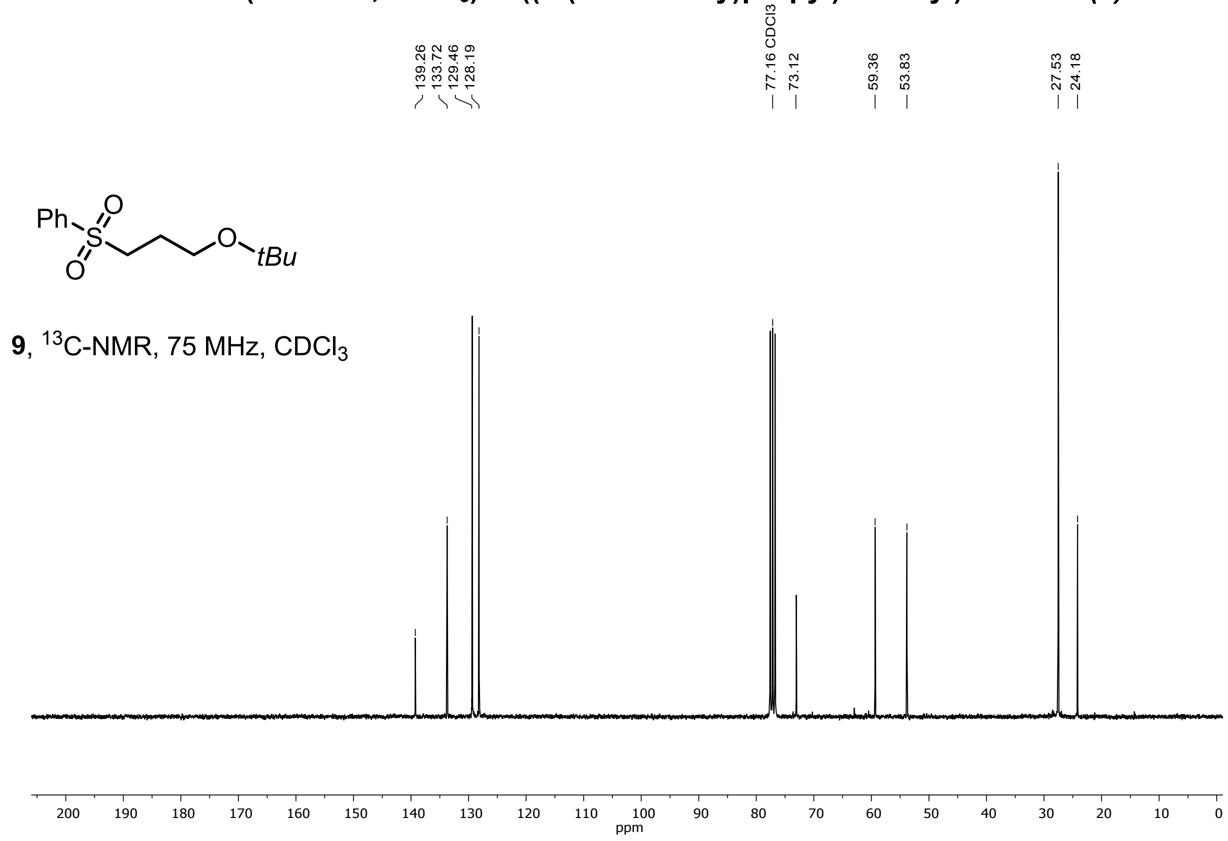

^{13}C NMR (75 MHz, $CDCl_3$) of **((3-(tert-Butoxy)propyl)sulfonyl)benzene** ***(9)***

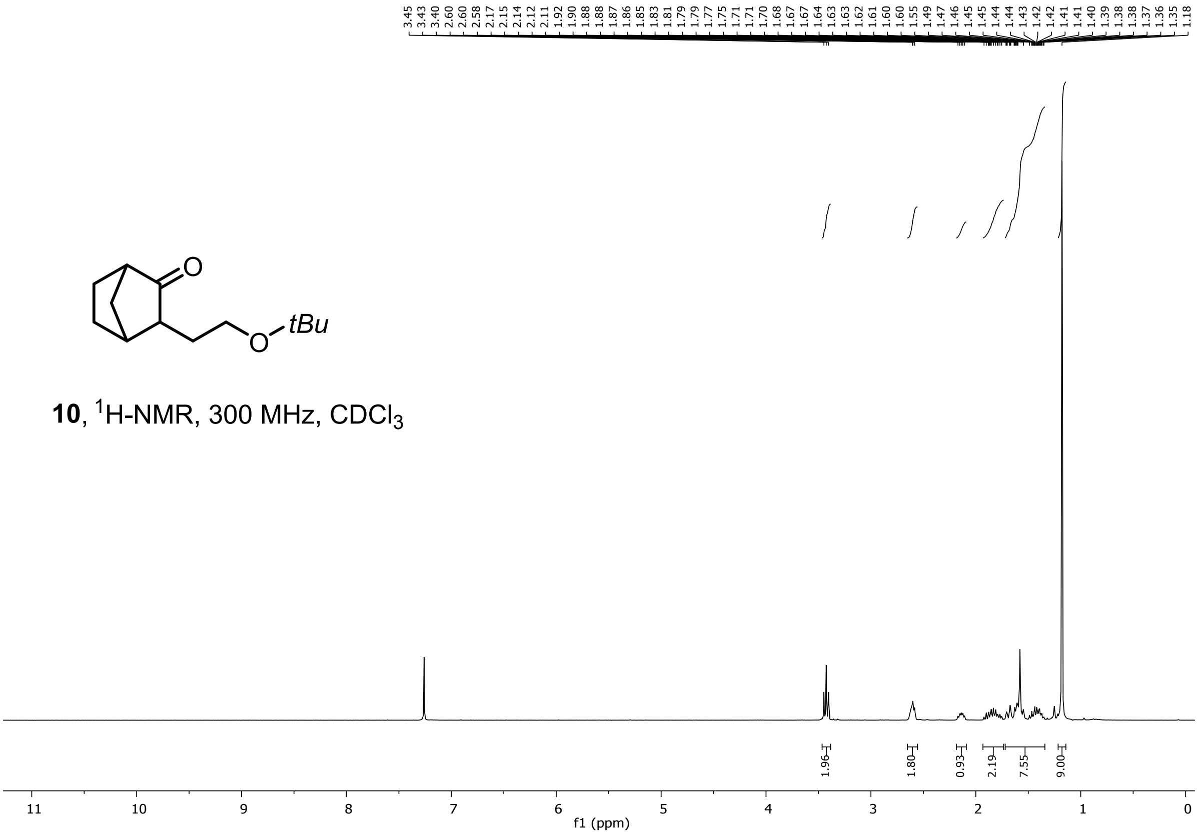

^{1}H NMR (300 MHz, $CDCl_3$) of **3-(2-(tert-Butoxy)ethyl)bicyclo[2.2.1]heptan-2-one *(10)***

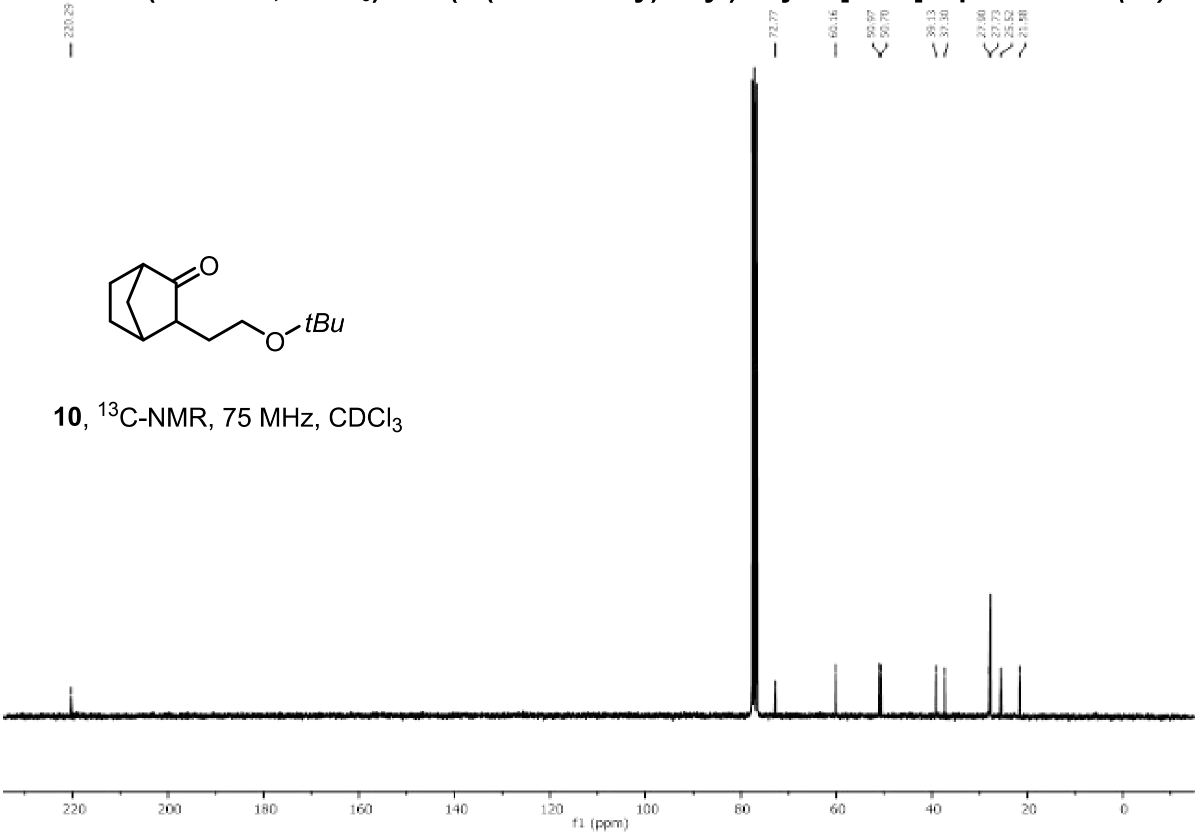

^{13}C NMR (75 MHz, $CDCl_3$) of **3-(2-(tert-Butoxy)ethyl)bicyclo[2.2.1]heptan-2-one *(10)***

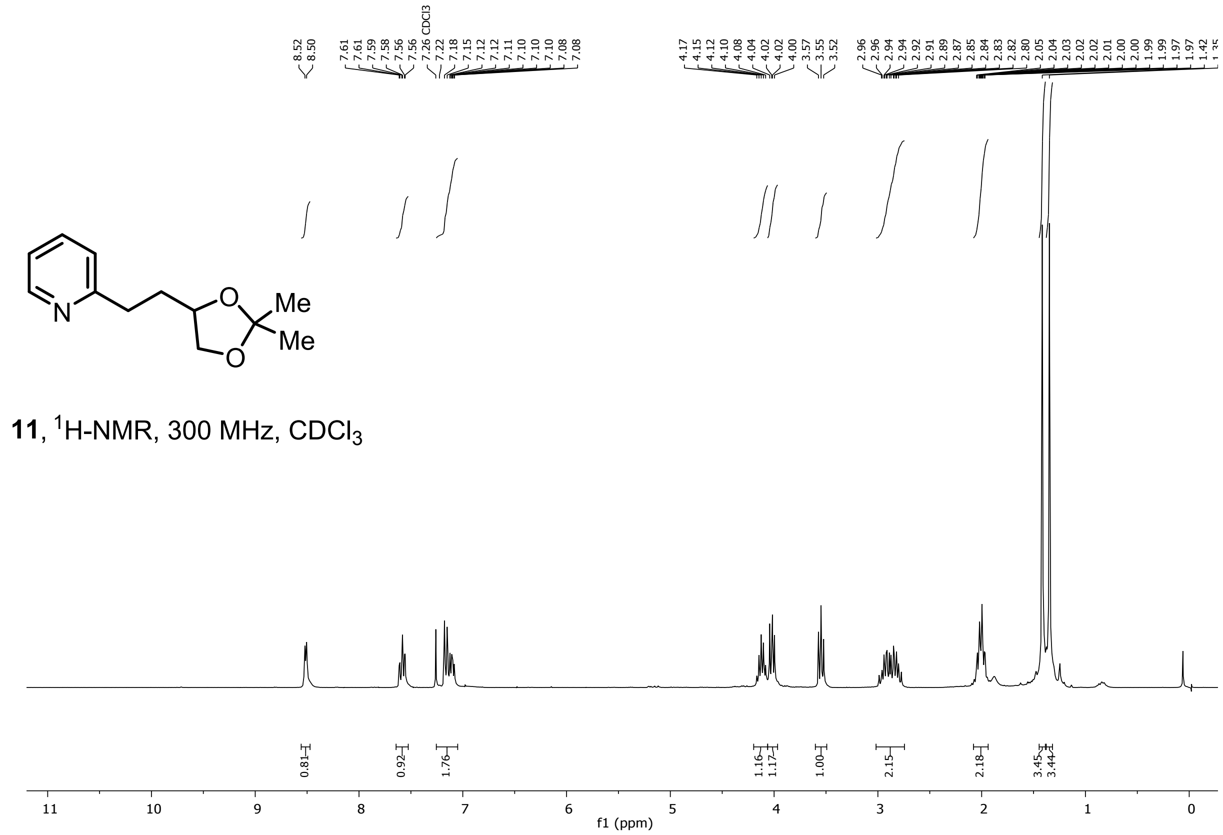

^{1}H NMR (300 MHz, $CDCl_3$) of **2-(2-(2,2-Dimethyl-1,3-dioxolan-4-yl)ethyl)pyridine (11)**

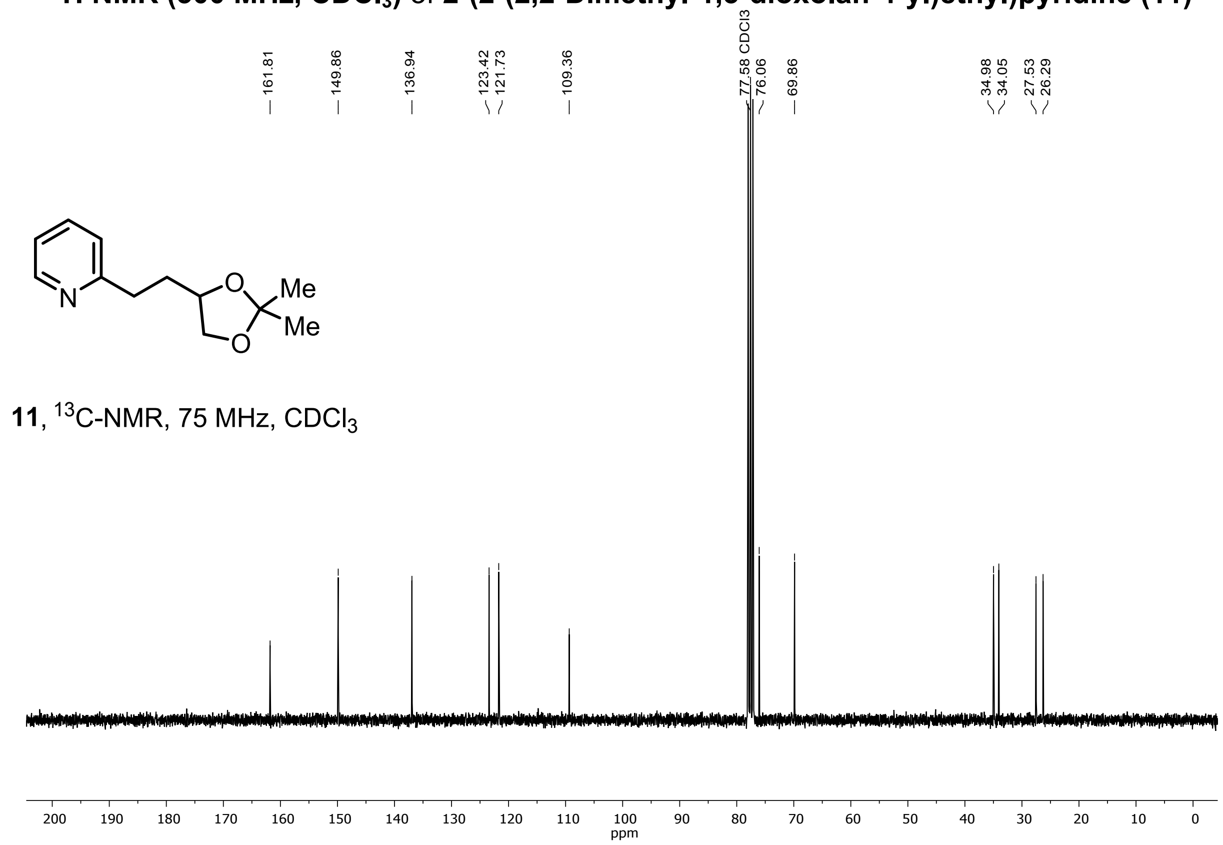

^{13}C NMR (75 MHz, $CDCl_3$) of **2-(2-(2,2-Dimethyl-1,3-dioxolan-4-yl)ethyl)pyridine (11)**

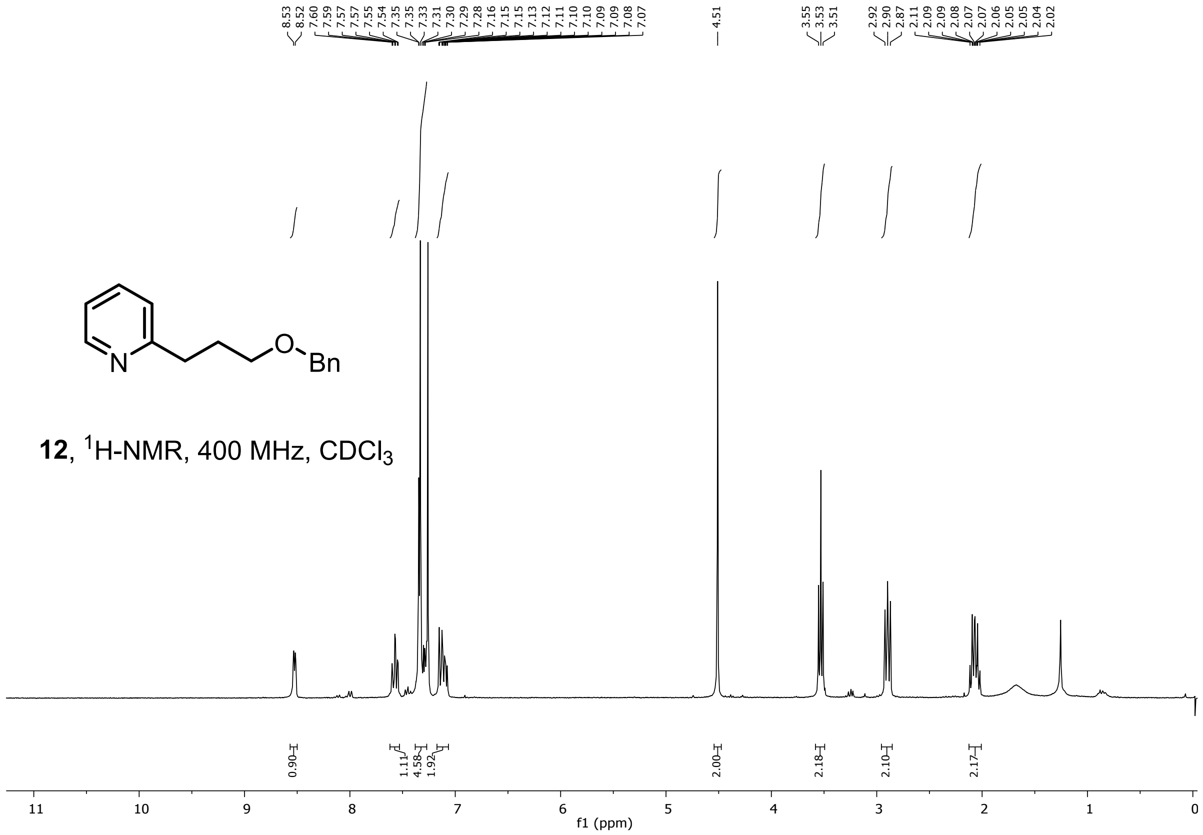

^{1}H NMR (400 MHz, $CDCl_3$) of **2-(3-(Benzyloxy)propyl)pyridine *(12)***

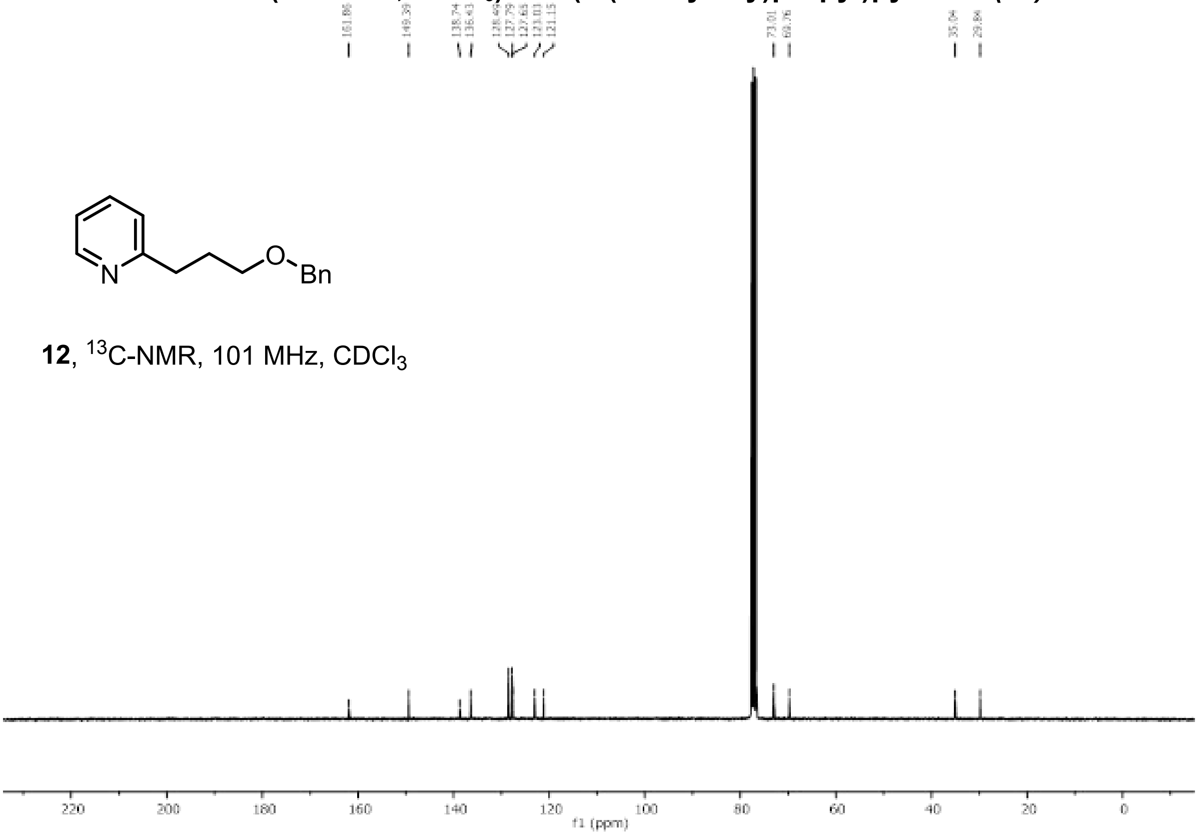

^{13}C NMR (101 MHz, $CDCl_3$) of **2-(3-(Benzyloxy)propyl)pyridine *(12)***

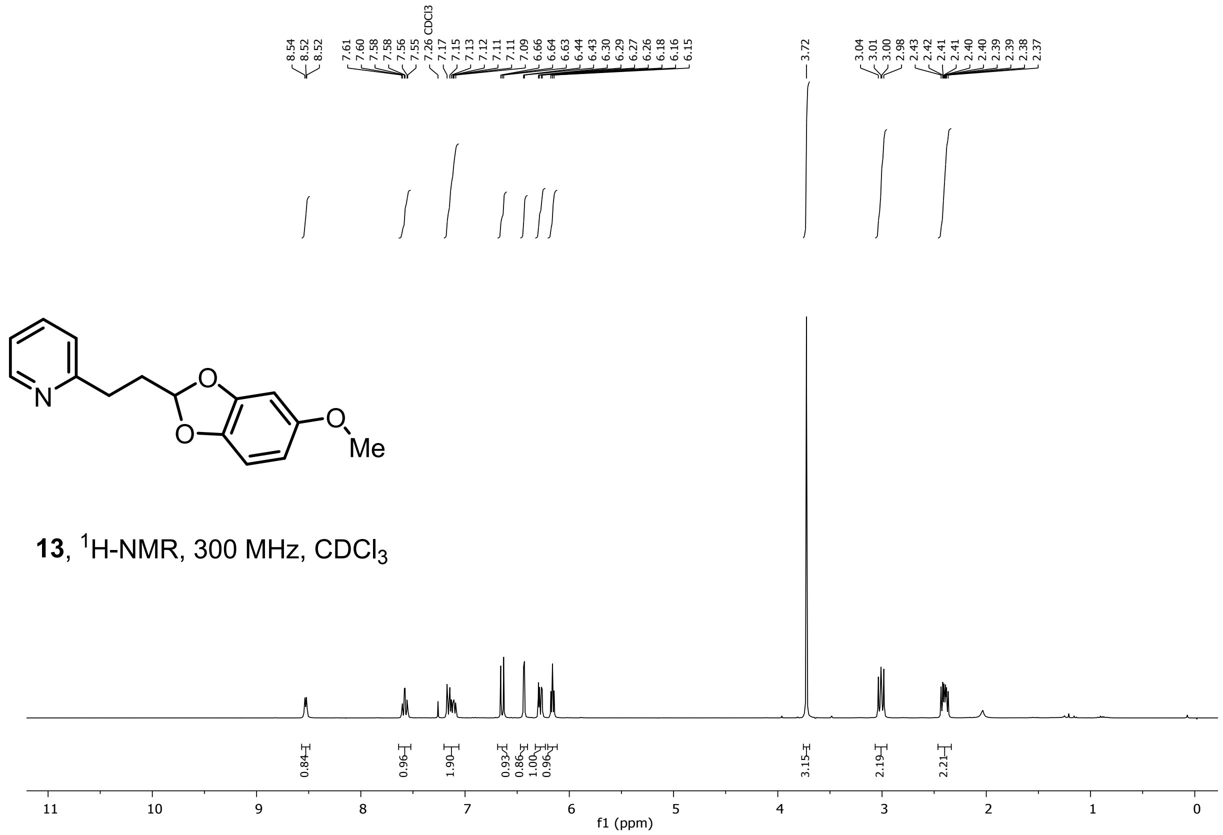

^{1}H NMR (300 MHz, $CDCl_3$) of **2-(2-(5-Methoxybenzo[1,3]dioxol-2-yl)ethyl)pyridine *(13)***

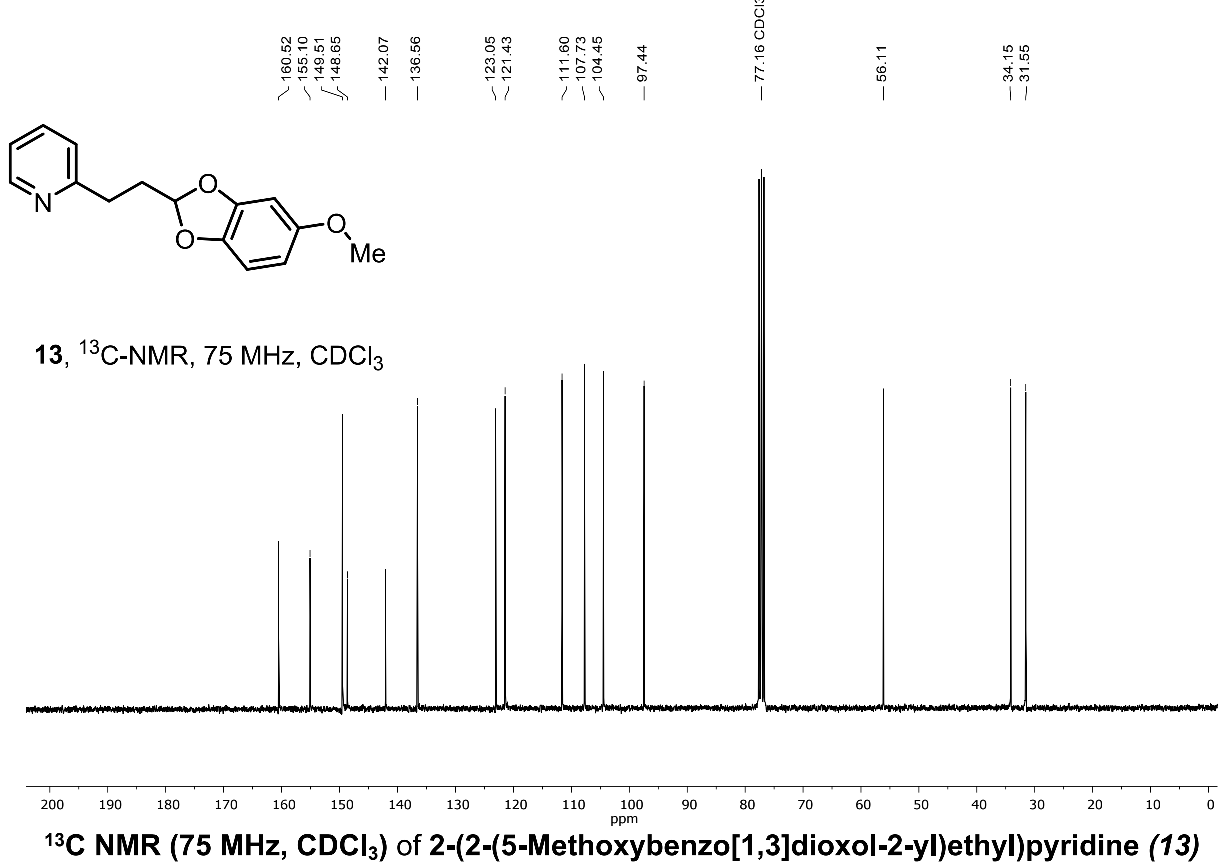

^{13}C NMR (75 MHz, $CDCl_3$) of **2-(2-(5-Methoxybenzo[1,3]dioxol-2-yl)ethyl)pyridine *(13)***

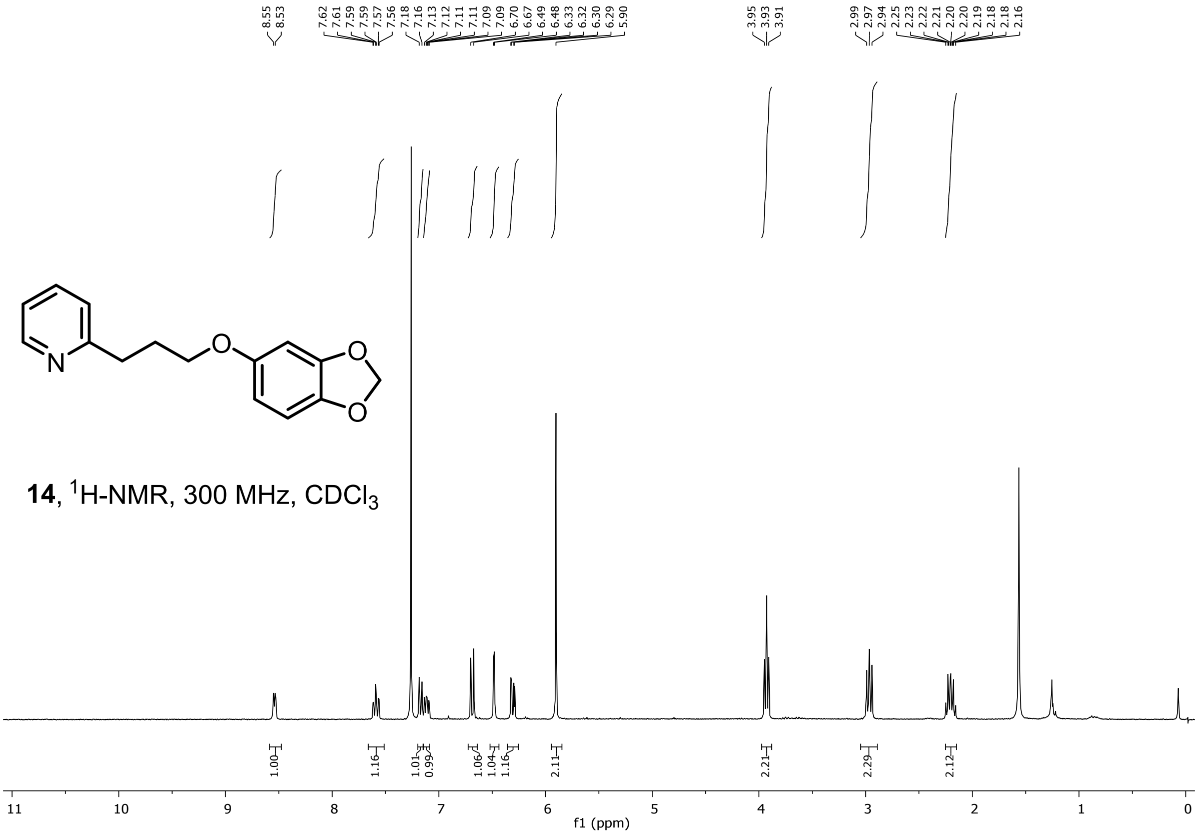

^{1}H NMR (300 MHz, $CDCl_3$) of **2-(3-(Benzo[d][1,3]dioxol-5-yloxy)propyl)pyridine *(14)***

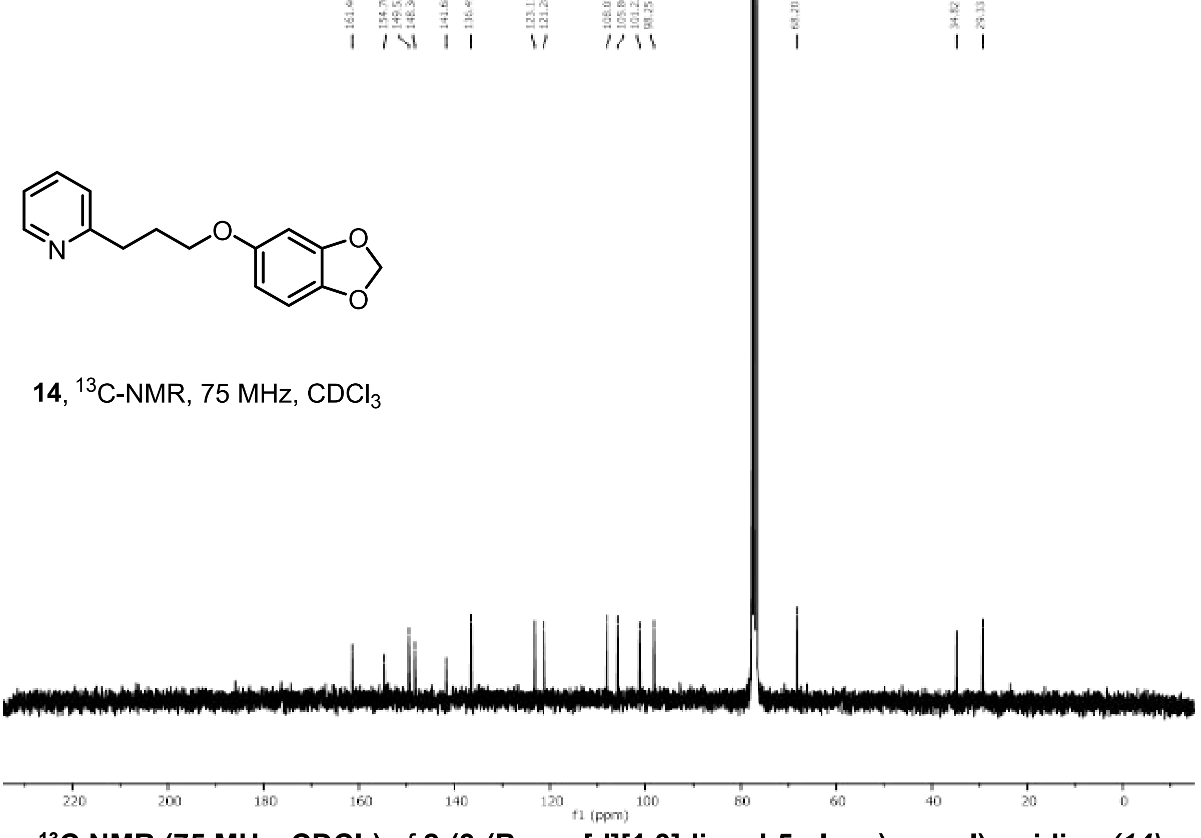

^{13}C NMR (75 MHz, $CDCl_3$) of **2-(3-(Benzo[d][1,3]dioxol-5-yloxy)propyl)pyridine *(14)***

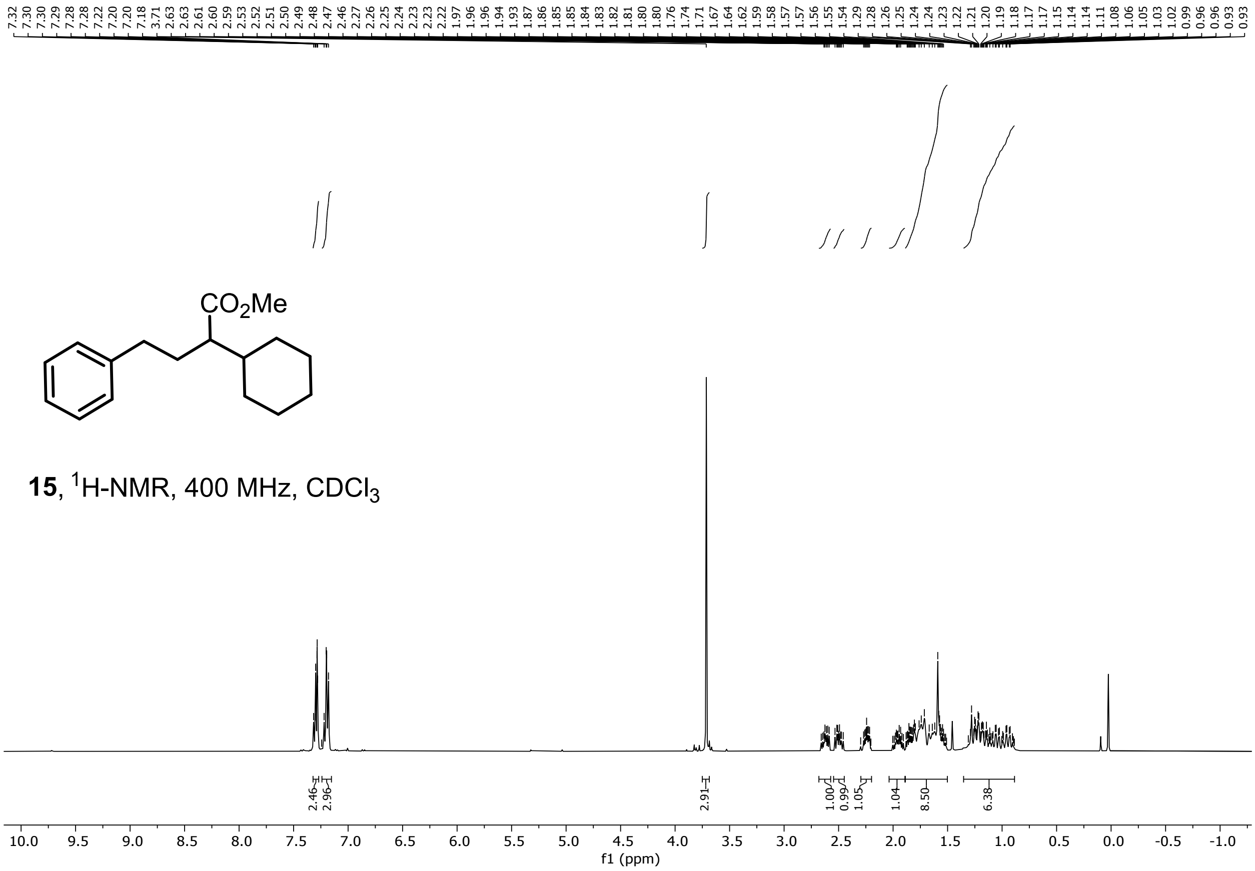

^{1}H NMR (400 MHz, CDCl$_3$) of ***methyl 2-cyclohexyl-4-phenylbutanoate (15).***

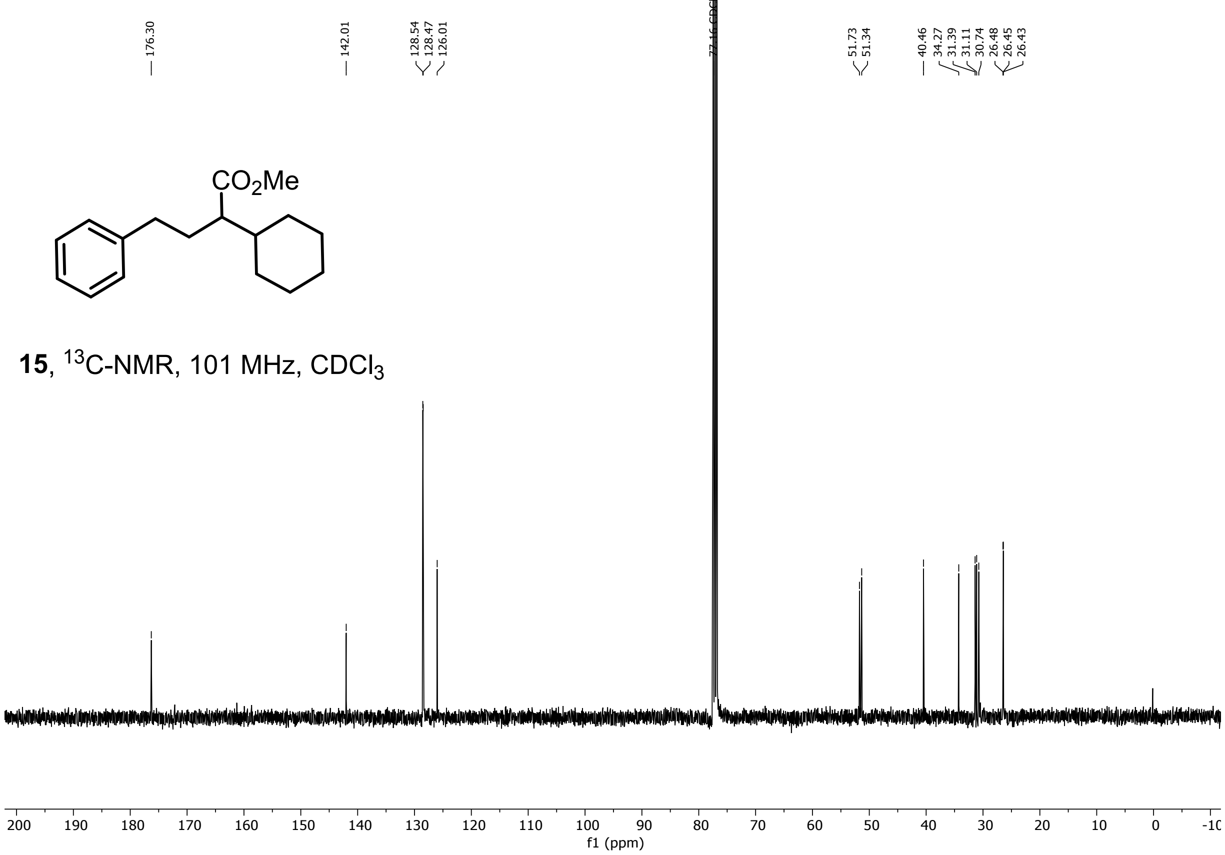

^{13}C NMR (101 MHz, CDCl$_3$) of ***methyl 2-cyclohexyl-4-phenylbutanoate (15).***

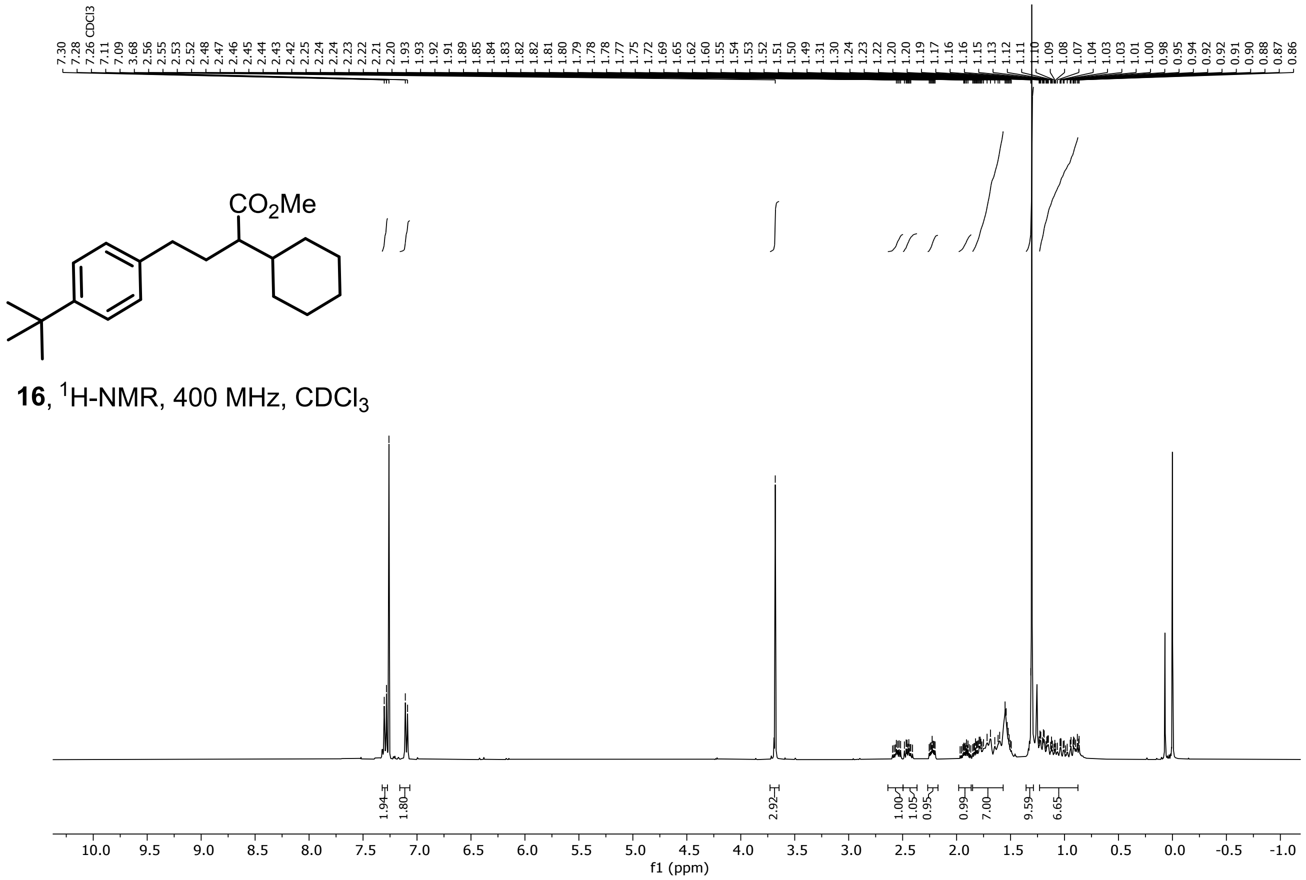

^{1}H NMR (400 MHz, $CDCl_3$) of ***methyl 4-(4-(tert-butyl)phenyl)-2-cyclohexylbutanoate (16).***

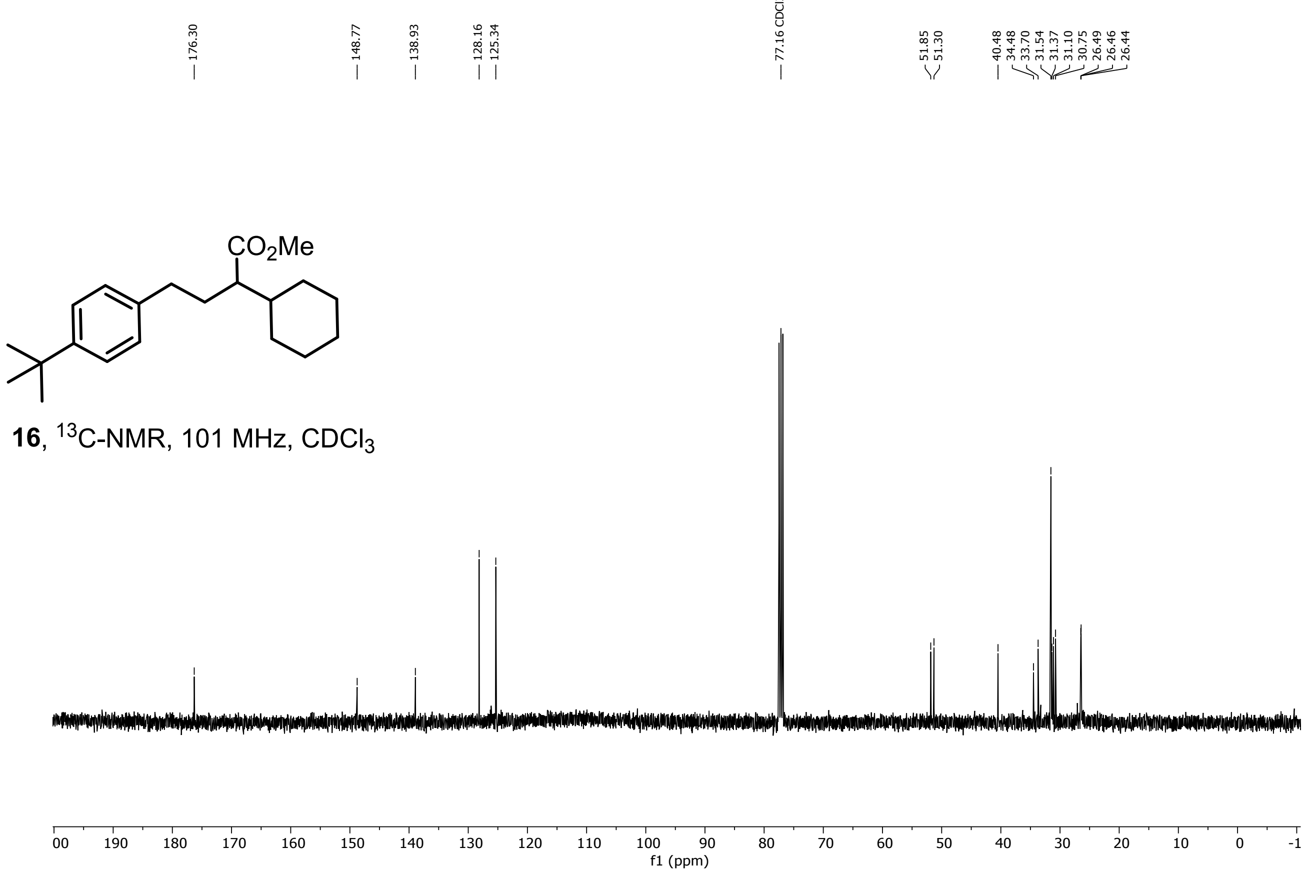

^{13}C NMR (101 MHz, $CDCl_3$) of ***methyl 4-(4-(tert-butyl)phenyl)-2-cyclohexylbutanoate (16).***

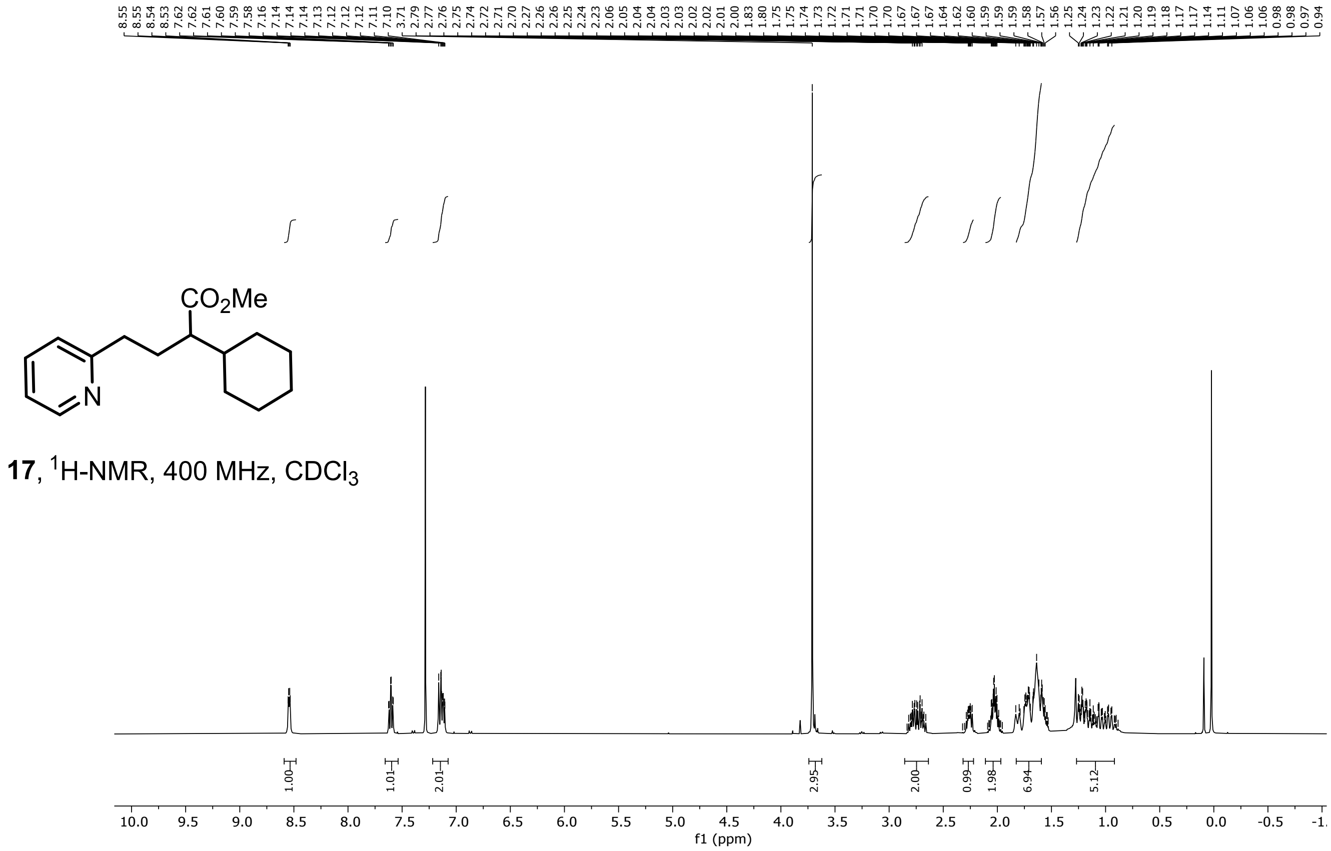

^{1}H NMR (400 MHz, CDCl$_3$) of ***methyl 2-cyclohexyl-4-(pyridin-2-yl)butanoate (17).***

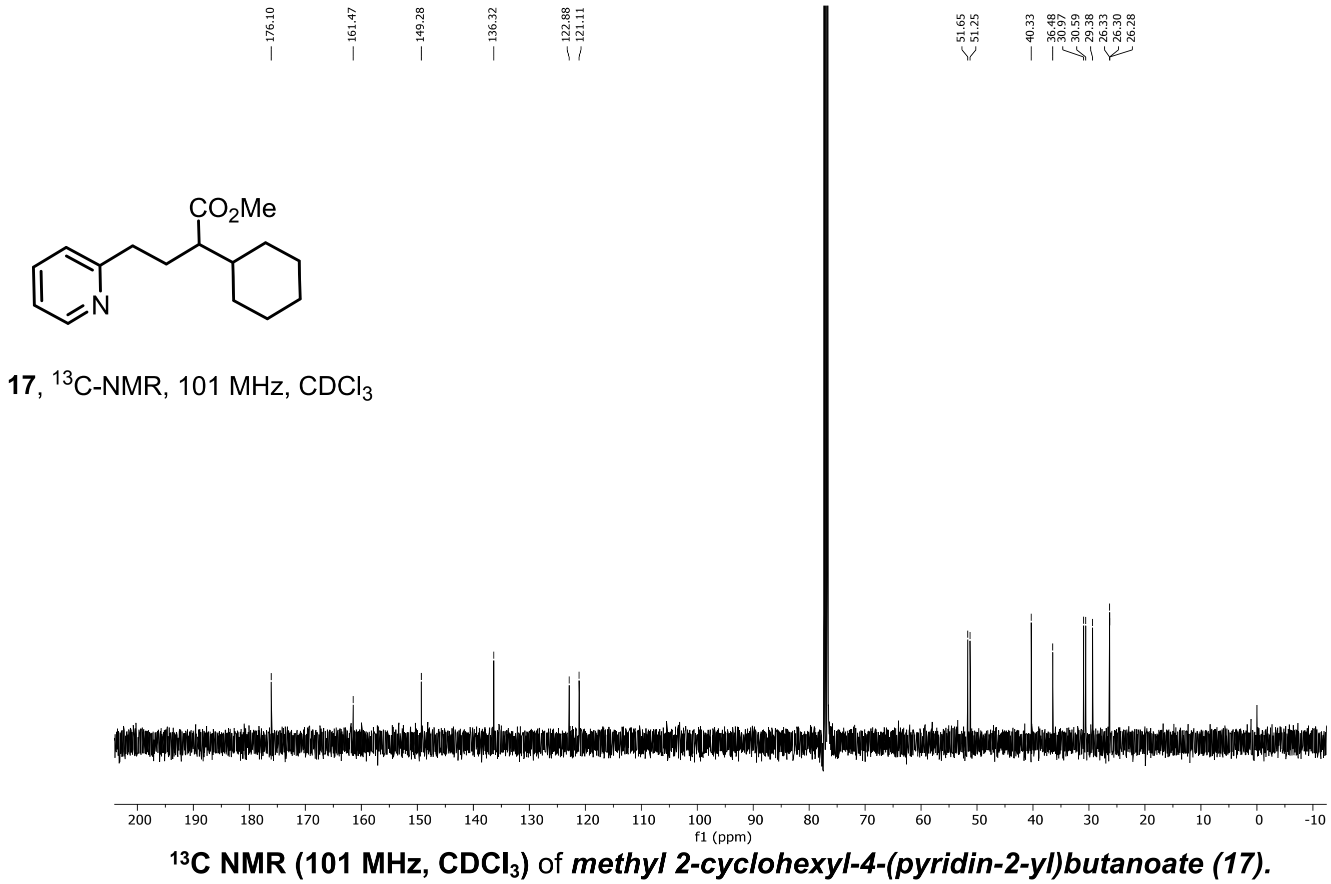

^{13}C NMR (101 MHz, CDCl$_3$) of ***methyl 2-cyclohexyl-4-(pyridin-2-yl)butanoate (17).***

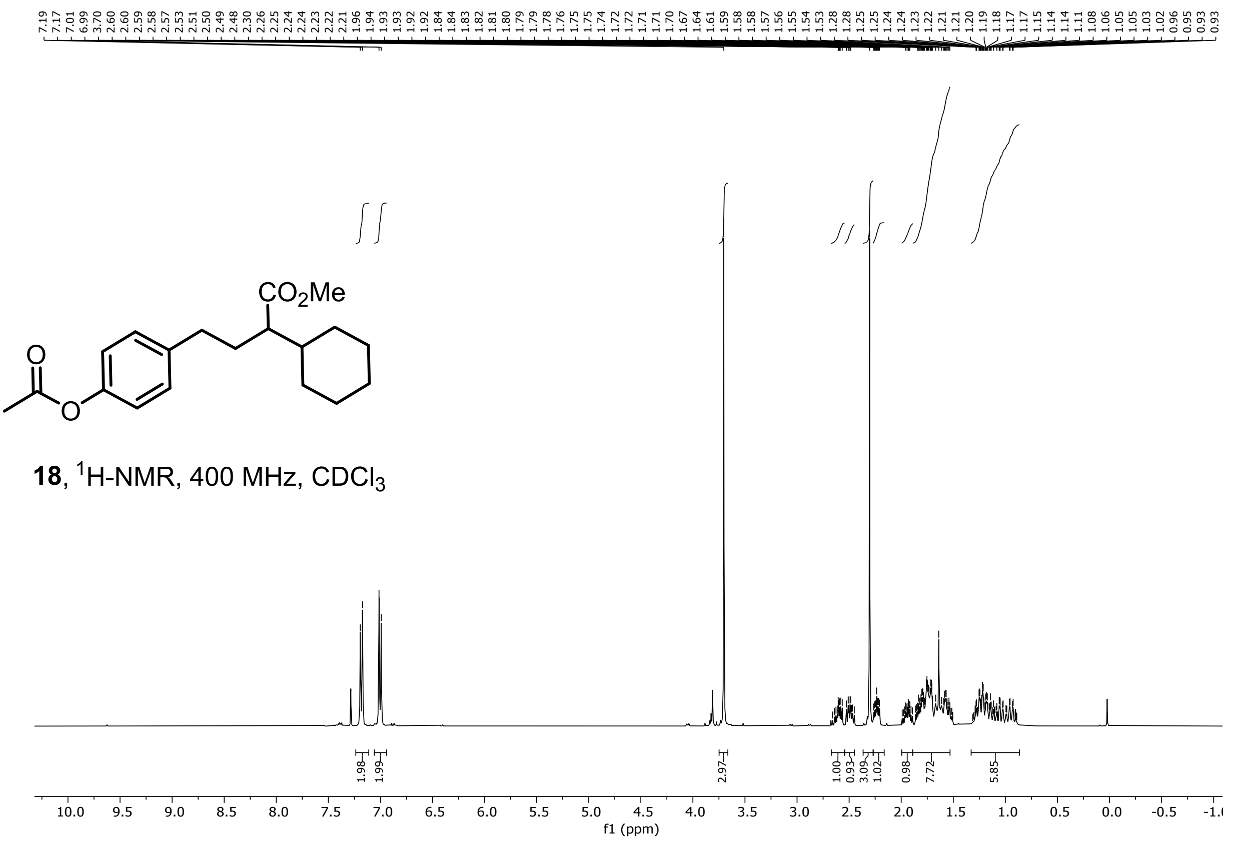

^{1}H NMR (400 MHz, $CDCl_3$) of ***methyl 4-(4-acetoxyphenyl)-2-cyclohexylbutanoate (18).***

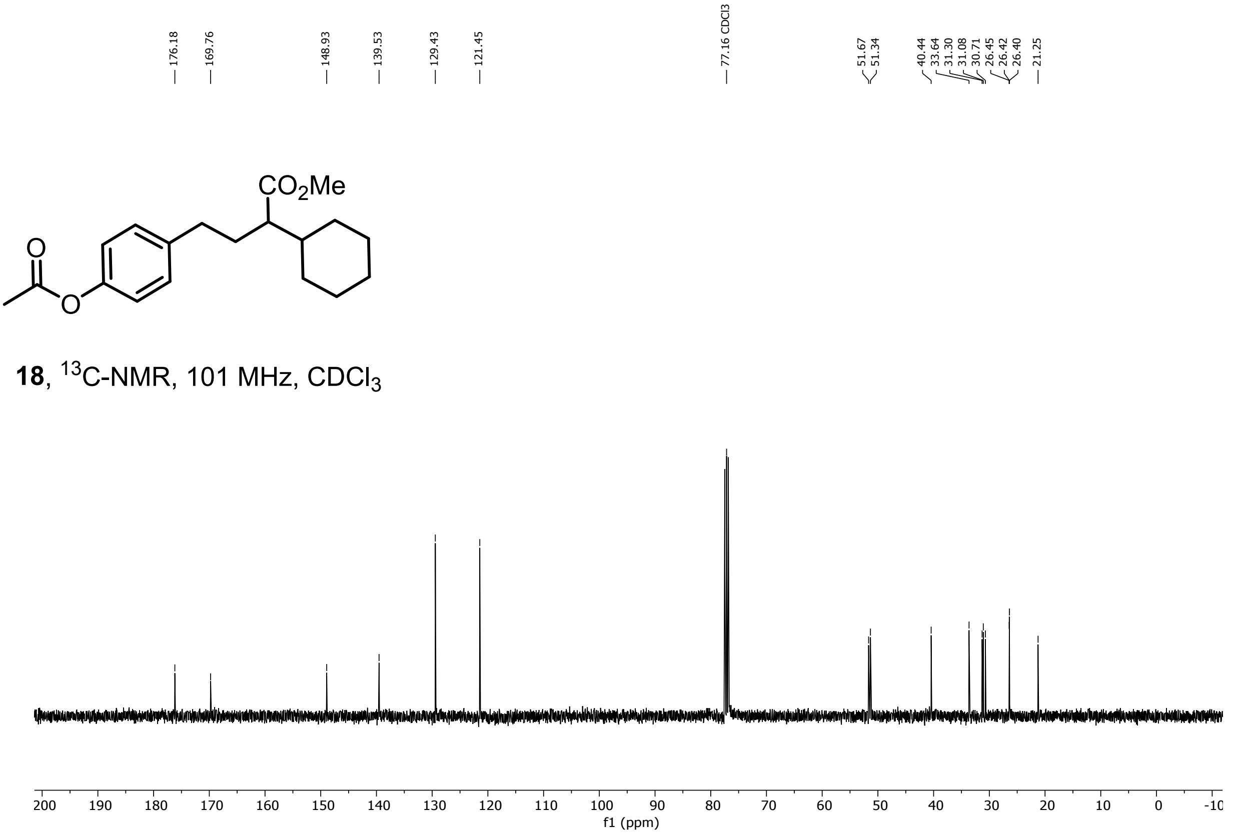

^{13}C NMR (101 MHz, $CDCl_3$) of ***methyl 4-(4-acetoxyphenyl)-2-cyclohexylbutanoate (18).***

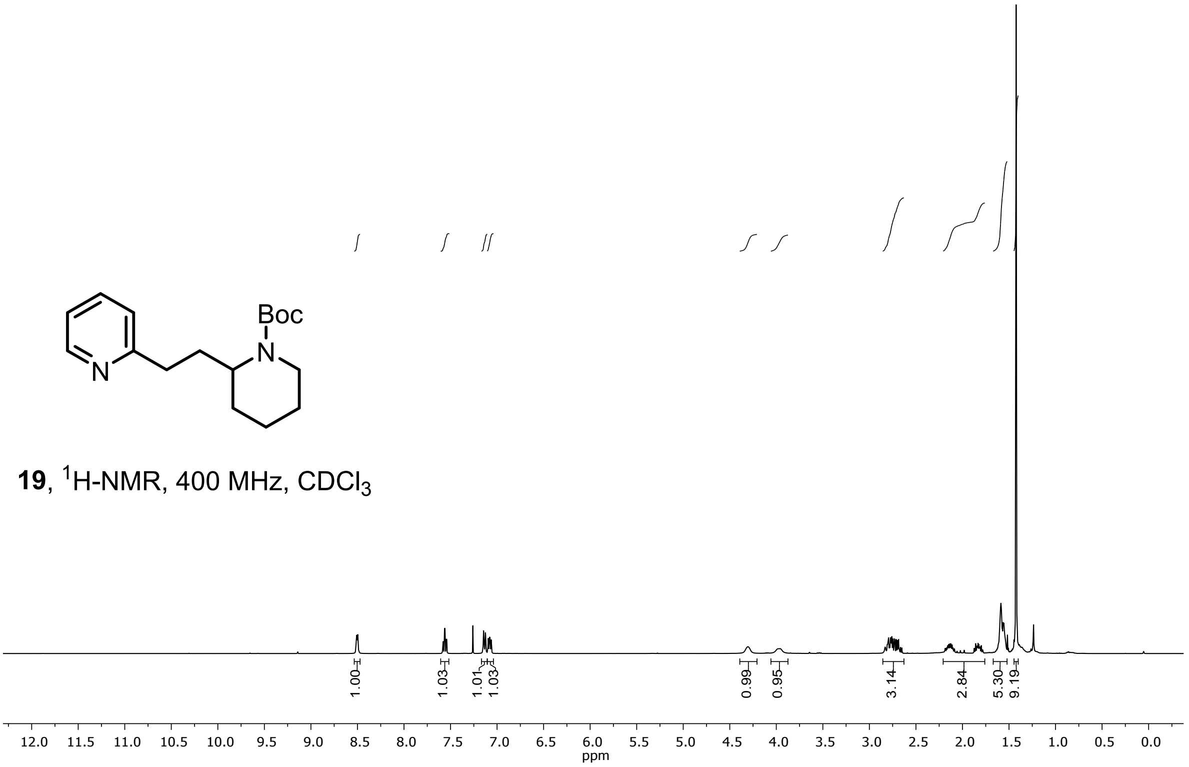

^{1}H NMR (400 MHz, $CDCl_3$) of **tert-Butyl-2-(2-(pyridin-2-yl)ethyl)piperidine-1-carboxylate (19).**

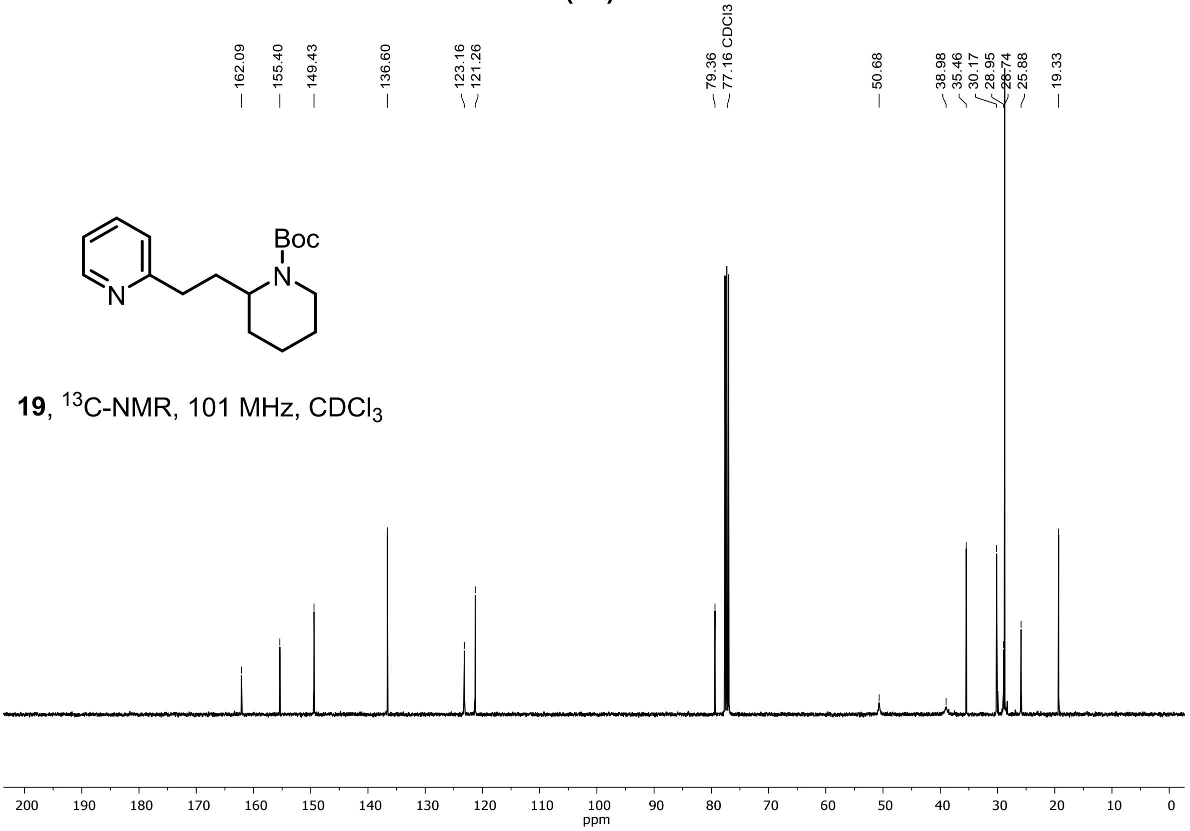

^{13}C NMR (101 MHz, $CDCl_3$) of **tert-Butyl-2-(2-(pyridin-2-yl)ethyl)piperidine-1-carboxylate (19)**

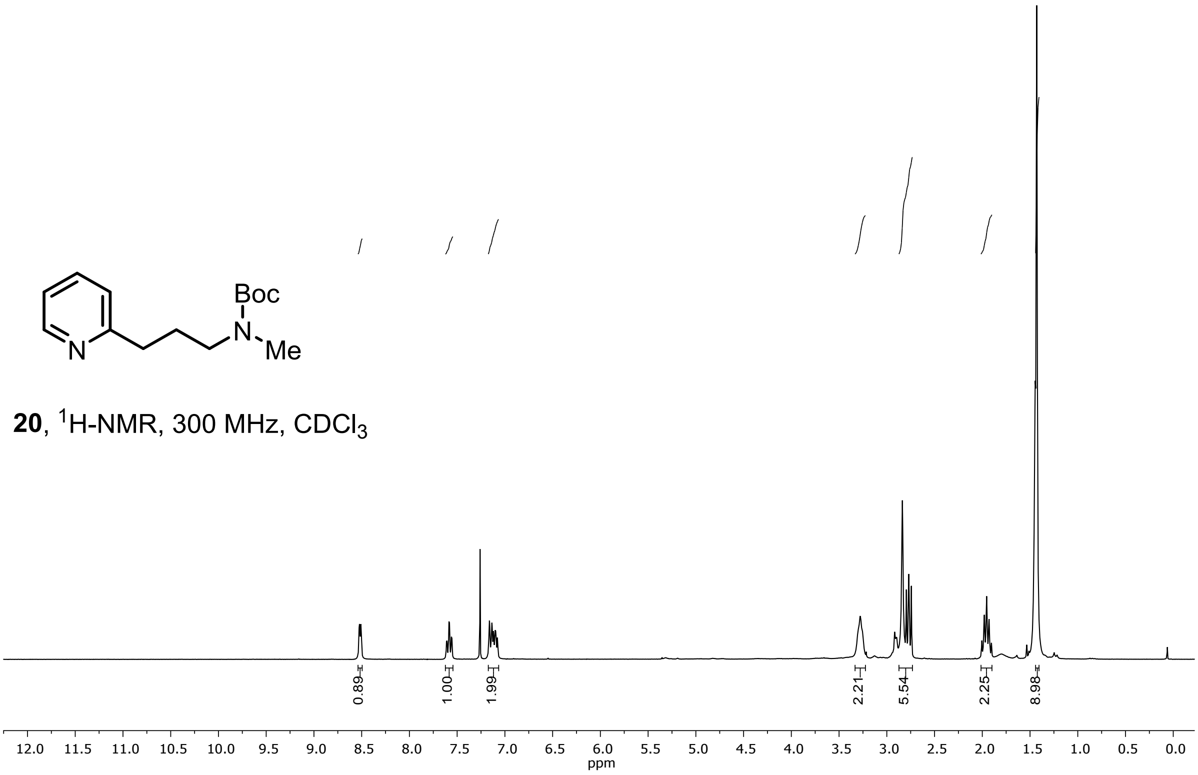

^{1}H NMR (300 MHz, $CDCl_3$) of **tert-Butyl methyl(3-(pyridin-2-yl)propyl)carbamate (20).**

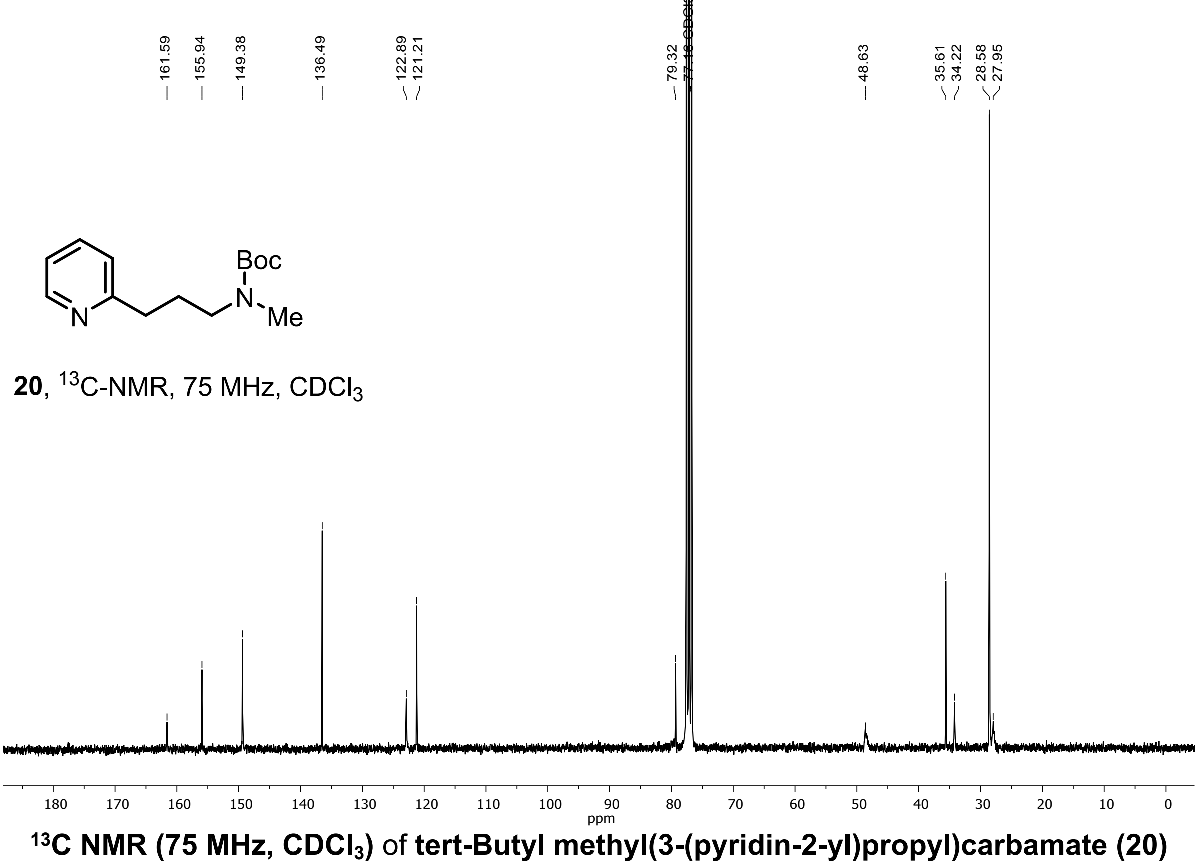

^{13}C NMR (75 MHz, $CDCl_3$) of **tert-Butyl methyl(3-(pyridin-2-yl)propyl)carbamate (20)**

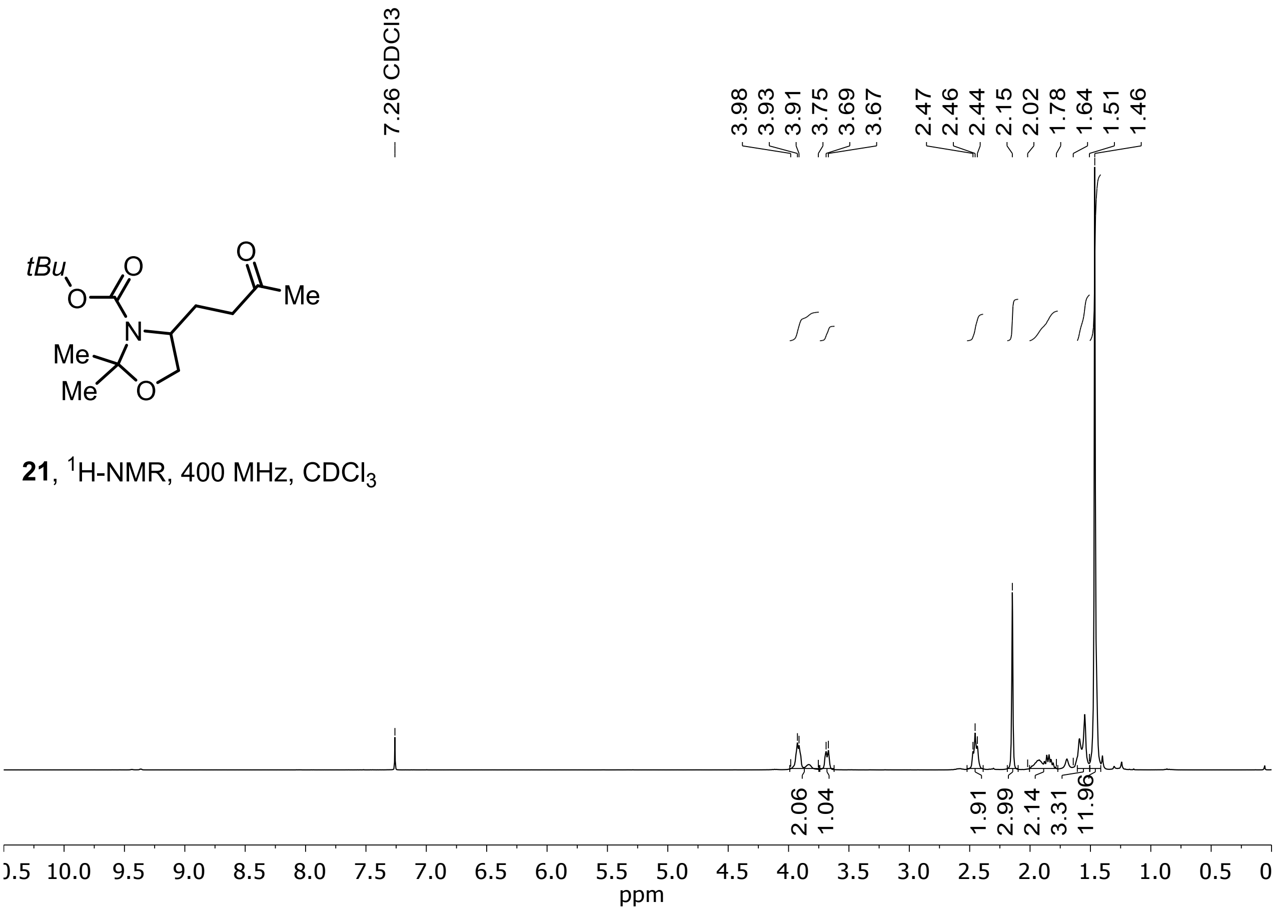

^{1}H NMR (400 MHz, $CDCl_3$) of ***tert-Butyl 2,2-dimethyl-4-(3-oxobutyl)oxazolidine-3-carboxylate (21)***

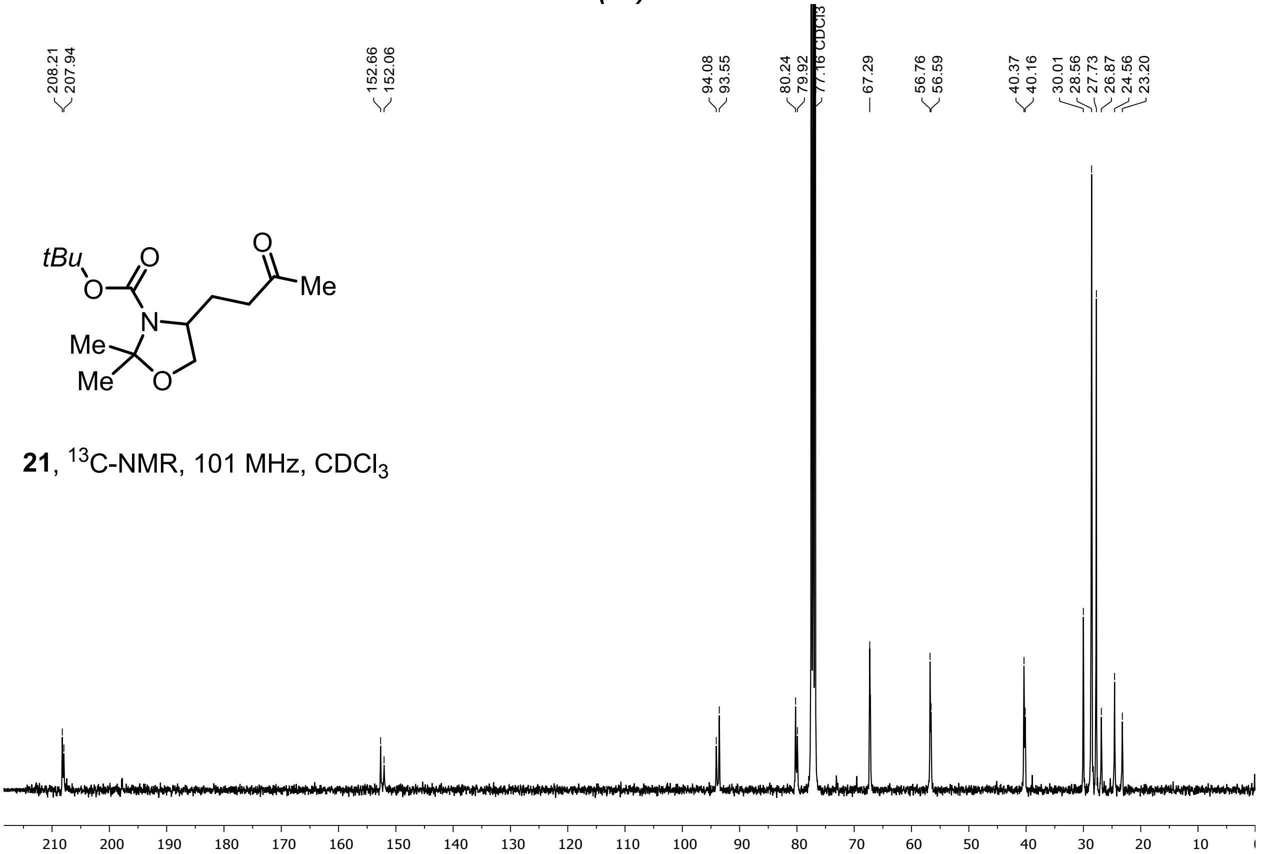

^{13}C NMR (101 MHz, $CDCl_3$) of ***tert-Butyl 2,2-dimethyl-4-(3-oxobutyl)oxazolidine-3-carboxylate (21)***

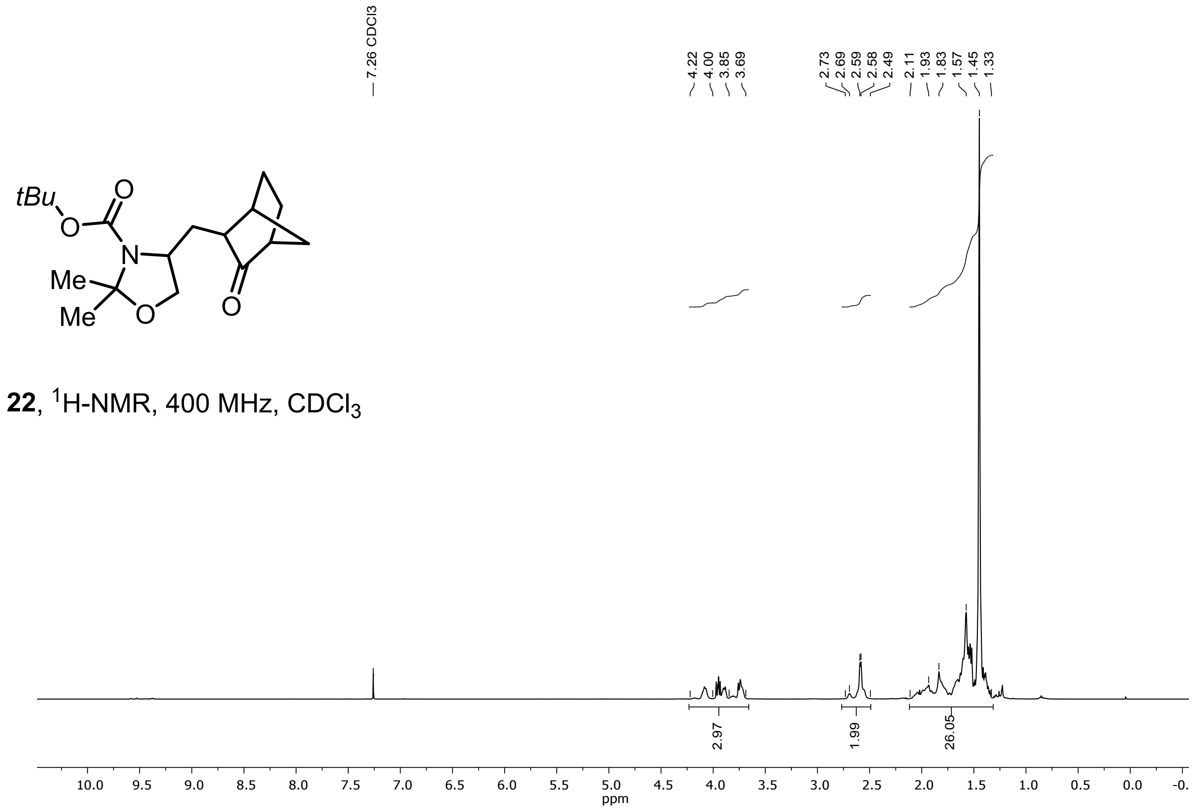

^{1}H NMR (400 MHz, CDCl$_3$) of ***tert-Butyl 2,2-dimethyl-4-(((1S,2S,4R)-3-oxobicyclo[2.2.1]heptan-2-yl)methyl)oxazolidine-3-carboxylate (22).***

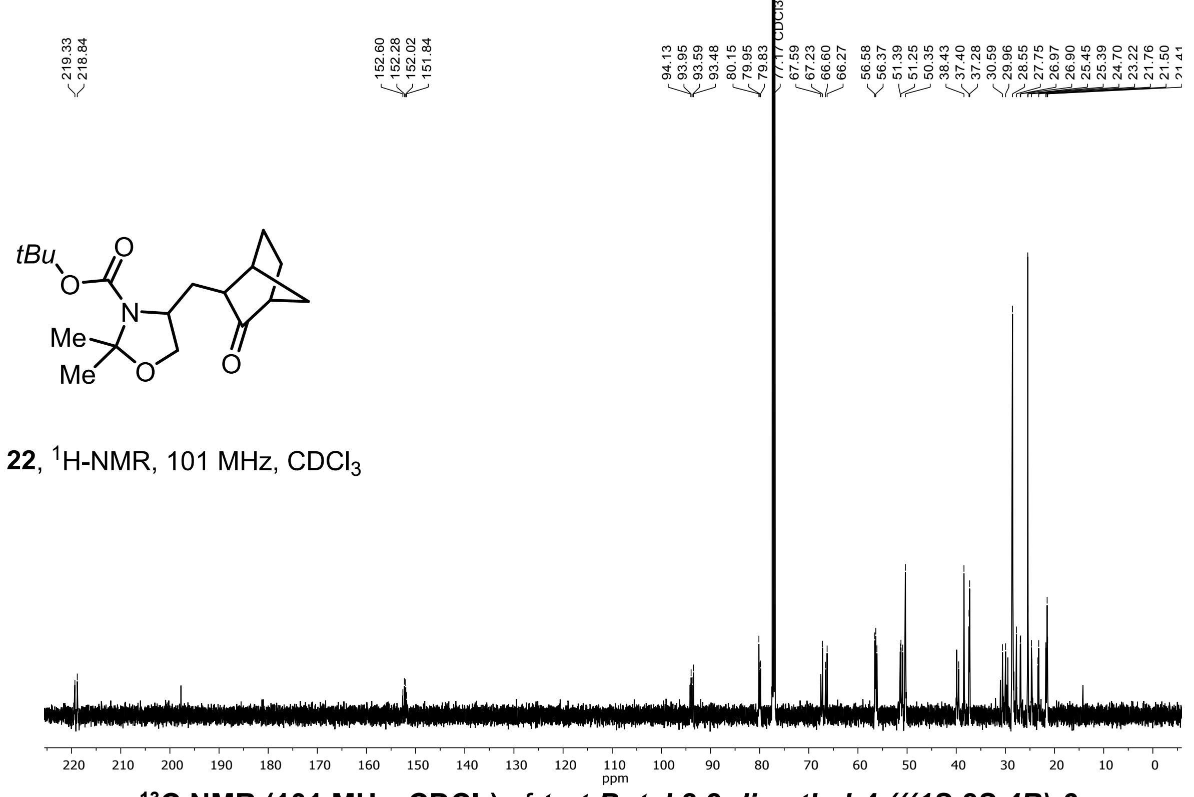

^{13}C NMR (101 MHz, CDCl$_3$) of ***tert-Butyl 2,2-dimethyl-4-(((1S,2S,4R)-3-oxobicyclo[2.2.1]heptan-2-yl)methyl)oxazolidine-3-carboxylate (22).***

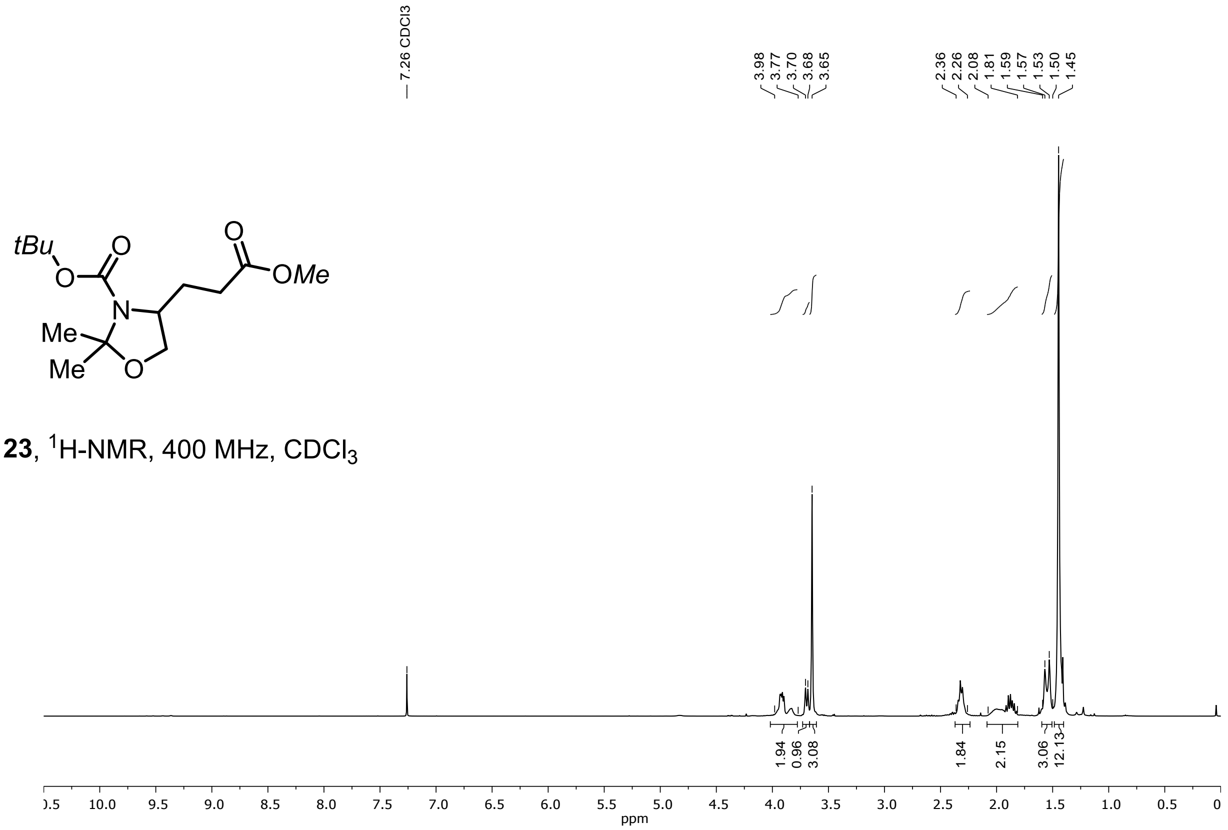

^{1}H NMR (400 MHz, $CDCl_3$) of ***tert-Butyl 4-(3-methoxy-3-oxopropyl)-2,2-dimethyloxazolidine-3-carboxylate (23).***

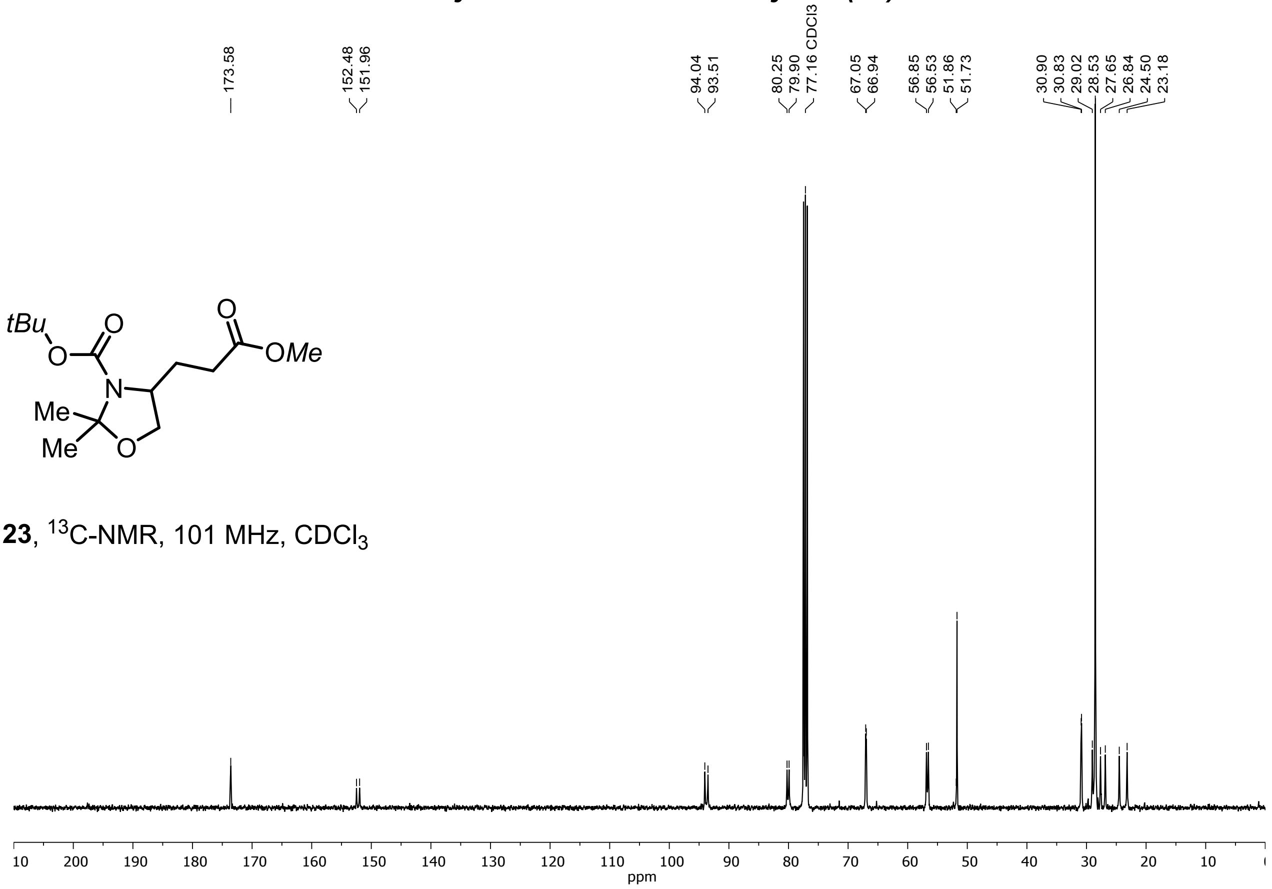

^{13}C NMR (101 MHz, $CDCl_3$) of ***tert-Butyl 4-(3-methoxy-3-oxopropyl)-2,2-dimethyloxazolidine-3-carboxylate (23).***

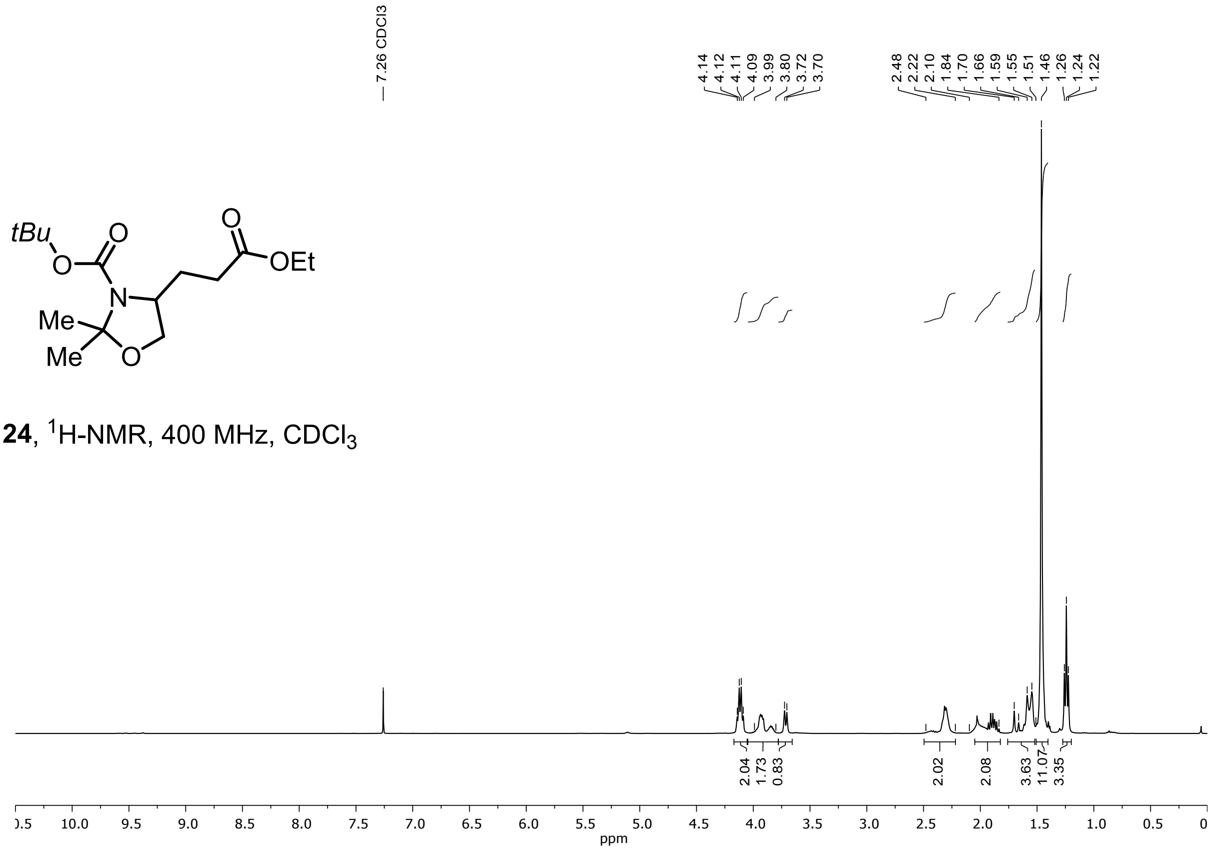

^{1}H NMR (400 MHz, $CDCl_3$) of ***tert-Butyl 4-(3-ethoxy-3-oxopropyl)-2,2-dimethyloxazolidine-3-carboxylate (24).***

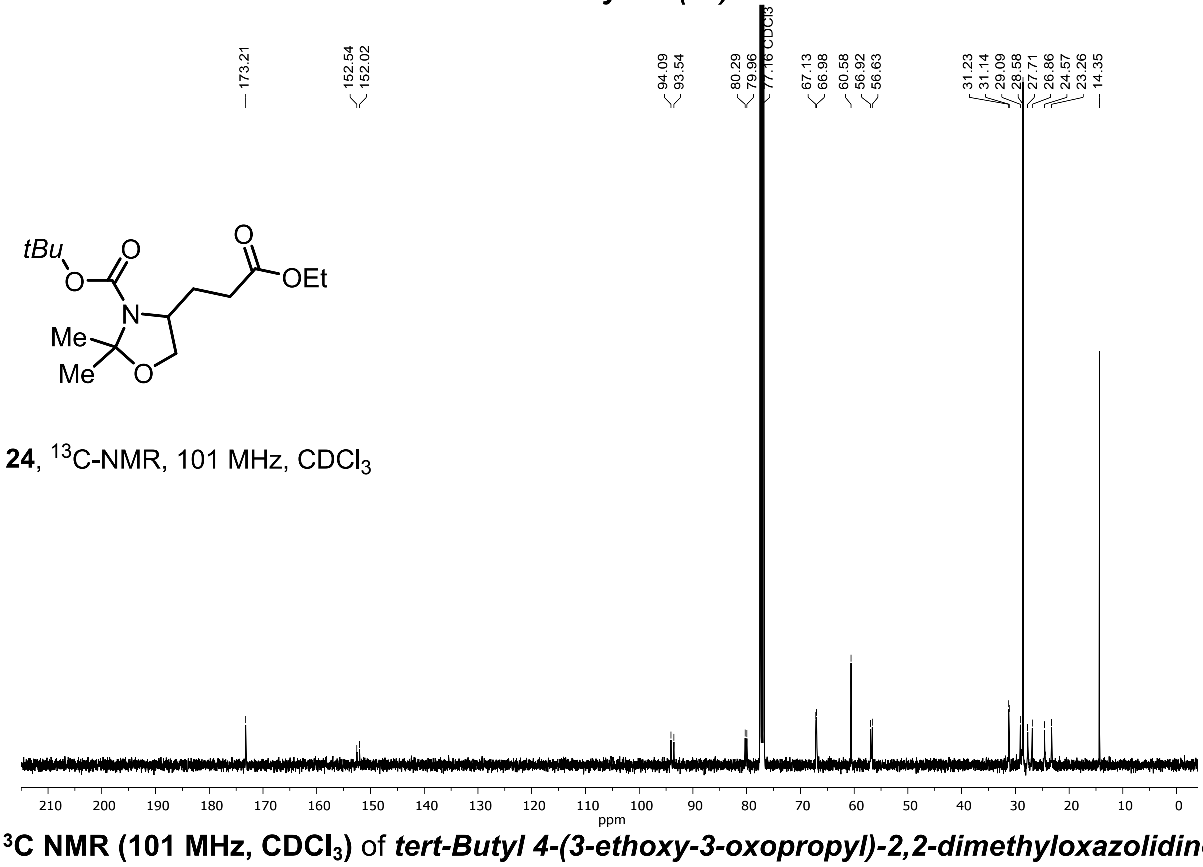

^{13}C NMR (101 MHz, $CDCl_3$) of ***tert-Butyl 4-(3-ethoxy-3-oxopropyl)-2,2-dimethyloxazolidine-3-carboxylate (24).***

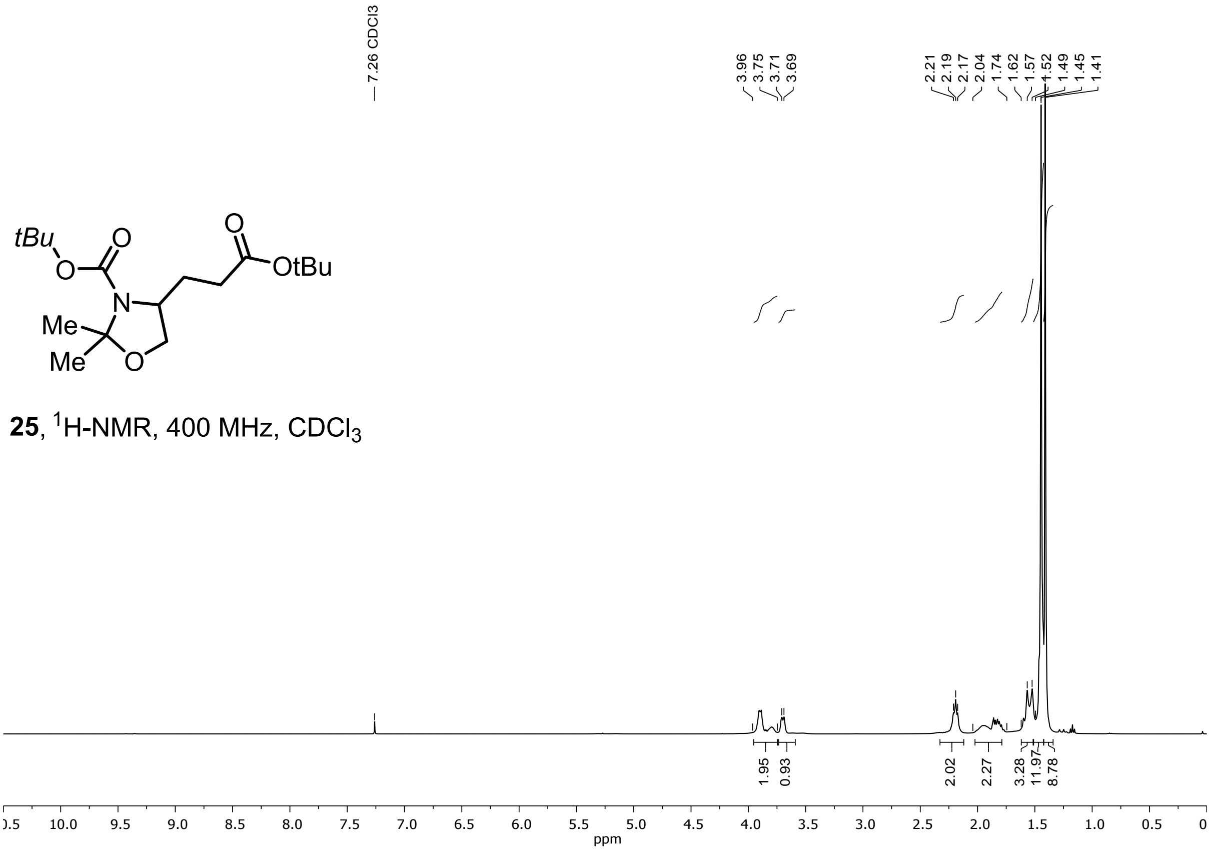

^{1}H NMR (400 MHz, $CDCl_3$) of ***tert-Butyl 4-(3-(tert-butoxy)-3-oxopropyl)-2,2-dimethyloxazolidine-3-carboxylate (25).***

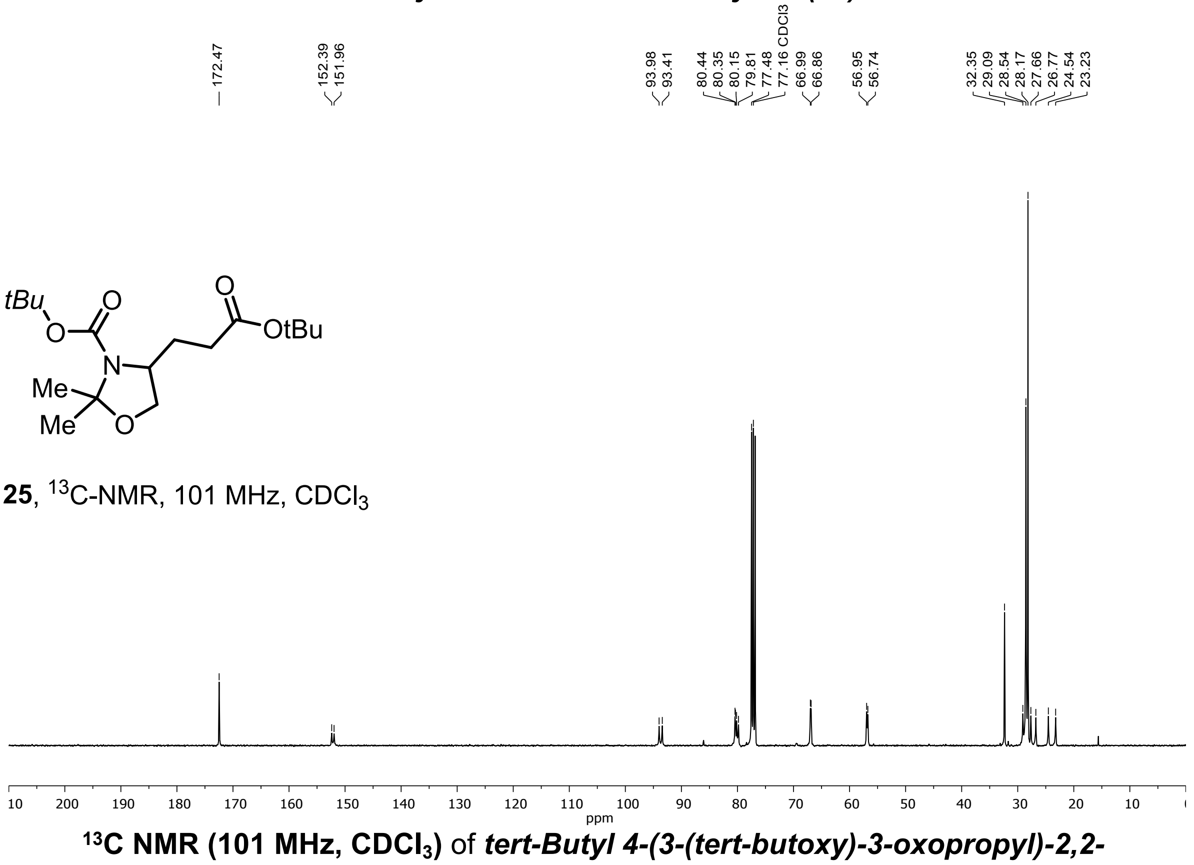

^{13}C NMR (101 MHz, $CDCl_3$) of ***tert-Butyl 4-(3-(tert-butoxy)-3-oxopropyl)-2,2-dimethyloxazolidine-3-carboxylate (25).***

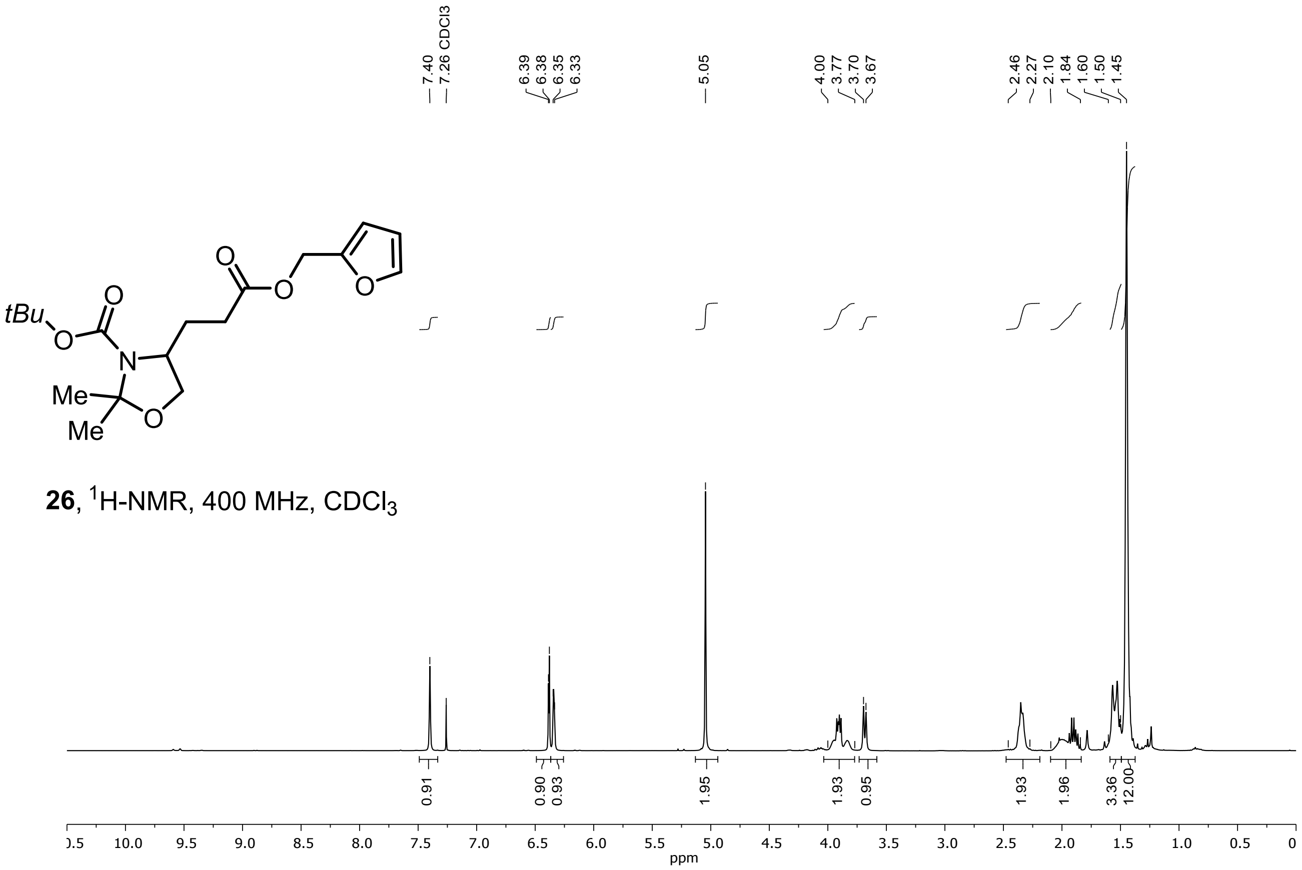

^{1}H NMR (400 MHz, CDCl$_3$) of ***tert-Butyl 4-(3-(furan-2-ylmethoxy)-3-oxopropyl)-2,2-dimethyloxazolidine-3-carboxylate (26).***

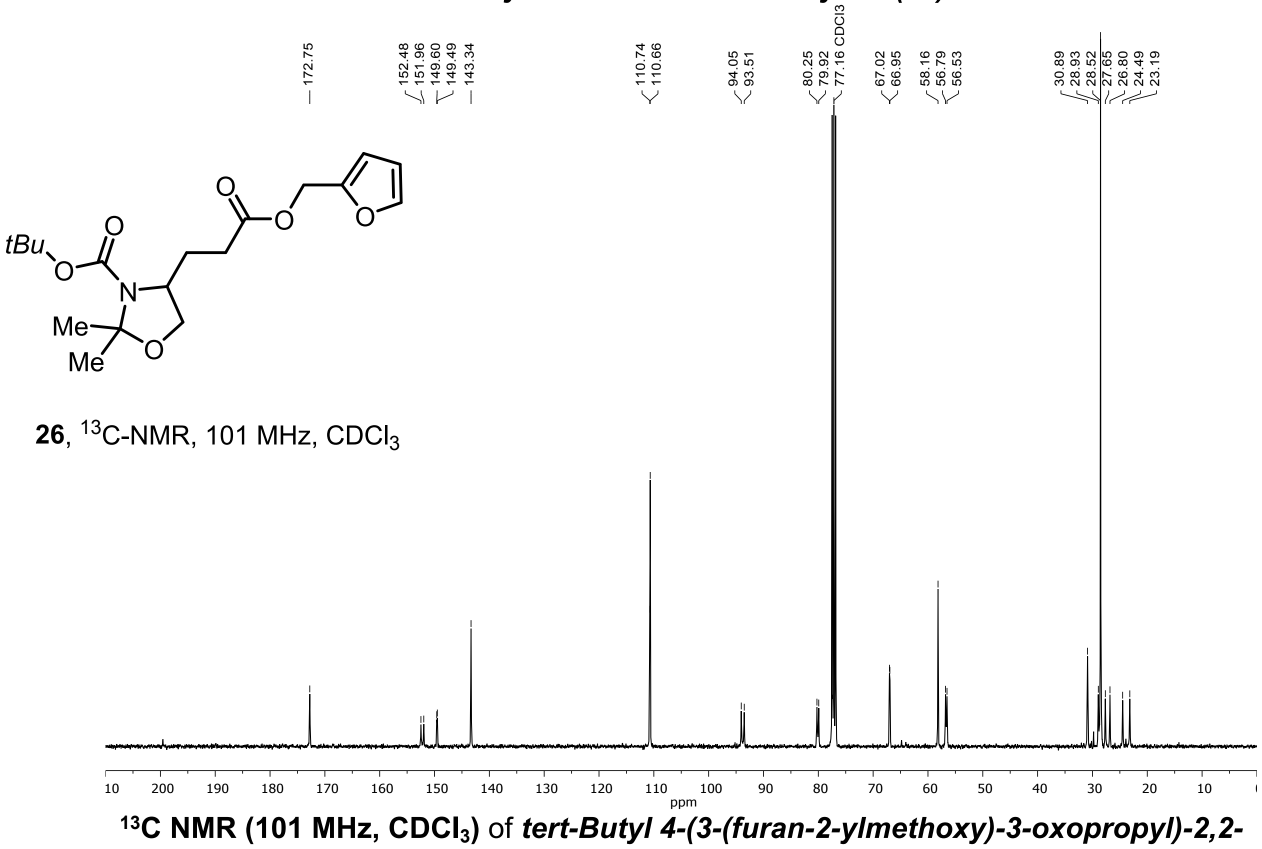

^{13}C NMR (101 MHz, CDCl$_3$) of ***tert-Butyl 4-(3-(furan-2-ylmethoxy)-3-oxopropyl)-2,2-dimethyloxazolidine-3-carboxylate (26).***

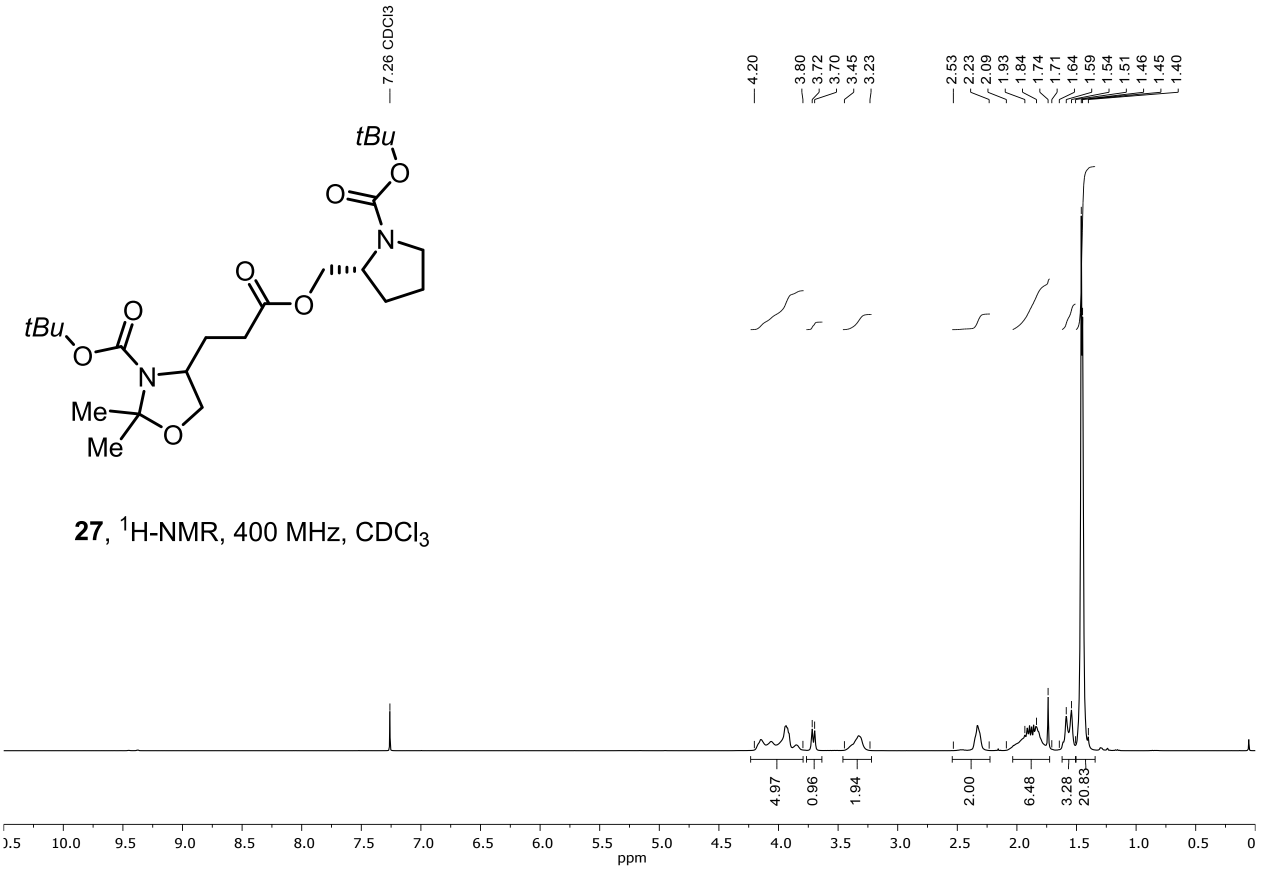

^{1}H NMR (400 MHz, $CDCl_3$) of ***tert-Butyl 4-(3-(((R)-1-(tert-butoxycarbonyl)pyrrolidin-2-yl)methoxy)-3-oxopropyl)-2,2-dimethyloxazolidine-3-carboxylate (27).***

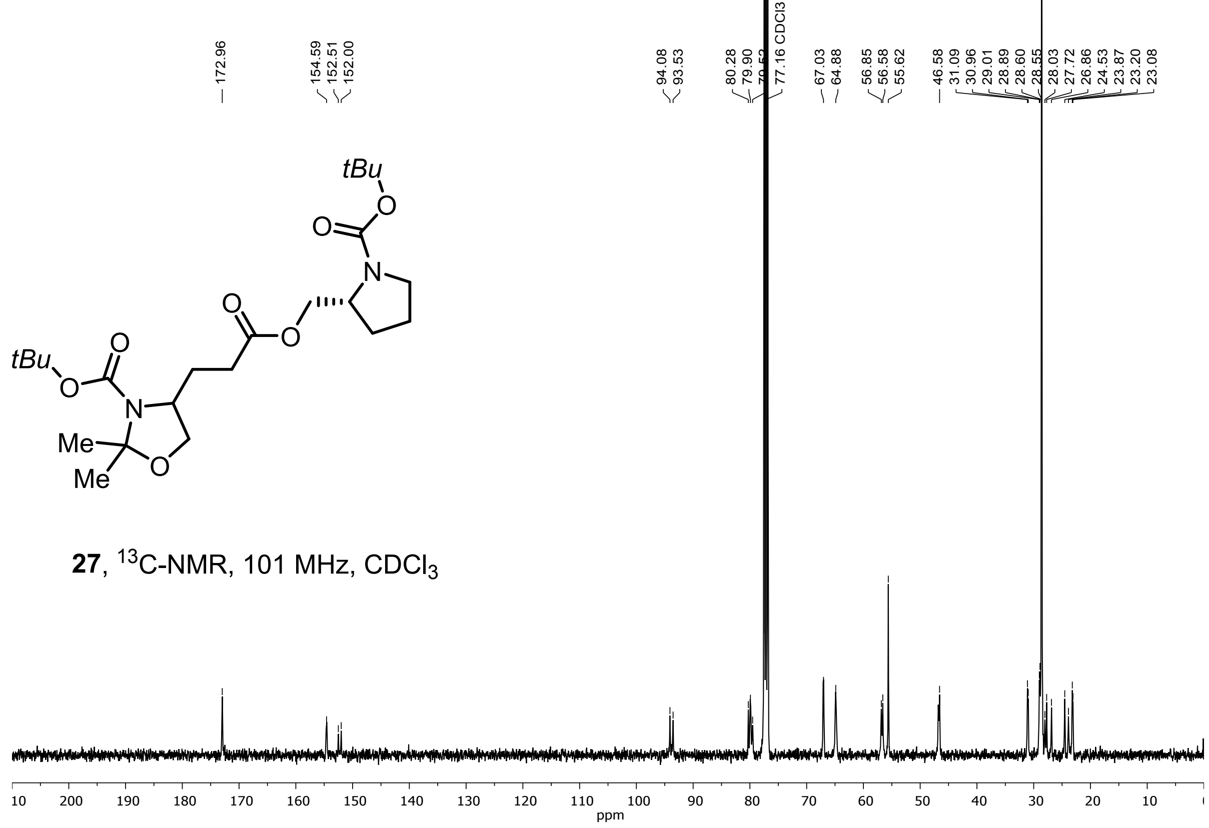

^{13}C NMR (101 MHz, $CDCl_3$) of ***tert-Butyl 4-(3-(((R)-1-(tert-butoxycarbonyl)pyrrolidin-2-yl)methoxy)-3-oxopropyl)-2,2-dimethyloxazolidine-3-carboxylate (27).***

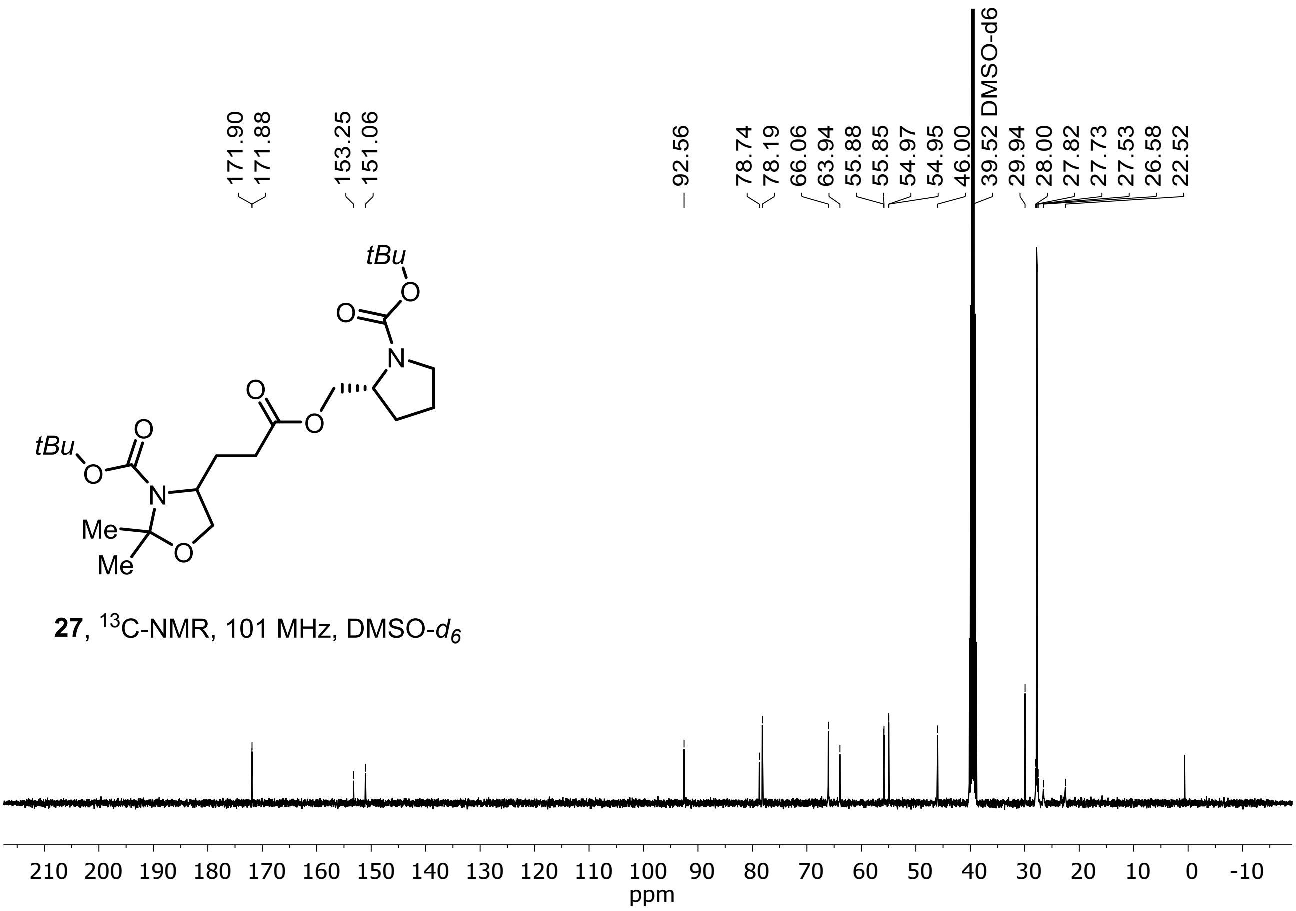

^{13}C NMR (101 MHz, DMSO, 70 °C) of ***tert-Butyl 4-(3-(((R)-1-(tert-butoxycarbonyl)pyrrolidin-2-yl)methoxy)-3-oxopropyl)-2,2-dimethyloxazolidine-3-carboxylate (27).***

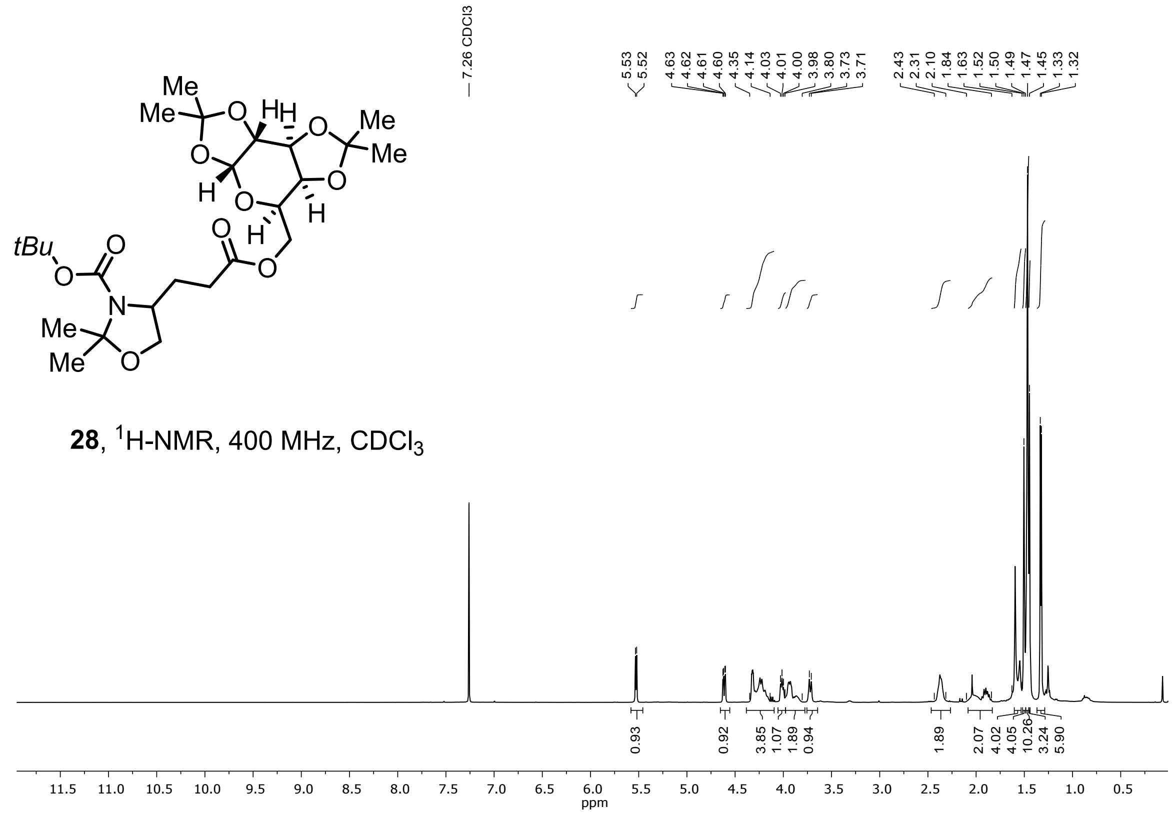

^{1}H NMR (400 MHz, CDCl$_3$) of ***tert-Butyl 2,2-dimethyl-4-(3-oxo-3-(((3aR,5R,5aS,8aS)-2,2,7,7-tetramethyltetrahydro-5H-bis([1,3]dioxolo)[4,5-b:4',5'-d]pyran-5-yl)methoxy)propyl)oxazolidine-3-carboxylate (28).***

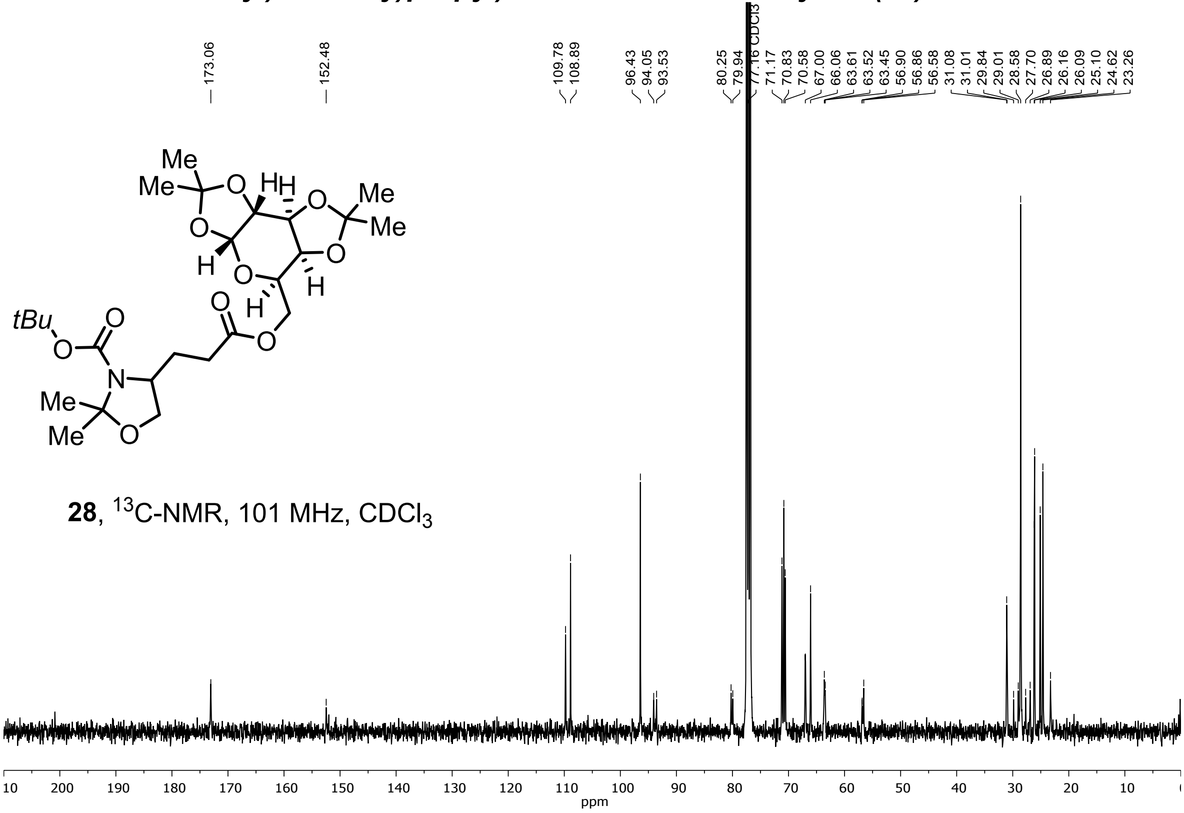

^{13}C NMR (101 MHz, CDCl$_3$) of ***tert-Butyl 2,2-dimethyl-4-(3-oxo-3-(((3aR,5R,5aS,8aS)-2,2,7,7-tetramethyltetrahydro-5H-bis([1,3]dioxolo)[4,5-b:4',5'-d]pyran-5-yl)methoxy)propyl)oxazolidine-3-carboxylate (28).***

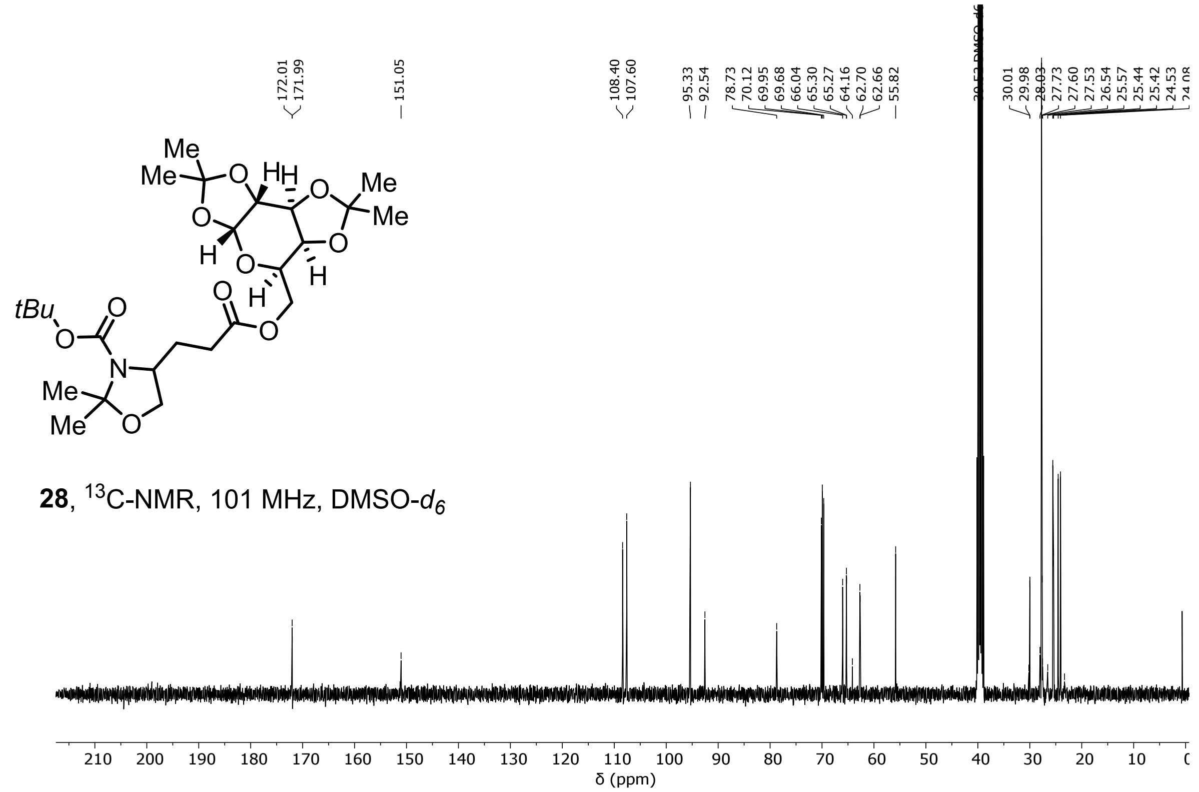

^{13}C NMR (101 MHz, DMSO, 70 °C) of ***tert-Butyl 2,2-dimethyl-4-(3-oxo-3-(((3aR,5R,5aS,8aS)-2,2,7,7-tetramethyltetrahydro-5H-bis([1,3]dioxolo)[4,5-b:4',5'-d]pyran-5-yl)methoxy)propyl)oxazolidine-3-carboxylate (28).***

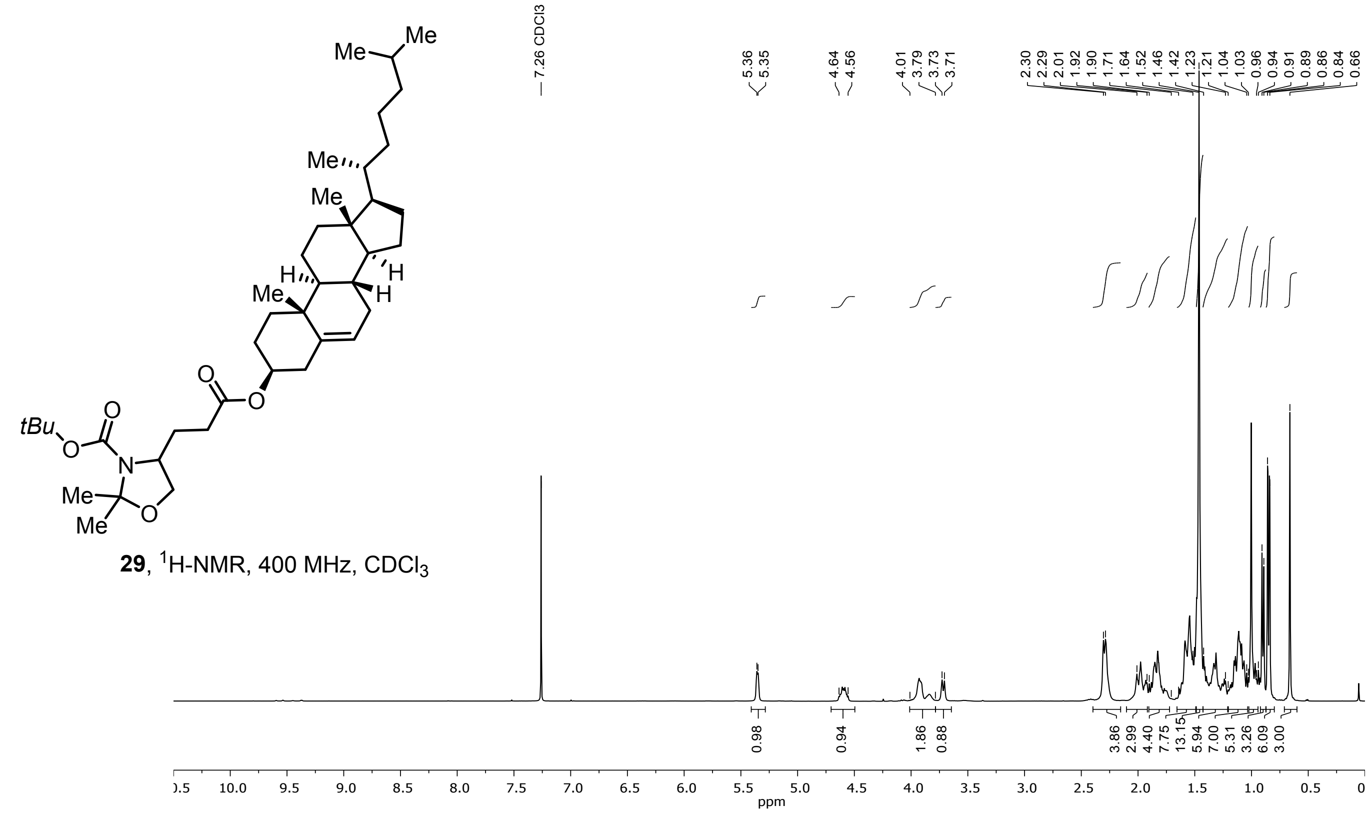

^{1}H NMR (400 MHz, CDCl$_3$) of ***Tert-butyl 4-(3-(((3S,8S,9S,10R,13R,14S,17R)-10,13-dimethyl-17-((R)-6-methylheptan-2-yl)-2,3,4,7,8,9,10,11,12,13,14,15,16,17-tetradecahydro-1H-cyclopenta[a]phenanthren-3-yl)oxy)-3-oxopropyl)-2,2-dimethyloxazolidine-3-carboxylate (29)***

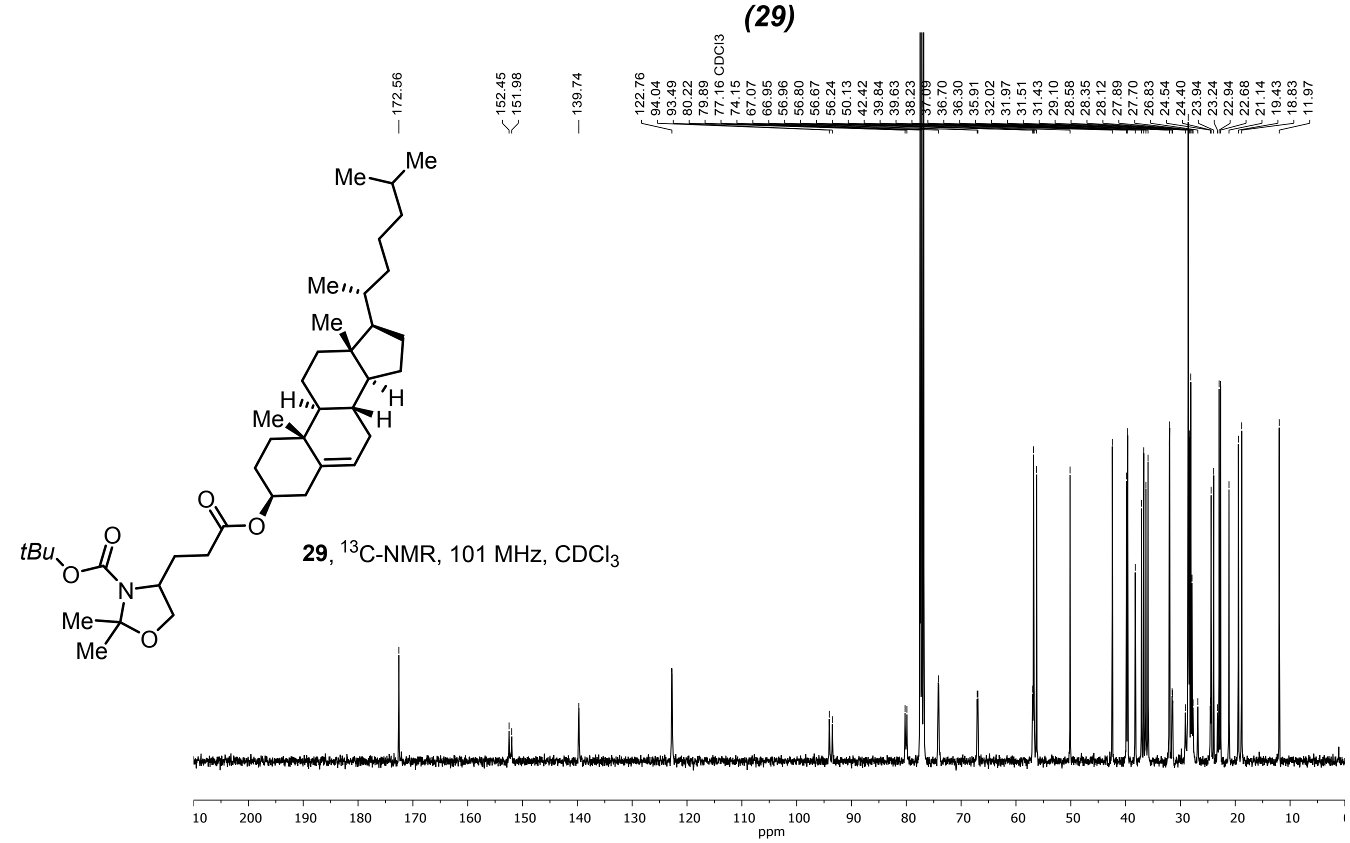

^{13}C NMR (101 MHz, CDCl$_3$) of ***Tert-butyl 4-(3-(((3S,8S,9S,10R,13R,14S,17R)-10,13-dimethyl-17-((R)-6-methylheptan-2-yl)-2,3,4,7,8,9,10,11,12,13,14,15,16,17-tetradecahydro-1H-cyclopenta[a]phenanthren-3-yl)oxy)-3-oxopropyl)-2,2-dimethyloxazolidine-3-carboxylate (29)***

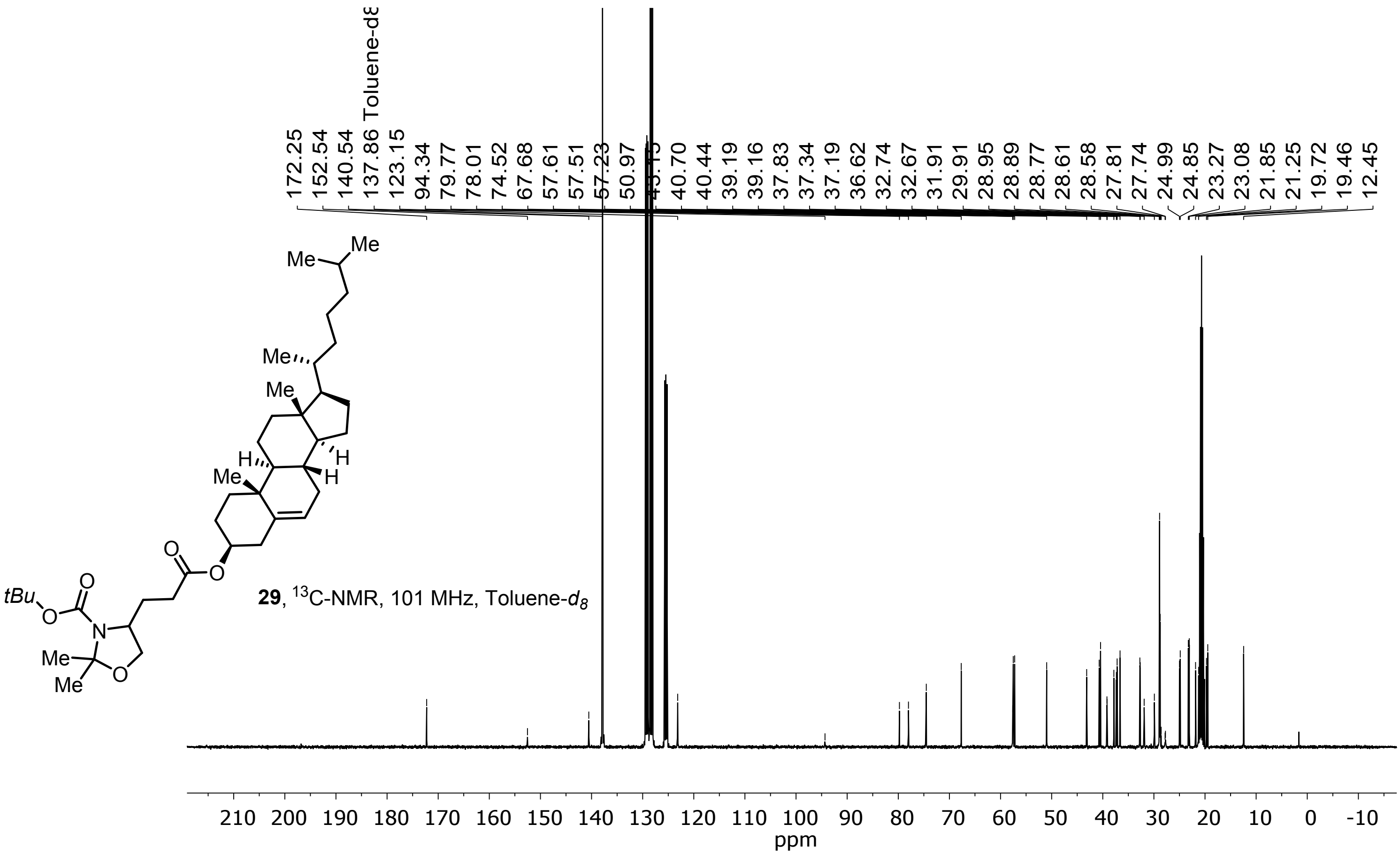

^{13}C NMR (101 MHz, Toluene-d$_8$,70 °C) of ***tert-Butyl 4-(3-(((3S,8S,9S,10R,13R,14S,17R)-10,13 dimethyl-17-((R)-6-methylheptan-2-yl)-2,3,4,7,8,9,10,11,12,13,14,15,16,17-tetradecahydro-1H-cyclopenta[a]phenanthren-3-yl)oxy)-3-oxopropyl)-2,2-dimethyloxazolidine-3-carboxylate (29)***

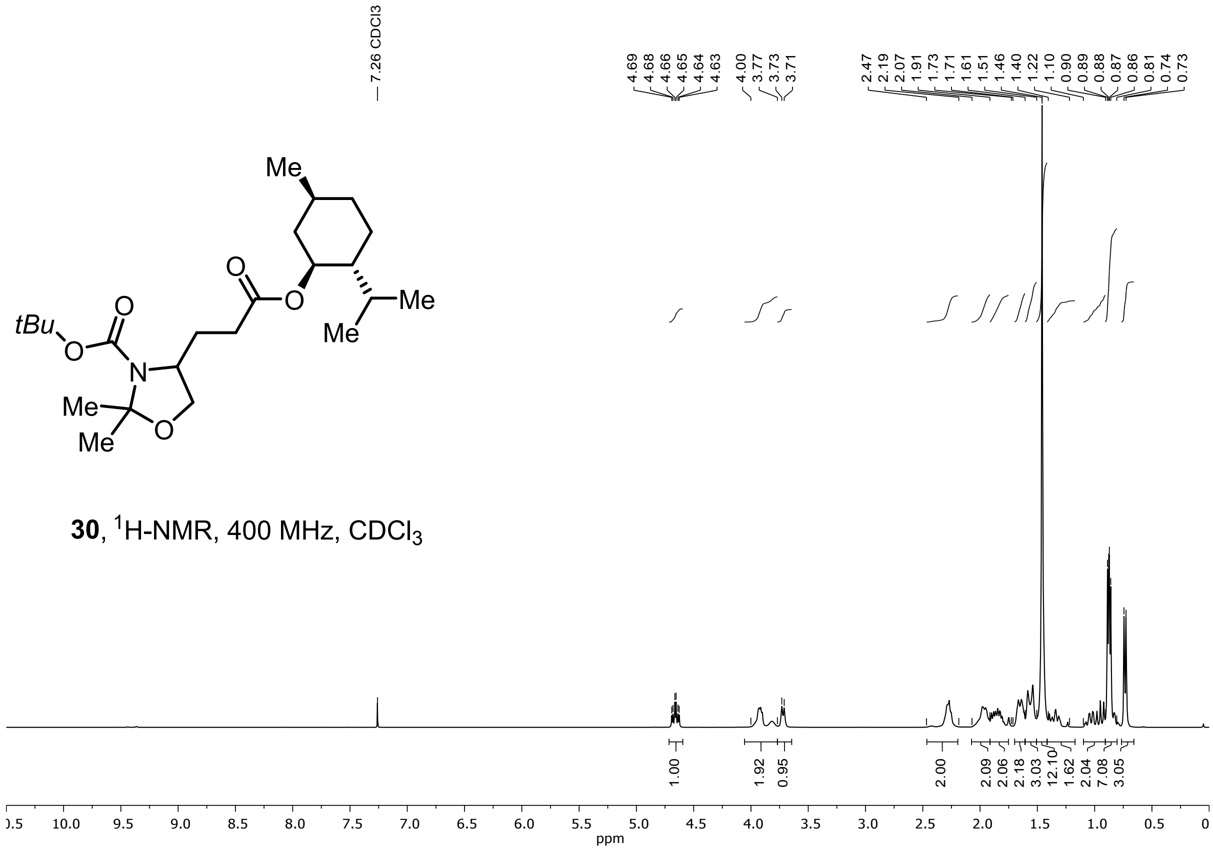

^{1}H NMR (400 MHz, $CDCl_3$) of ***tert-Butyl 4-(3-(((1S,2R,5S)-2-isopropyl-5-methylcyclohexyl)oxy)-3-oxopropyl)-2,2-dimethyloxazolidine-3-carboxylate (30).***

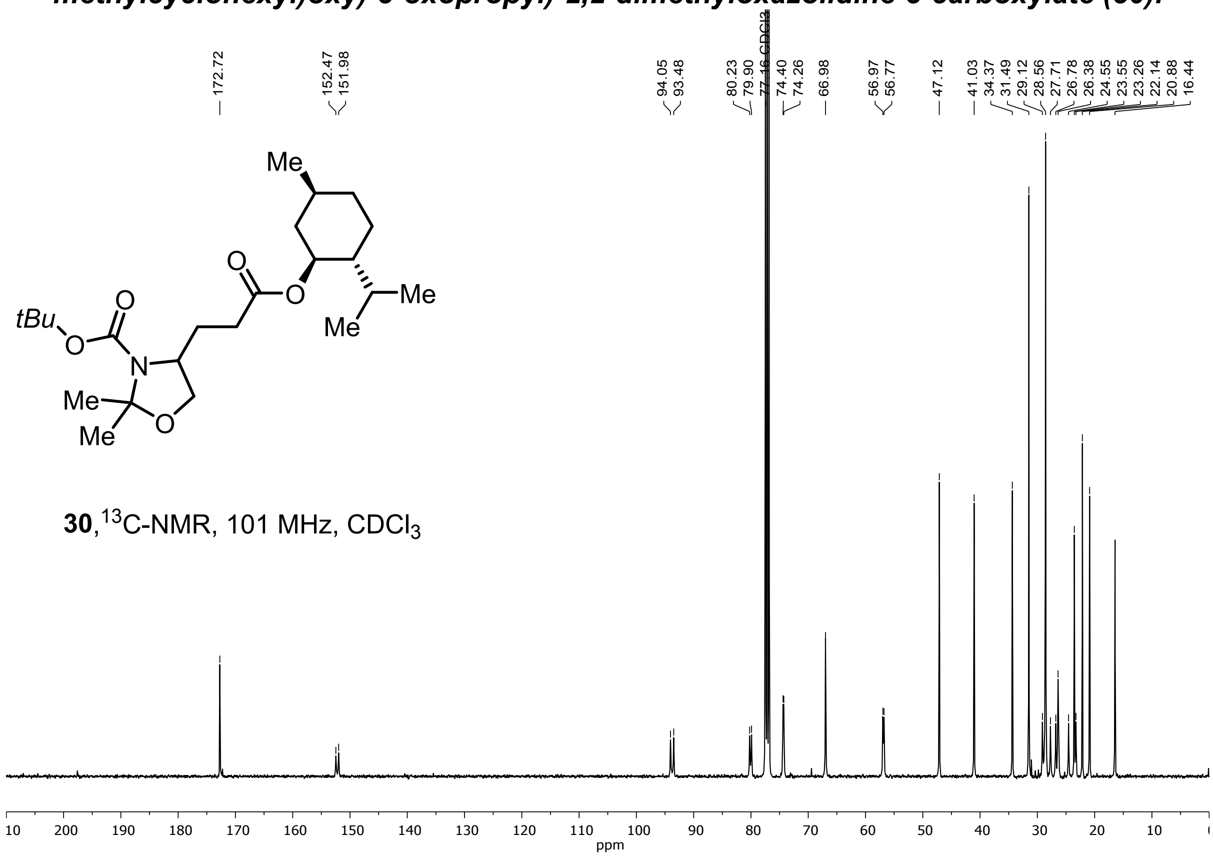

^{13}C NMR (101 MHz, $CDCl_3$) of ***tert-Butyl 4-(3-(((1S,2R,5S)-2-isopropyl-5-methylcyclohexyl)oxy)-3-oxopropyl)-2,2-dimethyloxazolidine-3-carboxylate (30).***

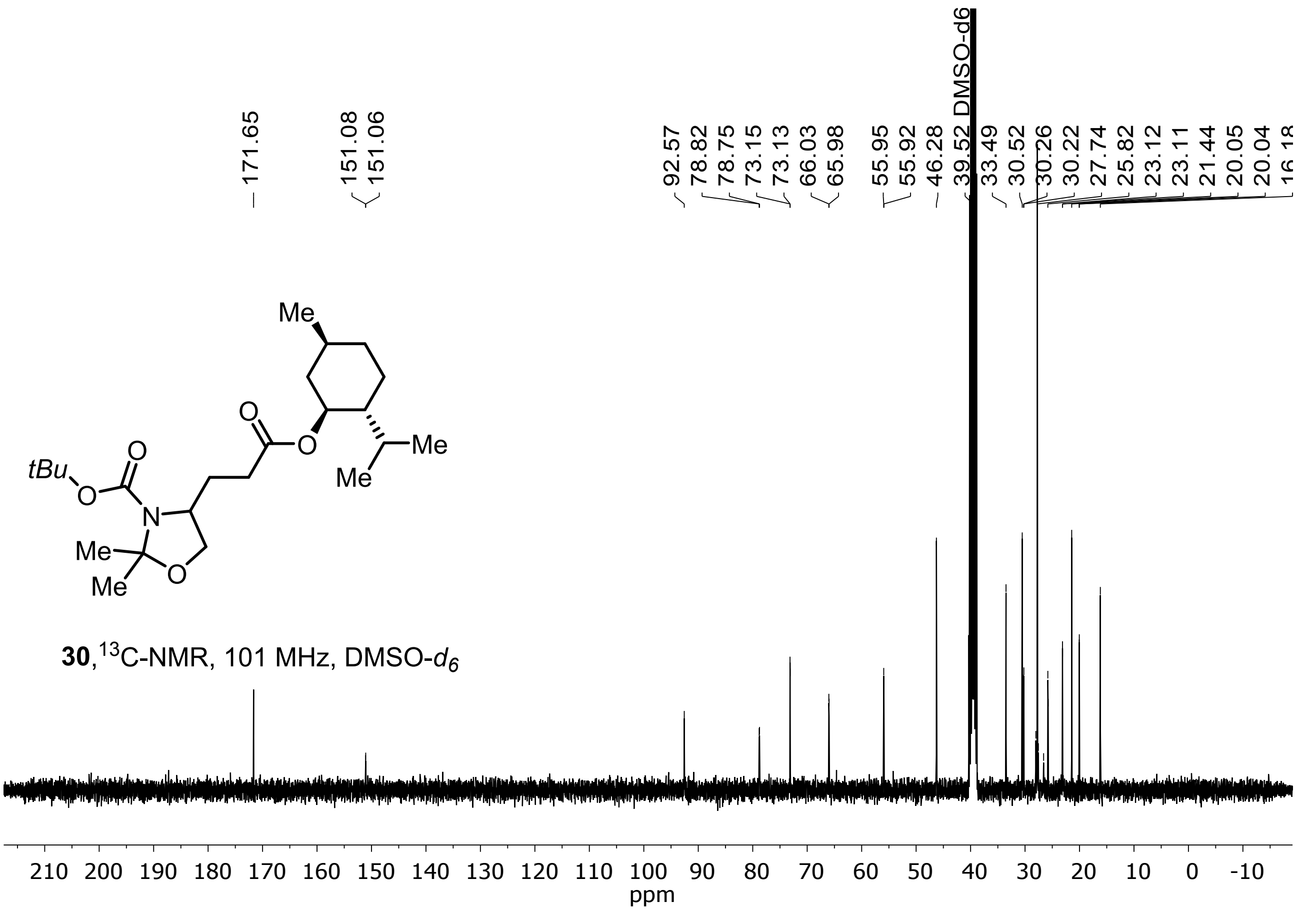

^{13}C NMR (101 MHz, DMSO, 70 °C) of ***tert-Butyl 4-(3-(((1S,2R,5S)-2-isopropyl-5-methylcyclohexyl)oxy)-3-oxopropyl)-2,2-dimethyloxazolidine-3-carboxylate (30).***

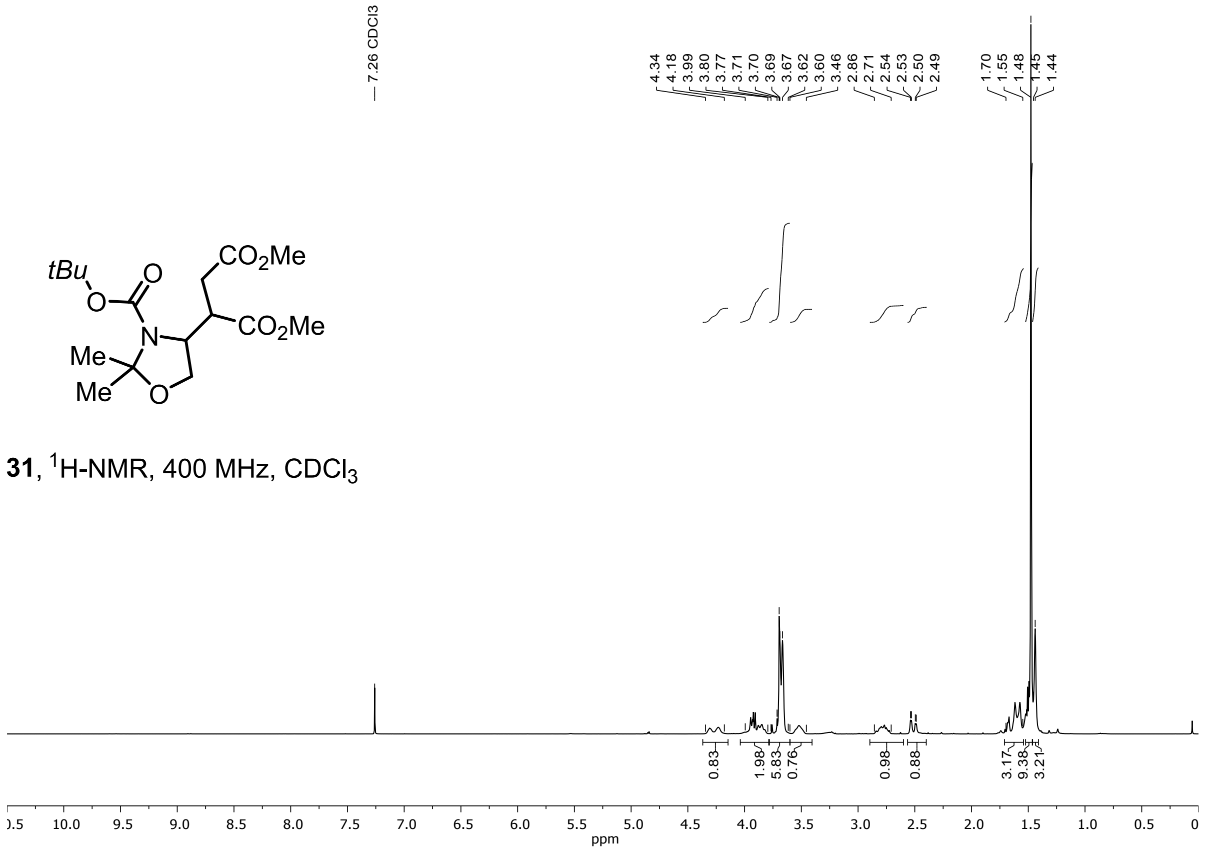

^{1}H NMR (400 MHz, CDCl$_3$) of ***Dimethyl 2-(3-(tert-butoxycarbonyl)-2,2-dimethyloxazolidin-4-yl)succinate (31).***

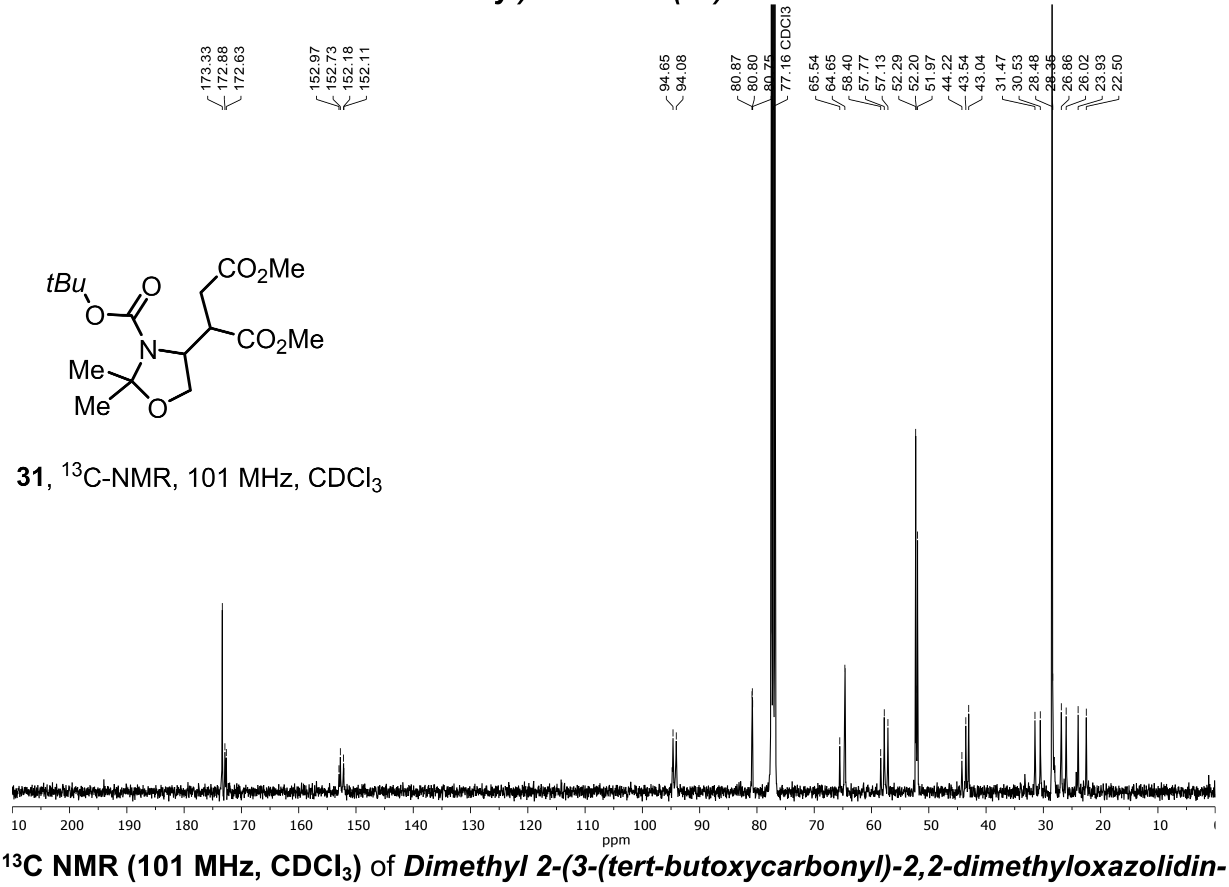

^{13}C NMR (101 MHz, CDCl$_3$) of ***Dimethyl 2-(3-(tert-butoxycarbonyl)-2,2-dimethyloxazolidin-4-yl)succinate (31).***

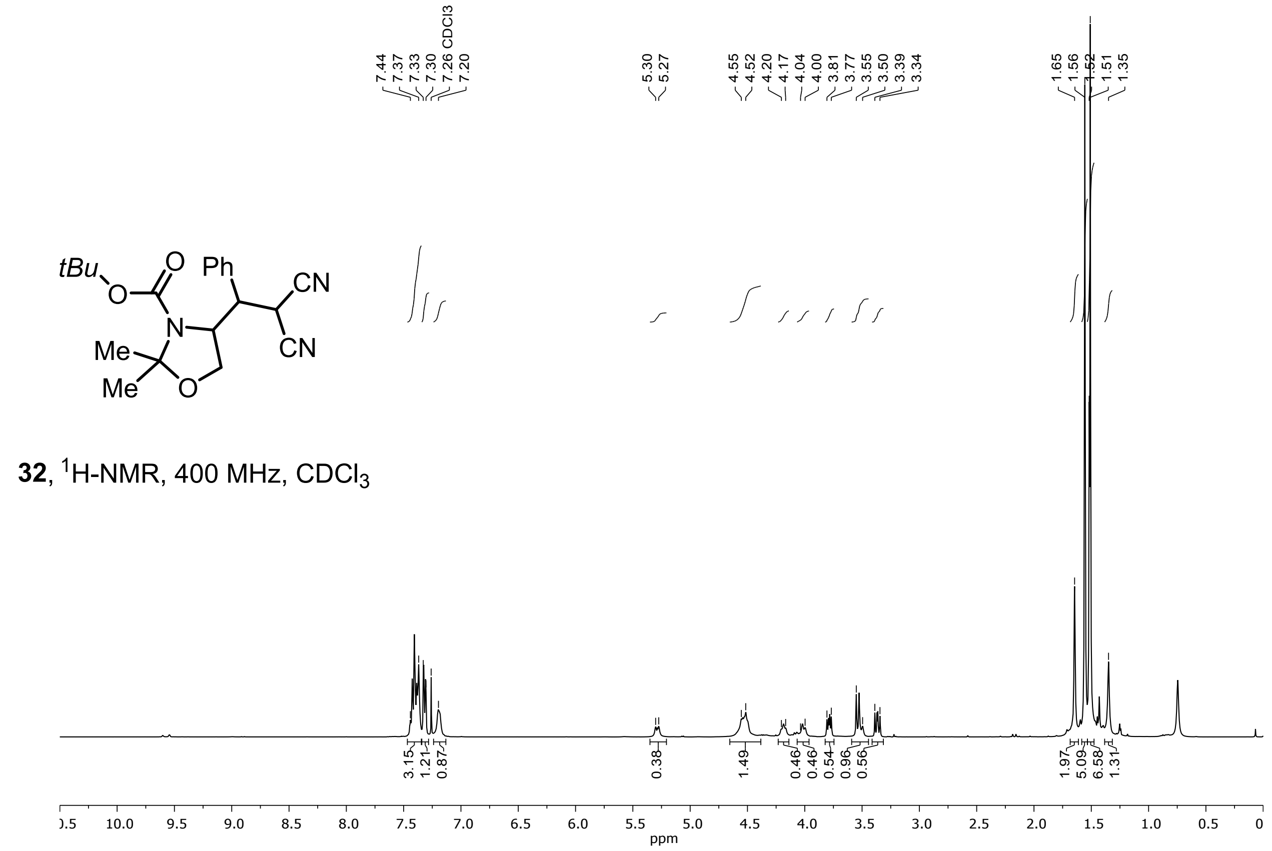

¹H NMR (400 MHz, CDCl$_3$) of ***tert-Butyl 4-(2,2-dicyano-1-phenylethyl)-2,2-dimethyloxazolidine-3-carboxylate (32).***

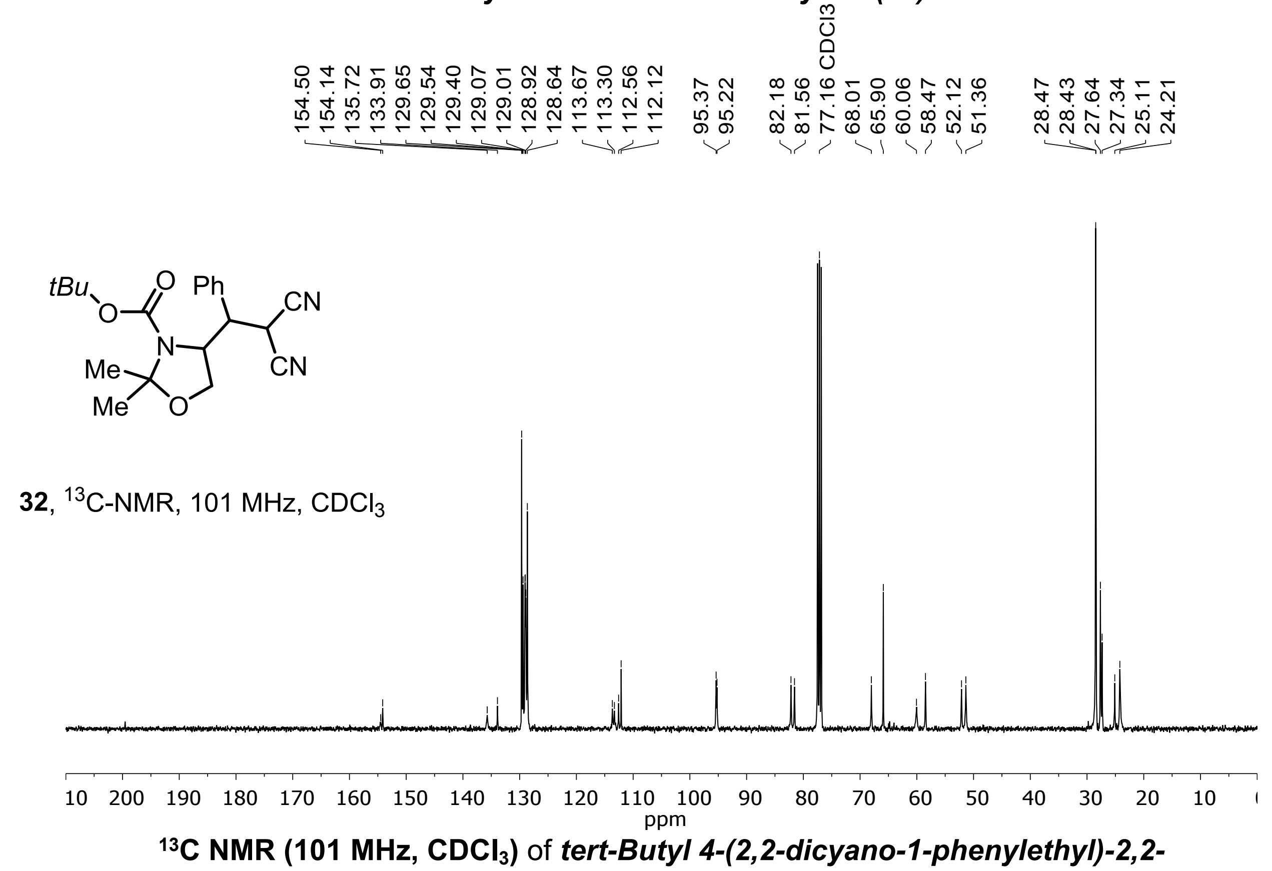

¹³C NMR (101 MHz, CDCl$_3$) of ***tert-Butyl 4-(2,2-dicyano-1-phenylethyl)-2,2-dimethyloxazolidine-3-carboxylate (32).***

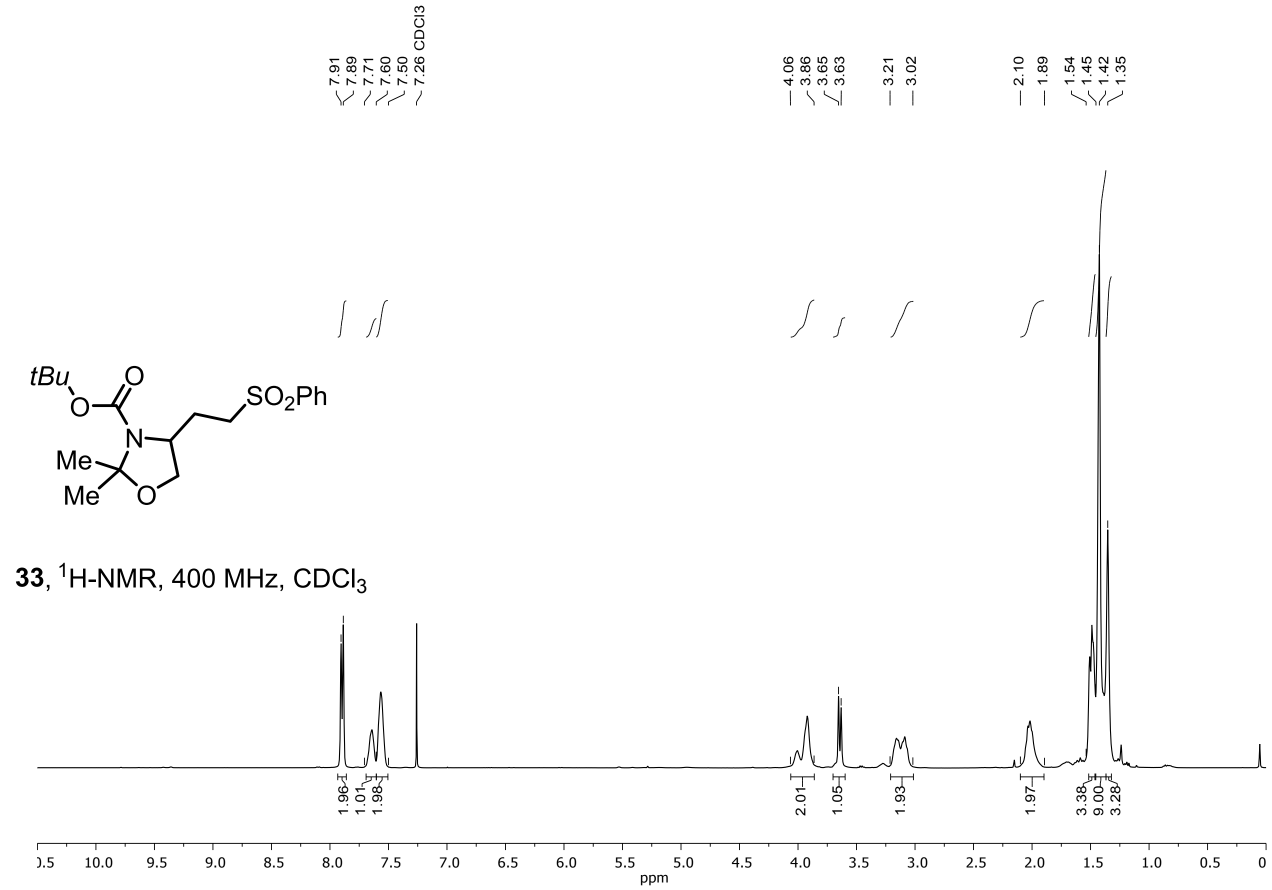

^{1}H NMR (400 MHz, CDCl$_3$) of ***tert-Butyl 2,2-dimethyl-4-(2-(phenylsulfonyl)ethyl)oxazolidine-3-carboxylate (33).***

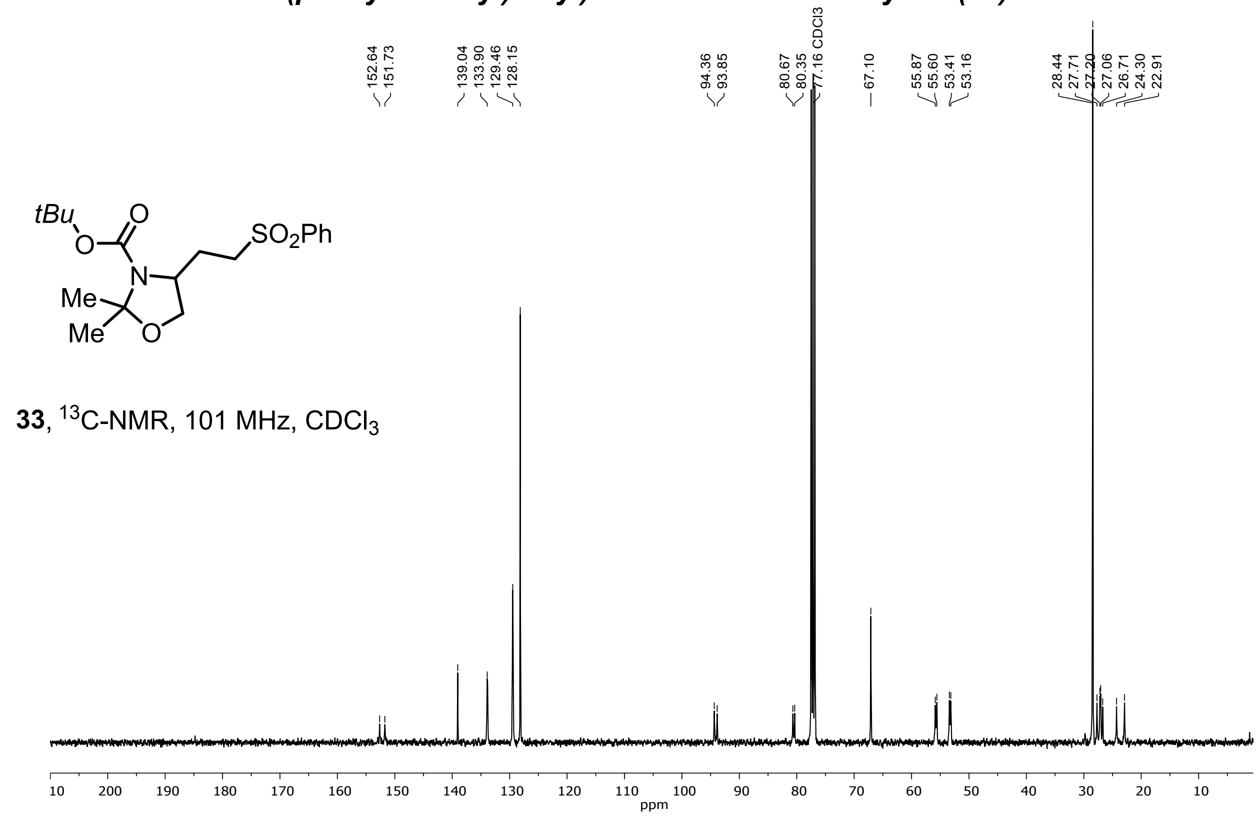

^{13}C NMR (101 MHz, CDCl$_3$) of ***tert-Butyl 2,2-dimethyl-4-(2-(phenylsulfonyl)ethyl)oxazolidine-3-carboxylate (33).***

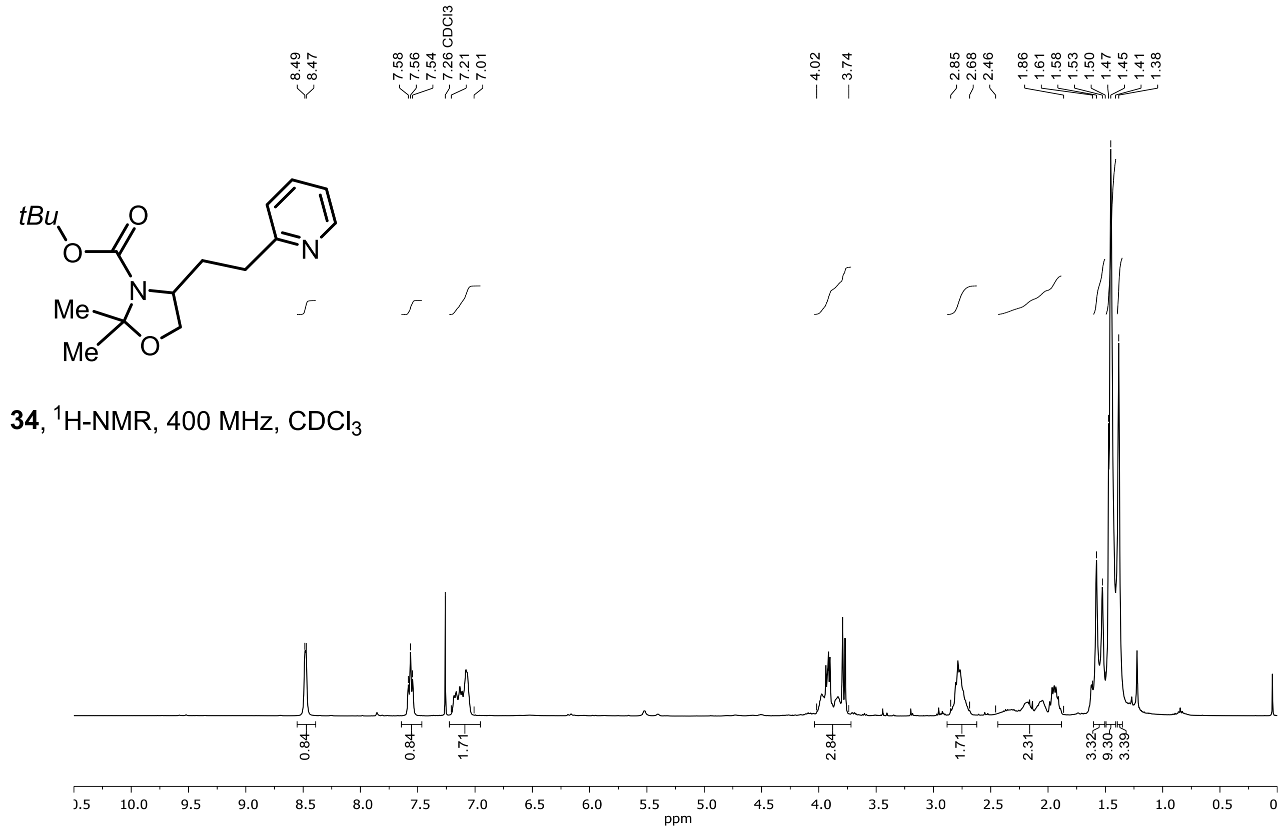

^{1}H NMR (400 MHz, CDCl$_3$) of ***tert-Butyl 2,2-dimethyl-4-(2-(pyridin-2-yl)ethyl)oxazolidine-3-carboxylate (34).***

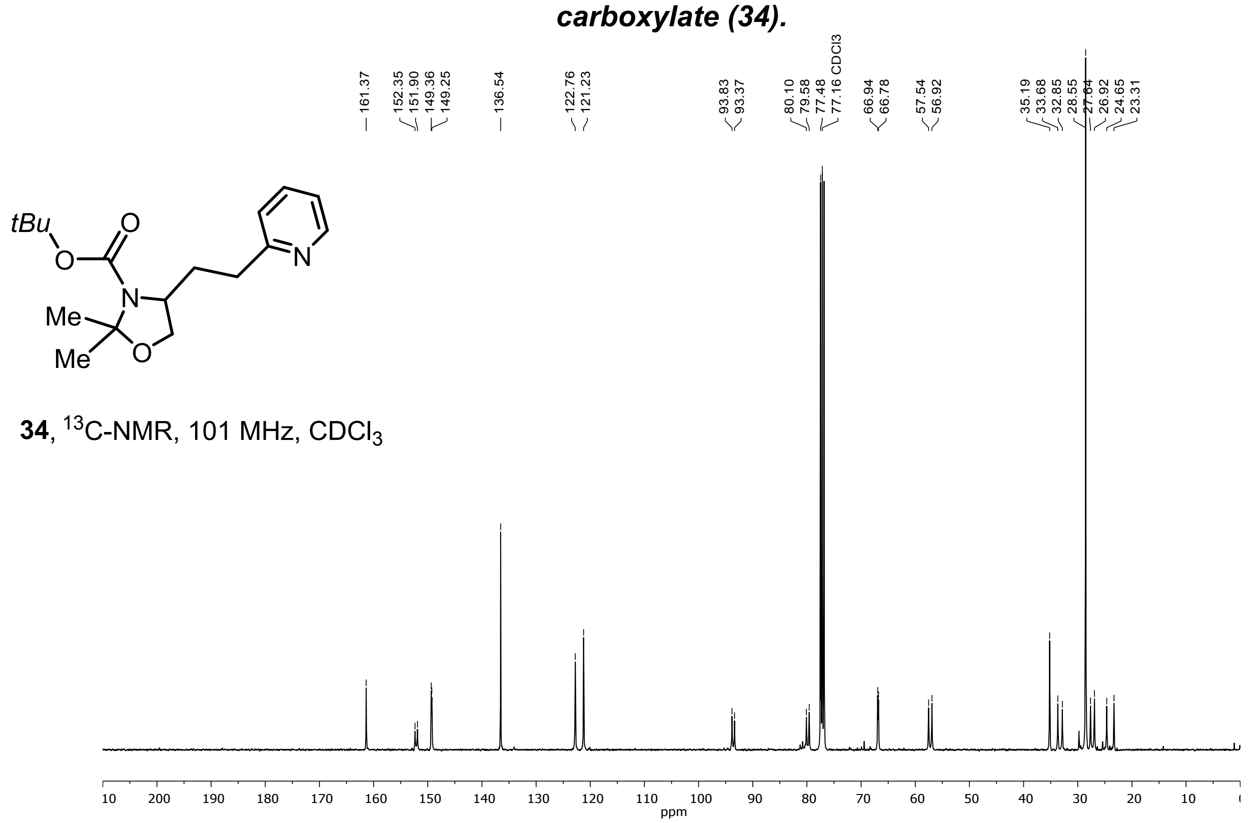

^{13}C NMR (101 MHz, CDCl$_3$) of ***tert-Butyl 2,2-dimethyl-4-(2-(pyridin-2-yl)ethyl)oxazolidine-3-carboxylate (34).***

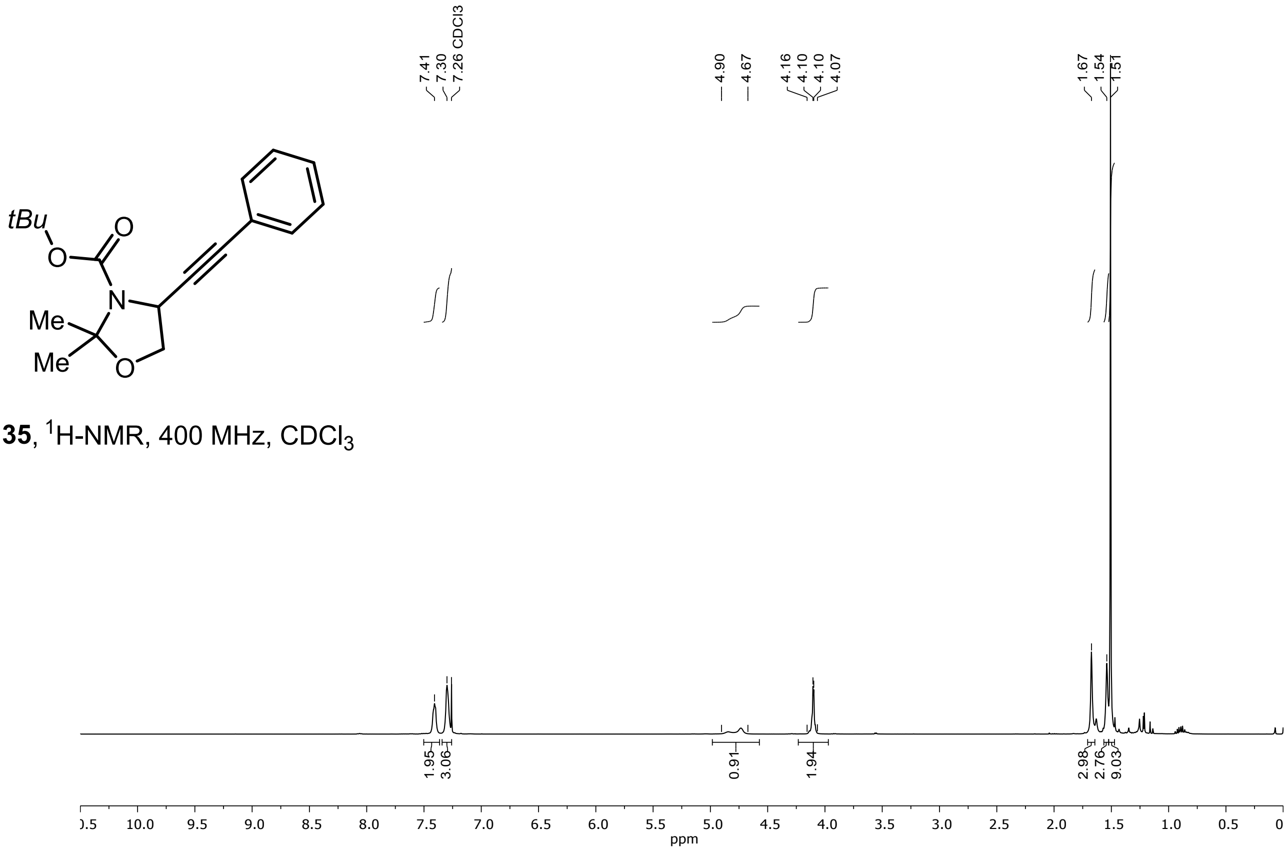

^{1}H NMR (400 MHz, CDCl$_3$) of ***tert-Butyl 2,2-dimethyl-4-(phenylethynyl)oxazolidine-3-carboxylate (35).***

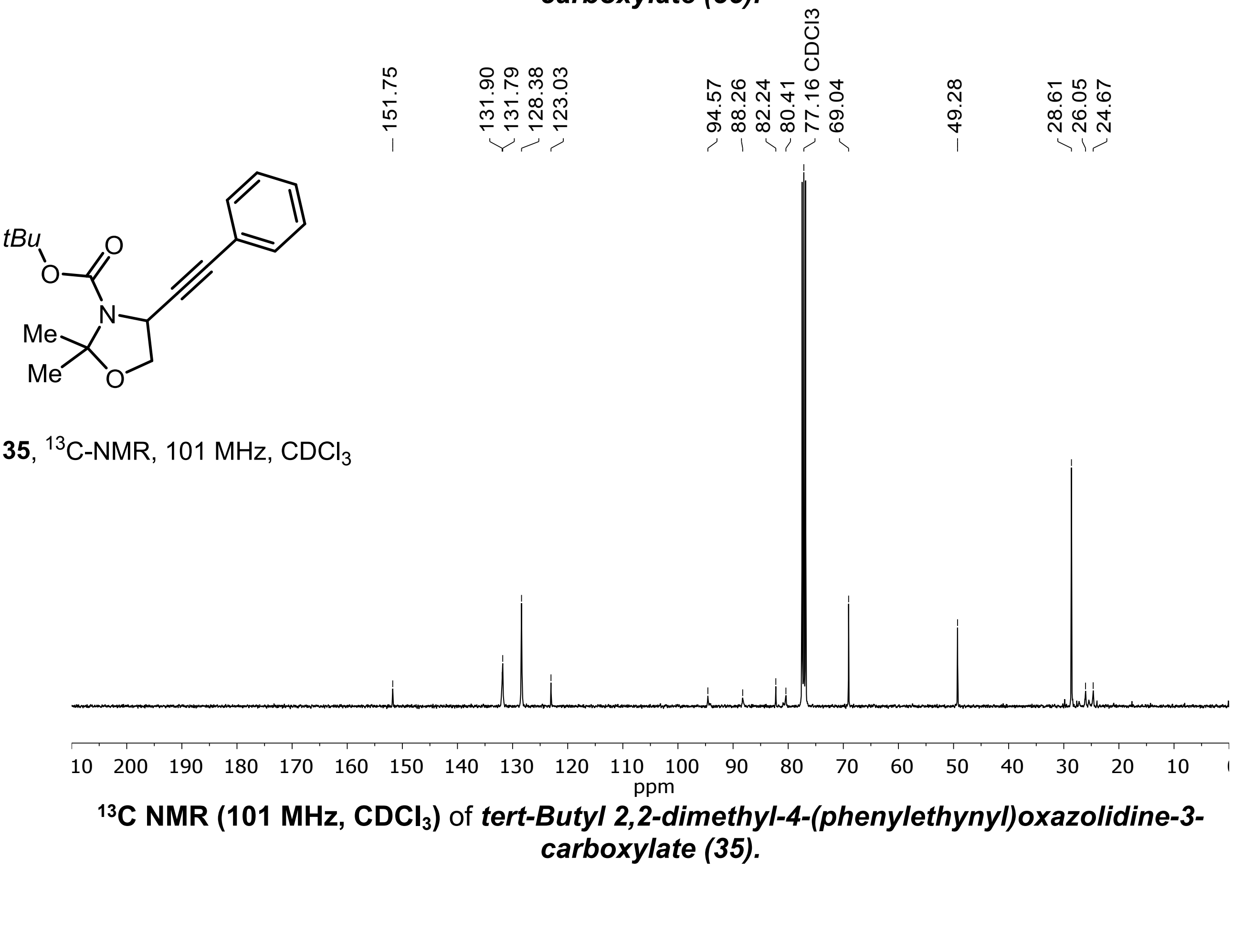

^{13}C NMR (101 MHz, CDCl$_3$) of ***tert-Butyl 2,2-dimethyl-4-(phenylethynyl)oxazolidine-3-carboxylate (35).***

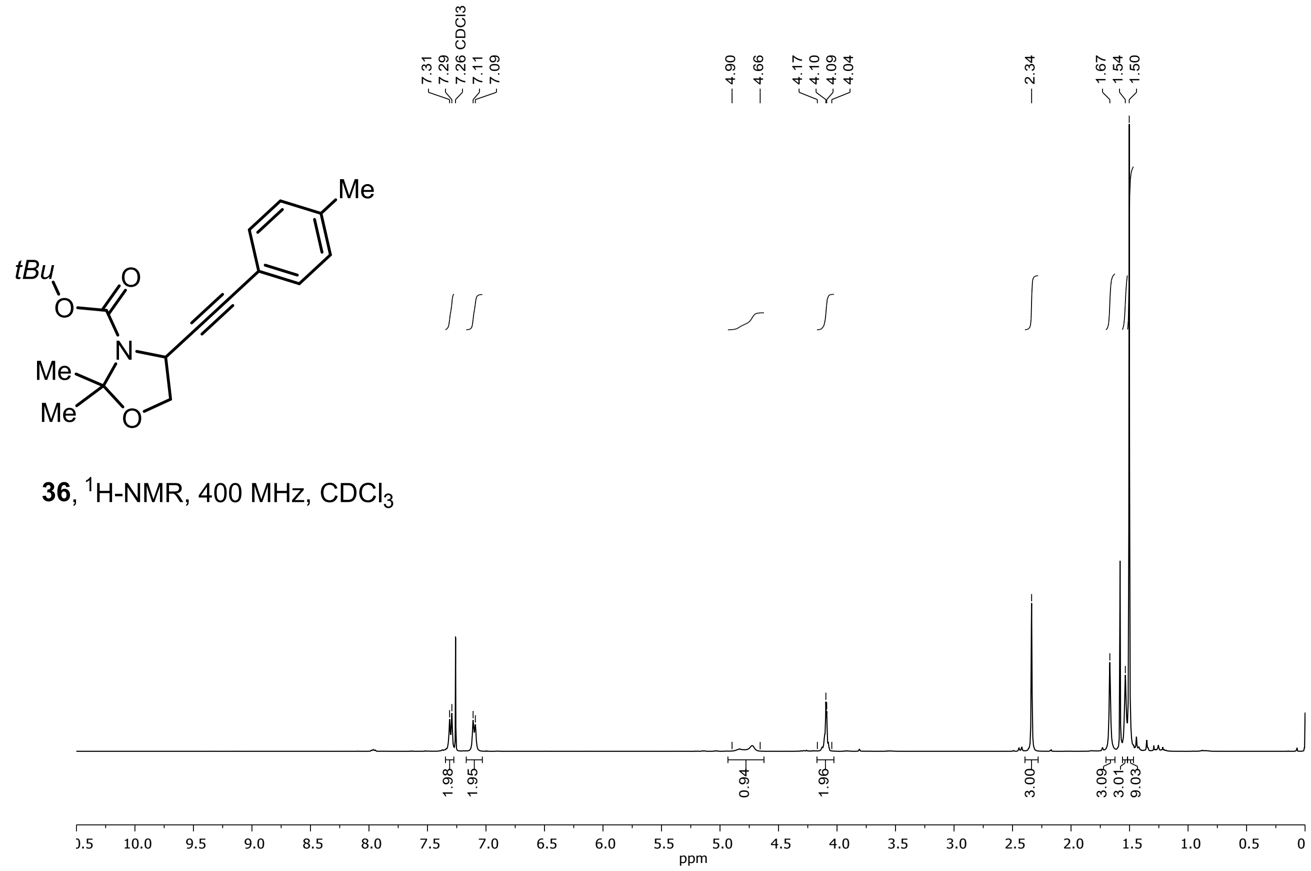

^{1}H NMR (400 MHz, $CDCl_3$) of ***tert-Butyl 2,2-dimethyl-4-(p-tolylethynyl)oxazolidine-3-carboxylate (36).***

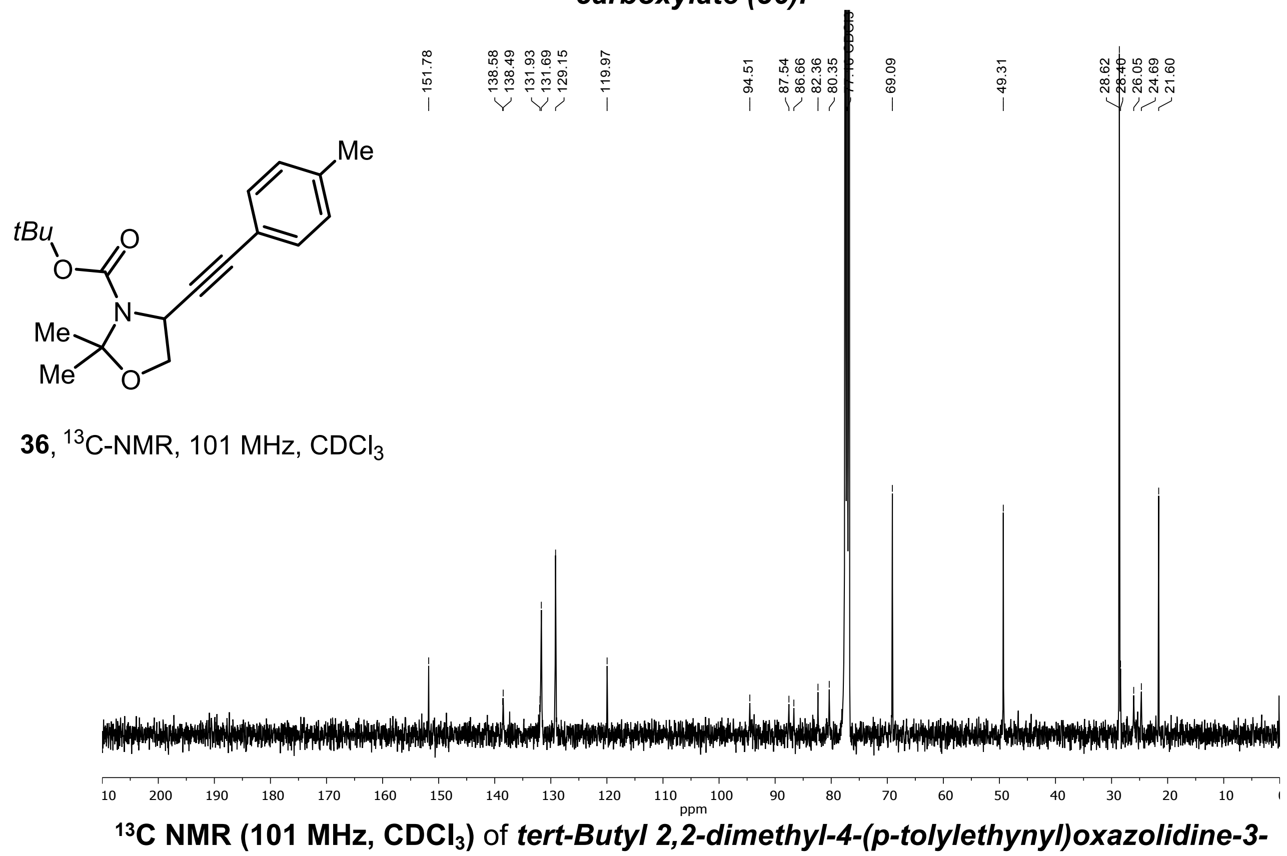

^{13}C NMR (101 MHz, $CDCl_3$) of ***tert-Butyl 2,2-dimethyl-4-(p-tolylethynyl)oxazolidine-3-carboxylate (36).***

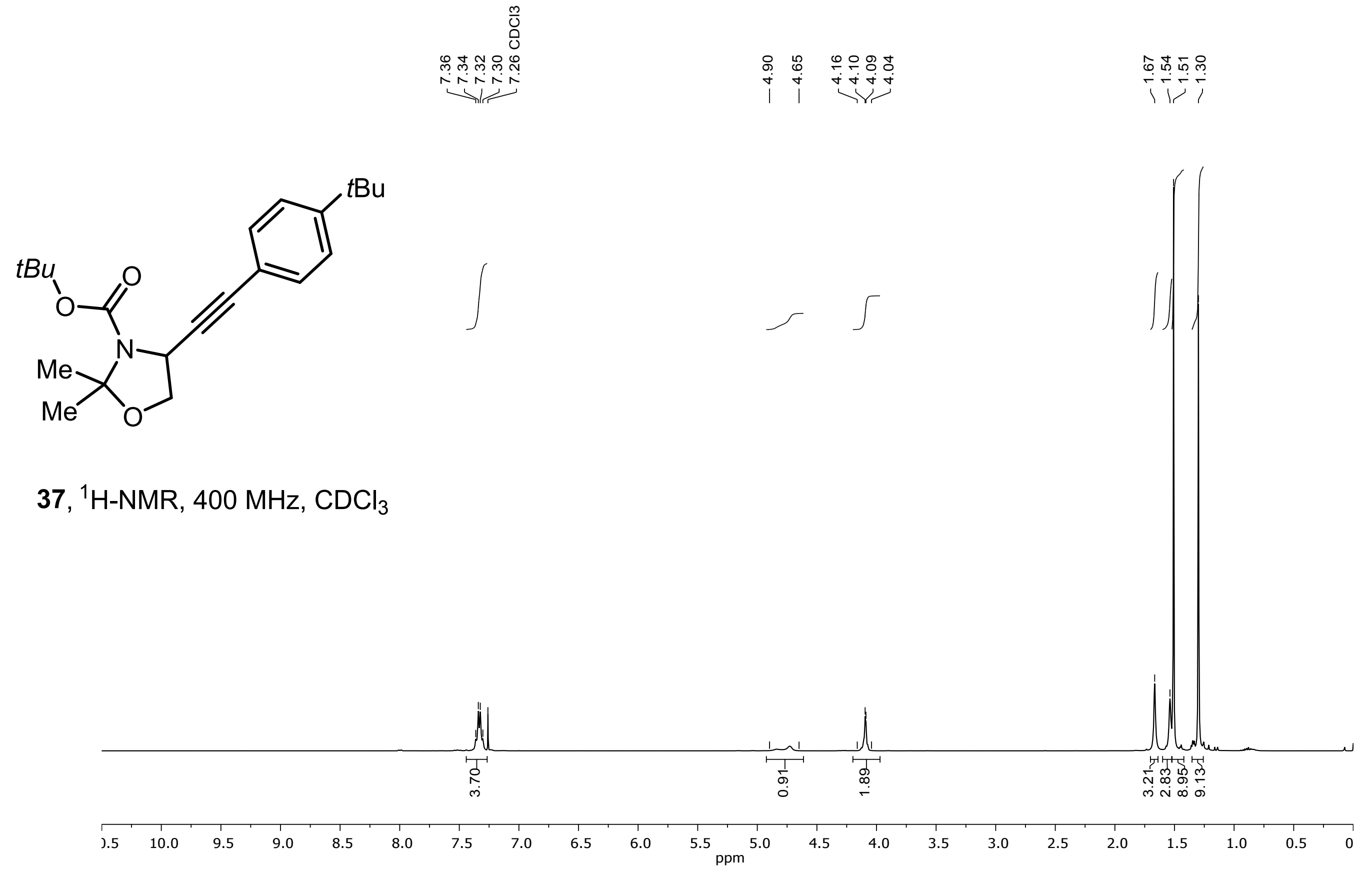

^{1}H NMR (400 MHz, CDCl$_3$) of ***tert-Butyl 4-((4-(tert-butyl)phenyl)ethynyl)-2,2-dimethyloxazolidine-3-carboxylate (37).***

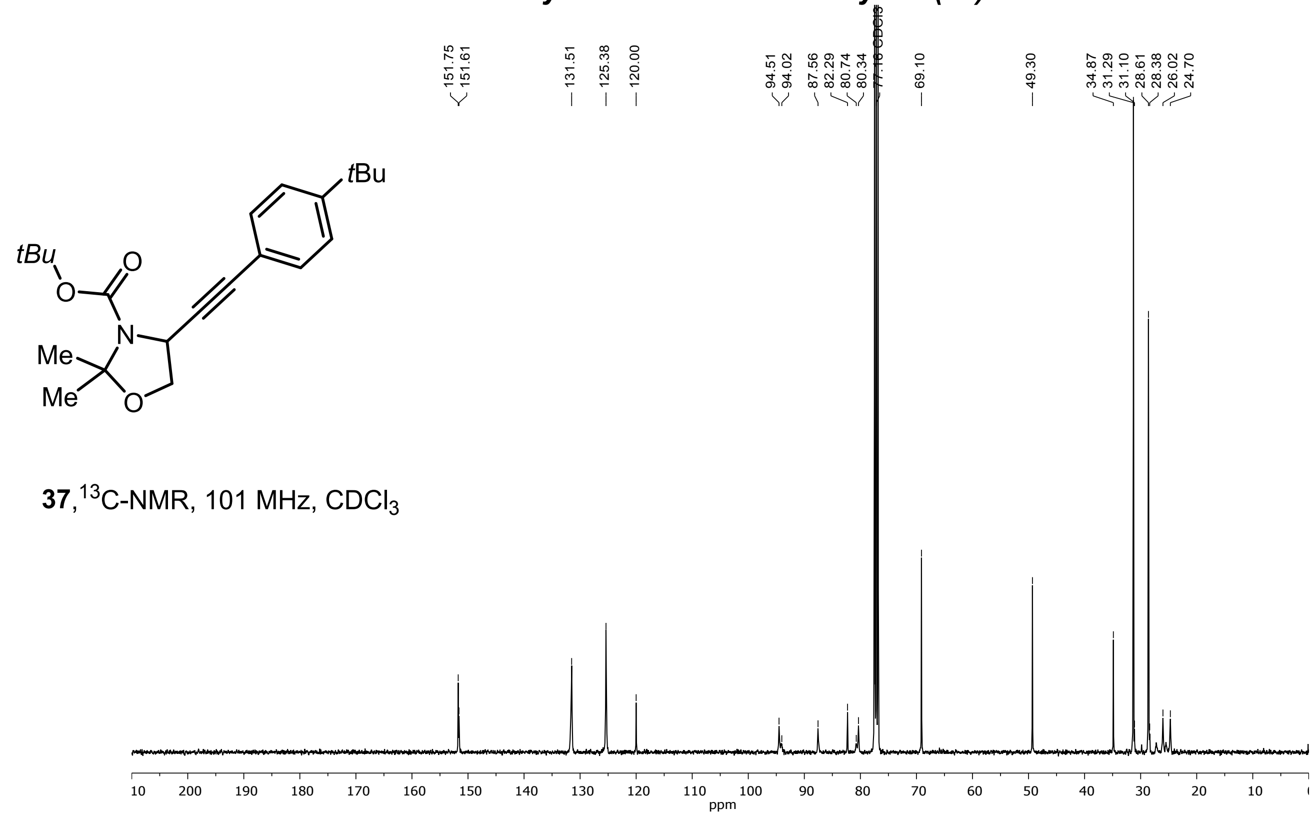

^{13}C NMR (101 MHz, CDCl$_3$) of ***tert-Butyl 4-((4-(tert-butyl)phenyl)ethynyl)-2,2-dimethyloxazolidine-3-carboxylate (37).***

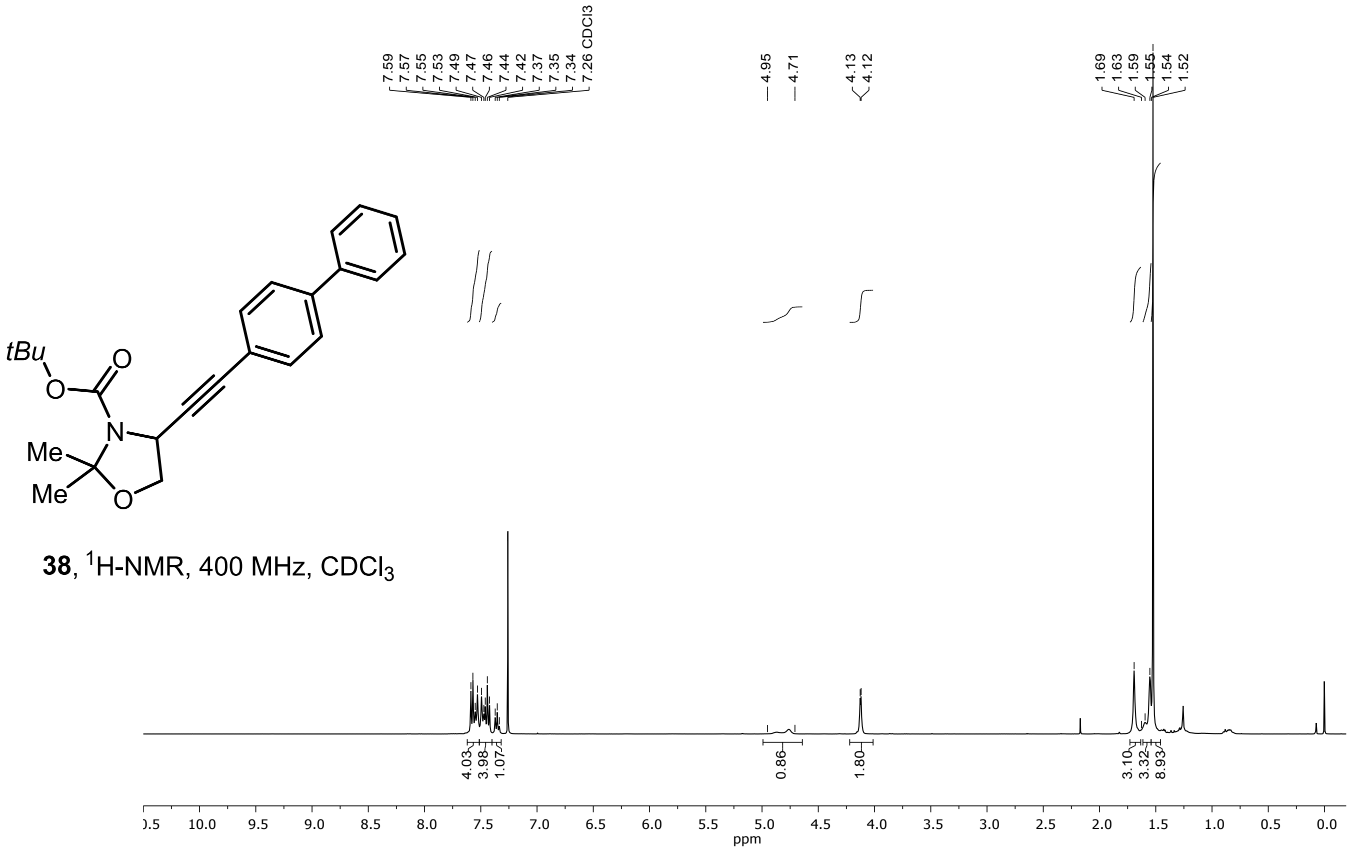

^{1}H NMR (400 MHz, $CDCl_3$) of ***tert-Butyl 4-((1,1'-biphenyl)-4-ylethynyl)-2,2-dimethyloxazolidine-3-carboxylate (38).***

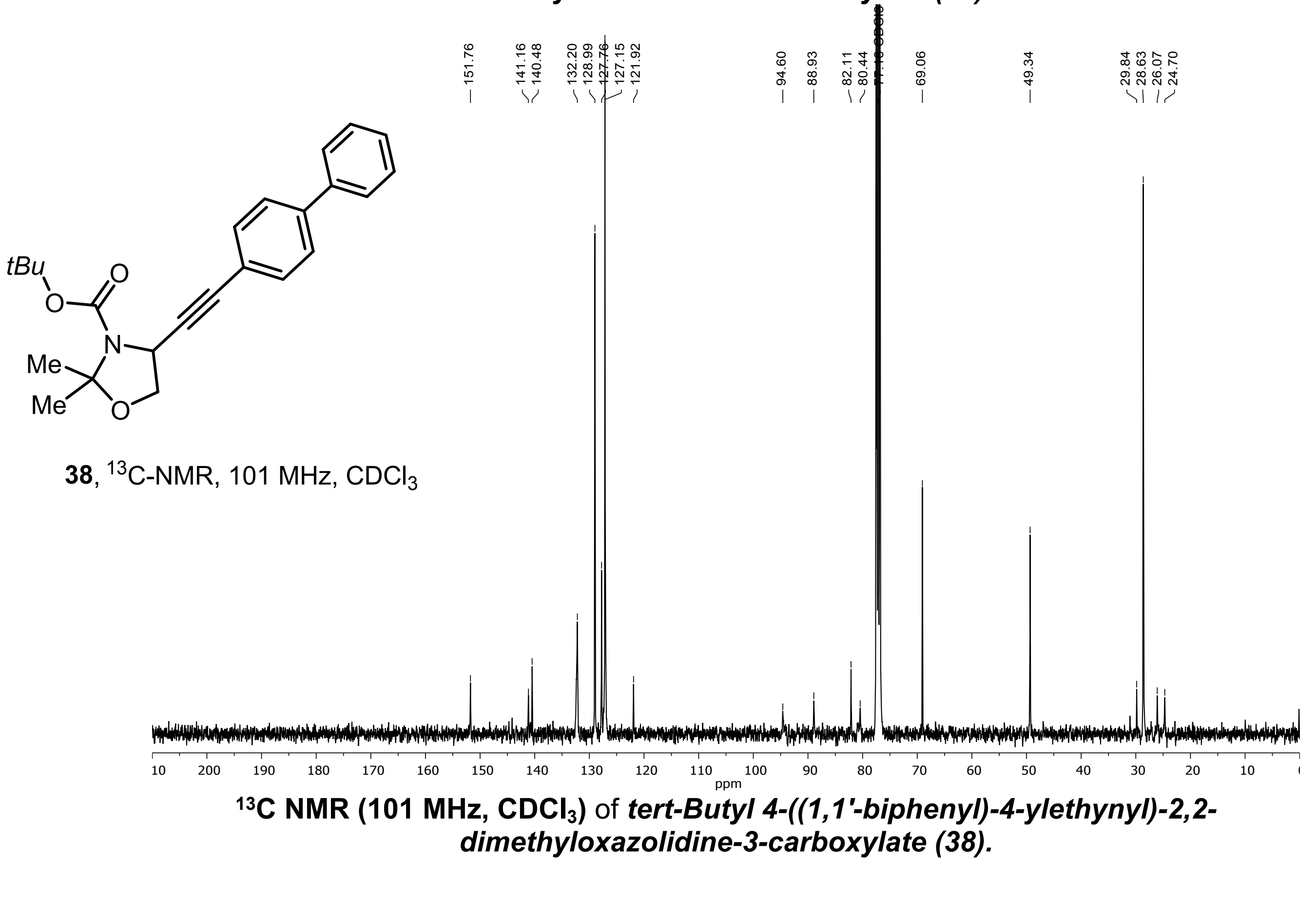

^{13}C NMR (101 MHz, $CDCl_3$) of ***tert-Butyl 4-((1,1'-biphenyl)-4-ylethynyl)-2,2-dimethyloxazolidine-3-carboxylate (38).***

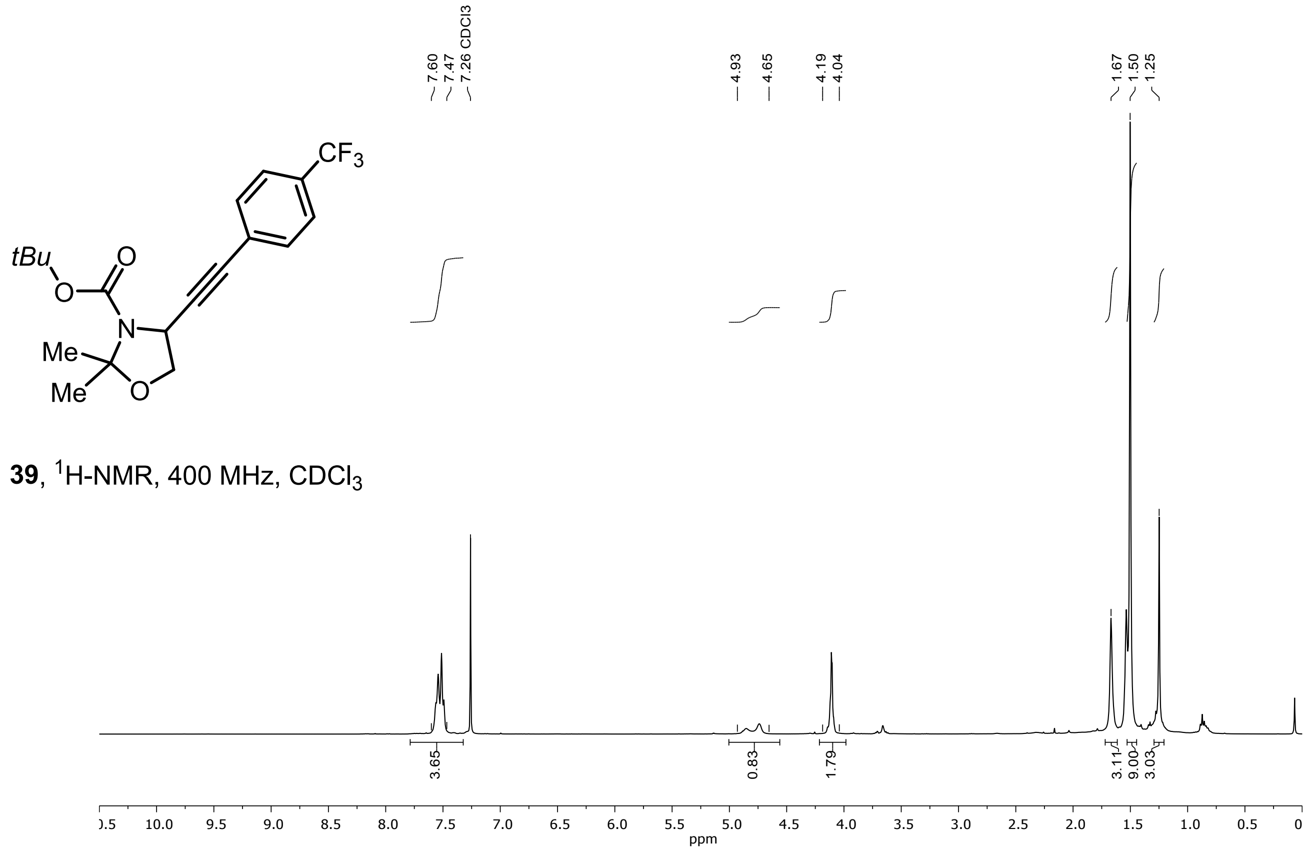

^{1}H NMR (400 MHz, $CDCl_3$) of ***tert-Butyl 2,2-dimethyl-4-((4-(trifluoromethyl)phenyl)ethynyl)oxazolidine-3-carboxylate (39).***

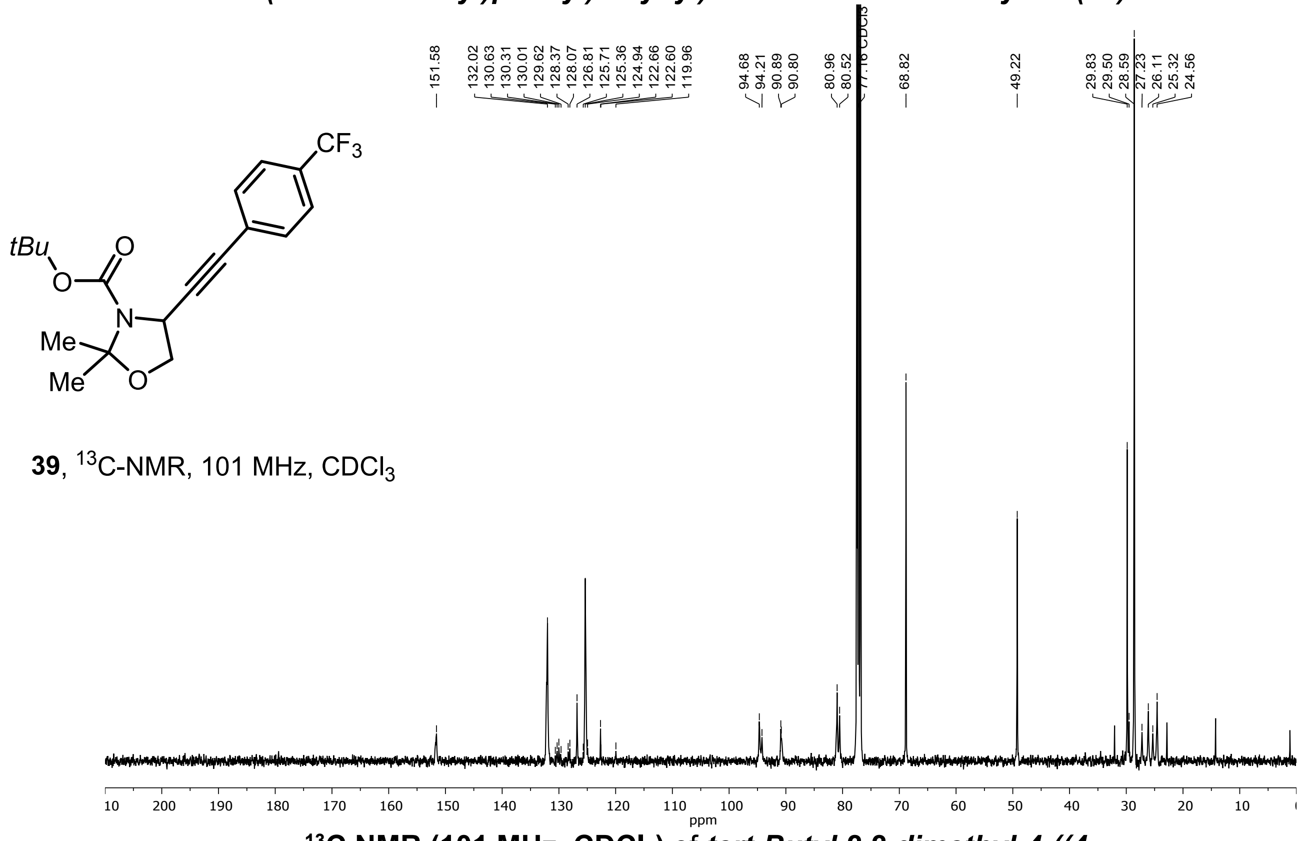

^{13}C NMR (101 MHz, $CDCl_3$) of ***tert-Butyl 2,2-dimethyl-4-((4-(trifluoromethyl)phenyl)ethynyl)oxazolidine-3-carboxylate (39).***

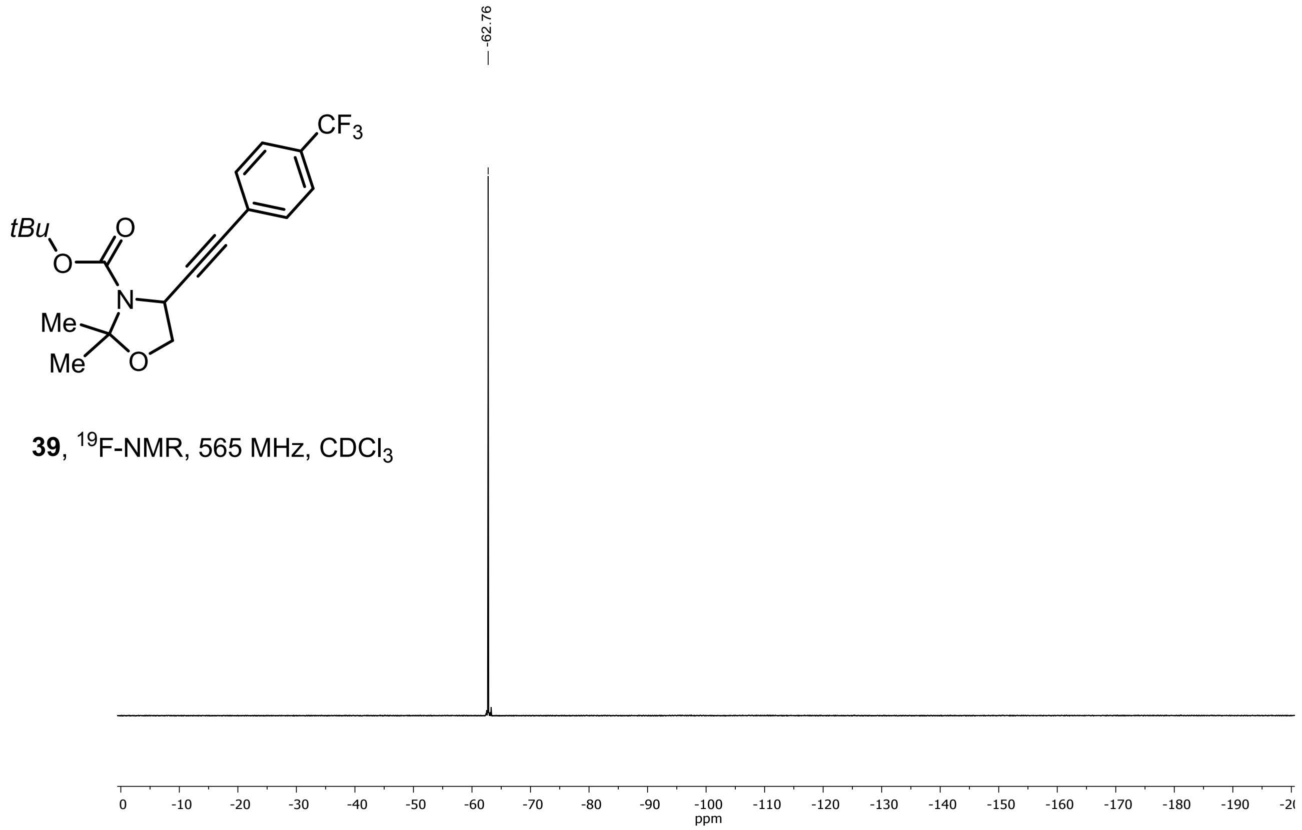

^{19}F NMR (565 MHz, $CDCl_3$) of ***tert-Butyl 2,2-dimethyl-4-((4-(trifluoromethyl)phenyl)ethynyl)oxazolidine-3-carboxylate (39).***

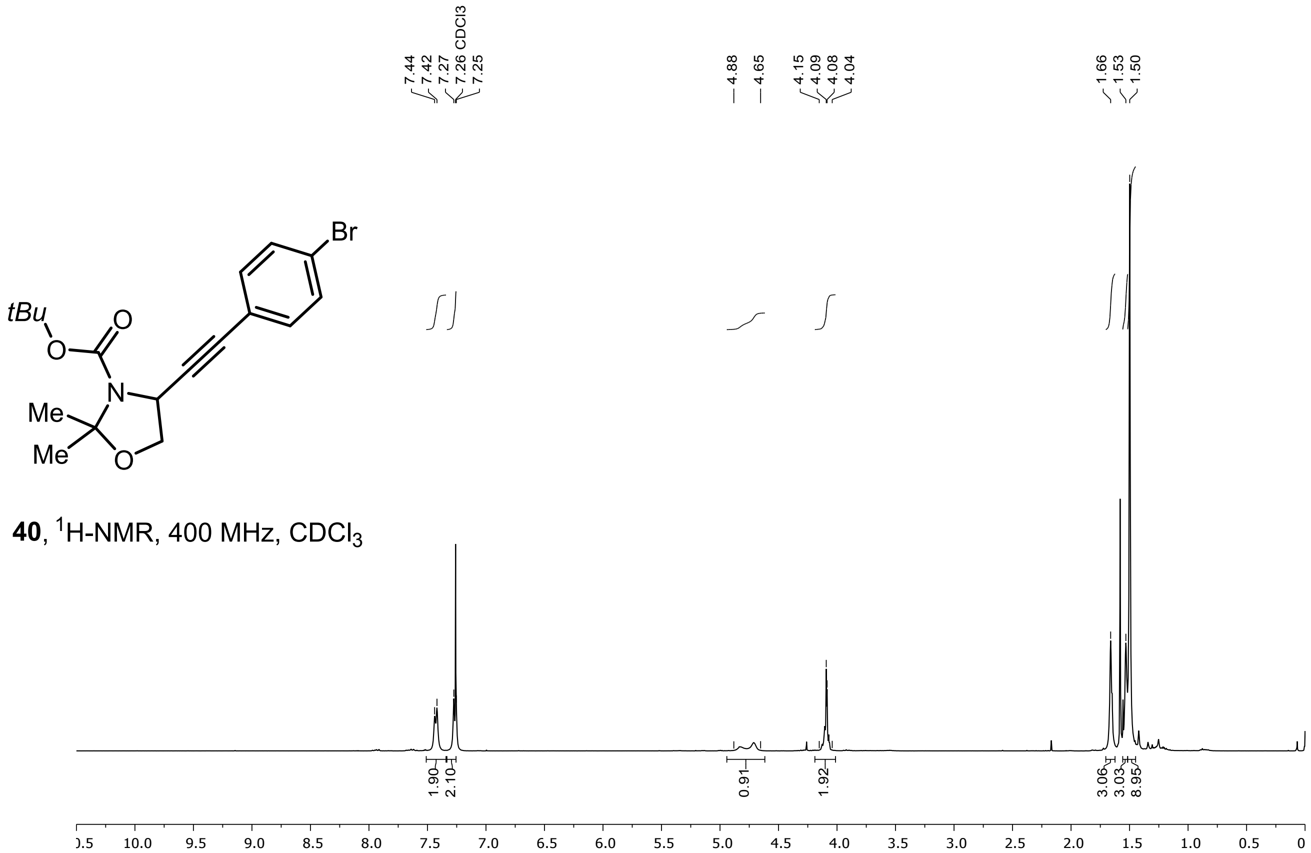

^{1}H NMR (400 MHz, $CDCl_3$) of ***tert-Butyl 4-((4-bromophenyl)ethynyl)-2,2-dimethyloxazolidine-3-carboxylate (40).***

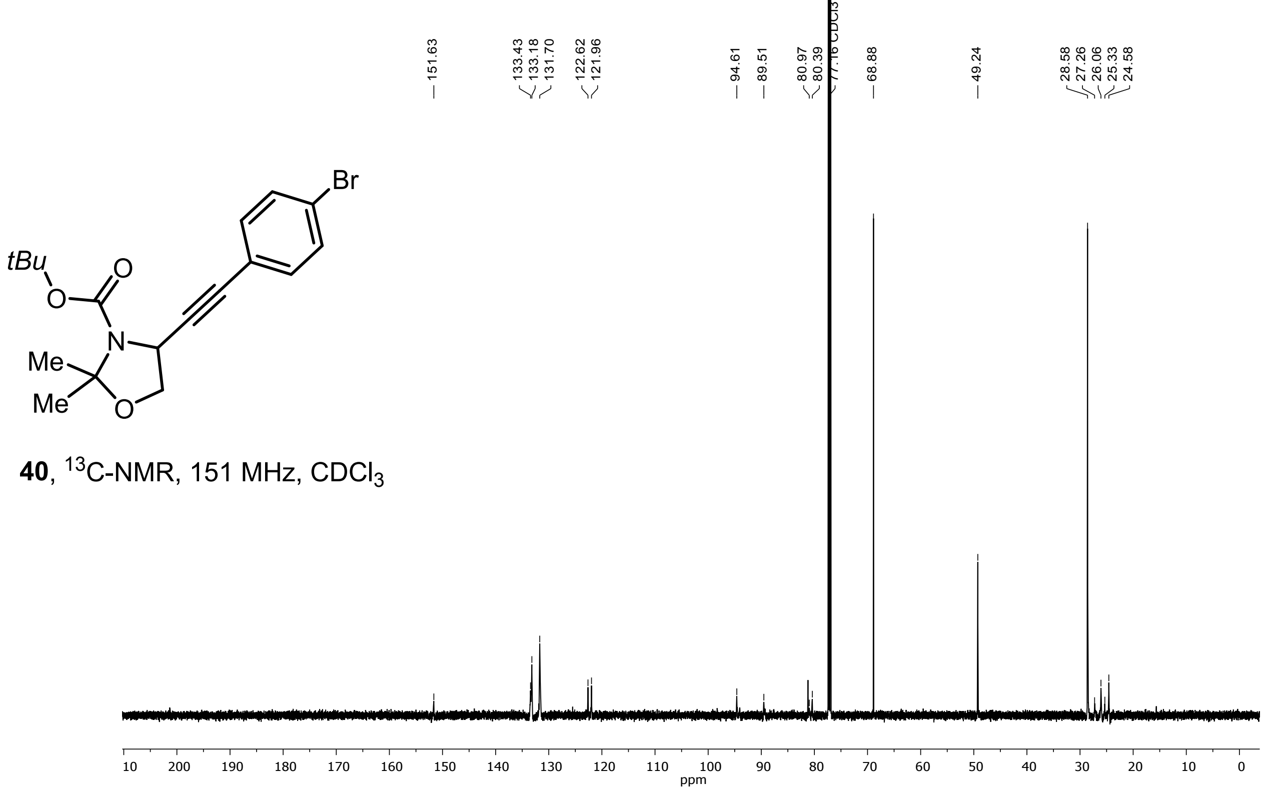

^{13}C NMR (151 MHz, $CDCl_3$) of ***tert-Butyl 4-((4-bromophenyl)ethynyl)-2,2-dimethyloxazolidine-3-carboxylate (40).***

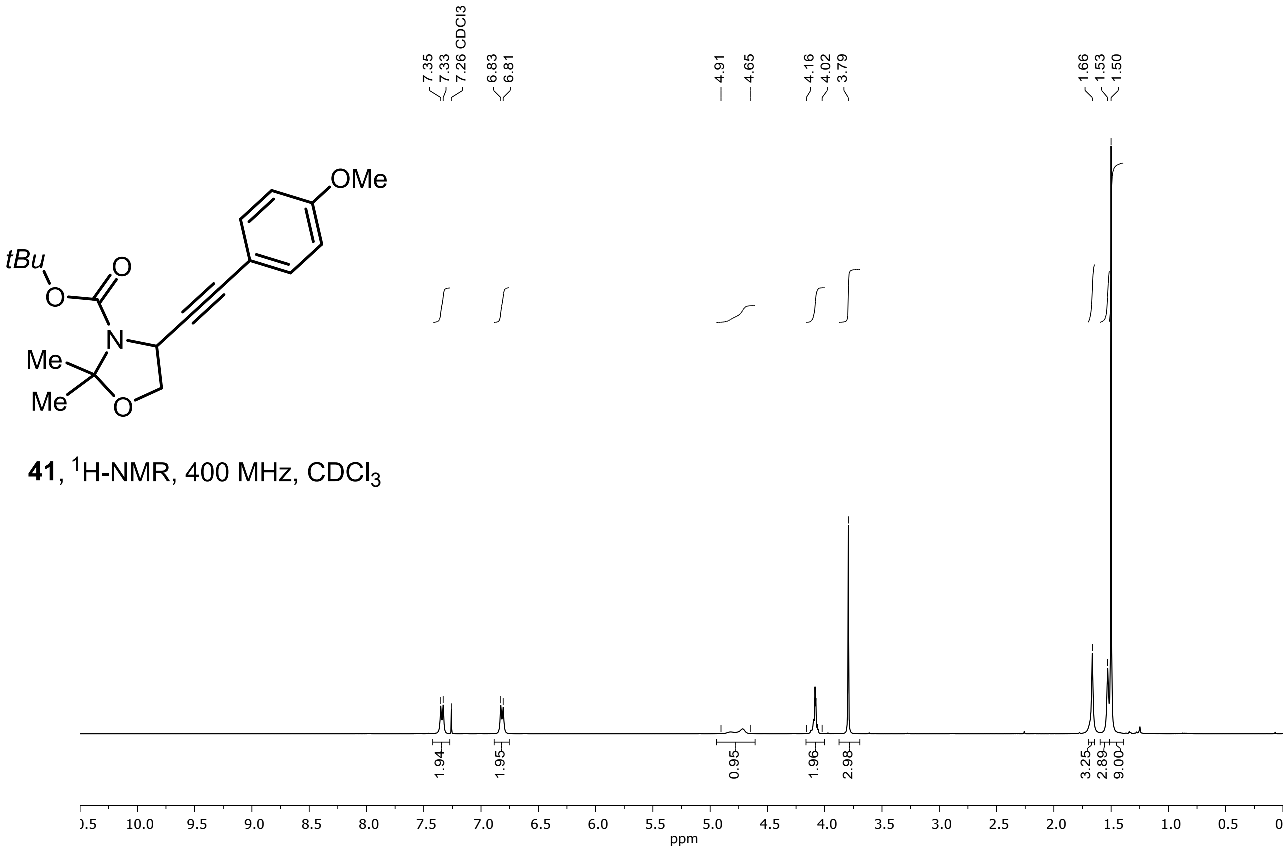

^{1}H NMR (400 MHz, CDCl$_3$) of ***tert-Butyl 4-((4-methoxyphenyl)ethynyl)-2,2-dimethyloxazolidine-3-carboxylate (41).***

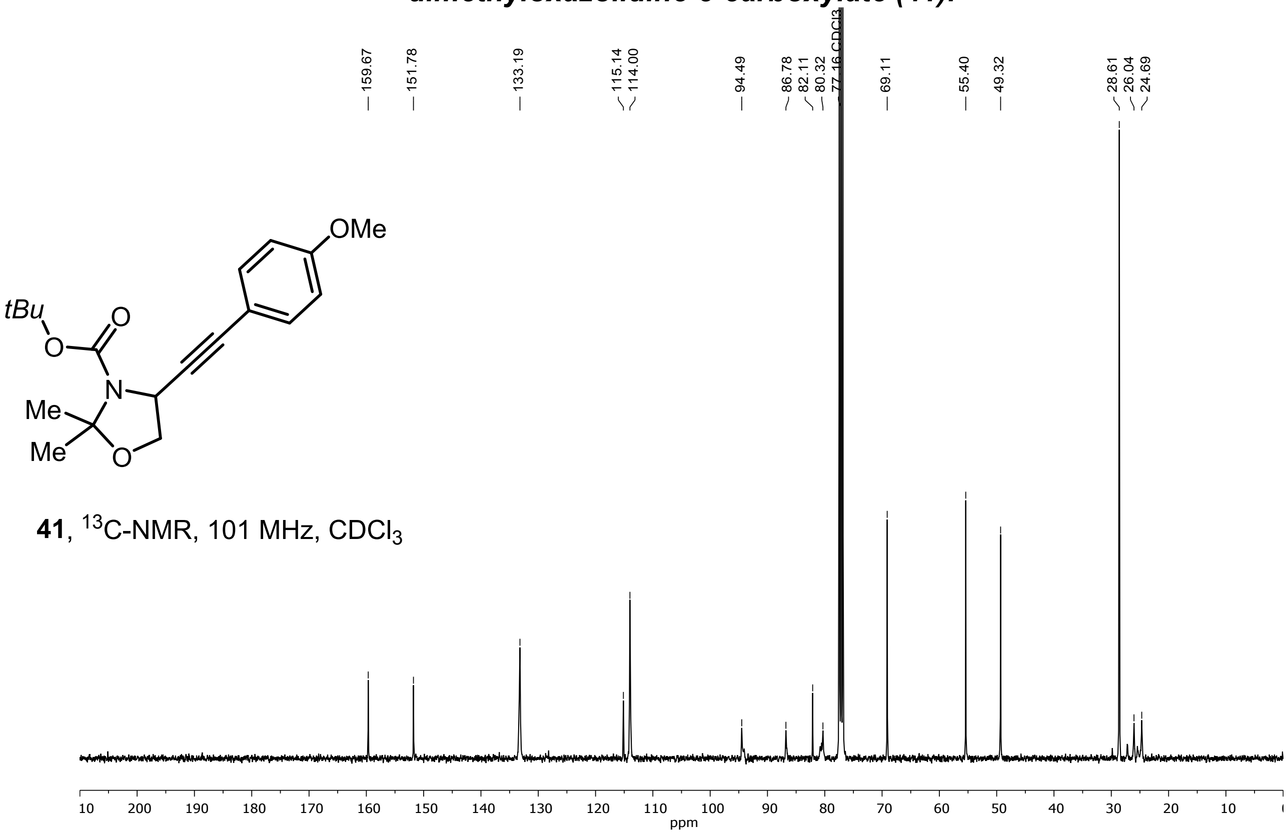

^{13}C NMR (101 MHz, CDCl$_3$) of ***tert-Butyl 4-((4-methoxyphenyl)ethynyl)-2,2-dimethyloxazolidine-3-carboxylate (41).***

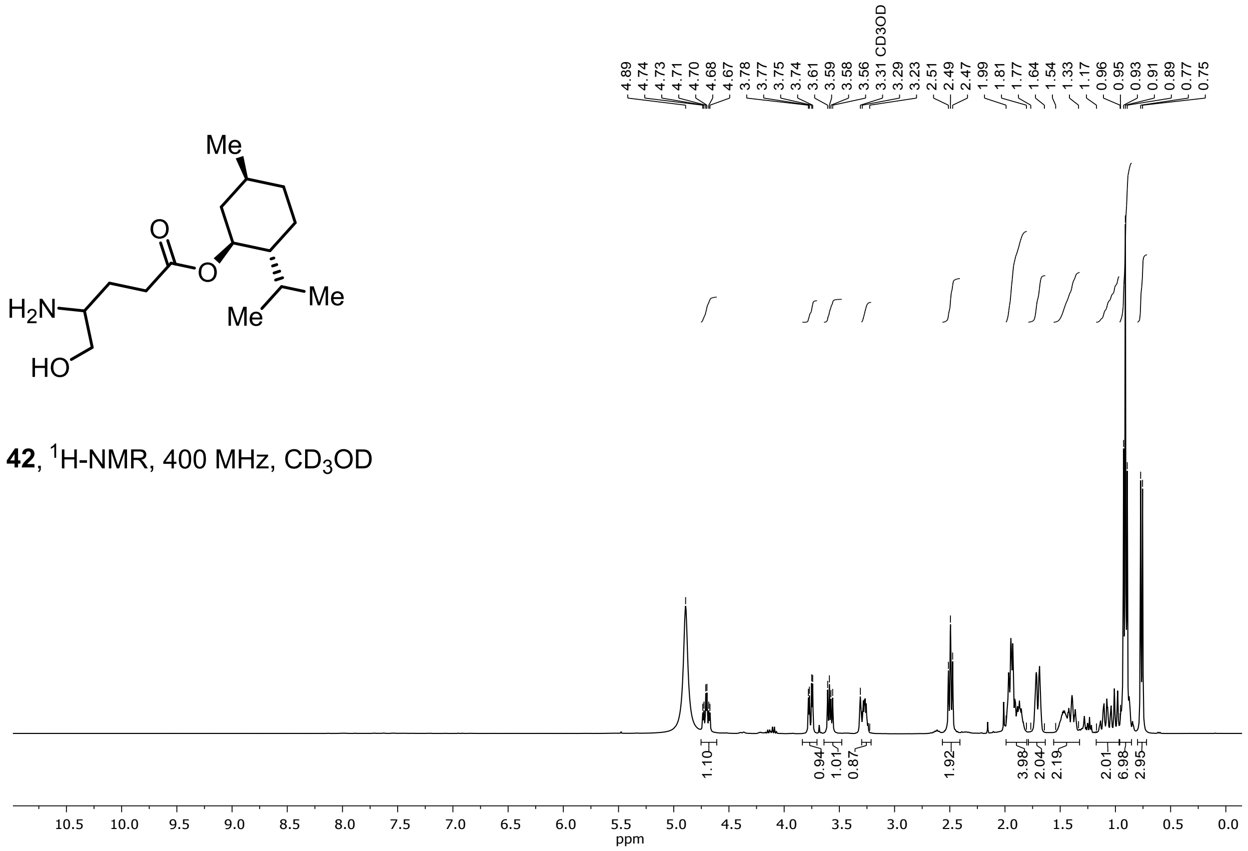

^{1}H NMR (400 MHz, CD_3OD) of ***(1S,2R,5S)-2-Isopropyl-5-methylcyclohexyl 4-amino-5-hydroxypentanoate (42).***

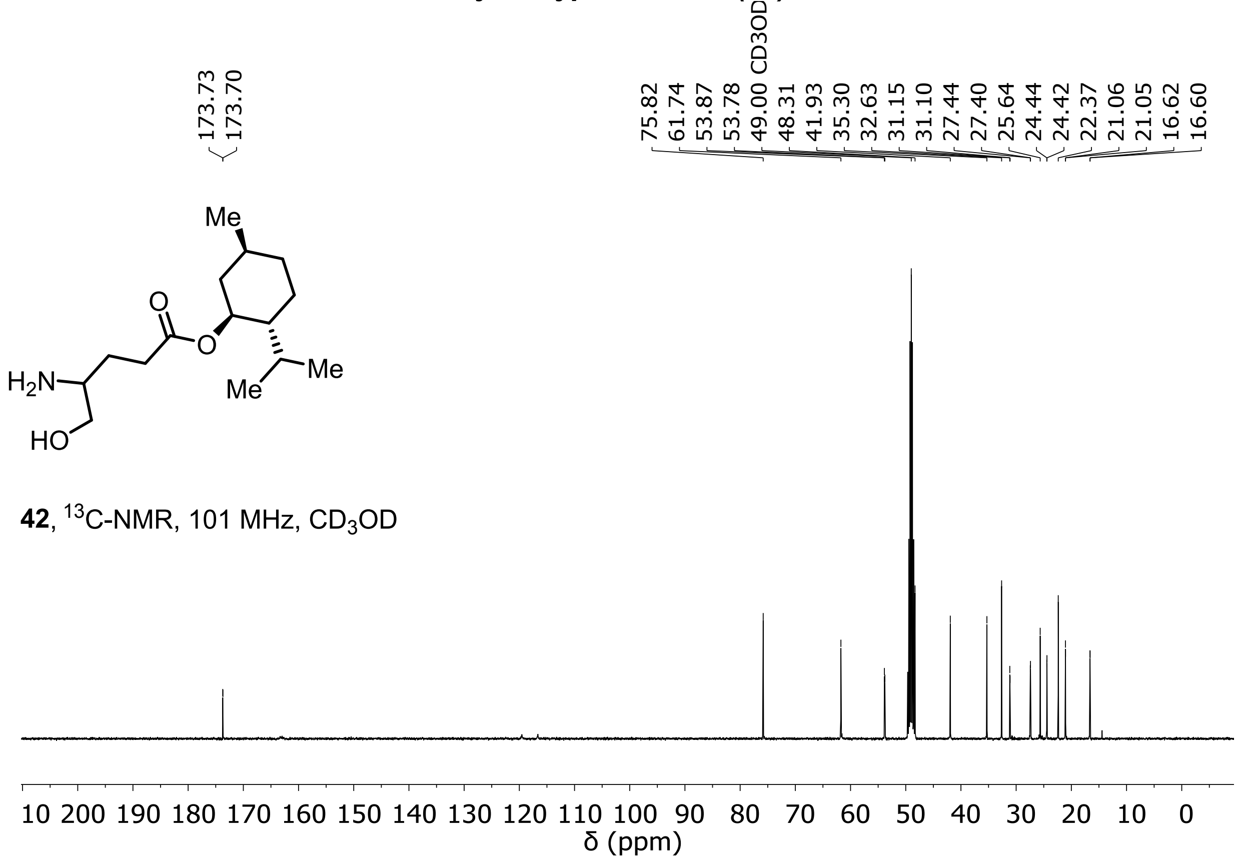

^{13}C NMR (101 MHz, CD_3OD) of **(1S,2R,5S)-2-Isopropyl-5-methylcyclohexyl 4-amino-5-hydroxypentanoate (*42*).**

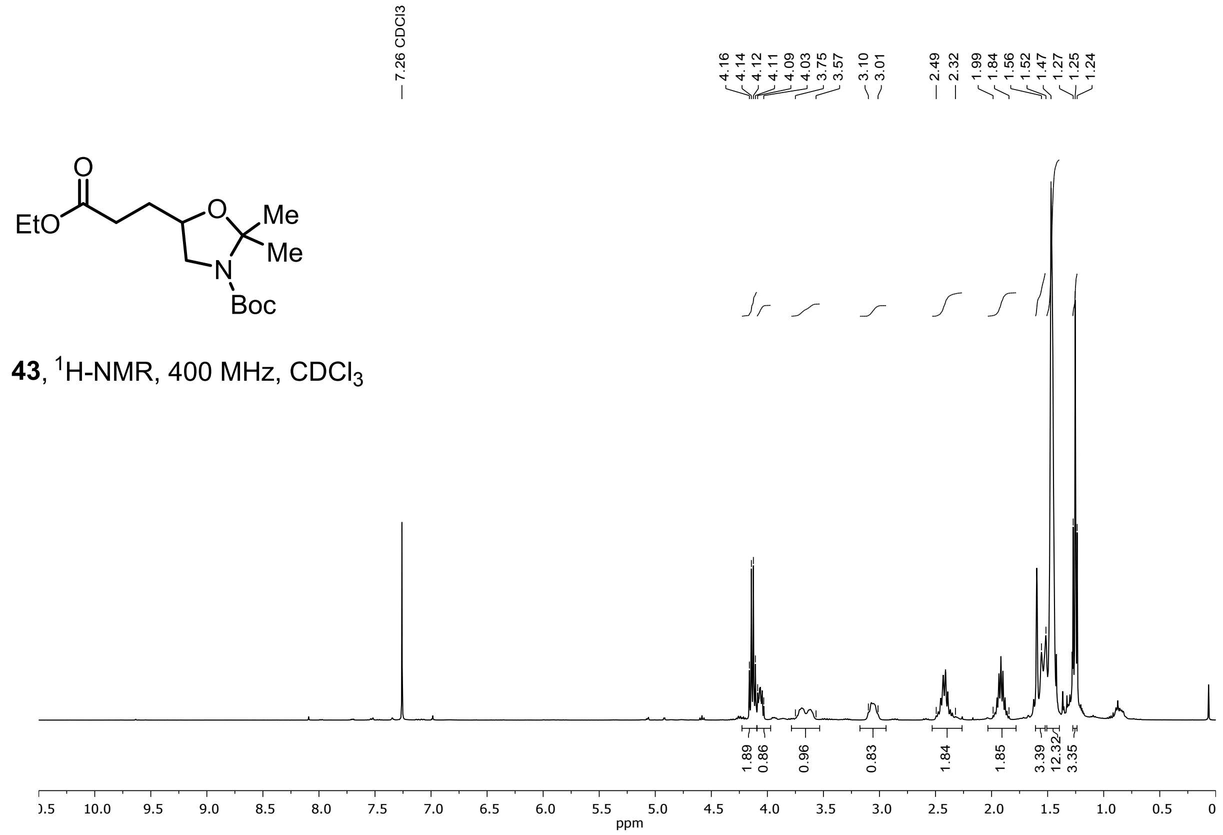

^{1}H NMR (400 MHz, CDCl$_3$) *of* ***tert-butyl 5-(3-ethoxy-3-oxopropyl)-2,2-dimethyloxazolidine-3-carboxylate (43).***

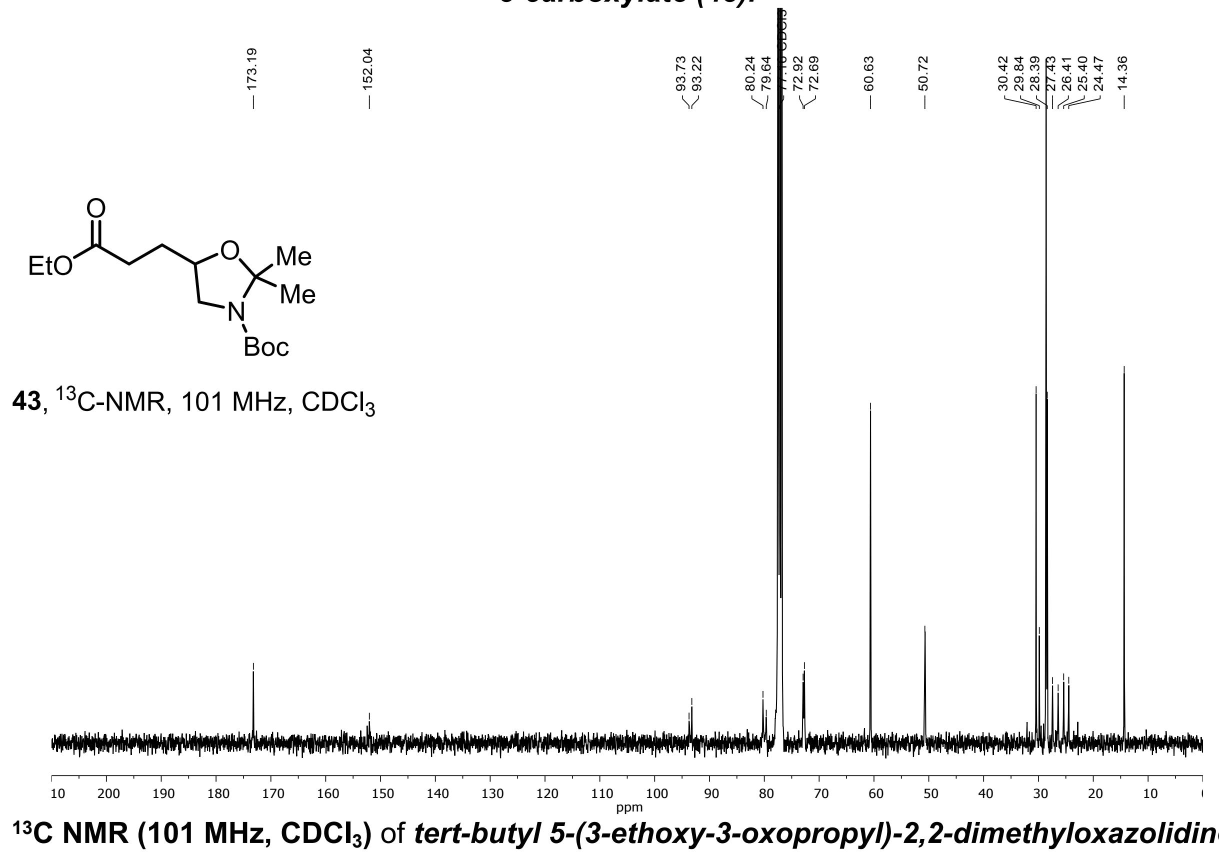

^{13}C NMR (101 MHz, CDCl$_3$) of ***tert-butyl 5-(3-ethoxy-3-oxopropyl)-2,2-dimethyloxazolidine-3-carboxylate (43).***
